# ﻿Phylogenomic inference of the African tribe Monodoreae (Annonaceae) and taxonomic revision of *Dennettia*, *Uvariodendron* and *Uvariopsis*

**DOI:** 10.3897/phytokeys.233.103096

**Published:** 2023-09-22

**Authors:** Léo-Paul M. J. Dagallier, Frank M. Mbago, Marie Couderc, Myriam Gaudeul, Aurélie Grall, Caroline Loup, Jan J. Wieringa, Bonaventure Sonké, Thomas L. P. Couvreur

**Affiliations:** 1 DIADE, Université de Montpellier, IRD, CIRAD, Montpellier, France; 2 Institute of Systematic Botany, The New York Botanical Garden, Bronx, New York 10458, USA; 3 The Herbarium, Botany Department, Box 35060, University of Dar es Salaam, Dar es Salaam, Tanzania; 4 Institut de Systématique, Evolution, Biodiversité (ISYEB), Muséum National d’Histoire Naturelle-CNRS-SU-EPHE-UA, 57 rue Cuvier, CP 39, 75231 Paris, Cedex 05, France; 5 Herbaria Basel, Department of Environmental Sciences, University of Basel, Basel, Switzerland; 6 Herbarium, Royal Botanic Gardens, Kew, Richmond, Surrey, TW9 3AE, UK; 7 Herbier MPU, DCSPH – CC 99010, Université de Montpellier, 163 rue A. Broussonnet, F-34090 Montpellier, France; 8 Naturalis Biodiversity Center, Darwinweg 2, 2333 CR, Leiden, Netherlands; 9 Laboratoire de Botanique systématique et d'Ecologie, Département des Sciences Biologiques, Ecole Normale Supérieure, Université de Yaoundé I, BP 047, Yaoundée, Cameroon

**Keywords:** conservation, evolution, new species, phylogeny, tropical rain forests

## Abstract

Monodoreae (Annonaceae) is a tribe composed of 11 genera and 90 species restricted to the tropical African rain forests. All the genera are taxonomically well circumscribed except the species rich genera *Uvariodendron* and *Uvariopsis* which lack a recent taxonomic revision. Here, we used a robust phylogenomic approach, including all the 90 currently accepted species, with several specimens per species, and based on more than 300 Annonaceae-specific nuclear genes, to infer the phylogenetic tree of the Monodoreae and test the limits between the genera and species. We recover all the genera as monophyletic, except the genus *Uvariopsis* for which the species *Uvariopsistripetala* falls outside this clade. We thus reinstate the monotypic genus *Dennettia* for its single species *Dennettiatripetala*. We also erect a new tribe, Ophrypetaleae **trib. nov.**, to accommodate the genera *Ophrypetalum* and *Sanrafaelia*, as we recover them excluded from the Monodoreae tribe with good support. Below the genus level, the genera *Isolona*, *Monodora*, *Uvariastrum*, *Uvariodendron* and *Uvariopsis* show weakly supported nodes and phylogenetic conflicts, suggesting that population level processes of evolution might occur in these clades. Our results also support, at the molecular level, the description of several new species of *Uvariodendron* and *Uvariopsis*, as well as several new synonymies. Finally, we present a taxonomic revision of the genera *Dennettia*, *Uvariodendron* and *Uvariopsis*, which contain one, 18 and 17 species respectively. We provide a key to the 11 genera of the Monodoraeae and describe four new species to science: *Uvariodendronkimbozaense* Dagallier & Couvreur, **sp. nov.**, *Uvariodendronmossambicense* Robson ex Dagallier & Couvreur, **sp. nov.**, *Uvariodendronpilosicarpum* Dagallier & Couvreur, **sp. nov.** and *Uvariopsisoligocarpa* Dagallier & Couvreur, **sp. nov.**, and provide provisional descriptions of three putatively new species. We also present lectotypifications and nomenclatural changes implying synonymies and new combinations (*Uvariodendroncitriodorum* (Le Thomas) Dagallier & Couvreur, **comb. et stat. nov**., Uvariodendronfuscumvar.magnificum (Verdc.) Dagallier & Couvreur, **comb. et stat. nov.**, Uvariopsiscongensisvar.angustifolia Dagallier & Couvreur, **var. nov.**, Uvariopsisguineensisvar.globiflora (Keay) Dagallier & Couvreur, **comb. et stat. nov.**, and Uvariopsissolheidiivar.letestui (Pellegr.) Dagallier & Couvreur, **comb. et stat. nov.**).

## Abstract (es)

La tribu des Monodoreae (Annonaceae) est composée de 11 genres et 90 espèces des forêts tropicales humides d’Afrique. Tout les genres sont taxonomiquement bien résolus, à part les genres *Uvariodendron* et *Uvariopsis* qui manquent d’une révision taxonomique récente. Ici, nous avons utilisé une approche phylogénomique robuste pour estimer l’arbre phylogénétique des Monodoreae, et tester les limites de genres et d’espèces. Pour cela, nous avons inclut les 90 espèces acceptées, et avons séquencé plus de 300 gènes. Tous les genres sont retrouvés monophylétiques, à part le genre *Uvariopsis* pour lequel l’espèce *Uvariopsistripetala* se retrouve exclue. Nous rétablissons donc le genre monotypique *Dennettia* et son unique espèce *Dennettiatripetala*. Nous érigeons une nouvelle tribu, les Ophrypetaleae **trib. nov.**, pour accueillir les genres *Ophrypetalum* et *Sanrafaelia*, car nous les retrouvons exclus de la tribu des Monodoreae avec un bon support. Au niveau infra-générique, les genres *Isolona*, *Monodora*, *Uvariastrum*, *Uvariodendron* et *Uvariopsis* montrent de faibles supports de noeuds et des conflits phylogénétiques, ce qui suggère que des processus d’évolution se déroulent au niveau des populations. Nos résultats soutiennent également, sur un plan moléculaire, la description de plusieurs nouvelles espèces d’*Uvariodendron* et d’*Uvariopsis*, de même que plusieurs synonymies. Enfin, nous présentons une révision taxonomique des genres *Dennettia*, *Uvariodendron* et *Uvariopsis*, qui contiennent respectivement un, 18 et 17 espèces. Nous fournissons une clé des 11 genres de Monodoreae, et décrivons quatre nouvelles espèces pour la science: *Uvariodendronkimbozaense* Dagallier & Couvreur, **sp. nov.**, *Uvariodendronmossambicense* Robson ex Dagallier & Couvreur, **sp. nov.**, *Uvariodendronpilosicarpum* Dagallier & Couvreur, **sp. nov.** et *Uvariopsisoligocarpa* Dagallier & Couvreur, **sp. nov.**, et fournissons une description provisoire de trois autres potentielles. Nous effectuons des lectotypifications et des changements nomenclaturaux tels que des synonymies et des nouvelles combinaisons (*Uvariodendroncitriodorum* (Le Thomas) Dagallier & Couvreur, **comb. et stat. nov**., Uvariodendronfuscumvar.magnificum (Verdc.) Dagallier & Couvreur, **comb. et stat. nov.**, Uvariopsiscongensisvar.angustifolia Dagallier & Couvreur, **var. nov.**, Uvariopsisguineensisvar.globiflora (Keay) Dagallier & Couvreur, **comb. stat. nov.**, et Uvariopsissolheidiivar.letestui (Pellegr.) Dagallier & Couvreur, **comb. stat. nov.**).

## ﻿Introduction

Annonaceae is a diverse pantropical family of trees, shrubs and lianas mostly restricted to tropical rain forests. Recent work based on molecular phylogenetics has classified Annonaceae into four subfamilies and several tribes (Chatrou et al. 2012; Guo et al. 2017a; Couvreur et al. 2019). Within the Annonoideae subfamily, tribe Monodoreae – previously referred to as the “African long-branch clade” (Couvreur et al. 2008a) — is composed of species distributed exclusively in tropical Africa and Madagascar. Before this study, the tribe was composed of 11 genera and ca. 90 species.

Monodoreae is a diverse clade of trees and small shrubs with one species (*Monodoracrispata*) reported to be a lianescent (Couvreur 2009). Some genera, such as *Uvariodendron*, conform to the typical Annonaceae floral model of three sepals with alternating two whorls of three free petals, numerous free carpels (apocarpy) and bisexual flowers (van Heusden 1992; Couvreur et al. 2008b). In contrast, several genera within the tribe deviate from this pattern. For example, the genus *Uvariopsis* has dimerous flowers (two sepals and four petals) and individuals are unisexual, while the genera *Asteranthe*, *Hexalobus*, *Isolona*, *Monodora*, *Sanrafaelia*, and some species of *Uvariopsis* have fused inner and outer petals forming a single whorl. The genera *Isolona* and *Monodora* have prezygotically fused carpels, i.e. syncarpous gynoecia, which is unique in Annonaceae (Deroin 1985; Couvreur et al. 2008b; Couvreur 2009). This important diversity renders a clear morphological circumscription of the Monodoreae tribe complicated. To date it has however been recognized by the combination of the mainly tree or shrub habit, indumentum of simple hairs, and especially sessile or shortly stipitate monocarps (Couvreur et al. 2008b; Chatrou et al. 2012). Most of the genera have been taxonomically revised (Couvreur 2009, 2014; Botermans et al. 2011; Gosline et al. 2019) except for the monotypic genera *Monocyclanthus*, *Ophrypetalum* and *Sanrafaelia*, and the genus *Asteranthe* with two species (Vollesen 1980). Recently, a new genus including two species, *Lukea*, was described from East Africa (Cheek et al. 2022). Morphologically, *Lukea* was suggested to be close to the Monodoreae genera *Mischogyne* and *Monocyclanthus* and thus potentially belongs to this tribe (Cheek et al. 2022).

In contrast to the recent taxonomic revisions cited above, recent revisionary work for the two most diverse genera of the tribe, *Uvariodendron* and *Uvariopsis*, are more than 80 years old (Fries 1930; Robyns and Ghesquière 1933a). Several new species were described since then (Verdcourt 1971a; Gereau and Kenfack 2000; Kenfack et al. 2003; Couvreur and Luke 2010; Dagallier et al. 2021; Couvreur et al. 2022; Gosline et al. 2022) warranting a new taxonomic revision of both genera. The taxonomic history of *Uvariopsis* is complex with several expansions of the concept of the genus. For example, Kenfack et al. (2003) combined the monotypic genus *Dennettia* Baker f. into *Uvariopsis*, expanding the concept of this genus to having trimerous and dimerous flowers. This was supported by a molecular phylogeny based on few plastid markers (Couvreur et al. 2008a; but only included three species of *Uvariopsis*), and was recently contested based on morphological grounds (Cheek et al. 2022).

Reconstruction of phylogenetic relationships in species-rich clades such as Annonaceae is challenging. Several phylogenetic analyses have focused on the whole family (Doyle et al. 2004; Richardson et al. 2004; Guo et al. 2017a; Xue et al. 2020) or on particular tribes or genera (Couvreur et al. 2008a, b; Couvreur 2009; Thomas et al. 2012; Chaowasku et al. 2014; Tang et al. 2015; Guo et al. 2017b; Ortiz-Rodriguez et al. 2018). These phylogenies were reconstructed using few molecular markers (generally less than 10 chloroplast markers), although more recent studies included more and more markers (Guo et al. 2018; Lopes et al. 2018). In the Monodoreae tribe, phylogenetic relationships between the genera have been inferred with relatively strong statistical support in several molecular phylogenetic studies based on a few chloroplast markers (Couvreur et al. 2008a; Guo et al. 2017a; Xue et al. 2020). These studies inferred three main clades, referred to here as: the “*Isolona* clade”, (containing the genera *Asteranthe*, *Hexalobus*, *Uvariastrum*, *Isolona*, and *Monodora*), the “*Uvariopsis* clade” (containing the genera *Uvariopsis*, *Monocyclanthus*, *Uvariodendron*, and *Mischogyne*), and the *Ophrypetalum* – *Sanrafaelia* clade (with just two species). This later clade was recovered as sister to the rest of the Monodoraeae tribe with moderate to strong support based on phylogenetic analyses of plastid markers. The development of phylogenomics in Annonaceae (Couvreur et al. 2019) not only brought new insights to the understanding of the evolution of the family and of the tropical rain forests (Brée et al. 2020; Helmstetter et al. 2020a, b), but also clarified the systematics of the family. For example, the African genus *Annickia* was inferred to be excluded from Piptostigmateae, and erected as a new monogeneric tribe Annickieae (Couvreur et al. 2019). Some Monodoreae genera were also included in this study and in contrast to previous analyses (Couvreur et al. 2008a, b; Guo et al. 2017a), they found a strong phylogenetic affinity of *Sanrafaelia* to another tribe of Annonaceae, namely Uvarieae (Couvreur et al. 2019). This suggested that, based on a poor taxon sampling, the *Ophrypetalum*–*Sanrafaelia* clade might not be part of Monodoreae.

Species level relationships within Monodoreae have only been inferred, based on few chloroplast markers, in *Isolona*, *Monodora* and more recently in *Mischogyne* (Couvreur 2009; Gosline et al. 2019) providing support to species and generic delimitation within the tribe. Moreover, the placement of *Lukea* within Monodoreae and the relationships in the species-rich *Uvariodendron* and *Uvariopsis* genera have never been assessed with a near-complete taxon sampling.

The aim of the present study is to generate a robust species-level phylogenomic tree of the Monodoreae and test the limits between the genera and species. Given that the species rich genera *Uvariodendron* and *Uvariopsis* have never been fully assessed phylogenetically and taxonomically, we give a particular focus to these two genera and present a taxonomic revision of these two genera. We discuss species delimitation based on both molecular and morphological evidence.

## ﻿Materials and methods

### ﻿Taxonomic revision

We examined the morphological characters of the Monodoreae species based on the protologues of the species and on the taxonomic revisions of those genera that were available (Diels 1936; Keay 1953; Verdcourt 1960, 1996; Vollesen 1980; Couvreur 2009, 2014; Botermans et al. 2011; Gosline et al. 2019; Cheek et al. 2022). In addition, we examined numerous specimens of *Uvariodendron* and *Uvariopsis*, as well as several specimens of *Monocyclanthus*. The specimens came from B, BM, DSM, EA, G, K, M, MO, MPU, P, WAG and YA herbaria (herbaria acronyms follow Thiers 2016). The high quality scans of some additional specimens or sheets from A, BR, BRLU, COI, E, FHO, GOET, HBG, L, LE, MA, MHU, S, US, WU and Z were also examined, as well as pictures taken in the field or personal fieldwork observations. For the type specimens, an exclamation mark (!) is appended stating either the specimen itself or a high quality scan was seen. Additional duplicates from herbaria not listed above might appear in the lists of specimens, but these were not seen and are cited for information. The format for the list of specimens follow recommendations from Bénichou et al. (2018) and includes a bullet point (•) at the beginning of each specimen to visually ease the reading.

When a holotype was composed of several sheets from a same gathering within a herbarium, we designated a single sheet as holotype (identified by its barcode) with the mention ‘sheet here designated’. The other sheets were listed as isotypes. This was done for all names except when the protologue explicitely specified that the holotype is composed of several sheets from a same gathering within a herbarium (e.g. Uvariodendronfuscumvar.magnificum),

Measurements, colors and other details given in the descriptions are based on living material, spirit and herbarium specimens, and data derived from field notes. Morphological descriptions followed terminologies from Hickey et al. (1999) and the Systematics Association Committee for Descriptive Biological Terminology (1962) for leaf and plane shapes, Payne (1978) for the indumentum, and Harris and Harris (2001) for the other terms. To characterise the seeds, we used the term ‘semicircular’ to describe the three dimensional shape of a semicircle, like a D-shaped ellipsoid (see e.g. Fig. 29H). We used custom scripts inspired by the *exsic* (Simon and Spooner 2013) and *monographR* (Reginato 2016) packages in R 4.1.3 (R Core Team 2022) to automatically generate initial species descriptions and specimens’ citations.

For this work, we applied a combination of the phylogenetic and morphological species concept. Thus, a species is recognized if it has support for monophyly and if it exhibits morphological characters that can differentiate it from the others. We also recognized varieties (or sunk previously described species into varieties) when a difference of morphology could be identified, but no monophyly was recovered by the phylogenetic analyses. In some cases, we recognized varieties even if they present an overlap in some of the morphological characters used to differentiate them. This is to recognize the existence of different morphological groups (or “archetypes”) in the morphological variation of the species. This reflects the uncertainty around the species, given the material available. The collection of additional material will certainly provide a better understanding of these morphologically complex groups.

To avoid ambiguities, when necessary, the names *Uvariodendron* and *Uvariopsis* were abbreviated to *Ud.* and *Up.*, respectively.

IUCN assessments were retrieved from https://www.iucnredlist.org/ (accessed 21 September 2022). For species not present in the Red List, we made a preliminary conservation status assessment. We calculated the extent of occurrence (EOO) and the area of occupancy (AOO) using the ConR package (Dauby et al. 2017) and followed IUCN Red List Categories and Criteria Version 3.1 (IUCN 2012) to assign a preliminary conservation status. For the distribution of the species, we specify the country(ies) in which they are distributed, as well as their chorological classification. The chorological part follows White (1979, 1983, 1993) but simplifies the chorological categories (“regional (sub)centre of endemism” and other) into Region and Domain.

### ﻿Phylogenomics

#### ﻿Taxon sampling

We sampled at least one specimen for every species of the Monodoreae tribe, several specimens from the Uvarieae tribe representing different genera, as well as several other Annonaceae outgroups (Chatrou et al. 2012; Suppl. material 3). Within Monodoreae, we sampled, when possible, more than one specimen per species following these cases: one specimen per subspecies or variety; one specimen per major geographic region for widespread species (between East Africa, West Africa, Central Africa); several specimens for morphologically variable species representing that variability (Suppl. material 3).

For all specimens, DNA was extracted from either silicagel dried leaf samples or leaf material taken from herbarium specimens. Details about specimens and vouchers are available in Suppl. material 3.

For the downstream analyses, every sample was assigned a unique identifier as follow: RUN_##_I##_T##_Genus_species-COL_NUM. “RUN_##_I##_T##” is the identifier used for the sequencing process and bioinformatics analyses. “Genus_species” is the most up to date taxonomic identification of the specimen (i.e. after applying the taxonomic changes treated in this paper). To ease the reading of updated names, some of the specimens have their old identification in parentheses like “Genus_species_(ex_old name)”. “COL_NUM” is the three first letters of the name of the collector and the number of collection of the specimen (e.g. “VAL_2540” stands for the specimen n°2540 collected by J.L.C.H van Valkenburg). The details about every sample can be found in the Suppl. material 3.

#### ﻿DNA extraction, library preparation and sequencing

DNA extraction and NGS library preparations for nuclear exon capture followed Couvreur et al. (2019). Libraries were sequenced using an Illumina HiSeq 2000 plateform using paired-end sequencing of length 150 bp by Novogene Co. Ltd.

#### ﻿Raw sequence bioinformatics

Raw read sequences were demultiplexed using the demultadapt script (https://github.com/Maillol/demultadapt). Adapters were removed from the reads using cutadapt 1.18 (Martin 2011) with the “-b” option and the “-O 7 -m 35 -q 20 -e 0.1” parameters. Low quality reads were then excluded (mean quality phred score below 30 and read less than 35 bp long) using a script modified from https://github.com/SouthGreenPlatform/arcad-hts/blob/master/scripts/arcad_hts_2_Filter_Fastq_On_Mean_Quality.pl. Reverse and forward reads were paired according to their names in the fastq files using a custom script (https://github.com/SouthGreenPlatform/arcad-hts/blob/master/scripts/arcad_hts_3_synchronized_paired_fastq.pl). A final step of 6 bp trimming was performed on the reverse reads using FASTX-Toolkit (https://github.com/agordon/fastx_toolkit).

#### ﻿Contig assembly and alignment

To process the clean reads, we used the HybPiper v1.3.1 pipeline (Johnson et al. 2016) following Couvreur et al. (2019). This pipeline mapped the reads on to the targeted nuclear exons using BWA v0.7.12 (Li and Durbin 2009). The mapped reads were assembled into contigs using SPAdes v3.11.1 (Bankevich et al. 2012). Overlapping contigs were then assembled into ‘supercontigs’ containing both targeted sequence (i.e. exon) and off-target sequence data (i.e. partial intron). The pipeline ends up with one file per gene (supercontig) containing the sequence for every individual. The sequences were then aligned for each gene using MAFFT v7.305 (Katoh and Standley 2013) using the automatic selection of the alignment algorithm (--auto). Alignments were trimmed with Gblocks 0.91b (Talavera and Castresana 2007), to remove poorly aligned regions.

#### ﻿Phylogenetic reconstruction

We used two different approaches for the phylogenetic reconstruction: the concatenation approach and the gene tree approach. For each approach, we first removed the supercontigs that were putatively identified as paralogs within the HybPiper pipeline, and then filtered our dataset to select only the supercontigs that are present in 75% of the individuals and for which the sequence contains at least 75% of the length of the target sequence (75/75 filter, following Couvreur et al. 2019).

#### ﻿Concatenation approach

For each supercontig, the alignments were filled with gaps and missing individuals so that every sequence in the alignment has the same length and every alignment contains all the individuals. The alignments were then concatenated into a single “supermatrix” using the *pxcat* function in the phyx program (Brown et al. 2017). A phylogenetic tree was inferred from the supermatrix using an optimized maximum likelihood (ML) tree search implemented in RAxML 8.2.9 with the GTRGAMMA model after a rapid bootstrap analysis (Stamatakis 2014). We specified *Anaxagoreacrassipetala* as an outgroup (“-o” option) and constrained the ML search on a backbone topology (“-g” option), in order to avoid long-branch attraction due to the scarce sampling of the outgroups. The backbone topology only includes the outgroup species and follows the results of Couvreur et al. (2019): (Anaxagorea_crassipetala-MAA_9408, (((((Uvariodendron_kirkii-DAG_09, Uvaria_grandiflora-COU_838), Annona_glabra-CHA_467), Duguetia_staudtii-COU_1014), Guatteria_jefensis-MAA_9553), Greenwayodendron_suaveolens-COU_746)).

#### ﻿Gene trees approach

We inferred a phylogenetic tree for every supercontig using RAxML 8.2.9 (Stamatakis 2014) under the GTRGAMMA model and 100 bootstrap replicates. For every tree obtained, we removed the branches with a bootstrap support less than 10% using the Newick Utilities program (https://github.com/tjunier/newick_utils), in order to improve the accuracy of the inferred species tree (Zhang et al. 2018). We then inferred the summary species tree from all the (unrooted) genes trees using ASTRAL-III 5.7.5 under the multi-species coalescent model (Zhang et al. 2018, 2018). The support at the branches was estimated both with the quartet support (QS) values (“-t 1” option) and with the local posterior probabilities (LPP) (default parameters). The species tree was then rooted on *Anaxagoreacrassipetala* (Anaxagorea_crassipetala-MAA_9408). The phylogenetic trees obtained were then plotted and annotated using the *ggtree*, *ggplot* and *treeio* packages (Wickham 2016; Yu et al. 2017; Wang et al. 2020).

## ﻿Results

### ﻿Specimens examined

In total, we examined ca. 1,500 herbarium sheets, representing 928 herbarium specimens, including 486 of *Uvariodendron*, 405 of *Uvariopsis*, 35 of *Dennettia*, seven of *Monocyclanthus* and two of *Lukea* (Suppl. material 4, https://doi.org/10.15468/zdvvkh). We compiled the morphological characters of the Monodoreae genera in the Table 1.

**Table 1.** Morphological comparison of the Monodoreae genera. In bold, the combination of morphological characters diagnostic to each specific genus. H:w, height:width ratio.

	Number of accepted species	Leaf length	Tertiary veination	Sepals	Petals	Receptacle H:w	Flower sexuality	Carpels
*Asteranthe*	2	40–165 (180) mm	reticulate	3, free to shortly connate at base	2 whorls of 3, **fused at base**, inner and outer petals subequal	ca. 1	bisexual	**free**
*Dennettia*	1	**< 150 mm long**	reticulate	**3, fused at base, forming a persistent ring with the 3 lobes generally clearly distinct, curved downward on the fruiting pedicel**	**1 whorl of 3**, free	**< 1**	bisexual	free
*Hexalobus*	5	36–360 mm long	reticulate	3, free to shortly connate at base	**1 whorl of 6, fused at base, folded in bud**	ca. 1	bisexual	**free**
*Isolona*	20	**50–280 mm long**	between reticulate and percurrent	**3, free, valvate**	**1 whorl of 6**, **fused** at base	**< 1**	bisexual	fused (true syncarpy)
*Lukea*	2	68–210 mm long	prominent reticulate	3, **fused, forming a bowl-shaped** receptacle, persistent on the fruiting pedicel	**1 whorl of 3**, free	> 1	bisexual	**free**
*Mischogyne*	5	**70–320 mm long**	prominent reticulate	**3, free, reduplicate-valvate in bud**	2 whorls of 3, free	**> 1**	bisexual	free, **connivent at base and separated and slightly curved outwards at apex**
*Monocyclanthus*	1	> 150 mm long	reticulate	**3, fused at base, forming a ring** with the **lobes generally unconspicious, persistent on the fruiting pedicel**	1 whorl of 6, free	> 1	bisexual	**free**
*Monodora*	14	**40–500 mm long**	reticulate or more rarely percurrent	**3, free, valavate**	**2 whorls of 3**, **fused** at base, outer petals longer than inner petals	**<1 to 1**	bisexual	fused (true syncarpy)
*Uvariastrum*	5	60–220 mm long	reticulate	3, free, **reduplicate-valvate** in bud, enclosing the petals in bud	2 whorls of 3, free	ca. 1	bisexual	**free, connivent all along their length**
*Uvariodendron*	18	**65–750 mm long**	reticulate	**3, connivent to fused at base**	**2 whorls of 3, free**, **inner and outer subequal** in length, **inner petals connivent** at apex	**ca. 1**	bisexual	free
*Uvariopsis*	17	70–615 mm long	reticulate	**2**, free to fused at base	**1 whorl of 4** (1 whorl of 3 in *Uvariopsiscongolana*), free to fused	1–1.2	**unisexual** (except for *Uvariopsisbisexualis*)	free

### ﻿Phylogenomics

A total of 464 exon regions were recovered out of the 469 contained by the Annonaceae baiting kit (Couvreur et al. 2019). Of these, 39 exon regions were identified as potential paralogs using HybPiper. After discarding the potential paralogs and applying the 75/75 filter (see Methods), the dataset comprised 334 supercontigs. This subset was used for the downstream phylogenetic analyses with both the concatenation (after concatenation to a supermatrix) and gene trees approaches. After alignment, the supercontigs ranged in length between 131 and 9422 distinct sites (mean: 1,234; median: 844). After concatenation of the 334 supercontigs, the supermatrix totaled 449,445 distinct sites.

### ﻿Phylogenetic relationships

**Figure 1.**
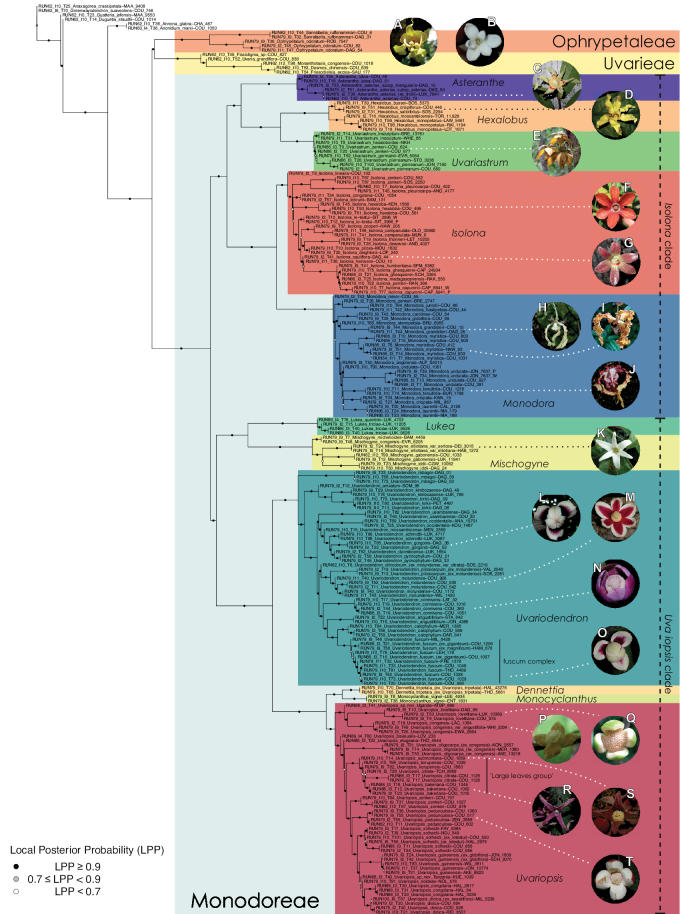
Phylogenetic trees of the Monodoreae inferred with ASTRAL, based on 334 nuclear genes trees. The branch support is given as local posterior probability (LPP) in three shades of greys. For the details on the specimens see Suppl. material 3. Insets: flowers of **A***Ophrypetalumodoratum* (Couvreur 82) **B***Sanrafaeliaruffonammari* (Dagallier 31) **C***Asterantheasterias* (Dagallier 10) **D***Hexalobuscrispiflorus* (no specimen associated) **E***Uvariastrumzenkeri* (no specimen associated) **F***Isolonahexaloba* (no specimen associated) **G***I.cauliflora* (Dagallier 44) **H***Monodoragrandidieri* (Dagallier 26) **I***M.myristica* (living specimen at NY conservatory) **J***M.tenuifolia* (Couvreur 1019) **K***Mischogyneelliotana* (no specimen associated) **L***Uvariodendrongorgonis* (Dagallier 38) **M***Ud.molundense* (Bidault 2222) **N***Ud.connivens* (Couvreur 1016) **O**Ud.var.fuscum (Couvreur 990) **P***Uvariopsiscongensis* (Lachenaud 1384) **Q***Up.lovettiana* (Couvreur 97b) **R***Up.bakeriana* (Couvreur 1000) **S***Up.submontana* (Couvreur 627) **T***Up.pedunculosa* (Couvreur 878). Photos CC BY-NC 4.0 **A, D–F, J, N, O, Q–T** Thomas Couvreur **B, C, G–I, L** Léo-Paul Dagallier **K** Carel Jongkind **M** Ehoarn Bidault **P** Olivier Lachenaud.

At the genus level and above, we recovered the same topology with both the gene tree (ASTRAL) and the concatenation (RAxML) approaches (Fig. 1, Suppl. materials 1, 2) with high levels of statistical support. The local posterior probabilities (LPP) were all above 0.9, the quartet supports (QS) were generally high, and the bootstrap (BS) values were all 100%. The *Ophrypetalum – Sanrafaelia* clade is retrieved as sister to the Uvarieae with maximum BS and LPP support but with relatively low QS (44%) (Fig. 1, Suppl. materials 1, 2). All the genera were retrieved as monophyletic, except *Uvariopsis* for which the species *Uvariopsistripetala* clustered as sister to *Monocyclanthus* (Fig. 1, Suppl. materials 1, 2). The two species of the newly described genus *Lukea* clustered together, as sister to *Mischogyne* with strong support. The branch leading to the crown node of *Mischogyne* was relatively long (Fig. 1, Suppl. materials 1, 2).

Species monophyly within the genera *Asteranthe*, *Hexalobus*, *Lukea*, *Mischogyne*, and *Monocyclanthus* received strong support, and the relationships between species were generally strong (BS > 90%, LPP > 0.9) and similar between both phylogenetic approaches (Fig. 1, Suppl. materials 1, 2). In *Hexalobus*, the three specimens of *Hexalobusmonopetalus* sampled from different countries (from Senegal, Mozambique and Angola) clustered together (Fig. 1, Suppl. materials 1–3) despite the species being widespread.

In *Isolona*, *Monodora*, *Uvariastrum*, *Uvariodendron* and *Uvariopsis*, there are incongruences in topologies between the RAxML and the ASTRAL trees, and the node support is generally strong (BS > 90%, LPP > 0.9), but some nodes are weakly (BS < 70%, LPP < 0.7) to moderately supported (70% < BS < 90%, 0.7 < LPP < 0.9) (Fig. 1, Suppl. materials 1, 2).

In *Uvariastrum*, *U.pierreanum* and *U.insculptum* are strongly supported as monophyletic, but *U.zenkeri* is recovered as paraphyletic. The species *U.germainii* is retrieved as sister to *U.pierreanum* with moderate to strong support in both approaches. *U.insculptum* is retrieved as sister to all the other species in the genus (Fig. 1, Suppl. materials 1, 2).

In *Uvariodendron* and *Uvariopsis*, most species were recovered as monophyletic (Fig. 1, Suppl. materials 1, 2), but in some cases we appear to have a species complex, that is either a cluster of closely related individuals from more than one species, or separate clusters of individuals from a single species (Sigovini et al. 2016). In *Uvariodendron*, the specimens identified as *Ud.giganteum* (Couvreur 1057 and Couvreur 1206) and *Ud.magnificum* (Hamilton 676) are nested in a clade with the specimens identified as *Ud.fuscum*, and the branches subtending *Ud.fuscum* specimens are generally weakly supported in both RAxML and ASTRAL trees (Fig. 1, Suppl. materials 1, 2). The specimens previously identified as *Ud.molundense* clustered together, but formed three genetically distinct and strongly supported groups: one including the single specimen Sosef 2219, previously identified as Ud.molundensevar.citrata, sister to the other two groups, another one composed of specimens Sosef 2261 and Valkenburg 2540, and a final group composed of the remaining sampled specimens. The three groups are separated by relatively long branches, especially the group formed by the specimens Sosef 2261 and Valkenburg 2540 (Fig. 1, Suppl. materials 1, 2).

In *Uvariopsis*, the crown node of the clade formed by *Up.bakeriana*, *Up.citrata*, *Up.korupensis*, *Up.submontana* (« large leaves » group on Fig. 1) is strongly supported, but forms a species complex: while *Up.bakeriana* is retrieved as monophyletic with strong support (Fig. 1, Suppl. materials 1, 2), *Up.korupensis* is retrieved as paraphyletic in both phylogenetic approaches (Fig. 1, Suppl. materials 1, 2), while *Up.citrata* is retrieved as monophyletic with moderate support in the gene tree approach only (QS < 50%, LPP < 0.7).

## ﻿Discussion

### ﻿Phylogenomics of the Monodoreae tribe

We provide here (Fig. 1, Suppl. materials 1, 2) the first complete species level phylogenomic reconstruction of the Monodoreae tribe based on 334 nuclear loci using a targeted sequencing approach (Couvreur et al. 2019). Indeed, we sequenced all of the 90 currently accepted species, and included the specimens of three potentially new species. Such a level of species sampling with hundreds of nuclear markers has never being achieved for a diverse tribe of Annonaceae to date. Couvreur et al. (2019) published a species level phylogenomic study using the Annonaceae baiting kit of tribe Piptostigmateae, but this study included 74% of know species in the tribe (Brée et al. 2020). In addition, we were also able to sample several specimens within species to test species limits and concepts. Our high level of inter- and intra-specific sampling for Monodoreae was possible thanks to the successful sequencing of DNA extracted from herbarium specimens. Indeed, 49% (101/207) of specimens sequenced in this study were sampled from herbarium specimens, versus 51% (106/207) available from different silicagel DNA banks. Four specimens included here were more than 110 years old dating back to 1898, 1900, 1910 and 1911. Our results underline once again the central importance of herbarium specimens for phylogenetic inference (Bebber et al. 2010; Bakker 2019; Brewer et al. 2019) of tropical plants, in particular to achieve high levels of taxon sampling needed to test species limits but also infer more accurate macroevolutionary patterns.

Generic level relationships within Monodoreae generally agree with molecular phylogenies based on few plastid markers (Couvreur et al. 2008a; Chatrou et al. 2012; Guo et al. 2017a). There are however, two major differences when compared to plastid data: the position of the two east African genera *Ophrypetalum* and *Sanrafaelia* not being sister to the rest of Monodoreae, and the position of the species *Uvariopsistripetala*. These differences and taxonomic implications are discussed in detail below.

Even though sequencing of hundreds of markers combined with a dense taxon sampling appear as a silver bullet to understand species limits, it doesn’t always provide clear cut answers. Indeed, phylogenetic conflicts are common in plant systematics: incongruences can occur between concatenation and gene tree approaches (Shen et al. 2021), or reconstructions based on nuclear loci versus chloroplast loci (Morales‐Briones et al. 2018). Such incongruences are often attributed to reticulate processes of evolution occurring at the population level, like horizontal gene transfer, hybridizations or incomplete lineage sorting (ILS) (Maddison 1997; Naciri and Linder 2015; Morales‐Briones et al. 2018; Schrempf and Szöllősi 2020). Phylogenetic conflict, associated with little divergence between species, is also known to coincide with rapidly evolving taxa in case of adaptive radiations (Meyer et al. 2017) and morphological innovations (Parins-Fukuchi et al. 2021). Widespread species can also be retrieved as non-monophyletic, with diverging range-restricted species embedded within them. This is due to the retention of ancestral polymorphisms in widespread species where both the population size and the generation time are too high to achieve complete lineage sorting from the divergence time until the present day (Pennington and Lavin 2016). This has been documented in Amazonia where tree species restricted to seasonally drought-affected forests are retrieved embedded in rain forests tree species (Pennington and Lavin 2016). These processes at the population level might be the cause of the observed phylogenetic conflicts in *Isolona*, *Monodora*, *Uvariastrum*, *Uvariodendron*, and *Uvariopsis*, and likely explain the species complex in the later two genera. Nevertheless, our study provides a solid phylogenetic framework to test different relationships within this tribe, confirming some and disagreeing with previous results. Below we discuss our results from a taxonomy point of view.

### ﻿Re-circumscription of the tribe

The two monotypic sister genera from East Africa, *Ophrypetalum* Diels (Kenya and Tanzania) and *Sanrafaelia* Verdc. (Tanzania) (Diels 1936; Verdcourt 1960, 1996), called the *Ophrypetalum*–*Sanrafaelia* clade here, were informally linked to some genera (i.e. the *Hexalobus* group, *Asteranthe*, *Hexalobus*, *Isolona*, *Monodora*) now placed in Monodoreae based on floral morphology (van Heusden 1992). Phylogenetic studies at the genus level and based on few chloroplast markers recovered these two genera as sister to all the other Monodoreae genera with moderate to strong support (Couvreur et al. 2008b; Chatrou et al. 2012; Guo et al. 2017a; Xue et al. 2020). Here, with more than 300 nuclear loci and a much denser species level sampling, we retrieved both genera as sister to the Uvarieae tribe, with strong support (Fig. 1, Suppl. materials 1, 2). Our analyses thus confirm the result of Couvreur et al. (2019) using the same baiting kit, but only sampling *Sanrafaelia* and fewer species within Monodoreae. The discrepancy between the relationships inferred in the previous studies and here can be attributed to the higher number of loci and species sequenced, but also to the use of nuclear vs. chloroplast loci.

**Figure 2.**
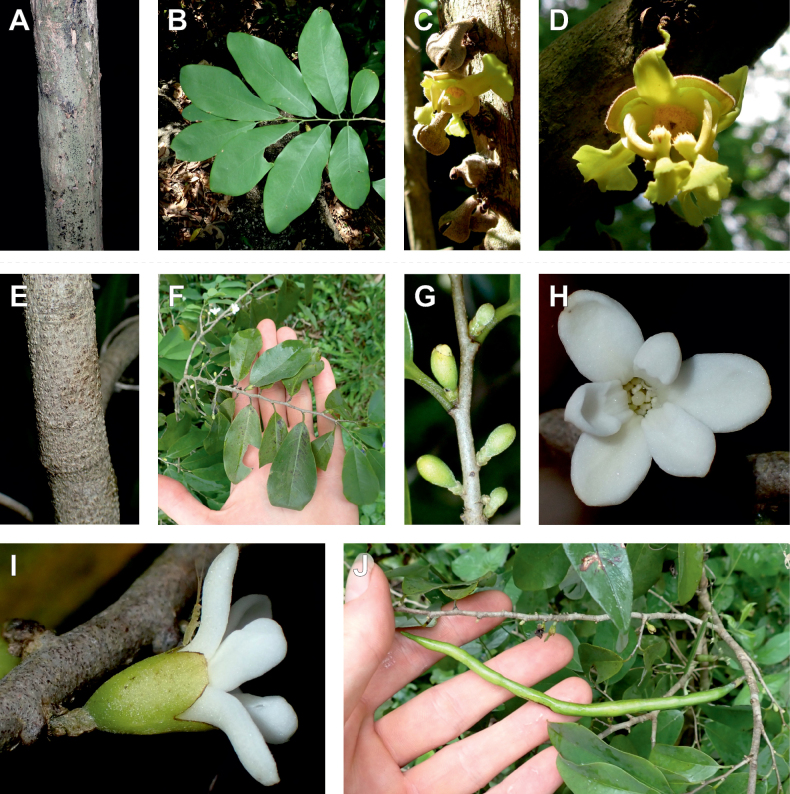
Ophrypetaleae tribe. *Ophrypetalumodoratum* Diels **A** trunk **B** young branch with leaves, upper side **C** flower and flower buds **D** flower, from below. *Sanrafaeliaruffonammari* Verdc **E** trunk **F** young branch with leaves and flower buds **G** flower buds **H** flower, top view **I** flower, side view **J** fruit. **A, B** Dagallier 54 **C** Couvreur 56 **D** Couvreur 82 **E–J** Dagallier 31. Photos **A,,B, E–J** Léo-Paul Dagallier **C, ,D** Thomas Couvreur.

The *Ophrypetalum*–*Sanrafaelia* clade share several morphological characters also found in other Monodoreae genera (Fig. 2), in addition to occurring in tropical Africa, such as being trees with simple indumentum, monocarps (individual fruiting units) that are sessile or shortly stipitate (stipes shorter than 10 mm) or pollen units forming tetrads (except for *Isolona* (monads)) (Couvreur et al. 2008b). The morphological link to tribe Uvarieae based on morphological grounds is harder to make. Uvarieae is mainly composed of genera having a lianescent habit, except for *Cleistochlamys* (East Africa) and *Dasymaschalon* (South East Asia) which are trees or shrubs (Verdcourt 1971a; Wang et al. 2009, 2012). In addition, most species have an indumentum of stellate hairs (more rarely simple in some *Uvaria* species) and fruits with generally stipitate monocarps and pollen in monads (Couvreur et al. 2008b; Chatrou et al. 2012; Doyle and Le Thomas 2012). Likely most of these characters are novelties for the Uvarieae, while both the Monodoreae and the *Ophrypetalum*–*Sanrafaelia* clade show the plesiomorph condition. This precludes *Ophrypetalum* and *Sanrafaelia* as being part of Uvarieae based on morphology alone. This distinction from the tribe Uvarieae is also supported genetically by the long branches sustaining the *Ophrypetalum*–*Sanrafaelia* clade from the crown node of Uvarieae (Fig. 1, Suppl. materials 1, 2). We thus propose the erection of the new tribe Ophrypetaleae to accommodate these two genera. The description of the new tribe is provided in the Taxonomic treatment section below. Interestingly, both genera are quite different in terms of flower morphology (Verdcourt 1971a, 1996). *Ophrypetalum* has two whorls of three free petals, with the inner ones clawed and presenting a small brush-like appendage on the inner side and has numerous carpels (10–15) (Fig. 2A–D). In contrast, *Sanrafaelia* has six petals united into a single whorl and has a single carpel (Fig. 2E–J). These differences between sister genera underline once again the great morphological diversity of African genera probably linked to differences in pollination vectors (Gottsberger et al. 2011).

After our study presented here, the tribe Monodoreae is composed of 11 genera and 90 species, and the main morphological characters are provide in Table 1.

### ﻿*Asteranthe*

*Asteranthe* is an East African genus characterised by large fleshy flowers with basally fused petals and numerous free carpels (Verdcourt 1971a). *Asteranthe* was strongly supported as monophyletic (Fig. 1, Suppl. materials 1, 2), and recovered as sister to the mainly central African genera *Hexalobus* and *Uvariastrum* confirming previous analyses (Couvreur et al. 2008a). The name *Asteranthetrollii* Diels (Diels 1936) was synonymized by Verdcourt with *Asterantheasterias* (S. Moore) Engl. & Diels (Verdcourt 1971a). Verdcourt argued that the type species of *A.trollii* was very similar to *A.asterias* (Verdcourt 1971a, b). Here, we sequenced the specimen Luke 7641, originally identified as *A.trollii*, which clustered with the other sampled specimens of *A.asterias* (Fig. 1, Suppl. materials 1, 2). Thus, even though we did not sequence the type specimen, our results support the synonymy proposed by Verdcourt (1971a, b).

In addition, Verdcourt (1971a) described the subspecies Asterantheasteriassubsp.triangularis based on the petals being narrowly triangular (vs. linear-oblong to linear in Asterantheasteriassubsp.asterias). In our analysis, both subspecies are genetically separated but sister groups (Fig. 1, Suppl. materials 1, 2), supporting Verdcourt’s proposal. However, sampling more specimens of A.asteriassubsp.triangularis would be necessary to decide whether the subspecies should be erected to the species level.

### ﻿*Hexalobus*

The genus *Hexalobus* contains five accepted species (Botermans et al. 2011). This genus is characterized by basally fused petals which are longitudinally folded (wrinkled) in bud, a character unique within the family (van Heusden 1992; Botermans et al. 2011). The species *H.monopetalus* has a very widespread “ring” type distribution, from Senegal to the north of South Africa (and parts of Angola). It occurs in drier woodland savannah or gallery forests (Botermans et al. 2011). This is unique among the Monodoreae species, which generally have ecological affinities with wetter areas and have a more restricted geographical distribution. Our geographically widespread sampling of *H.monopetalus* (three specimens from Angola, Mozambique and Senegal) was recovered as monophyletic with strong support (Fig. 1, Suppl. materials 1, 2), confirming that despite its widespread distribution, it is a single species. In addition, *H.monopetalus* is inferred as sister to the other dry adapted species of the genus *H.mosambicensis* (Botermans et al. 2011).

The widespread central African species *H.crispiflorus* is recovered as sister to the more narrowly distributed *H.salicifolius* (Cameroon and Gabon) with maximum support in both analyses (Fig. 1, Suppl. materials 1, 2). This confirms they are closely related as suggested by Le Thomas (1969). They do however differ by several morphological characters such as smaller leaves and smoother monocarps in *H.salicifolius*. Monophyly, however, needs to be confirmed by a denser sampling within each species.

### ﻿*Uvariastrum*

*Uvariastrum* is a genus containing five accepted species characterized by reduplicate-valvate (*i.e.* curved outwards) sepals margins (Couvreur 2014). Morphologically, the five species are clearly differentiated based on several characters such as the indumentum and leaf morphology. Our results, however, underline some level of phylogenetic conflict (Fig. 1, Suppl. materials 1, 2). The geographical distributions of all the species overlap, at least partially, in central Africa, except for *U.hexaloboides* that is distributed in Zambia and in Katanga region (south Democratic Republic of the Congo) (Couvreur 2014). The lack of support for the nodes subtending *U.zenkeri* and *U.germainii* could be caused by old or occasional hybridization between the species but the inter-fertility has never been accounted for for any of the *Uvariastrum* species. Another explanation is that they diverged too recently to exhibit much differentiation at the molecular level (Erkens et al. 2012).

### ﻿The case of the newly described genus *Lukea*

Recently, the genus *Lukea* Cheek & Gosline was described, endemic to East Africa with two species: *Lukeaquentinii* Cheek & Gosline and *Lukeatriciae* Cheek & Gosline (Cheek et al. 2022). Morphologically, *Lukea* resembles *Mischogyne* by the finely reticulate tertiary venation of the leaves and the absence of connective shield on the stamens (Gosline et al. 2019). *Lukea* presents a bowl-shape calyx persisting in fruit, a character unique within the Monodoreae tribe, although resembling the fused sepals found in *Monocyclanthus* (Cheek et al. 2022) and in *Uvariodendronschmidtii* W.R.Q. Luke, Dagallier & Couvreur (Dagallier et al. 2021). Our results recovered *Lukeaquentinii* as sister to *Lukeatriciae*, and the whole genus *Lukea* as sister to *Mischogyne*, with great genetic divergence (Fig. 1, Suppl. materials 1, 2). This supports the genus level status of *Lukea*, and its inclusion in Monodoreae as suggested previously (Cheek et al. 2022).

### ﻿*Mischogyne*

*Mischogyne* is a genus of five accepted species characterized by the combination of different morphological features such as prominent reticulate tertiary veins and carpels touching each other at their base but separated at their apex (Gosline et al. 2019). The clade formed by *M.congensis* and *M.micheloides* is consistent in both our analyses (Fig. 1, Suppl. materials 1, 2) and with previous phylogenetic work on the genus based on a few plastid locus (Gosline et al. 2019). The position of the East African species *M.idii* remains however uncertain, either retrieved as sister to the clade *M.congensis*–*M.micheloides* (Gosline et al. 2019), sister to *M.gabonensis* (Fig. 1, Suppl. materials 1) or to *M.elliotana* (Suppl. materials 2).

### ﻿*Uvariopsis*

*Uvariopsis* Engl. is one of the most diverse genera of the Monodoreae tribe with 17 currently accepted species and until recently, several species were unknown to science (Couvreur and Luke 2010; Couvreur and Niangadouma 2016). Morphologically, *Uvariopsis* is characterised by monoecious individuals (separate male and female flowers on a single individual) and by exhibiting a type of flower uncommon in Annonaceae, composed of two sepals and four petals (Robyns and Ghesquière 1933a). *Uvariopsiscongolana* (De Wild.) Fries however has two sepals and three petals (De Wildeman 1909). *Uvariopsiscongolana* was previously described under a different genus name (*Thonnera* De Wild.), but was later combined into *Uvariopsis* (Fries 1953). Both our molecular phylogenetic analyses retrieved *Up.congolana* as sister species of *Up.dioica* with strong support (Fig. 1, Suppl. materials 1, 2), confirming its placement within *Uvariopsis*. In 1986, Verdcourt described the species *Up.bisexualis* from Tanzania, having the typical *Uvariopsis* flower with two sepals and four petals (and other typical pollen microscopic characters, see Verdcourt (1986)), but with bisexual flowers, thus extending the concept of the genus as also having bisexual flowers (Verdcourt 1986).

#### ﻿*Dennettia*, *Monocyclanthus* and flower variations in *Uvariopsis*

In 1913, Baker described the monotypic genus *Dennettia* Baker f., with a single species *Dennettiatripetala* Baker f. having bisexual flowers with “2 rarely 3 sepals” and three petals in a single whorl (Baker 1913). In 2003, Kenfack et al. combined the genus *Dennettia* into the genus *Uvariopsis* under the name *Uvariopsistripetala* (Baker f.) G.E. Schatz, based on four main arguments (Kenfack et al. 2003). First, the morphological circumscription of *Uvariopsis* had been extended to also include bisexual flowers by Verdcourt (1986, *Up.bisexualis*); second, Kenfack et al. (2003), observed specimens of *D.tripetala* exhibiting flowers with 2 sepals and 4 petals, thus reminding of the typical *Uvariopsis* flower; third, Kenfack et al. (2003) considered that because *Up.congolana* has 3 petals, the number of perianth segments is unreliable as a generic distinction for *Uvariopsis*; and fourth, the pollen microscopic features of *D.tripetala* are very close to those of *Uvariopsis* (Kenfack et al. 2003; Doyle et al. 2004). This was initially confirmed based on molecular phylogenetic analyses using few plastid regions, recovering *Uvariopsistripetala* as nested within *Uvariopsis* but based on just three species sampled within *Uvariopsis* (Couvreur et al. 2008a; Chatrou et al. 2012).

After careful examination of 16 flowering specimens identified as *Uvariopsistripetala* (including the type Dennett 44), we found that this species invariably exhibits 3 sepals and 3 petals. The sepals are fused at the base up to more than half of their length, forming a ring curved downward and persisting on the fruiting pedicel. In some rare cases we observed that the lobes of the calyx (i.e. the free parts at the apices of the basally fused sepals) are sometimes almost inconspicuous on the fruiting pedicel, allowing for the confusion with a two sepal-like calyx. To date, and based on the specimens we observed, we haven’t seen any flowers of *Up.tripetala* with four petals, contrary to the affirmation of Kenfack et al. (2003). If such flower arrangements exist, they must be extremely rare. It is also possible that the four petal flowers observed by Kenfack et al. (2003) were actually flower buds of the new species, *Uvariopsisoligocarpa* Dagallier & Couvreur (see the Taxonomic Treatment section), as their geographic ranges overlap and leaves are quite similar (but *Up.oligocarpa* is unisexual). In contrast to the previous phylogenetic analyses, we did not recover Dennettia (Uvariopsis) tripetala as nested within *Uvariopsis*, but as sister to the monotypic West African genus *Monocyclanthusvignei* Keay, with strong support in both phylogenetic approaches (Fig. 1, Suppl. materials 1, 2). Dennettia (Uvariopsis) tripetala differs from *Monocyclanthusvignei* (Keay 1953) by having three petals in one whorl (vs. six petals in one whorl), the lobes of the fused sepals generally distinct (vs. generally inconspicuous), and a receptacle height:width ratio less than 1, i.e. wider than tall (vs. a receptacle height:width ratio greater than 1, i.e. taller than wide) (see Table 1). The single whorl of six petals of *Monocyclanthus* supposedly results from the compression of separate ancestral whorls (Saunders 2010). For *Dennettia*, the single whorl of three petals might result from a similar compression of separate ancestral whorls followed by a loss of petals, or from the loss of a whorl of petals.

All these morphological and phylogenetic elements suggest that Dennettia (Uvariopsis) tripetala is part of neither the genus *Uvariopsis* nor the genus *Monocyclanthus*. We thus reinstate the monotypic genus *Dennettia*, with its single species *Dennettiatripetala* Baker f. (see Taxonomic treatment). This was also suggested previously by Cheek et al. (2022) based on morphology.

Given the above, the morphological circumscription of *Uvariopsis* must be reevaluated. *Uvariopsis* was generally circumscribed as having 2 sepals and 4 petals (Kenfack et al. 2003). However, *Uvariopsiscongolana* differs from all other *Uvariopsis* species by having flowers with 3 petals instead of 4. *Uvariopsiscongolana* was successfully sequenced and nested within *Uvariopsis* with strong support (sister to *Up.dioica*, Fig. 1, Suppl. materials 1, 2). Thus, the number of petals (4 versus 3) is not a good diagnostic character for *Uvariopsis*. However, *Up.congolana* has 2 sepals, similarly to all the other *Uvariopsis* species, making this character a good synapomorphy for the genus. The synoptic characters of the genus *Uvariopsis* Engl. are given in the Taxonomic Treatment section below.

#### ﻿The “large leaves” group

Based on our dense taxon sampling, we identified a group of closely related species for which the phylogenetic limits remain uncertain. Indeed, the four species *Up.bakeriana*, *Up.citrata*, *Up.korupensis* and *Up.submontana* clustered as monophyletic with strong support (Fig. 1, Suppl. materials 1, 2), but support for the monophyly of each individual species and the relationships between them remains ambiguous (Fig. 1, Suppl. material 2) with some gene tree conflict (Suppl. material 1). They share some morphological characters in particular the large size and shape of their leaves, hence referred to as the “large leaves” group here. They are all trees with large obovate leaves (up to 30–60 cm long) having rounded to cordate bases, and display flowers borne along the trunk with varying degrees on density, from densely packed in *Up.submontana* to sparsely distributed in *Up.citrata* (Kenfack et al. 2003; Couvreur and Niangadouma 2016). In addition, flowering pedicels are shorter than 7 cm and petals generally 3 to 10 times longer than wide. Despite these similarities, they differ by several marked characters. *Uvariopsisbakeriana* is characterized by its unique petals that are narrowly ovate to linear and bright pinkish to deep red in color (Hutchinson and Dalziel 1927b). *Uvariopsiscitrata* is characterized by its leaves emitting a strong lemon scent when crushed and sessile flowers (Couvreur and Niangadouma 2016). *Uvariopsiskorupensis* and *Up.submontana* have similar flowers with pedicels generally 20–70 mm long, ovate to narrowly ovate shortly fused at base, but are differentiated by their ecology (*Up.korupensis* occurs below 200 m, *Up.submontana* above 900 m), the degree of flowers density on the trunk (less densely clustered in *Up.korupensis*) and the sepals being smaller in *Up.korupensis* (1–5(7.5) mm long) than in *Up.submontana* (5–11 mm long) (Gereau and Kenfack 2000; Kenfack et al. 2003).

Based solely on the phylogeny, this group could be considered as a single species. However, given the morphological differences of each species detailed above we choose to keep the taxonomy of this group as it is. Phylogenetic conflict is known to be coincident with morphological innovations (Parins-Fukuchi et al. 2021), as observed in recent and fast diverging groups (Meyer et al. 2017). Here we retrieve such a pattern, with substantial morphological differentiation between *Up.citrata*, *Up.bakeriana* and *Up.korupensis*, but a lack of support at subtending nodes. *Uvariopsiskorupensis* is retrieved as paraphyletic and the two specimens sampled here (Couvreur 1039 and Louis 1863) come from different localities. They might belong to different isolated relictual populations failing to coalesce due to the persistence of ancestral polymorphisms (Pennington and Lavin 2016). The species *Up.bakeriana* and *Up.citrata* likely emerged from two populations that diverged from the ancestral *Up.korupensis* population. This remains to be clarified with a denser sampling (in particular of *Up.submontana*) or with a phylogeographic and adapted population genomics approaches (Hardy et al. 2013; Migliore et al. 2019; Helmstetter et al. 2020a).

#### ﻿New and under collected species

In addition to the results discussed above, our sampling and analysis confirmed the presence of new and/or under-collected species, which we detail here. Thanks to our dense sampling in the genus *Uvariopsis*, we were also able to test species limits, and concluded that several names are actually synonyms, which we detail under each concerned species in the Taxonomic treatment section below.

Morphologically, *Uvariopsissessiliflora* stands out from the other *Uvariopsis* species by having sessile flowers and leaves less than 20 cm long (*Up.citrata* also has sessile flowers but leaves more than 30 cm long). The only known specimen representing this species is its type specimen (Mildbraed 5239), collected in 1911 in Lomié, a relatively well collected locality of Cameroon (Sonké and Couvreur 2014). This specimen is relatively poor in information as it has only has a small branch, one flower and one monocarp. Here we were able to sequence this specimen and it turns out that it is phylogenetically embedded within *Up.dioica* (Fig. 1, Suppl. materials 1, 2), indicating that *Up.sessiliflora* and *Up.dioica* are the same phylogenetic species. It seems that the specimen Mildbraed 5239 represents a rare extreme in the morphological variation of *Ud.dioica*, with severe reduction of the flower pedicel. This also explains why no similar specimen was collected after all this time in a such relatively well explored locality. We thus make it synonym under the name *Up.dioica*.

The recently described species *Uvariopsisetugeana* Dagallier & Couvreur (Couvreur et al. 2022), is a rare tree species only known from two localities in Cameroon. Our results confirm it is phylogenetically different from other *Uvariopsis* species (Fig. 1, Suppl. materials 1, 2).

*Uvariopsisnoldeae* Exell & Mendonça is a species endemic to Angola (Exell and Mendonça 1951; Paiva 1966) and only known from the type specimen (Nolde 576). It is morphologically similar to *Uvariopsissolheidii* (De Wild.) Robyns & Ghesq., except for the young branches that are sparsely pubescent to glabrous whereas young branches of *Up.solheidii* are tomentose to shortly tomentose suggesting the names might be synonymous. Our phylogenetic analyses, however, revealed with strong support that *Up.noldeae* is genetically different from *Up.solheidii*, being sister to *Up.dioica* and *Up.congolana*, with *Up.solheidii* forming a strongly supported monophyletic group (Fig. 1, Suppl. materials 1, 2).

*Uvariopsiscongensis* differs from the other *Uvariopsis* species by its relatively small to medium sized leaves (less than 180 mm long), globose flower buds (vs. conical), and short flowering pedicels (less than 12 mm long). It is a widespread species distributed from Central Africa (Cameroon, Gabon) to East Africa (Uganda, Kenya) (Robyns and Ghesquière 1933a; Verdcourt 1971a). Interestingly, some of the specimens we identified as *Up.congensis* were from West Africa, (Sierra Leone to Ghana), thus outside of its suggested range. A thorough examination of these West African specimens revealed they differed by the morphology of their fruits. Moreover, our molecular phylogenetic analyses retrieved these West African specimens (referred as *Uvariopsisoligocarpa* on the Fig. 1 and Suppl. materials 1, 2) as monophyletic with strong support, and clearly separated from the Central and East African specimens (Fig. 1, Suppl. materials 1, 2). Sterile specimens of *Up.oligocarpa* are similar to *Up.congensis* and *Dennettiatripetala*, but are easily differentiated when female flowers or fruits are available. We thus describe these specimens under the new species *Uvariopsisoligocarpa* Dagallier & Couvreur.

Finally, the specimens of *Uvariopsiscongensis* from East Africa (Verdcourt 1971a) are also morphologically different from the specimens distributed in Central Africa. The East African specimens represent trees from 7 to 15 meters tall (vs. shrubs to trees from 2 to 7 meters tall for the Central African specimens) and they have narrowly elliptic laminas with a length:width ratio between 3 and 4 (vs. elliptic to obovate with a length:width ratio comprised between 2.1 and 3.1). Interestingly, the morphological differences between the two morphotypes are not supported by our phylogenetic analyses (Fig. 1, Suppl. materials 1, 2). Thus given our sampling we shall refrain from splitting *Up.congensis* in two different species. However, to account for the morphological differences between the two morphotypes, we describe a new variety Uvariopsiscongensisvar.angustifolia Dagallier & Couvreur corresponding to the East African morphotype (see Taxonomic Treatment below).

Collection ATBP 666 (African Tropical Biodiversity Programme, MO04937010) collected from the Budongo Forest Reserve in Uganda (as “*Uvariopsis* sp. nov. Uganda” in Fig. 1 and Suppl. materials 1, 2), is a sterile specimen tentatively identified as *Up.congensis*. Indeed, the leaves of this specimen resemble *Up.congensis* except for its longer petioles when compared to typical *Up.congensis* (ca. 6 mm vs. 2.5–5 mm in *Up.congensis*). Interestingly, our results recovered this specimen as sister to the clade formed by *Up.lovettiana* and *Up.congensis*, with strong support (Fig. 1, Suppl. materials 1, 2) and thus genetically very different from *Up.congensis*. This specimen could thus be an undescribed species. Unfortunately, the available material is too poor for a proper species description. This species might be rare occurring only in the Budongo Forest Reserve. Plant surveys of this locality are relatively old (Synnott 1985) and it has recently been assessed as a key biodiversity area (Plumptre et al. 2019). Similarly, a specimen collected from Kigoma area in western Tanzania (Kyoto University Expedition (KUE) 1039, EA000009849), and referred to as “*Uvariopsis* sp. nov. Tanzania” (Fig. 1, Suppl. materials 1, 2), appears to be a new taxon. Morphologically, it is close to *Up.lovettiana* but differs in having lamina with rounded base (vs. acute to decurrent), and subsessile pubescent monocarps (vs. stipitate and glabrous). The sequenced specimen is recovered as sister to either *Up.noldeae* with strong support (Suppl. material 2) or to the group formed by *Up.noldeae*, *Up.dioica* and *Up.congolana* (Fig. 1, Suppl. material 1) with weak support. This uncertainty probably comes from the fact that the specimen has low overall sequencing statistics (22% of the total reference covered at at least 10×; mean depth 6.1×). This would also explain its long sustaining branch. Nevertheless, the specimen passed our quality thresholds and is included. Our results confirm that it is a new species, although its phylogenetic placement remains uncertain. These new species emphasize once again the great diversity of the East African region, with locations (mainly forest remnants) containing numerous endemic species or genera (e.g. Scharff 1992; Couvreur et al. 2009; Gosline et al. 2019; Dagallier et al. 2021).

### ﻿*Uvariodendron*

Morphologically, the genus *Uvariodendron* R.E.Fr. represents the typical Annonaceae flower (van Heusden 1992) and does not stand out by any particular character (in contrast to *Uvariopsis* for example with its 2 sepals and generally 4 petals, see above). Species of this genus can be recognized, however, by the combination of the following characters: the flowers are generally large in size, bisexual, sessile or shortly pedicellate (generally less than 2 cm, but up to 6.5 cm in *Ud.anisatum*) with numerous bracts, 3 sepals and two whorls of 3 free and sub-equal petals. The flower bud is globose, and in bud, the petals of the outer whorl are valvate all along their margins, whereas the petals of the inner whorl are valvate only at the apex. The stamens are numerous (ca. 200 to 3000) and there are generally many carpels (5 to 160 in number). The monocarps are sessile to shortly stipitate (with stipes up to 12 mm long). Based on our dense species sampling, the genus is retrieved as monophyletic with strong support (Fig. 1, Suppl. materials 1, 2). We discuss below some implications of our results at the taxonomic level.

#### ﻿East African *Uvariodendron*’s: an endless source of botanical discoveries

East Africa is a region that harbours a great plant diversity, with several species being endemic or narrow endemics (Scharff 1992; Burgess et al. 1998, 2007; Couvreur et al. 2009; Gosline et al. 2019; Dagallier et al. 2021; Cheek et al. 2022). This pattern is known to be the result of the topographic heterogeneity of this region (Droissart et al. 2018; Guillocheau et al. 2018) that could promote both recent divergence and persistence of more anciently lineages through time (Fjeldså and Lovett 1997; Rahbek et al. 2019; Dagallier et al. 2020).

Recently, we described three new *Uvariodendron* species from the East African coastal forests based on their morphology: *Uvariodendrondzomboense* Dagallier, W.R.Q. Luke & Couvreur, *Uvariodendronmbagoi* Dagallier & Couvreur, and *Uvariodendronschmidtii* W.R.Q. Luke, Dagallier & Couvreur (Dagallier et al. 2021). Here, our phylogenetic analyses retrieved these species as monophyletic, confirming their species status (Fig. 1, Suppl. materials 1, 2).

**Figure 3.**
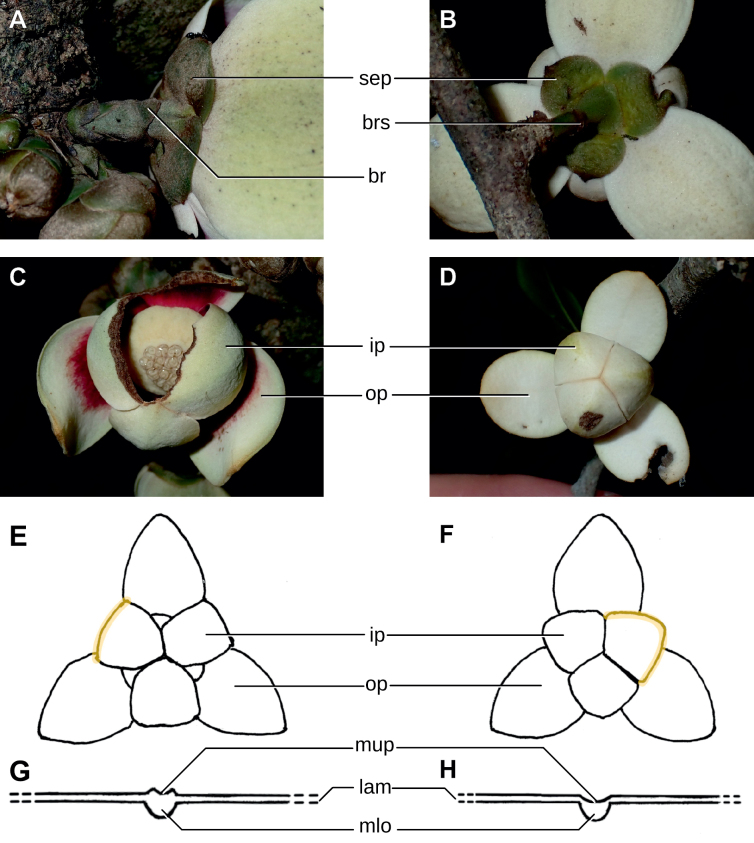
Morphological characters differentiating *Uvariodendronkimbozaense* Dagallier (**A, C, E, G**) and *Uvariodendronkirkii* Verdc. (**B, D, F, H**) **A** flowering pedicel with bracts and sepals, side view, note the imbricate sepals **B** flowering pedicel with bract scar and sepals **C** flower, with one outer petal and two inner petals gnawed, top view, note the slight transversal curvature of the inner petals **D** flower, top view, note the ‘boat-shaped’ inner petals **E, F** simplified representations of the flowers, from above, yellow marking highlights transversal curvature of the petals **G, H** Simplified representations of the transversal cut of the leaf, by the midrib. **A, C** Dagallier 49 (type) **B, D** Dagallier 23. Photos and drawings Léo-Paul Dagallier. Abbreviations: B, bract; brs bract scar; ca, carpel; ip, inner petal; lam, lamina; mlo, lower (abaxial) side of the midrib; mup, upper (adaxial) side of the midrib; op, outer petal; sep, sepal.

In addition to these recently described species, our results reveal another undescribed species. It is represented by four specimens sampled here that were previously identified as *Uvariodendronkirkii*. These specimens all come from a restricted area in the Kimboza Forest Reserve (Morogoro, Tanzania), and this species is thus described as *Uvariodendronkimbozaense* Dagallier & Couvreur. Phylogenetically, it is sister to *Ud.kirkii* although clearly divergent at the molecular level (Fig. 1, Suppl. materials 1, 2). Morphologically, it also resembles *Ud.kirkii*, but differs by having larger flowers and different leaf dimensions (see Fig. 3, Table 2 and Taxonomic Treatment). Although the Kimboza Forest Reserve covers just 4.05 km^2^ of lowland rain forest, the description of yet another new tree species underlines once again the major biological importance of this forest for biodiversity conservation (Rodgers et al. 1983). To date, an unpublished check list (see http://www.mikepalmer.co.uk/woodyplantecology/tropical/Kimboza.html) reports that Kimboza harbors 18 strict endemic plant species, including the monotypic Annonaceae genus *Mwasumbia* (Couvreur et al. 2009) as well as many other trees such as *Colakimbozensis* Cheek and *C.quentinii* Cheek (Cheek et al. 2007), *Turraeakimbozensis* Cheek (Cheek 1989) or *Vitexmorogoroensis* Walsingham & S.Atkins. (Cheek et al. 2019). This suggests that Kimboza is “probably the richest site for forest limestone point endemic plant species in tropical Africa” (Cheek et al. 2007, 2019).

**Table 2.** Morphological comparison between *Uvariodendronkimbozaense* and *Uvariodendronkirkii* (see also Fig. 3). In bold: character unique to the species.

	Lamina base	Midrib relief above	Flower bud	Bracts	Sepals	Petals size	Inner petals shape
*Uvariodendronkirkii*	acute to **decurrent**	slightly impressed (Fig. 3H)	globose, **1.3–6 mm diameter**	1 at base of the pedicel and sometimes 1 on the upper half of the pedicel (Fig. 3B)	3–6.5 mm long, 4–8 mm wide, very broadly to broadly ovate, **connivent** (Fig. 3B)	10–20 mm long, 6–13 mm wide	**strongly** transversally curved (“boat-shape”) (Fig. 3D, F)
*Uvariodendronkimbozaense*	acute to **rounded**	slightly raised with a central groove all along (Fig. 3G)	globose to oblate, **7–16 mm diameter**	1 at base of the pedicel and from **1 to 4 along the pedicel** (Fig. 3A)	6–12 mm long, 12–21 mm wide, depressed ovate, **imbricate** (Fig. 3A)	16–39 mm long, 9–19 mm wide	**slightly** transversally curved (Fig. 3C, E)

In the Flora Zambesiaca (Robson 1960) a potential new species of *Uvariodendron* was documented without being named because of the scarcity of the material. It is only known from a single specimen (Mendonça 2558A) collected in 1944 in Mozambique. This specimen was sequenced here and it appears genetically distinct from the other *Uvariodendron* species, being sister to the group of several other East African species (*Ud.gorgonis*, *Ud.schmidtii*, *Ud.dzomboense* and *Ud.pycnophyllum*) with strong support (see *Uvariodendronmossambicense* in Fig. 1 and Suppl. materials 1, 2). Our results thus confirm that this specimen represents a new species. Morphologically, it is very similar to *Ud.dzomboense* by the short leaves (less than 130 mm long), narrowly elliptic and acute to slightly decurrent laminas, and the globose, ca. 5 mm in diameter and sessile flower buds. It differs by the number of carpels (ca. 5 vs. 50–75 in *Ud.dzomboense*) (Table 3). The description from the Flora Zambesiaca was based on a sheet from LISC. Our examination of this species is based on a sheet from WAG that contains a single monocarp, thus slightly enhancing the initial description of the species (Robson 1960). The monocarp has a short stipe (ca. 12 mm long) which also differentiates it from *Ud.dzomboense* that has sessile monocarps. Even if the material remains scarce for a complete description, we provide evidence from both phylogeny and morphology that this species is distinct from the other known *Uvariodendron* species. We thus name this species *Uvariodendronmossambicense* Robson ex Dagallier & Couvreur and provide a description as complete as possible (see the Taxonomic Treatment section).

**Table 3.** Morphological comparison between *Uvariodendrondzomboense* and *Uvariodendronmossambicense*. In bold: character unique to the species.

	Lamina shape	Lamina size	Flower bud	Carpels number
*Uvariodendrondzomboense*	elliptic to narrowly elliptic, acute to slightly decurrent at base, attenuate at apex	65–132 mm long, 20–45 mm wide	globose, **ca. 4.5 mm in diameter**, pubescent	**50 to 75**
*Uvariodendronmossambicense*	elliptic, acute to slightly decurrent at base, attenuate at apex	80–135 mm long, 30–50 mm wide	globose, **ca. 6.5 mm in diameter**, pubescent	**ca. 5**

East Africa has been relatively well explored botanically, as shown by the completion of the Flora of Tropical East Africa (Beentje 2015) and the publication of several parts of the Flora Zambesiaca (Exell and Wild 1960). However, our descriptions of several new species suggest that other botanical discoveries might arise from further exploration (Cheek et al. 2022). This stresses the importance of local botanical initiatives (e.g. Binggeli 2019; Bingham et al. 2021; Hyde et al. 2021).

#### ﻿The morphological continuum of *Uvariodendronfuscum*

In the Flora of Cameroon (Couvreur et al. 2022), the name *Uvariodendronmirabile* R.E.Fr. was synonymized under *Uvariodendronfuscum* (Benth.) R.E.Fr. Here we sequenced both the type (Preuss 1378) and paratype (Lehmbach 178) of the name *Ud.mirabile*, both specimens clustering with specimens identified as *Ud.fuscum* (e.g. Mildbraed 6428, a paratype of the name *Ud.fuscum*) (Fig. 1, Suppl. materials 1, 2), confirming they are conspecific. Note that when described, *Ud.occidentale* was distinguished from *Ud.fuscum* (as *Ud.mirabile*) based on morphology (Le Thomas 1967). This holds up from a phylogenetic point of view as *Ud.occidentale* is clearly separated from *Ud.fuscum* (Fig. 1, Suppl. materials 1, 2).

**Figure 4.**
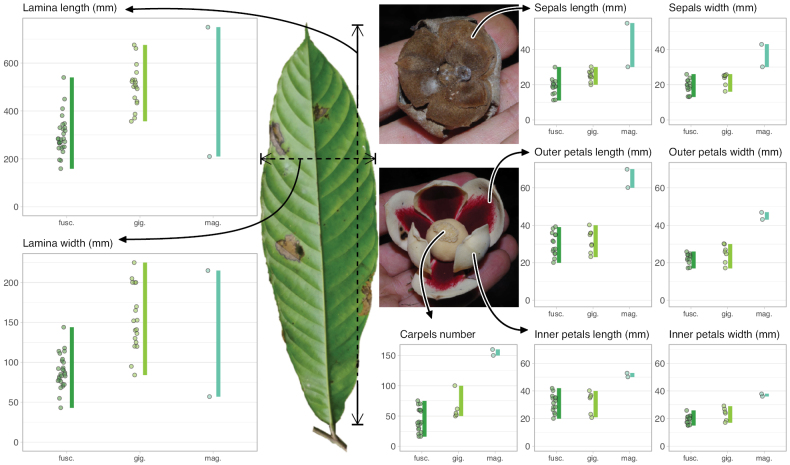
Morphological continuum in *Uvariodendronfuscum*: traits comparison for var. fuscum (fusc.), var. giganteum (gig.) and var. magnificum (mag.). Each point represents a measurement. Vertical bars span the minimum and maximum values of the focal traits. Photos, left: leaf of Couvreur 1046; top right: flower, below view, of Couvreur 1046; below right: flower, top view, of Couvreur 990 (CC BY-NC 4.0 Thomas Couvreur).

Morphologically, *Ud.fuscum* (Benth.) R.E.Fr., *Ud.giganteum* (Engl.) R.E.Fr. and *Ud.magnificum* Verdc. share morphological similarities and differences. They mainly diverge along a morphological continuum by the size of their leaves (lamina length) and the size of their flowers (sepals and petals dimensions, number of carpels), with *Ud.magnificum* having larger leaves and flowers than *Ud.giganteum*, with the latter having larger leaves and flowers than *Ud.fuscum* (Fig. 4, Table 4). In addition, *Ud.magnificum* flowers have apically imbricate inner petals while the other *Uvariodendron* species have apically connate inner petals (Verdcourt 1969). In terms of similarities, they all present elliptic to obovate leaves. *Uvariodendronfuscum* and *Ud.giganteum* have similar sepals (fused over up to half of their length), inner petals (free, obovate) and outer petals (free, ovate). *Uvariodendrongiganteum* and *Ud.magnificum* have young branches covered with long and soft hairs producing a whitish appearance quickly falling off with age, whereas the young branches of *Ud.fuscum* vary from being sparsely pubescent to glabrous. Some of the characters that supposedly discriminate the species are overlapping (e.g. lamina length), particularly concerning *Ud.fuscum* and *Ud.giganteum*.

**Table 4.** *Uvariodendronfuscum*: morphological comparison between var. fuscum, var. giganteum and var. magnificum. In bold: character useful to differentiate the varieties.

*Ud.fuscum*	Young branches indumentum	Lamina size	Sepals size	Petals size	Carpels number
var. fuscum	**sparsely pubescent to glabrous**	160–450 mm long, 43–118 mm wide	**11–23 mm long**, 13–26 mm wide	20–42 mm long, 15–26 mm wide	20 to 70
var. giganteum	with long soft hairs producing a whitish appearance quickly falling off	357–676 mm long, 84–225 mm wide	**20–30 mm long**, 16–26 mm wide	21–40 mm long, 17–30 mm wide	50 to 100
var. magnificum	with long soft hairs producing a whitish appearance quickly falling off	210–750 mm long, 57–215 (250) mm wide	**30–55 mm long**, 30–43 mm wide	50–70 mm long, 36–47 mm wide	**150 to 160**

Phylogenetically, the specimens of the three species clustered together, with strong support at the crown node and a weak to moderate support at the internal nodes (Fig. 1, Suppl. materials 1, 2). The phylogenetic branches are short (Fig. 1, Suppl. material 2) indicating a low divergence between the specimens and both *Ud.fuscum* and *Ud.giganteum* are retrieved as paraphyletic. This suggests that gene flow is still occurring between populations. We thus propose that the three described species are actually three different morphotypes of the same species. *Uvariodendronfuscum* represents the smaller morphotype (small leaves and flowers relatively to the others), *Ud.magnificum* represents the greater morphotype (large leaves and flowers relatively to the others), and *Ud.giganteum* represents the intermediate morphotype. The imbrication of inner petals of *Ud.magnificum* that Verdcourt (1969) described as a synapomorphy for the species should be considered as a developmental constraint imposed by the size of the petals. Indeed, in bud, we can imagine that the only way for such long (50–53 mm long) and wide (36–38 mm wide) petals to arrange with the other floral elements is to overlap.

The name *Ud.giganteum* was already reduced into synonymy with *Ud.fuscum* in the Flora of Cameroon (Couvreur et al. 2022). Here we thus synonymize the name *Ud.magnificum* with *Ud.fuscum*. The first mention of *Ud.fuscum* dates back to 1862, as *Uvariafusca* (Bentham 1862), whereas the name *Ud.magnificum* dates back to 1969 (Verdcourt 1969). Given the priority rule (Turland et al. 2018), the name *Uvariodendronfuscum* prevails. The description of the species is given in the Taxonomic Treatment section below.

#### ﻿The complexity of *Uvariodendronmolundense*

The species *Uvariodendronmolundense* (Engler & Diels) R.E.Fr. is distributed in Cameroon and Gabon. In 1969, Le Thomas synonymized the names *Uvariodendronmayumbense* and *Uvariodendronletestui* with *Ud.molundense*. Indeed, the position of the flowers along the trunk and branches, previously used to discriminate these species (Fries 1930), was no longer valid as some specimens are both cauliflorous and ramiflorous (Le Thomas 1969). Le Thomas (1969) also described the variety Uvariodendronmolundensevar.citrata form some specimens from Gabon exhibiting a strong lemon scent. The examination of 106 specimens identified as *Ud.molundense* revealed that this species shows a great morphological variability, especially for the leaf shape. For example, some specimens show young leaves with decurrent bases, whereas older leaves have a rounded base. The specimens identified as *Ud.molundense* form three phylogenetically supported groups separated by relatively high molecular divergence (Fig. 1, Suppl. materials 1, 2), at least as much molecular divergence as between other clearly morphologically divergent species (e.g. between *Ud.dzomboense* and *Ud.pycnophyllum*). Although the three groups have morphological similarities, we were able to distinguish them and we thus propose to separate them in three distinct species: *Ud.molundense*, *Ud.citriodorum* (Le Thomas) Dagallier & Couvreur, and *Ud.pilosicarpum* Dagallier & Couvreur. The full and valid descriptions of *Ud.citriodorum* and *Ud.pilosicarpum* are given in the Taxonomic Treatment section.

## ﻿Taxonomic revision of *Dennettia*, *Uvariodendron* and *Uvariopsis*

### ﻿Taxonomic history

*Uvariodendron*

The names referring to species later placed within *Uvariodendron* were first described by Bentham (1862) as *Uvariaconnivens* and *Uvariafusca* and by Engler and Diels (1899) as *Uvariaangustifolia* and *Uvariagigantea* (Engler and Diels 1899). The placement of these four tree species within the mainly liana genus *Uvaria* L. was based on similarities of the flowers. Based on this, Engler and Diels (1901) grouped them into the new section within *Uvaria*: sect. Uvariodendron Engl. & Diels (literally meaning “tree *Uvaria*’s”). For the next 30 years, several new tree species from Central and East Africa were described within this section: *Uvariamegalantha* Diels and *Uvariawinkleri* Diels (Diels 1907); *Uvariamolundensis* Diels and *Uvariapycnophylla* Diels (Diels 1915); *Uvarialetestui* Pellegrin (Pellegrin 1920); *Uvariagossweileri* Exell and *Uvariamayumbensis* Exell (Exell 1926) and finally *Uvariamannii* Hutch. & Dalziel (Hutchinson and Dalziel 1927b).

In 1930, Fries (1930) revised several Annonaceae genera, and erected sect. Uvariodendron to genus status. The morphological characters that distinguished *Uvariodendron* from the *Uvaria* L. were the tree habit (vs. liana in *Uvaria*), the indumentum of simple hairs (vs. generally stellate hairs) and flowers borne on the trunk or axillary (vs. terminal). Fries thus combined the “tree *Uvaria*” names cited above as: *Uvariodendronangustifolium* (Engl. & Diels) R.E.Fr., *Uvariodendronconnivens* (Benth.) R.E.Fr., *Uvariodendronfuscum* (Benth.) R.E.Fr., *Uvariodendrongiganteum* (Engl.) R.E.Fr., *Uvariodendronletestui* (Pellegrin) R. E. FR., *Uvariodendronmayumbense* (Exell) R. E. Fr., *Uvariodendronmolundense* (Diels) R. E. Fr. and *Uvariodendronpycnophyllum* (Diels) R. E. Fr. He also synonymised the name *Uvariamegalantha* Diels and *Uvariawinkleri* Diels with *Uvariodendronconnivens* (Benth.) R.E.Fr., and described three new species: *Uvariodendroncalophyllum* R.E.Fr., *Uvariodendronmirabile* R.E.Fr. and *Uvariodendronusambarense* R.E.Fr. (Fries 1930). A few years later, Exell and Mendonça combined the Angolan *Uvariagossweileri* into *Uvariodendrongossweileri* (Exell and Mendonça 1937). In 1955, Verdcourt published the new species *Uvariodendronanisatum* Verdc. from Kenya (Verdcourt 1955). Towards the end of the 1960’s, several new species were described. Le Thomas described the new *Uvariodendronoccidentale* Le Thomas distinguishing it from *Uvariodendronmirabile* R.E.Fr. (Le Thomas 1967). In her Flore du Gabon, Le Thomas also described a new variety Uvariodendronmolundensevar.citrata Le Thomas, and synonymised the names *Uvariodendronletestui* Pellegrin and *Uvariodendronmayumbense* (Exell) R.E.Fr. with *Uvariodendronmolundense* (Diels) R.E.Fr. (Le Thomas 1969). The same year, Verdcourt (1969) described three new species from East Africa: *Uvariodendrongorgonis* Verdc., *Uvariodendronkirkii* Verdc., and *Uvariodendronmagnificum* Verdc. (Verdcourt 1969). Later, he also described *Uvariodendronoligocarpum* Verdc. (Verdcourt 1986), a species that was recently combined into *Polyceratocarpusoligocarpus* (Verdc.) Dagallier (Dagallier et al. 2021). Finally, Dagallier et al. (2021) published three new species from Kenyan and Tanzanian coastal forests: *Uvariodendrondzomboense* Dagallier, W.R.Q. Luke & Couvreur, *Uvariodendronmbagoi* Dagallier & Couvreur, and *Uvariodendronschmidtii* W.R.Q. Luke, Dagallier & Couvreur. In the Annonaceae treatment for the Flora of Cameroon (Couvreur et al. 2022) the name *Uvariodendronmirabile* R.E.Fr. was synonymized with *Uvariodendronfuscum* (Benth.) R.E.Fr., as well as the name *Uvariodendrongiganteum* (Engl.) R.E.Fr., that was combined into the variety Uvariodendronfuscumvar.giganteum. Here, based on molecular and morphological analysis, we reassess the name *Uvariodendronmolundense* into three species: *Ud.molundense*, *Ud.citriodorum* comb. et stat. nov. (ex. Ud.molundensevar.citrata) and *Ud.pilosicarpum* sp. nov. and describe *Ud.kimbozaense* sp. nov. and *Ud.mossambicense* sp. nov. from East Africa. We also combine the name *Ud.magnificum* within *Ud.fuscum* as the variety Ud.fuscumvar.magnificum comb. et stat. nov. Thus to date, and after this taxonomic work, *Uvariodendron* contains 18 species.

Note about the genus name *Uva*: According to Kuntze, who undertook nomenclatural work between the end of the 19^th^ and the beginning of the 20^th^ centuries, the generic name *Uva* should be used instead of *Uvaria* (Kuntze 1891). Indeed, Burman was the first to use the name *Uva* in his Thesaurus Zelanica (Burman 1736), but Linné changed the name to *Uvaria* while citing the same specimen and describing the same species (Linné 1747). Kuntze underlines that no nomenclatural rule precludes the use of the name *Uva* and favours its use over the name *Uvaria* (Kuntze 1891). Among other invalid *Uvaria* names, he corrects the names *Uvariagigantea* Engler to *Uvagigantea* Engler, *Uvariaangustifolia* Engler & Diels to *Uvaangustifolia* Engler & Diels (Kuntze 1891), *Uvariaconnivens* Benth. to *Uvaconnivens* Benth. and *Uvariafusca* Benth to *Uvafusca* Benth. (Kuntze 1903). Regardless, the use of *Uva* was apparently not followed by the scientific community even today as the name *Uvaria* continues to be used (Diels 1907; Meade and Parnell 2018; Turner 2018).

#### ﻿*Dennetia* and *Uvariopsis*

The genus *Uvariopsis* Engler was erected in 1899 with the description of *Uvariopsiszenkeri* Engler (Engler and Diels 1899). As circumscribed at the time, the genus was distinguished by its mainly unisexual flowers, two sepals and four fused petals. A few years later, Diels erected the genus *Tetrastemma* Diels with the description of *Tetrastemmadioicum* Diels (Diels 1907). *Tetrastemma* was also characterised by unisexual flowers with two sepals and four petals, but differed from *Uvariopsis* by being dioecious and cauliflorous (vs. monoecious and ramiflorous), and by having free petals (vs. fused at base). Based on the free petals character, four other *Tetrastemma* species were described in the following years: *Tetrastemmasolheidii* De Wild. in 1909 (De Wildeman 1909), *Tetrastemmapedunculosum* Diels and *Tetrastemmasessiliflorum* Mildbr. & Diels in 1915 (Diels 1915), and *Tetrastemmabakerianum* Hutch. & Dalziel in 1927 (Hutchinson and Dalziel 1927a).

In parallel, two other genera, morphologically closed to *Uvariopsis* and *Tetrastemma* were described: *Thonnera* De Wild. and *Denettia* Baker f. The genus *Thonnera* included a single species *Thonneracongolana* De Wild. differing from *Tetrastemma* by flowers having three petals (De Wildeman 1909). In 1953, however, Fries combined this name into *Uvariopsis* as *Uvariopsiscongolana* (De Wild.) R.E.Fr. without further justification (Fries 1953). The genus *Denettia* Baker f. was characterised by having bisexual flowers with three sepals and three petals, which differed from *Tetrastemma*, *Thonnera*, and *Uvariopsis*. It included a single species *Denettiatripetala* Baker f. (Baker 1913).

In 1933, Robyns & Ghesquière described *Uvariopsisvanderystii* Robyns & Ghesq., and suggested it was a kind of intermediate species showing characters of *Tetrastemma*, e.g. cauliflory, and of *Uvariopsis*, e.g. fused petals (Robyns and Ghesquière 1933b). The same year, they revised the two genera with their ‘Essai de révision des genres *Uvariopsis* Engl. & Diels et *Tetrastemma* Diels (Annonacées)’ (Robyns and Ghesquière 1933a). In this publication, they described several new species: *Uvariopsisbatesii* Robyns & Ghesq., *Uvariopsischevalieri* Robyns & Ghesq. and *Uvariopsiscongensis* Robyns & Ghesq. The later species is ramiflorous and has free petals, which again showed intermediate characters between *Uvariopsis* and *Tetrastemma*. Based on these intermediate species, Robyns & Ghesquière (1933a) extended the morphological concept of *Uvariopsis* and combined the name *Tetrastemma* with *Uvariopsis*. The new species combinations were: *Uvariopsisbakeriana* (Hutch. & Daltz.) Robyns & Ghesq., *Uvariopsisdioica* (Diels) Robyns & Ghesq., *Uvariopsispedunculosa* (Diels) Robyns & Ghesq., *Uvariopsissessiliflora* (Mildb. & Diels) Robyns & Ghesq., *Uvariopsissolheidii* (De Wild.) Robyns & Ghesq. In 1948, Pellegrin described *Uvariopsisletestui* Pellegr., a species from Gabon (Pellegrin 1948), and three years later Exell and Mendonça described a species from Angola: *Uvariopsisnoldeae* Exell & Mendonça (Exell and Mendonça 1951). In 1952, Keay published the species *Uvariopsisglobiflora* Keay (Keay 1952). He also combined a species previously described by Chevalier under the name *Uvariaspectabilis* Chev. (Chevalier 1920) and cited in the Flora of Tropical West Africa as *Uvariaspectabilis* Chev. ex Hutch. & Dalziel (Hutchinson and Dalziel 1927a) into *Uvariopsisguineensis* (Chev. ex Hutch. & Dalziel) Keay (Keay 1952). More than 30 years later, Verdcourt described *Uvariopsisbisexualis* Verdc. from Tanzania. Although this species has bisexual flowers, Verdcourt placed it in *Uvariopsis* based on pollen morphology closely ressembling species of *Uvariopsis* (*Verdcourt 1986*). The concept of *Uvariopsis* was thus enlarged to include bisexual species.

Gereau and Kenfack (2000) described the species *Uvariopsiskorupensis* Gereau & Kenfack followed by the publication of *Uvariopsissubmontana* Kenfack, Gosline & Gereau (Kenfack et al. 2003). In that later publication, the name *Denettiatripetala* Baker f. was combined into *Uvariopsistripetala* (Baker f.) G.E. Schatz. based on the concept of *Uvariopsis* now also included species with bisexual flowers (Verdcourt 1986; Kenfack et al. 2003). More recently, the Tanzanian species *Uvariopsislovettiana* Couvreur & W.R.Q. Luke (Couvreur and Luke 2010) and the Gabonese species *Uvariopsiscitrata* Couvreur & Niangadouma (Couvreur and Niangadouma 2016) were described. In the Flora of Cameroon Annonaceae (Couvreur et al. 2022), the species *Uvariopsisetugeana* Dagallier & Couvreur was described and the name *Uvariopsisvanderystii* Robyns & Ghesq. was synonymized with *Uvariopsispedunculosa* (Diels) Robyns & Ghesq. Finally, the species *Uvariopsisdicaprio* Cheek & Gosline was described from Cameroon (Gosline et al. 2022). In the present work, based on molecular and morphological analyses, we retain the name *Dennettiatripetala* and thus the genus *Dennettia* (see discussion above). We also describe a new species, *Up.oligocarpa* sp. nov., and synonymize the names *Up.sessiliflora* with *Up.dioica*, *Up.globiflora* with *Up.guineensis* and *Up.letestui* with *Up.solheidii*. For the two laters, we combine the names into varieties as Up.guineensisvar.globiflora comb. et stat. nov. and Up.solheidiivar.letestui comb. et stat. nov., respectively. We also describe a new variety of *Up.congensis*, Up.congensisvar.angustifolia var. nov. and provide a preliminary description of potentially three new species which lack sufficient material to be formally described. This brings the total number of *Uvariopsis* species to 17 (and three that have yet to be formally described).

### ﻿Morphology and informative characters

#### ﻿Vegetative characters

The genera *Dennettia*, *Uvariodendron* and *Uvariopsis* are trees or shrubs with plagiotropic branches on an orthotropic axis, with indumentum of simple hairs and with no latex or exudate. A distichous phyllotaxis of the primary axis (the trunk) was reported for *Ud.pycnophyllum* (Johnson 2003) but no information was reported for the other species nor for *Dennettia* and *Uvariopsis*. The trunks are generally cylindrical and straight, with no buttresses. The base of the trunk of some *Uvariopsis* species (*Up.dioica*, *Up.submonana*, *Up.korupensis*) can be thickened by the growth of numerous inflorescences clumped on the trunk between the ground to generally ca. 50 cm (up to 4 m).

The branches can be sparsely pubescent when young, but the hairs generally fall off with age. The dense brown tomentum on the young branches persisting on older branches distinguishes *Ud.calophyllum* from the other species of *Uvariodendron*. The species *Ud.fuscum* (var. giganteum and var. magnificum) also have a characteristic indumentum of long soft hairs producing a whitish appearance on the younger branches, but that quickly disappear in older branches.

In the three genera, the leaves are distichous, simple, entire, exstipulate, with the midrib impressed above, raised below, the secondary veins weakly brochidodromous to brochidodromous and the tertiary veins reticulate. *Dennettia* has relatively small (less than 160 mm long) elliptic leaves, while *Uvariodendron* and *Uvariopsis* have a range of leaf shapes, from elliptic to obovate to oblong, ranging from 65 to 750 mm long. Uvariodendronfuscumvar.giganteum has one of the longest leaves of Annonaceae in Africa, up to 60 cm long (Couvreur et al. 2022). In contrast to other genera (e.g. *Uvariastrum*; Couvreur 2014) the leaf base shape is not a very useful taxonomic character to distinguish species because several different shapes (e.g. from acute to rounded) can occur within species. However, subcordate leaf bases are generally found in *Up.bakeriana*, *Up.citrata*, *Up.korupensis*, and *Up.submontana*, and decurrent leaf bases in *Ud.pilosicarpum*, *Up.bisexualis*, *Up.congensis*, *Up.lovettiana*, *Up.oligocarpa* and *Up.zenkeri*. Some species have apparent complex leaf bases, like rounded in shape but minutely decurrent when looking at the very base (*Ud.citriodorum*, *Ud.gorgonis*, *Ud.molundense*). In *Uvariodendronkimbozaense*, the midrib is slightly raised above with a central groove all along the length of the midrib. This is unique in the genus, but raised midribs occur in other genera such as *Isolona*, *Monodora* and *Ophrypetalum* (Couvreur 2009), and raised and grooved midribs occur in *Cremastosperma* (Pirie et al. 2018). The petiole in *Uvariopsis* is generally short and thick, less than 6 mm long with a length:width ratio less than 2, whereas in *Uvariodendron* the petiole is generally longer than 4 mm with a length:width ratio between 2 and 5.

In *Uvariodendron*, several species (*Ud.dzomboense*, *Ud.gorgonis*, *Ud.mossambicense* and *Ud.schmidtii*) show ‘eragrostiform’ leaf buds, that are terminal or axillary buds composed of several (generally 5 to 12) distichous and densely pubescent scales (Figs 25A, 36C, 44B). The term ‘eragrostiform’ relates to the genus *Eragrostis* (Poaceae) and its typical form of flattened spikelet composed of compact and clustered florets. Even though this feature is striking, it should be noted that not all the specimens of a same species present this kind of buds, so it is hard to use it as a diagnostic character. The phenology or the environment may influence the establishment of eragrostiform buds, which might protect the meristem against drought or herbivores.

The scent of crushed leaves and/or bark can be used as a distinctive character. *Uvariodendroncitriodorum* and *Up.citrata* emit a strong lemon scent (Le Thomas 1969; Couvreur and Niangadouma 2016), a feature also shared by some specimens of *Ud.gorgonis*. Crushed leaves and bark of *Ud.mbagoi* have a strong bergamot scent (between orange and lemon) (Dagallier et al. 2021), and all parts of *Ud.anisatum* smell of anise. *Ud.angustifolium* is also reported as having a strong aromatic scent.

#### ﻿Inflorescences and flowers

In contrast to sterile parts, *Dennettia*, *Uvariodendron* and *Uvariopsis* have very different fertile parts. In Annonaceae, the inflorescence is a rhipidium, i.e. determinate (sympodial) inflorescence with a terminal flower and lateral cymose and monochasial partial inflorescences (Weberling and Hoppe 1996). Inflorescences of *Dennettia*, *Uvariodendron* and *Uvariopsis* are axillary and borne on leafy twigs or borne on old branches (ramiflory) or on the trunk (cauliflory), depending on the species. The position of the flower was regarded as an important character to characterize some species like *Ud.molundense* and *Up.guineensis* (Keay 1952; Le Thomas 1967, 1969) but it appears to be unreliable in *Uvariodendron* and *Uvariopsis* (see also Le Thomas 1969). In the three genera, the flower pedicels are not articulate and display from one to six bracts at their base and from one to four bracts on the lower half of the pedicel. As flowers mature, most of the bracts fall off, except the uppermost bract. Usually, fallen bracts leave a scar on the pedicel.

In *Dennettia*, the inflorescences are axillary and borne on leafy twigs or borne on old branches, held by a tiny peduncle. They can be composed of up to six flowers, but one or two-flowered inflorescence are the most common. The flowers of *Dennettia* are bisexual. They are composed of one whorl of three sepals and one single whorl of three petals, which is characteristic of the genus in the Monodoreae. In Annonaceae, single whorls of three petals can be found in *Annickia*, *Annona*, and *Dasymaschalon* (van Heusden 1992).

In *Uvariodendron*, the inflorescences are axillary and borne on leafy twigs, borne on old branches or borne on the trunk. The axis of the inflorescences are contracted, rendering their interpretation difficult without a detailed morpho-anatomical study. They appear to be rhipidia composed of one or two (in most species), three (*Ud.calophyllum*, *Ud.gorgonis*, *Ud.mbagoi*) or rarely up to 11 (*Ud.kimbozaense*) flowers. Generally, in multi-flowered rhipidia, the flower in the terminal position is fully developed while the lower flowers on the axis are buds.

Flowers in *Uvariodendron* are bisexual with one whorl of three sepals and two whorls of three petals, as usually found in Annonaceae (van Heusden 1992). The fusion and arrangement of the sepals is used to distinguish between species: always fused (*Ud.angustifolium*, *Ud.dzomboense*, *Ud.fuscum*, *Ud.mossambicense*, *Ud.occidentale*, *Ud.pycnophyllum*, *Ud.schmidtii*), always imbricate (*Ud.citriodorum*, *Ud.kimbozaense*, *Ud.mbagoi*, *Ud.pilosicarpum*), or from free to fused to imbricate (*Ud.anisatum*, *Ud.calophyllum*, *Ud.connivens*, *Ud.gorgonis*, *Ud.kirkii*, *Ud.molundense*). The sepals are smaller and morphologically different to the petals. The outer and inner petals are subequal in length, ranging from 10 to 70 mm at anthesis. In bud, the outer petals are valvate (adpressed against each other all along the margin) whereas inner petals are valvate only at the apex. In mature flowers, the outer and inner petals are free, but the inner petals can remain adpressed at apex. In most species, the petals are cream to yellow in color, with a red/purple marking at the base of the inner side. *Uvariodendronconnivens* has petals that are pink to red all over and on both sides at anthesis, while the outer petals of *Ud.calophyllum* are covered with a dense tomentum leading to a brown flower. The stamens are numerous (more than 200), with linear anthers and truncate apical prolongation of the connective. The carpels vary from five to 160, they are free, linear, with a coiled stigma. The monocarps are sessile or subsessile and cylindrical.

*Uvariopsis* has inflorescences borne on trunk in most of the species, but can be axilary to leaves on young branches (*Up.congensis*, *Up.guineensis*, *Up.lovettiana*, *Up.oligocarpa*, *Up.zenkeri*). They are generally composed of one to three flowers. Like in *Uvariodendron*, the peduncle and partial peduncle are contracted, rendering their interpretation as rhipidium difficult. In particular, *Up.dioica*, *Up.submontana* and *Up.korupensis* have inflorescences in clumps of up to 50 flowers on the trunk.

The flower buds of *Uvariopsis* have different shapes important to discriminate between species (Kenfack et al. 2003). They can be globose (*Up.congensis*, *Up.guineensis*, *Up.lovettiana*, *Up.oligocarpa*, *Up.pedunculosa*, *Up.zenkeri*), ovoid to conical (*Up.bisexualis*, *Up.citrata*, *Up.dioica*, *Up. noldeaea Up.solheidii*, *Up.submontana*), pyramidal (*Up.congolana*, *Up.dicaprio*, *Up.etugeana*), or narrowly and long conical (*Up.bakeriana*, *Up.korupensis*). Flowering pedicels in *Uvariopsis* varies greatly from less than 11 mm (*Up.bakeriana*, *Up.citrata*, *Up.congensis*, *Up.etugeana*, *Up.oligocarpa*, and *Up.zenkeri*) to more than 100 mm up to 400 mm (*Up.congolana*, *Up.pedunculosa*). The flowers of *Uvariopsis* have one whorl of two sepals and one whorl of four petals (except *Up.congolana* which has three petals). In Annonaceae, flowers with two sepals and four petals are rare and can be found in only a few other genera such as *Disepalum* or *Tridimeris* (van Heusden 1992). The petals are usually free, but can be fused in some species (*Up.congolana*, *Up.guineensis*, *Up.korupensis*, *Up.pedunculosa*, *Up.submontana*, and *Up.zenkeri*). Another rare character in Annonaceae exhibited by *Uvariopsis* is its exclusively unisexual flowers (except for *Up.bisexualis* that has bisexual flowers). In Annonaceae, unisexual flowers are found in several androdioecious species, e.g. in *Polyceratocarpus* (Couvreur et al. 2009), *Diclinanona* (Erkens et al. 2014), *Ephedranthus* (Lopes et al. 2014) , *Pseudephedranthus* (Erkens et al. 2017), *Klarobelia* or *Pseudomalmea* (Lopes et al. 2018). Species with exclusively unisexual male and female flowers are rarer and found only in a few species within genera such as *Anonidium*, *Monanthotaxis*, *Pseuduvaria*, *Stelechocarpus* and *Winitia* (van Heusden 1992; Chaowasku et al. 2013; Hoekstra et al. 2021; Couvreur et al. 2022). Unisexual *Uvariopsis* species are monoecious, i.e. male and female flowers on the same individual, even in the species *Up.dioica* that has a misleading specific epithet. The number of stamens varies from 100 to 900, with anthers linear and connective prolongation truncate or absent. The number of carpels varies from three to 280; they are free, linear, with a coiled, truncate or globose stigma.

The fruits of *Uvariopsis* are composed of several sessile to subsessile monocarps generally cylindrical in shape. Their texture and pubescence can be informative: either smooth and not ridged (*Up.congensis*, *Up.dioica*, *Up.guineensis*, *Up.korpensis*, *Up.lovettiana*, *Up.oligocarpa*) or wrinkled to verrucose (*Up.bakeriana*, *Up.congolana*, *Up.pedunculosa*, *Up.solheidii*), and either pubescent to tomentose (*Up.pedunculosa*, *Up.oligocarpa*, *Up.zenkeri*) or sparsely pubescent to glabrous.

### ﻿Pollination and seed dispersal

The pollination biology of *Dennettia*, *Uvariodendron* and *Uvariopsis* is poorly known for most of the species, except for *Ud.connivens*, *Ud.calophyllum*, *Up.bakeriana* and *Up.pedunculosa* (Gottsberger et al. 2011; the specimen identified as *Up.congolana* in this reference is actually *Up.pedunculosa*), and also *Up.dioica* (Mertens et al. 2018). *Uvariodendron* are mainly pollinated by beetles (Coleoptera, Scarabaeidae and Curculionidae). The inner petals remain connate at the apex until anthesis, forming a semi-closed pollination chamber in which the pollinators feed on pollen and mate (e.g. Figs 19G, 24F). The pollinators are attracted by the scent emitted by the flowers. Flowers of *Ud.connivens* and *Ud.calophyllum* were found to be thermogenetic, which could enhance the scent diffusion and the pollinator attraction (Gottsberger et al. 2011). However, rather than active thermogenesis, it is possible that the pollination chamber buffers the air temperature from outside. The petals of *Uvariodendron* flowers are thick and the pollinators feed on the petals and deposit their eggs in the petal tissue, which is testified by the gnaw marks on the petals (e.g. Fig. 29J), similarly to other Annonaceae species having thick petals (Gottsberger and Webber 2018). Although this probably doesn’t play a role in pollination, we have seen completely eaten petals on flowers of *Ud.kimbozaense* (Fig. 27D), with animal hairs stuck on the stigma exudate (Fig. 27G), suggesting that small mammals like rodents may feed on the petals. Direct observation of such feeding on petals should, however, confirm this hypothesis.

In *Uvariopsis*, the species *Up.bakeriana* and *Up.pedunculosa* have been reported as emitting a strong pungent scent, and to be pollinated by flies (Diptera) (Gottsberger et al. 2011). *Up.dioica* has been reported to be pollinated by flies, cockroaches and orthopterans (Mertens et al. 2018), and pictures of living specimens of *Up.dioica* (Bidault 1558) and *Up.guineensis* (Bidault 4798) show flies visiting the flowers (e.g. Fig. 61F). Petals of *Up.bakeriana* are dark red to dark pink and verrucose, which looks like body flesh. This is characteristic of the sapromiophylous pollination syndrome (Gottsberger et al. 2011). Most of the *Uvariopsis* species present petals with flesh-like colors and texture. This suggests that sapromiophyly is the dominant pollination syndrome in *Uvariopsis*. In contrast, the species *Up.dicaprio* present pale yellow-green flowers, suggesting nocturnal pollinators such as moths (Gosline et al. 2022).

Similarly to pollination biology, data on seed and fruit dispersal of *Dennettia*, *Uvariodendron* and *Uvariopsis* remain scarce. Fruits of *Uvariodendron* are part of Western gorillas (*Gorillagorilla*)’s diet (Rogers et al. 2004), as well as fruits of *Up.congolana* (Yamagiwa et al. 1994) and *Up.solheidii* (notes of specimen Fay 8384). Fruits of *Up.solheidii* are also eaten by small rodents and squirrels (Gautier-Hion et al. 1985). Chimpanzees (*Pan*) and guenons (*Cercopithecus*) consume the fruits of *Up.congensis* (Lambert 2011; Watts et al. 2012; Kagoro-Rugunda and Hashimoto 2015).

### ﻿Ethnobotany

*Uvariodendron* and *Uvariopsis* species are used for food. The ripe fruits of *Ud.angustifolium* (Jones 3480) and *Ud.fuscum* (van Andel 3761) are eaten, as well as the young leaves of *Ud.connivens* (Cheek 5180) and *Ud.fuscum* (Cheek 5145) that are boiled and used in soup. The bark of *Ud.mbagoi* (Dagallier 39) is used as infusion for tea or as spice in meat meals. The leaves of *Up.citrata* (Letouzey 9017) are used to wrap the fish during the cooking to give it an aromatic taste.

*Uvariodendron* and *Uvariopsis* species are also part of the pharmacopoeia of several local communities, such as in Benin and Cameroon where *Ud.angustifolium*, *Ud.connivens*, *Ud.fuscum*, *Up.bakeriana*, *Up.dioica* and *Up.korupensis* are used to cure various conditions (Jiofack et al. 2009; Bele et al. 2011; Bada Amouzoun et al. 2019) or in Kenya where *Ud.kirkii* is used as contraceptive (Kaingu et al. 2017).

**Figure 5.**
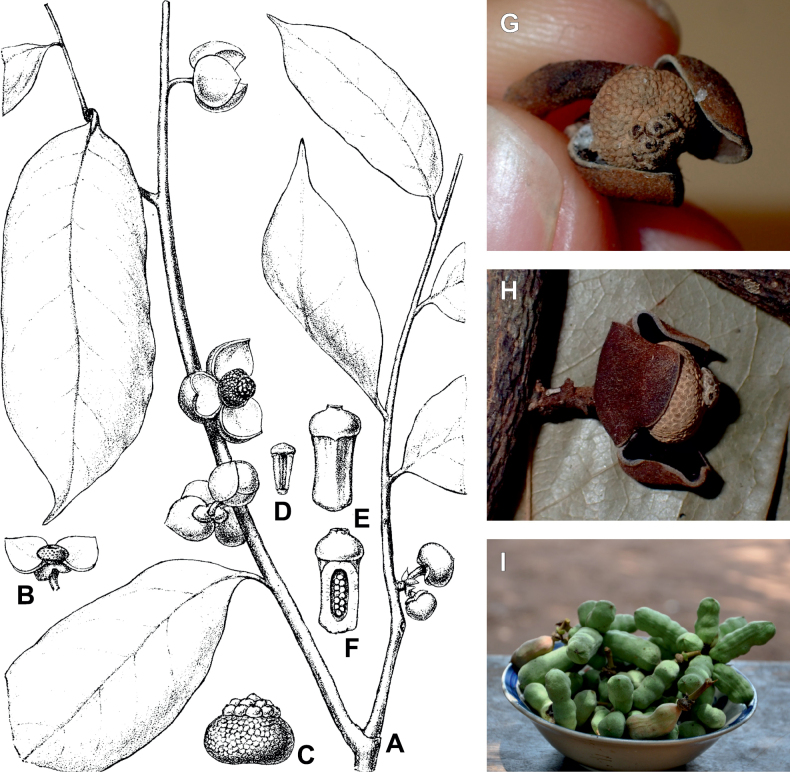
*Dennettiatripetala* Baker f **A** flowering branch **B** flower, one petal removed, showing receptacle **C** receptacle with stamens and stigmas **D** stamen, front view **E** carpel, front view **F** carpel, longitudinal section showing ovules **G** flower, top view **H** flower, side view **I** fruits collected for local use in Nigeria **A–F** material of drawings unknown, author of drawing unknown, modified from Baker (1913; plate 2) **G** Aké Assi 9543 **H** Jongkind 4356 **I** material unknown. Photos **G, H** Léo-Paul Dagallier **I** World Agroforestry (https://flic.kr/p/pZycpY).

The fruits of *Dennettiatripetala*, also known as “pepper fruit”, are famous in West Africa for their spicy taste, especially in Nigeria (Fig. 5I). The species is traditionally used for the anti-rheumatic, stimulant, antioxidant, antimicrobial and analgesic effects of its leaves, fruits, seeds, roots and stems (Aiyeloja and Bello 2006; Ajibesin et al. 2008; Iseghohi 2015; Rafiu and Sonibare 2016; Olatokunbo et al. 2018; Omage et al. 2019; Muhammed et al. 2021).

Other usages such as firewood and craft were reported for *Ud.angustifolium*, *Up.bakeriana*, *Up.dioica*, and *Up.korupensis* (Bele et al. 2011; Bada Amouzoun et al. 2019), as well as dye with fruits of *Ud.connivens* (Mbani 14).

## ﻿Taxonomic treatment

### ﻿Ophrypetaleae

#### ﻿ ﻿Ophrypetaleae

Taxon classificationPlantaeMagnolialesAnnonaceae

Dagallier & Couvreur trib. nov.

urn:lsid:ipni.org:names:77326966-1

##### Type.

*Ophrypetalum* Diels.

##### Description.

Shrubs to trees; indument of simple hairs. Carpels 1–15. Monocarps 1–10, sessile to shortly stipitate, 5 cm long or more, narrowly cylindrical, length:width ratio more than 5.

##### Included genera.

*Ophrypetalum* Diels (1 species), *Sanrafaelia* Verdc. (1 species).

### ﻿Key to the genera of Monodoreae as recognized in this study

**Table:** 

1	Midrib of leaf blade clearly raised above; one single large fruit with seeds unordered	**2**
–	Midrib of leaf blade impressed above, or slightly raised with a central groove all along the midrib above; fruits with distinct monocarps with uni- or bi-seriate seeds	**3**
2	Corolla lobes similar and equal in length, forming a distinct tube at the base, margins generally straight	***Isolona***
–	Corolla lobes clearly differentiated into inner and outer petals (but basally fused); the outer ones longer than inner ones, margins generally undulated or crisped	***Monodora***
3	Sepals 2 and at least one of: petals 4, flowers unisexual	***Uvariopsis***
–	Sepals 3 and petals 3 or 6 and flowers bisexual	**4**
4	Sepals 3, petals 3	***5***
–	Sepals 3, petals 6	**6**
5	Calyx forming a short and thick receptacular tube (East Africa)	***Lukea***
–	Calyx forming a ring with 3 lobes generally distinct (West Central Africa)	***Dennettia***
6	Petals fused at base	**7**
–	Petals free	**8**
7	Petals plicate, transversely folded in bud	***Hexalobus***
–	Petals not plicate, not folded in bud	***Asteranthe***
8	Petals in a single whorl, calyx forming a thick receptacular tube	***Monocyclanthus***
–	Petals in two distinct whorls, calyx not forming a receptacular tube	**9**
9	Receptacle columnar; anther connective reduced to a tuft of hairs	***Mischogyne***
–	Receptacle convex to flat but not columnar; connective not reduced to a tuft of hairs	**10**
10	Sepals reduplicate-valvate (ridged) in bud	***Uvariastrum***
–	Sepals not reduplicate-valvate (not ridged) in bud	***Uvariodendron***

#### ﻿ Dennettia

Taxon classificationPlantaeMagnolialesAnnonaceae

Baker f., Cat. Pl. Oban 5 (1913)

Figs 5 , 6

Dennettia tripetala Baker f., Cat. Pl. Oban 5 (1913).
≡ Uvariopsis tripetala (Baker f.) G.E.Schatz, Novon 13(4): 447 (2003). Type. Nigeria – Edo State • R.E. Dennett 44 (lectotype: K! (K000040959), designated by Kenfack et al (2003), sheet designated by Couvreur et al (2022); isolectotype: K! (K000040961)), Benin City; 6°20'N, 5°38'E; 01 Jan. 1907.

##### Description.

Shrub to tree 1–5 m tall, D.B.H unknown; young branches sparsely pubescent to glabrous, old branches glabrous. Petiole 2–5 mm long, 1–1.5 mm wide, glabrous. Leaf lamina 72–155 mm long, 30–68 mm wide, length:width ratio 1.9–3.25, elliptic, papyraceous to coriaceous, base acute to decurrent, apex attenuate, acumen 6–13 mm long, surface above glabrous, surface below glabrous; midrib impressed above, raised below, glabrous above, glabrous below; secondary veins 5–10 pairs, brochidodromous to weakly brochidodromous, slightly raised above, raised below; tertiary veins reticulate. Flowers bisexual. Flower buds globose. Inflorescences borne on branches or axilary, composed of 1 to 6 flowers. Peduncle ca. 0–1 mm long, ca. 1 mm in diameter. Flower pedicel 3.5–10 mm long, 1–2.5 mm in diameter, pubescent to sparsely pubescent. Bracts 1 to 3 at base, upper bract 0.5–2 mm long, 1.5–2 mm wide, broadly ovate, pubescent outside, glabrous inside. Sepals 3, 1–3 mm long, 1.5–4 mm wide, broadly ovate, fused up to more than 50% of their length, forming a persistent ring with the 3 lobes generally clearly distinct, curved downward on fruit pedicel, pubescent outside, glabrous inside, color unknown. Petals 3, 5–14 mm long, 4.5–10 mm wide, length:width ratio 1–1.7, broadly ovate to ovate, connivent in bud, free at anthesis, pubescent to sparsely pubescent outside, glabrate inside, light brown to dark red outside, purplish pink inside. Stamens number unknown, 0.5–1 mm long, 0.3–0.5 mm wide, anthers linear, connective prolongation truncate. Carpels 8–30, 2–4.5 mm long, 0.5–2 mm wide, pubescent, free; stigma 0.5–1 mm long, 0.5–1 mm wide, coiled, pubescent. Fruiting pedicel 5–14 mm long, 1–2.5 mm in diameter, pubescent to glabrous. Monocarps, 1–7, 11–32 mm long, 5–18 mm wide, length:width ratio 1.5–2.3, cylindrical, slightly constricted between the seeds when dried, sparsely pubescent to glabrous, greyish green when unripe to pink-reddish when ripe, subsessile; stipe 0.1–3 mm long, 1–2 mm wide, pubescent. Seeds 4–12 per monocarp, biseriate, 7.5–10 mm long, 2–5 mm wide, ellipsoid.

##### Distribution.

Element of the Upper Guinean Domain and Lower Guinean Domain of the Guineo-Congolian Region: Guinea, Sierra Leone, Ivory Coast, Ghana, Benin, Nigeria, Cameroon.

##### Habitat and ecology.

Lowland mature or secondary rain forests, in west Africa reported from drier forests on rocky outcrops (Hawthorne and Jongkind 2006). Altitude: 250–975 m a.s.l.

##### Phenology.

Flowers collected from February to April. Fruits collected from January to July.

##### Vernacular names.

Cameroon: ‘Bushpèpè’ (Westphal 9932). Ivory Coast: ‘Michiti à petites feuilles’ (Aubréville 145). Nigeria: ‘Mmimmi’ in Igbo, ‘Ata Igebere’ or ‘Igberi’ in Yoruba, ‘Opipi’ in Idoma, ‘Imako’ in Urhobo, ‘Ako’ in Bini and Edo, ‘Nkarika’ in Efik and Ibibio (Aiyeloja and Bello 2006; Ajibesin et al. 2008; Rafiu and Sonibare 2016; Omage et al. 2019; Muhammed et al. 2021).

##### Uses.

Fruits used as spice and leaves, roots, fruits and seeds used as local medicine.

##### Notes.

This species resembles *Uvariopsiscongensis*, *Up.oligocarpa* and *Up.zenkeri* in having elliptic leaves generally less than 16 cm long with decurrent base, but it clearly differs by its bisexual flowers having three sepals and three petals.

##### Preliminary conservation status.

This species is distributed from Guinea to Cameroon. Its EOO is estimated at 705,671 km^2^ and its AOO at 120 km^2^. Following IUCN criterion B, it would thus be assigned a status of LC.

##### Additional specimens examined.

Benin – Mono • J.-P. Essou 1443 (BENIN, MO, WAG); Aplahoué, Kpédjihoundéhoué; 7°21'N, 1°44'E; 17 Feb. 1999 – Ouémé • A. Akoègninou 2201 (BENIN, WAG); Kétou, Ewè; 7°28'N, 2°35'E; 01 Feb. 1999 – Zou – K. Küppers 2059 (FR, WAG), Noyau Central forêt class.Lama; 6°57'N, 2°07'E; 28 Feb. 1998. Cameroon – South-West Region • D.W. Thomas 5661 (K, MO, P, WAG), Nganjo. West bank of Meme River on Kumba Mbonge road; 4°33'N, 9°24'E; alt. 50 m; 25 Feb. 1986 • E. Westphal 9932 (WAG), Victoria; 4°01'N, 9°12'E; 04 Apr. 1978 • G.W.J. Mildbraed 10515 (K), Likomba – Pflangzung, 15–35 km NE of Victoria; 4°06'N, 9°20'E; alt. 50 m; 18 Oct. 1928 • T.D. Maitland 626 (K); Fako, Victoria (= Limbe), Bonjongo; 4°06'N, 9°08'E; alt. 600 m; Apr. 1929 • T.D. Maitland 627 (P), Victoria (= Limbe), Bonjongo; 4°06'N, 9°08'E; 1929 • T.D. Maitland s.n (K), Mt Cameroon; 4°15'N, 9°13'E; alt. 920 m; 1930 • T.D. Maitland s.n.14 (K), Mt Cameroon, Buea area; 4°12'N, 9°11'E; alt. 975 m; 1930. Ghana – Ashanti Region • J.K. Morton A3394 (K), Nyinahin range, W of Kunasi; 6°36'N, 2°07'W; alt. 700 m; 07 Jun. 1958 – Central Region • J.B. Hall GC43611 (K, WAG), Ojobi near Awutu; 5°29'N, 0°32'E; 29 Feb. 1972 • J.K. Morton s.n (K), About 1 mile N of Ojubi, Senya Beraku; 5°23'N, 0°29'E; 24 May. 1953 – Unknown major area • J.B. Hall GC43276 (K), Yaugwu F.R; alt. 460 m; 19 Mar. 1972. Guinea – Kindia • D. Molmou 603 (P), Layah, Sousoude. Just avant Tonkoyah; 9°43'39.3"N, 13°12'26.6"W; alt. 12 m; 06 Jul. 2013. Ivory Coast – Bouaflé • C.C.H. Jongkind 4356 (MO, WAG), Parc National de la Marahoue. Near south border; 6°59'N, 6°10'W; alt. 250 m; 11 Feb. 1998 – Bouaké • L. Aké Assi 18031 (G), Forêt de Bamoro; 7°48'N, 5°05'W; 07 Jun. 1989 – Bouna • C. Geerling 2222 (K, WAG), Iringou (Gawi-Bania confl. Bamago); 8°58'N, 3°39'W; 15 Mar. 1968 • P.P. Poilecot 1224 (G), P.N. Comoé Sud; 8°45'N, 3°35'W; 13 Apr. 1986 • P.P. Poilecot 4164 (G, MO), P.N. Comoé Sud; 8°45'N, 3°35'W; 13 Apr. 1986 – Danané • A.J.M. Leeuwenberg 2999 (K, L, MO, P, WAG), 2 km E of Danané; 7°16'N, 8°07'W; alt. 410 m; 06 Mar. 1959 – Oumé • A. Le Thomas 27 (P), Lamto-Station, Fleuve Bandama; 6°13'N, 5°01'30"W; 07 Jul. 1985 • A. Le Thomas 97 (P), Lamto-Station, Bandama; 6°13'N, 5°01'30"W; 26 Jul. 1985 – Sassandra • A. Aubréville 1957/163 (BR, K, P); 4°59'N, 6°08'W; 07 Mar. 1957 – Soubré • L. Aké Assi 9490 (G, P), Foret du Bassin de la Lobo; 6°07'N, 6°48'W; 22 Feb. 1967 – Vavoua • F.N. Kouamé 1470 (CSRS, G), F.C. du Haut-Sassandra, Centre. forêt très dégradée, relevé FNK20; 7°07'N, 7°00'W; 07 Apr. 1995 – Unknown major area • A. Aubréville 145 (K, P); 7°37'26.4"N, 5°33'14.4"W; 28 Feb. 1957 • L. Aké Assi 13255 (G), Mont Niénokoué; 5°23'N, 7°10'W; 19 Jan. 1976 • L. Aké Assi 9543 (G, K, P), Mt Mafa; 5°52'N, 4°04'W; 14 Mar. 1967. Nigeria – Anambra State • J.C. Okafor FHI35869 (K), Onitsha; Nnwei; Ukpor – Nzagha village; 6°50'N, 6°50'E; 22 Oct. 1956 – Cross River State • C.F.A. Onochie FHI36331 (K), Calabar, Ikot Efanga, c. 12 miles from Calabar on the Oban road; 4°58'N, 8°29'E; 09 Feb. 1957 – Oyo State • C.E. Darter 41837 (K), Olokemeji Forest Reservee; agbola pool, Ogun River; 7°25'N, 3°32'E; 06 Apr. 1958 – Unknown major area • J.O. Ariwaodo ARS1184 (K), Munta Village. Sierra Leone – Western Area • J.K. Morton SL2013 (FHI, IFAN, K, SL, WAG); Western Area, near York by Whale Bay, Peninsula; 8°17'N, 13°11'W; 02 May. 1965.

**Figure 6.**
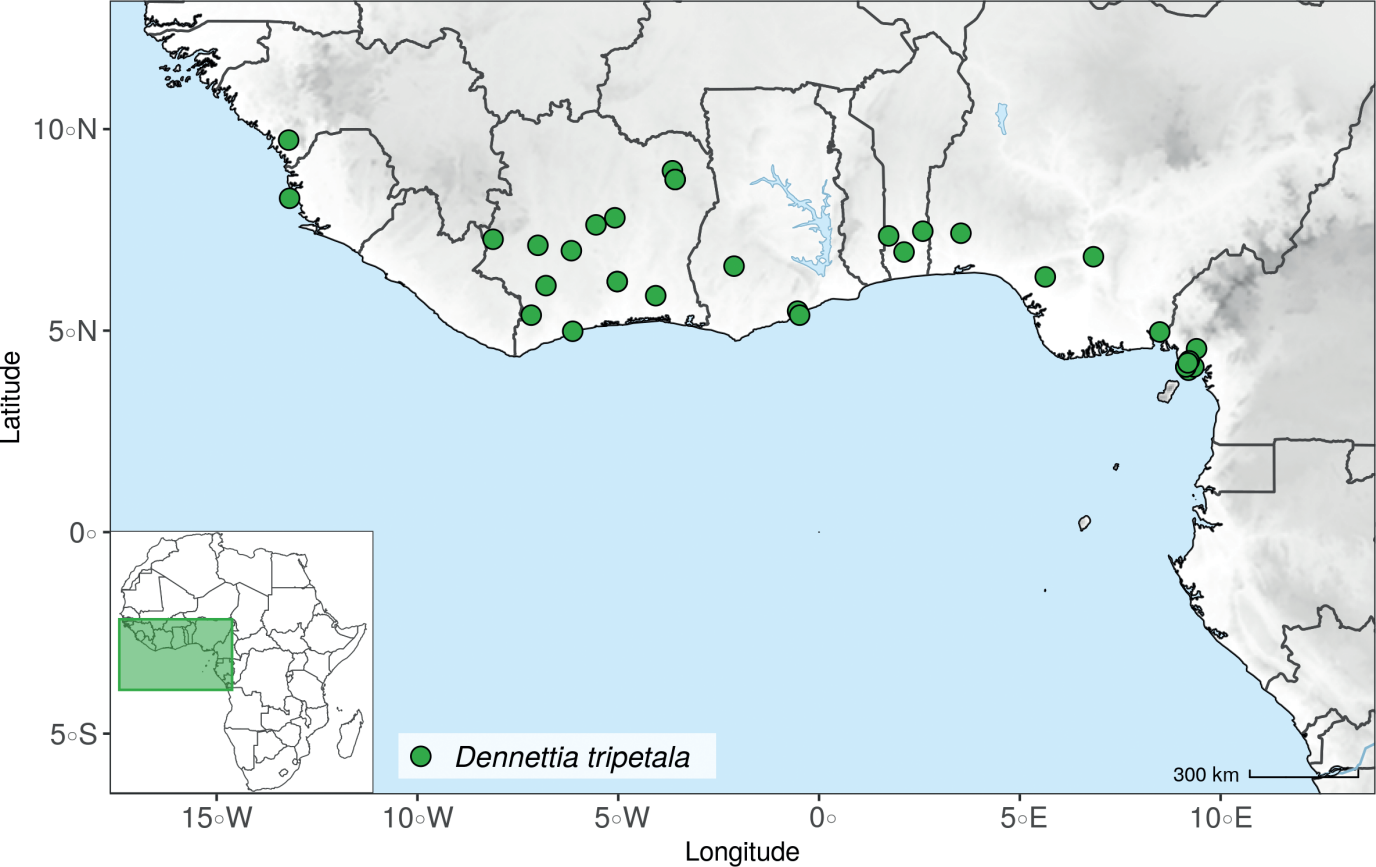
Distribution map of *Dennettiatripetala*. Shades of grey represent elevation, from white (sea level) to darker grey (higher elevation). The inset shows the extent of the map over Africa.

#### ﻿ Uvariodendron

Taxon classificationPlantaeMagnolialesAnnonaceae

(Engl. & Diels) R.E.Fr., Acta Horti Berg. 10: 51 (1930)

= Uvaria L. section Uvariodendron , Engl. & Diels in Engl. Afr. Pfl. VI 8 (1901).

##### Type species.

*Uvariodendronfuscum* (Benth.) R.E.Fr. (≡ *Uvariagigantea* Engl.).

##### Description.

Shrub to trees 2–30 m tall, D.B.H. 1–35 cm; young branches pubescent to tomentose to glabrous, old branches slightly tomentose to glabrous. Leaves with margin flat to slightly revolute. Petiole 2.5–35 mm long, 1–9 mm wide, pubescent to tomentose to glabrous. Leaf lamina 65–750 mm long, 20–250 mm wide, length:width ratio 2.1–6.2, elliptic to obovate to oblong, coriaceous, base acute to decurrent to rounded to subcordate, apex acute to acuminate, acumen 0.5–38 mm long; surface above glabrous, surface below pubescent to glabrous when young, glabrous when old; midrib impressed above, raised below, glabrous above, pubescent to glabrous below; secondary veins 8–41 pairs, brochidodromous to weakly brochidodromous, impressed above, raised below; tertiary veins reticulate. Inflorescences borne on trunk, on branches or axillary, composed of 1–11 sessile to pedicellate flowers. Flower pedicel 0–65 mm long, 1–9 mm in diameter, pubescent to glabrous. Flowers bisexual, buds globose to ovoid, sessile to pedicellate, 2–22 mm high, 4–30 mm in diameter, pubescent. Bracts 1 to 6, upper bract 1–35 mm long, 1–50 mm wide, depressed ovate to ovate, generally adpressed, enclosing the bud or semi-clasping the pedicel, pubescent outside, glabrous to pubescent inside. Sepals 3, 3–55 mm long, 2.5–43 mm wide, valvate or imbricate, free or fused at base, pubescent outside, glabrous inside, green to brown. Outer petals 3, 10–70 mm long, 9–47 mm wide, length:width ratio 0.9–2.5, ovate to elliptic, pubescent outside, puberulent to glabrous inside, cream to brown to wine red outside, cream with dark red at base to wine red inside. Inner petals 3, 10–53 mm long, 5–38 mm wide, length:width ratio 0.9–2.4, elliptic to obovate, pubescent outside, puberulent to glabrous inside, cream to brown to wine red outside, cream with dark red at base to wine red inside. Stamens 200 to 3000, 1–5 mm long, 0.1–0.7 mm wide, anthers linear, connective prolongation truncate. Carpels 5 to 160, 1–7 mm long, 0.5–2.2 mm wide, pubescent, free; stigma 0–2 mm long, 0.5–2 mm wide, coiled, pubescent, generally covered with an exsudate at anthesis. Fruiting pedicel 0–31 mm long, 2–8 mm in diameter, pubescent. Monocarps 1 to 80, 17–90 mm long, 4.5–32 mm wide, length:width ratio 1.3–11, generally cylindrical, pubescent to glabrous, green to orange to red to dark-blue black; sessile to stipitate; stipe 0–12 mm long, 1–10 mm wide, pubescent to glabrous. Seeds 1–18 per monocarp, uniseriate to biseriate, 3 to 29 mm long, 2–11 mm wide, ellipsoid to semicircular.

### ﻿Key to *Uvariodendron* species

**Table:** 

1	Largest leaves with lamina ≤ 350 mm long	**2**
–	Largest leaves with lamina > 350 mm long	**13**
2	Largest leaves with lamina < 160 mm long	**3**
–	Largest leaves with lamina > 160 mm long	**5**
3	Plant with strong bergamot scent (bark and crushed leaves), leaves stiff, between coriaceous and cartilaginous, apex acute to shortly acuminate; monocarps cylindrical and tomentose with regular tufts of high hair density (Tanzania)	***Ud.mbagoi***
–	Plant without bergamot scent, leaves coriaceous, apex attenuate; monocarps cylindrical and glabrous or ovoid and densely pubescent	**4**
4	Flower buds ca. 4 mm in diameter, covered by ca. 6 velutinous bracts 5–6 mm long; flowers with ca. 50–75 carpels; monocarps ovoid, densely pubescent, sessile (Kenya)	***Ud.dzomboense***
–	Flower buds ca. 6 mm in diameter, covered by 2–5 velutinous bracts ca. 4 mm long; flowers with ca. 5 carpels; monocarps cylindrical, glabrous, with stipe ca. 12 mm long (Mozambique)	***Ud.mossambicense***
5	Sepals fused at base over > 10% of their length	**6**
–	Sepals free or fused at base over ≤ 10% of their length, connivent or imbricate	**9**
6	Plant with a strong lemon scent; leaves narrowly elliptic, base acute to cuneate, flower sessile with petals 15–25 mm long and 5–15 mm wide (West and Central Africa)	***Ud.angustifolium***
–	Plants with no lemon scent; leaves elliptic to oblong to obovate, base acute to decurrent; flower pedicellate, or flower sessile with petals 20–36 mm long and 15–19 mm wide	**7**
7	Plant with bark reddish peeling off; flower bud globose to ovoid, sessile, > 6 mm high; flower with sepals ≥ 10 mm long and wide (Tanzania)	***Ud.pycnophyllum***
–	Plant with bark not reddish and not peeling off; flower bud globose, subsessile to pedicellate, ≤ 6 mm high; flower with sepals < 8 mm long	**8**
8	Leaves 170–350 mm long; flowers with sepals 3–5 mm long, outer petals elliptic 15–25 mm long, carpels 20 to 40; fruits with 6 to 11 stipitate (stipe 5–12 mm) monocarps (West Africa)	***Ud.occidentale***
–	Leaves 159–188 mm long, flowers with sepals 5.5–7 mm long, outer petals broadly obovate 11–12 mm long, carpels ca. 7; fruits with 3 to 5 sessile monocarps (Kenya)	***Ud.schmidtii***
9	Leaves obovate, base decurrent, apex acuminate; flowers with bract ca. 10 mm long, ca. 30 carpels (Gabon)	***Ud.pilosicarpum***
–	Leaves elliptic to oblong to obovate, base acute to decurrent or acute to rounded, apex acute to attenuate; flowers with bract ≤ 6 mm long, < 20 carpels	**10**
10	Plant with a strong aniseed smell; flowering pedicels 15–65 mm (Kenya, Tanzania)	***Ud.anisatum***
–	Plant with no aniseed smell; flowering pedicels ≤ 28 mm	**11**
11	Fresh leaves with strong lemon scent when crushed, leaves ≥ 250 mm (Central Africa)	***Ud.citriodorum***
–	Leaves with no lemon scent when crushed, leaves < 220 mm	**12**
12	Leaves 140–220 mm long with a midrib slightly raised above with a central groove all along; flower with imbricate sepals 6–12 mm long, and elliptic petals 16–39 mm long with a slight transversal curvature (Tanzania)	***Ud.kimbozaense***
–	Leaves 70–190 mm long with a midrib slightly impressed above; flower with connivent sepals 3–6.5 mm long, ovate petals 10–20 mm long with a strong transversal curvature (Kenya, Tanzania)	***Ud.kirkii***
13	Young branches, petioles, and midrib below the lamina covered with a brown tomentum, generally persisting on older branches (West and Central Africa)	***Ud.calophyllum***
–	Young branches, petioles, and midrib below the lamina pubescent to glabrous or covered with long soft hairs quickly falling off	**14**
14	Leaves with rounded to subcordate base (Tanzania)	***Ud.usambarense***
–	Leaves with acute to rounded base	**15**
15	Monocarps with l:w ratio ≥ 5, very narrowly cylindrical, torulose to torose (i.e. very strongly constricted between the seeds); seeds < 10 mm long (East Africa)	***Ud.gorgonis***
–	Monocarps with l:w ratio < 3.5, cylindrical, not constricted between the seeds; seeds > 10 mm long	**16**
16	Young branches glabrous; flowering pedicel ≥ 10 mm, petals wine red outside and inside; monocarps sparsely pubescent to glabrous (Central Africa)	***Ud.connivens***
–	Young branches pubescent to glabrous; flower pedicel ≤ 15 mm, petals wine red cream to light yellow outside, cream with dark red steak inside; monocarps pubescent to sparsely pubescent	**17**
17	Bracts 3–8 mm long and 3–10 mm wide; sepals free, sometimes fused at base, generally imbricate, 5–9 mm long and 5–10 mm wide (Central Africa)	***Ud.molundense***
–	Bracts 8–35 mm long and 10–50 mm wide; sepals fused at base over 20–50% of their length, 11–55 mm long and 13–43 mm wide	***Ud.fuscum* (18)**
18	Young branches sparsely pubescent to glabrous; sepals 11–23 mm long and 13–26 mm wide; carpels 20 to 70; fruiting pedicel ca. 5 mm long (Central Africa)	**Ud.fuscumvar.fuscum**
–	Young branches with long soft hairs producing a whitish appearance quickly falling off; sepals 20–55 mm long and 16–43 mm wide; carpels 50 to 160; fruiting pedicel ≥ 9 mm long	**19**
19	Flowering pedicels 0–7.5 mm long, sepals 20–30 mm long and 16–26 mm wide, petals 21–40 mm long and 17–29 mm wide, carpels 50 to 100 (Central Africa)	**Ud.fuscumvar.giganteum**
–	Flowering pedicels 10–15 mm long, sepals 30–55 mm long and 30–43 mm wide, petals 50–70 mm long and 36–47 mm wide, carpels 150 to 160 (Uganda)	**Ud.fuscumvar.magnificum**

### ﻿Species descriptions

#### ﻿ Uvariodendron angustifolium

Taxon classificationPlantaeMagnolialesAnnonaceae

(Engl. & Diels) R.E.Fr., Acta Horti Berg. 10: 58 (1930)

Figs 7E–J , 8

≡ Uvaria angustifolia Engl. & Diels, Notizbl. Königl. Bot. Gart. Berlin 2: 295 (1899); Uvaangustifolia (Engler & Diels) Kuntze, Deutsche Bot. Monatsschr. xxi. 173 (1903). Type. Cameroon – South-West Region • A. Staudt 742a (holotype: B! (B 10 0153115)), Johann Albrechts-Höhe; 4°10'N, 9°12'E; alt. 400 m; 20 Mar. 1896.

##### Description.

Shrub to tree 3–12 m tall, D.B.H. unknown; young branches sparsely pubescent to glabrous, old branches glabrous; plant with strong aromatic smell. Leaves with strong lemon smell when crushed. Petiole 3–7.5 mm long, 1–2 mm wide, pubescent to glabrous. Leaf lamina 100–199 mm long, 30–58 mm wide, length:width ratio 3–4, narrowly elliptic, coriaceous, base acute to cuneate, apex acute to broadly acuminate, acumen 11–14 mm long; surface above glabrous, surface below pubescent to glabrous when young, pubescent at base to glabrous when old; midrib slightly impressed above, raised below, glabrous above, pubescent to glabrous below; secondary veins 8–14 pairs, weakly brochidodromous, slightly impressed above, raised below; tertiary veins reticulate. Inflorescences borne on trunk and branches, composed of 1 (sub)sessile flower. Flower pedicel 0–6 mm long, 2–3 mm in diameter, velutinous. Flowers bisexual, buds globose, sessile to shortly pedicellate, 5–8 mm high, 6–8 mm in diameter, sericeous. Bracts 1 to 6, upper bract 6–11 mm long, 9–15 mm wide, ovate, appressed, enclosing the bud, sericeous outside, glabrous inside. Sepals 3, 9–13 mm long, 9–13 mm wide, fused at base, slightly imbricate at mid-length, sericeous outside, glabrous inside, color unknown. Outer petals 3, 15–25 mm long, 9–15 mm wide, length:width ratio 1.13–1.78, elliptic to oblong, velutinous outside, glabrous inside, yellowish brown outside, crimson with cream margins inside. Inner petals 3, 15–22 mm long, 5–10 mm wide, length:width ratio 1.9–3, obovate, velutinous outside, glabrous inside, yellowish brown outside, crimson with cream margins inside. Stamens 200 to 300, 3–3.5 mm long, 0.1–0.5 mm wide, anthers linear, connective prolongation truncate. Carpels 7 to 30, 3–4.5 mm long, 1–1.5 mm wide, pubescent, free; stigma 1–2 mm long, 0.8–1.5 mm wide, coiled, pubescent, covered with an exudate at anthesis. Fruiting pedicel ca. 5 mm long, 3.5 mm in diameter, pubescent. Monocarps 2 to 10, 23–40 mm long, 17–30 mm wide, length:width ratio ca 1.3, ellipsoid, smooth, sparsely pubescent to glabrous, yellow, with strong lemon smell; shortly stipitate, stipe 1–3.5 mm long, 1–2.5 mm wide, pubescent. Seeds 9 to 18 per monocarp, biseriate, 21 to 28 mm long, ca. 10 mm wide, 2–4 mm thick, semicircular.

**Figure 7.**
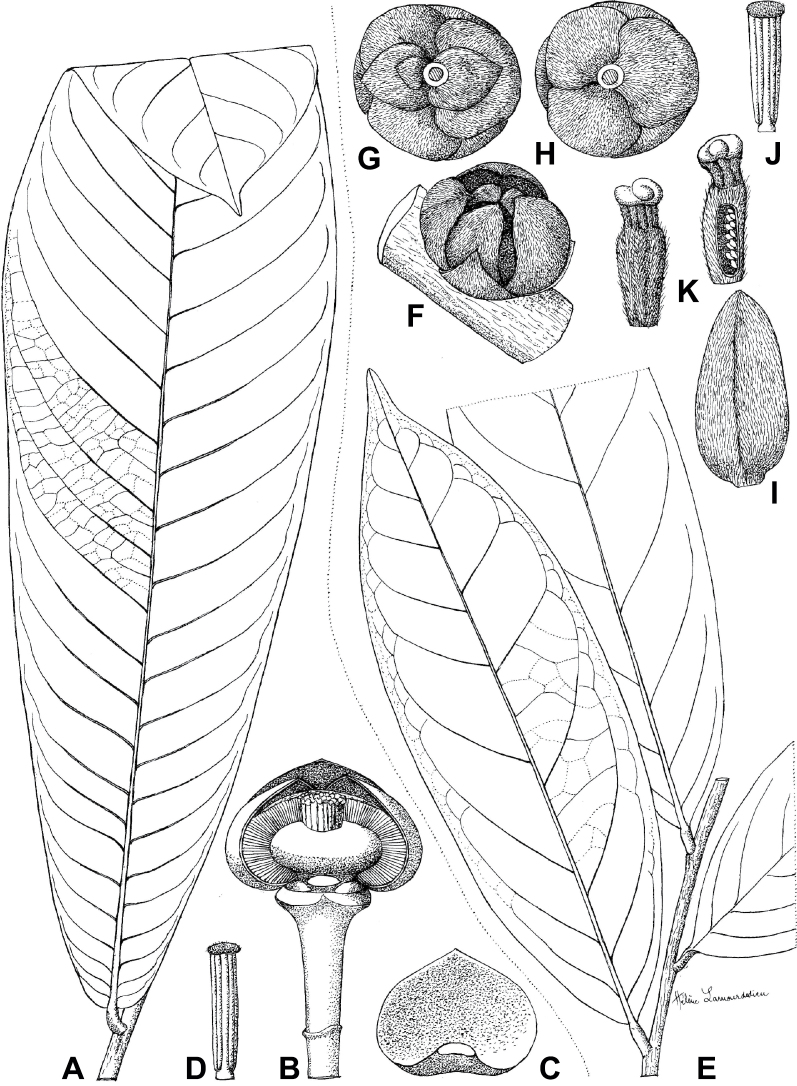
*Uvariodendronconnivens* (Benth.) R.E.Fr. **A** leaf **B** flower, two outer and one inner petal removed **C** outer petal inner view **D** stamen, front view. *Uvariodendronangustifolium* (Engl. & Diels) R.E.Fr. **E** leaves **F** flower, semi top view **G** flower, bottom view showing bracts **H** flower, bottom view, bracts removed **I** outer petal, outer view **J** stamen, front view **K** carpel, front view and detail of ovules. **A–D** from Mann 1159 **A–K** from Vigne 1610. Drawings by Hélène Lamourdedieu, Publications Scientifiques du Muséum national d’Histoire naturelle, Paris.

##### Distribution.

Endemic to Upper Guinean Domain and Lower Guinean Domain of the Guineo-Congolian Region: Cameroon, Ghana, Ivory Coast, Nigeria. This species has also been reported in Benin (Bada Amouzoun et al. 2019), but no herbarium specimen was associated with this study. Its presence in Benin can thus not be confirmed.

##### Habitat and ecology.

Lowland mature or secondary rain forests. Altitude: 152–400 m a.s.l.

##### Phenology.

Flowers collected from February to March and November to December. Fruits collected from January to May.

##### Vernacular names.

Ghana: ‘Esuno Kodu’ or ‘Bomborgo Kodu’ in Ashanti (Vigne 1610).

##### Uses.

The fruits are used to flavour the soup (Jones FHI 3480).

##### Notes.

This species can be easily distinguished from the other *Uvariodendron* species by its narrowly elliptic leaves. It also emits a strong citrus scent when fresh, a characteristic shared with other Annonaceae species: *Uvariodendroncitriodorum* (Le Thomas) Dagallier & Couvreur and *Uvariopsiscitrata* Couvreur & Niangadouma.

##### Preliminary conservation status.

This species is distributed from Cameroon to Ivory Coast. Its EOO is estimated at 186,201 km^2^ and its AOO at 52 km^2^. It occurs in ca. 10 locations, but the last collection dates back more than 20 years and two thirds of the collections are older than 50 years, suggesting a decline in the number of locations and in AOO. We thus assign a preliminary conservation status of Vulnerable VU under criterion B2ab(ii,iv).

**Figure 8.**
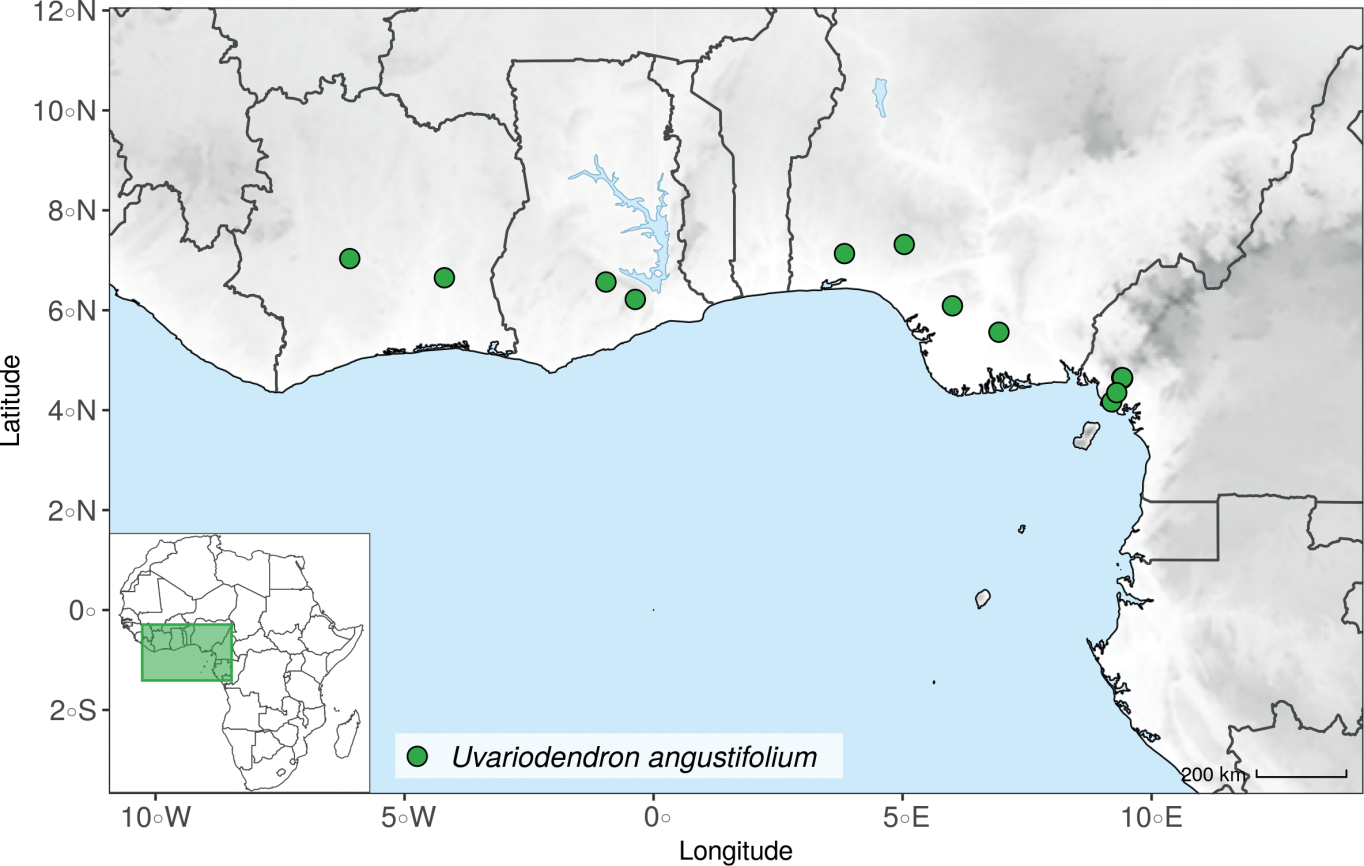
Distribution map of *Uvariodendronangustifolium*. Shades of grey represent elevation, from white (sea level) to darker grey (higher elevation). The inset shows the extent of the map over Africa.

##### Additional specimens examined.

Cameroon – Central Region • R.W.J. Keay FHI37524 (FHI, K), between Bafia and Likoko; 4°21'N, 9°18'E; 05 Feb. 1958 – South-West Region • D.W. Thomas 6087 (MO, YA); Meme, Vicinity of Lake Barombi, Kumba; 4°39'N, 9°24'E; alt. 300 m; Apr. 1986 • D.W. Thomas 7018 (YA), Cocoa farms along the road between Konye and Bakole; 4°39'N, 9°25'E; alt. 300 m; 25 May. 1987 – Unknown major area • A. Staudt 642 (BM, G, K), Kumba; 4°38'N, 9°25'E; 1896. Ghana – Ashanti Region • C. Vigne 1610 (K, P), Pra River, ashanti; 6°33'54.36"N, 0°57'21.6"E; alt. 152 m; Feb. 1929 – Western Region • N.K. Lovi 3964 (K, P), New Tafo E.P; 6°13'N, 0°22'E; 03 Dec. 1954. Ivory Coast – Bouaflé • C.C.H. Jongkind 4368 (IAGB, MO, WAG), Parc National de la Marahoue; 7°02'N, 6°06'W; alt. 270 m; 13 Feb. 1998 – Daoukro • A.J.B. Chevalier s.n (P), Morénou: environs du poste de Bangouanou; 6°39'N, 4°12'W; 26 Nov. 1909. Nigeria – Edo State • B.O. Daramola 94/337 (K, MO), Iyekoriomwon, Edo State, about 35 kilometers from Uyo (Ugo ?); 6°05'19.05"N, 6°00'04.46"E; 09 Jan. 1994 – Ondo State • A.P.D. Jones FHI3480 (K); Owo District, Owo forest reserve about 4 miles North of Igbatoro crossing; 5°33'36.36"N, 6°56'06"E; 19 Apr. 1943 • F. Anakwense FHI19702 (K); Akure, Akure Forest Reserve; 7°19'N, 5°02'E; 11 Dec. 1950 – Oyo State • D. Gledhill 817 (K); Ibadan District, Gambari Forest Reserve; 7°08'N, 3°50'E; 02 Feb. 1968.

#### ﻿ Uvariodendron anisatum

Taxon classificationPlantaeMagnolialesAnnonaceae

Verdc., Kew Bull. 10(4):596 (1956)

Figs 9 , 10

##### Type.

Kenya – Nairobi • B. Verdcourt 526 (holotype: EA! (EA000002461); isotypes: K! (K000198890, K000198891), PRE); Nairobi District, in Karura Forest and Thika Gorge; 1°14'S, 36°49'E; 24 Jun. 1951.

##### Description.

Shrub to tree 3.5–15 (22) m tall, D.B.H. up to 10 cm; young branches sparsely pubescent to glabrous, old branches glabrous; plant with aniseed smell. Leaves reddish pink when young, dark green when old. Petiole 3–9 mm long, 1.5–4 mm wide, sparsely pubescent to glabrous. Leaf lamina 80–313 mm long, 45–187 mm wide, length:width ratio 2.3–3.1, elliptic to obovate, coriaceous, base acute to decurrent, apex acute to attenuate, surface above glabrous, surface below sparsely pubescent to glabrate when young, glabrous when old; midrib impressed above, raised below, glabrous above, sparsely pubescent to glabrous below; secondary veins 12–21 pairs, weakly brochidodromous, impressed above, raised below; tertiary veins reticulate. Inflorescences clustered on the trunk or borne on branches, composed of 1–3 flowers. Flower pedicel 15–65 mm long, 1–2 mm in diameter, pubescent. Flowers bisexual, buds globose, pedicellate, 3.5–6 mm high, 4.5–6 mm in diameter, sericeous. Bracts 1 to 2 at base and 1 towards the upper half of the pedicel, upper bract 2–4.5 mm long, 3–8 mm wide, clasping the pedicel, velutinous outside, glabrous inside. Sepals 3, 3.5–6 mm long, 4–8 mm wide, valvate to fused at base over 10 % of their length, velutinous outside, glabrous inside, color unknown. Outer petals 3, 10–23 mm long, 9–16 mm wide, length:width ratio 1–1.6, broadly ovate to ovate, velutinous outside, glabrous inside, cream outside, cream inside. Inner petals 3, 12.5–15 mm long, 5–9 mm wide, length:width ratio 1.5–2, obovate, shortly velutinous outside, glabrous inside, cream outside, cream inside. Stamens 400 to 500, 1.5–2.5 mm long, 0.1–0.5 mm wide, anthers linear, connective prolongation truncate. Carpels 7 to 12, 2–5 mm long, 1–1.5 mm wide, pubescent, free; stigma 1–2 mm long, 1–1.5 mm wide, coiled, pubescent. Fruiting pedicel 21–59 mm long, 2–3 mm in diameter, sparsely pubescent to glabrous. Monocarps 3 to 14, 38–70 mm long, 13–20 mm wide, length:width ratio 2.3–3.5, cylindrical, longitudinally ridged, slightly constricted between the seeds, slightly acuminate, sparsely pubescent, dark olive green to dark-blue black; sessile to shortly stipitate, stipe 0–2 mm long, 3–4.5 mm wide, sparsely pubescent. Seeds 8–18 per monocarp, biseriate, 10 to 12 mm long, 5–8 mm wide, ellipsoid to semicircular.

**Figure 9.**
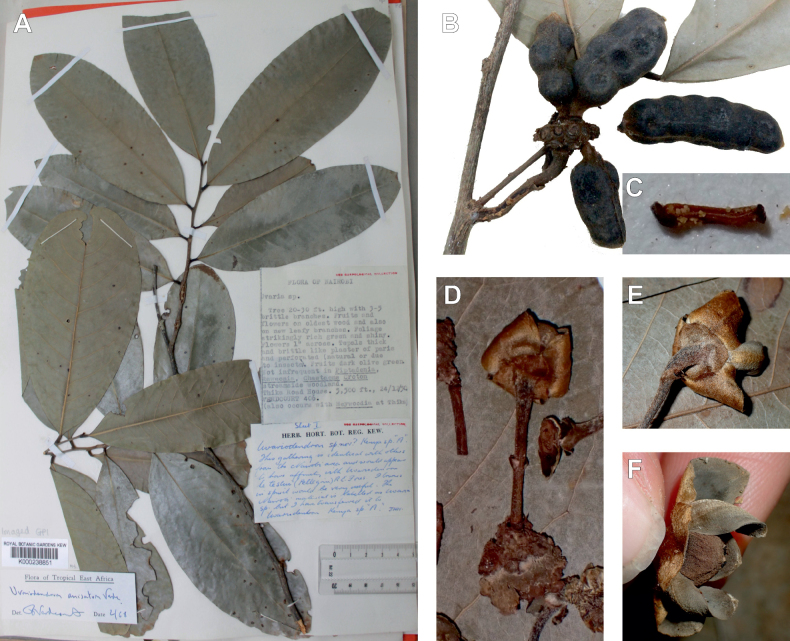
*Uvariodendronanisatum* Verdc **A** entire specimen sheet with young branch and leaves **B** fruit with four monocarps (one detached) **C** stamen, side view **D** flower and pedicel attached to bark clump, semi-bottom view **E** detail of flower, bottom view **F** detail of flower showing sepals, one outer petal removed, semi-top view. **A** Verdcourt 406 **B** Verdcourt 674 **D–F** Faden 74/886. Photos Léo-Paul Dagallier.

##### Distribution.

Endemic to Somalia-Masai Region: Kenya and Tanzania.

##### Habitat and ecology.

Submontane rain forests (sometimes dry forests), often along streams. Altitude: 300–1080 m a.s.l.

##### Phenology.

Flowers collected in January, March, from June to July and from November to December. Fruits collected in January, March, from May to July and from November to December.

**Figure 10.**
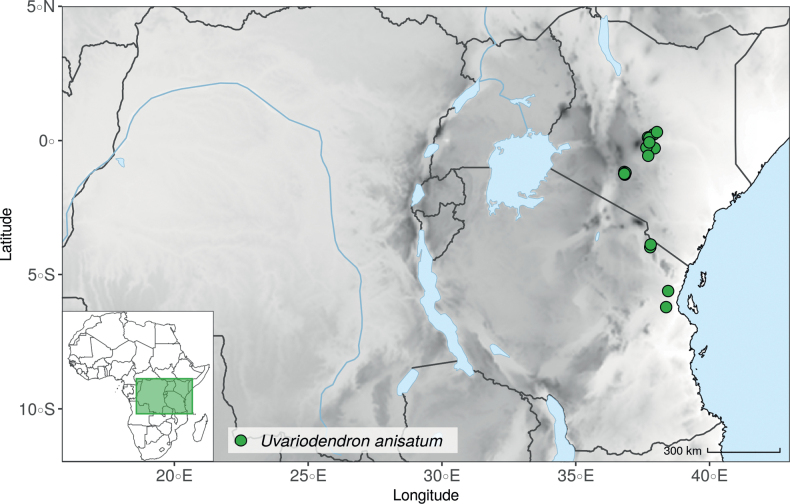
Distribution map of *Uvariodendronanisatum*. Shades of grey represent elevation, from white (sea level) to darker grey (higher elevation). The inset shows the extent of the map over Africa.

##### Vernacular names.

Kenya: ‘Mutongu’ in Meru (Parnell 13340).

##### Notes.

This species is characterized by a strong aniseed smell (lightly to not noticeable on herbarium specimens), a character unique in the Annonaceae family to my knowledge. Apart from the smell, it closely resembles *Uvariodendronkirkii* and can hardly be differentiated on dry sterile material. In flowers, it can be differentiated from *Ud.kirkii* by its flower pedicel generally longer (15–65 mm, vs. 5–28 mm), its relatively flat petals with a slight transversal curvature (vs. “boat-shaped” petals, i.e. with a strong transversal curvature). Note that the petals curvature is not easy to rely on dry specimen. In fruits, it can be differentiated with a greater fruit pedicel than *Ud.kirkii* (21–59 mm long, vs. 7–22 mm long). Verdcourt also used the monocarps to differentiate the 2 species, the monocarps of *Ud.anisatum* being dark blue and with a slight longitudinal ridge, and the monocarps of *Ud.kirkii* being dull-orange and without any pseudosuture (Verdcourt 1969). However, these 2 characters are no longer valid for a clear differentiation of the 2 species as the specimen Robertson MDE 293, which is *Ud.kirkii* (no aniseed smell reported, flower pedicel length of ca. 11 mm), has dark blue grey fruits, and several specimens of *Ud.kirkii* (e.g. Drummond 3983; Gilbert 4967; Greenway 2681) present a slight longitudinal ridge.

##### Conservation status.

This species has been assessed as Vulnerable VU under criteria B2ab(ii,iii,iv,v) (Luke et al. 2018).

##### Additional specimens examined.

Kenya – Central • E. Battiscombe 1285 (EA, K); Kiambu District, Kiambu Forest; 1°10'S, 36°49'E; alt. 1700 m; 17 Jun. 1924 • J.L. Moon 811 (K); Kiambu District, Kyambu; 1°11'48.48"S, 36°51'32.76"E; alt. 1737 m; 29 Jul. 1913 – Eastern • H.J. Beentje 4089 (EA); Meru, Kijegge Hill; 0°17'N, 37°57'E; alt. 1400 m; 08 Mar. 1989 • I. Malombe 1368 (EA), Ngaia Forest, Kiegoi village, NE border; 0°14'N, 37°55'E; alt. 1170 m; 22 May. 2008 • L.E. Parnell EA13340 (K); Meru, Lower Imenti Foest Reserve; 0°07'14.88"N, 37°41'52.08"E; 24 Aug. 1965 • M.A. Brunt 1546 (K), Embu Meru road; 0°15'43.26"N, 37°38'23.43"E; 16 Feb. 1964 • R.B. Faden 70/120 (EA, K); Meru District, Lower Imenti Forest, 6 mils. from Meru along Meru – Mikinduri Road; 0°06'N, 37°45'E; alt. 1190 m; 01 Mar. 1970 • S.A.L. Smith 287 (K); Embu District, Kiangombe, northern slopes; 0°33'52.56"N, 37°42'27.72"E; alt. 1550 m; 30 Nov. 2000 • W.R.Q. Luke 7123 (EA); Meru, Ngaia Forest, camp 142; 0°19'N, 38°02'E; alt. 1080 m; 22 Nov. 2000 – K4 Central • H.J. Beentje 1830 (EA, WAG), Nairobi, E. Karura forest near Kiambu R; 1°14'30"S, 36°49'30"E; alt. 1600 m; 13 Jan. 1985 • R.B. Faden 74/886 (K, WAG), Meru District. Lower Imenti Forest, on Meru-Mikinduri Road; 0°04'N, 37°45'E; alt. 1190 m; 26 Jun. 1974 – Nairobi • B. Verdcourt 3689 (K); Nairobi District, Karura Forest, by Ruaraka R. to W. of main Thika highway; 1°14'S, 36°49'E; 21 Jul. 1963 • B. Verdcourt 406 (B, K, K); Nairobi District, Thika Road House; 1°15'59.57"S, 36°50'04.08"E; alt. 1676 m; 24 Dec. 1950 • B. Verdcourt 674 (K); Nairobi District, Karura Forest; 1°14'S, 36°49'E; 29 Jun. 1952 • E. Polhill 324 (K); Nairobi District, Karura Forest; 1°14'S, 36°49'E; 10 Jan. 1961 • V.G.L. van Someren 95 (G, K); Nairobi District, Karura Forest; 1°14'S, 36°49'E; 13 Mar. 1940. Tanzania – Kilimanjaro • K.B. Vollesen 96/15 (K), Mkomazi Game Reserve, Ibaya Hill; 3°59'S, 37°47'E; alt. 1350 m; 03 Jun. 1996 • R.A. Abdallah 96/86 (K), Mkomazi National Park, Mkomazi Game Reserve, Maji Kununua Ridge; 3°53'S, 37°48'E; alt. 1500 m; 07 Jun. 1996 – Pwani • R.P. Sacleux 212 (P), Mandéra (Zanguebar); 6°12'51.22"S, 38°22'37.73"E; Jan. 1889 – Tanga • R.C. Wingfield 2880 (K), Msangasi Forest Reserve, 50 km S of Korogwe; 5°36'55.14"S, 38°27'06.26"E; alt. 300 m; 31 Mar. 1975.

#### ﻿ Uvariodendron calophyllum

Taxon classificationPlantaeMagnolialesAnnonaceae

R.E.Fr., Acta Horti Berg. 10: 63 (1930)

Figs 11 , 12

= Uvaria gigantea Engl., Notizbl. Königl. Bot. Gart. Berlin 2: 292. (1899) (quoad specimen Zenker 1738).

##### Type.

Cameroon – South Region • G.A. Zenker 2344 (holotype: B! (B100153116); isotypes: BM! (BM000636669), E!(E00704856), G! (G00412241), GOET! (GOET005732), HBG! (HBG502513), K! (K000198796, K000198797), M! (M0107940), P! (P00362658, P00362659, P00362661), S! (S07-13393, S07-13396), WAG! (WAG.1418666)), Bipindi; 3°05'N, 10°25'E; 1901.

##### Description.

Tree 2–20 m tall, D.B.H. 1–32.1 cm; young branches tomentose, old branches slightly tomentose to glabrous. Leaves reddish when young, dark green when old. Petiole 4–25 mm long, 2–9 mm wide, tomentose. Leaf lamina 258–765 mm long, 61–248 mm wide, length:width ratio 2.2–4.9, obovate, coriaceous, base rounded to slightly truncate, apex acuminate, acumen 6–32 mm long; surface above glabrous, surface below pubescent to glabrous; midrib impressed above, raised below, glabrous above, tomentose below; secondary veins 20–41 pairs, brochidodromous to weakly brochidodromous, impressed above, raised below; tertiary veins reticulate. Inflorescences borne on trunk and branches, composed of 2–3 (sub)sessile flowers. Flower pedicel 0–11 mm long, 4–9 mm in diameter, tomentose. Flowers bisexual, buds globose, sessile, 7–17 mm high, 7–30 mm in diameter, tomentose. Bracts 1 to 6, upper bract 10–23 mm long, 10–40 mm wide, ovate, appressed, enclosing the bud, tomentose outside, glabrous inside. Sepals 3, 10–27 mm long, 10–26 mm wide, imbricate to fused at base, tomentulose outside, glabrous inside, dark brown outside, reddish inside. Outer petals 3, 15–46 mm long, 10–33 mm wide, length:width ratio 0.9–1.5, broadly elliptic to elliptic, tomentulose outside, glabrous inside, cream outside, cream with dark red at base inside. Inner petals 3, 14–41 mm long, 11–32 mm wide, length:width ratio 1.2–1.9, obovate, shortly tomentulose outside, glabrous inside, cream outside, dark red with cream margins inside. Stamens around 3000, 3.5–4.9 mm long, 0.1–0.5 mm wide, anthers linear, connective prolongation truncate. Carpels 35 to ca. 150, 2–5 mm long, 0.5–2 mm wide, pubescent, free; stigma 1–2 mm long, 0.5–2 mm wide, coiled, velutinous, covered with an exudate at anthesis. Fruiting pedicel 1–7 mm long, 4–7 mm in diameter, densely pubescent. Monocarps 3 to 35, 27–55 mm long, 9–25 mm wide, length:width ratio 1.8–3.7, ellipsoid to obovoid, tomentose, brown; sessile to shortly stipitate, stipe 0–10 mm long, 1.5–3 mm wide, tomentose. Seeds 7–13 per monocarp, biseriate, ca. 13 mm long, 8–9 mm wide, semicircular.

**Figure 11.**
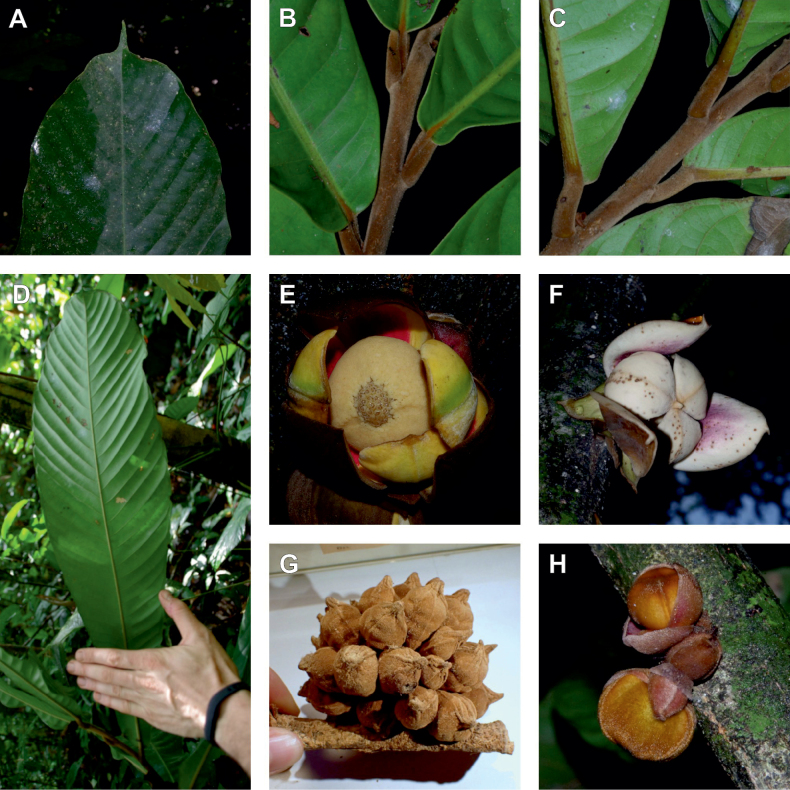
*Uvariodendroncalophyllum* R.E.Fr. **A** leaf apex upper side **B** detail of young branch and leaf base, upper side, note the brown tomentum **C** detail of young branch and leaf base, lower side **D** leaf **E** flower fully open, top view **F** flower with inner petals still attached to each other, semi-top view **G** fruit with many monocarps (dried) **H** flower buds, note the brown tomentum. **A, D, H** Couvreur 1157 **B, C** Couvreur 999 **E, F** Couvreur 1013 **G** Letouzey 14020. Photos **A–F, H** Thomas Couvreur, **G** Léo-Paul Dagallier.

##### Distribution.

Element of the Upper Guinean Domain and Lower Guinean Domain of the Guineo-Congolian Region: Cameroon, Gabon, Ghana, Ivory Coast, Nigeria.

##### Habitat and ecology.

Lowland mature or old secondary rain forests, near streams. Altitude: 30–140 m a.s.l.

##### Phenology.

Flowers collected from December to May. Fruits collected from March to May, from July to August, and in November.

##### Vernacular names.

Cameroon: ‘Ebom Bulu’ (Parren 223) or ‘Ebom Afame’ (Parren 68) in Bulu/Ewondo.

##### Notes.

This species is close to *Uvariodendronconnivens* and Ud.fuscumvar.giganteum by having large obovate leaves (more than 25 cm long). However, it can easily be distinguished from these three other species as it presents a tomentum of short dense brown matted hairs on the young parts (branches, petioles and lower midrib), producing a brown appearance with whitish reflections. The flower buds and outer petals are also covered outside with a similar brown and dense tomentum. The older parts of the plant are generally glabrous but can also present remnants of the tomentum.

**Figure 12.**
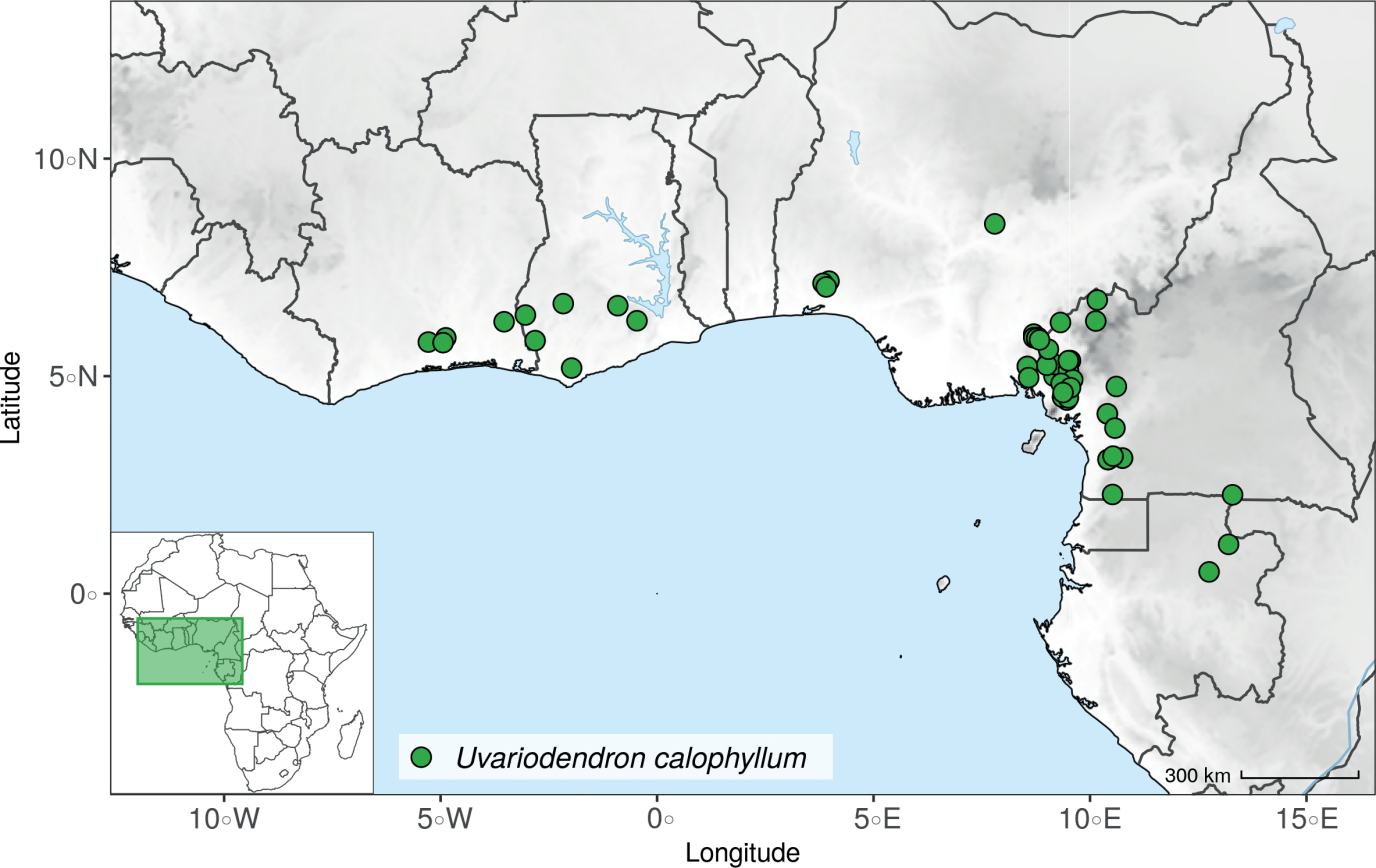
Distribution map of *Uvariodendroncalophyllum*. Shades of grey represent elevation, from white (sea level) to darker grey (higher elevation). The inset shows the extent of the map over Africa.

##### Conservation status.

This species is widespread, distributed from Ivory Coast to Gabon. It has been previously assessed as Least Concern LC (Cosiaux et al. 2019a).

##### Additional specimens examined.

Cameroon – Central Region • J.N. Asonganyi 421 (P, YA), Ndikiniméki, at Sonossi 26 km W. of Ndikinimeki. Map 1/200 000; 4°45'44.24"N, 10°36'29.26"E; 29 Mar. 1982 – Littoral • A.J.M. Leeuwenberg 5282 (BR, K, MO, P, PRE, WAG), 8 km W of Masok; 4°08'N, 10°24'E; alt. 400 m; 31 Mar. 1965 • A.J.M. Leeuwenberg 5282a (K, P, WAG), 8 km W. of Masok; 4°08'N, 10°24'E; alt. 400 m; 31 Mar. 1965 • R.G. Letouzey 12352 (P, YA), colline entre Tcherikoy et Sokelle II(30 km NW Eseka) – feuille IGN 1/200 000 EDEA; 3°48'18.03"N, 10°34'32.35"E; 14 Dec. 1973 – North-West Region • E.U. Ujor FHI29281 (FHI, K); Mentchum, Nkom-Wum, Bamenda Prov., Wum Distr., Nkom • Wum F.R. on German old road leading to Timber Camp by the left; 6°16'N, 10°08'E; 03 Jul. 1951 • R.G. Letouzey 14020 (P, WAG, YA), Piste Baji-Tumbo, entre Baji et rivière Chonogbonbon, 55 km NNE Wum; 6°45'N, 10°10'E; 12 Jul. 1975 – South Region • G.A. Zenker 1738 (B (B101178582), BM (BM000636667), COI (COI00004925), E (E00718583), G (G00412236), HBG (HBG502512), K (no barcode), LE (LE00012448), LECB (LECB0000053), M (M0239941), P (P06901474, P06901466, P06901467), WAG (WAG.1418665), WU (WU0025882, WU0025883), Z (Z-000056111, Z-000056112)), Bipindi; 3°05'N, 10°25'E; 1898 • G.A. Zenker s.n (P), Bipinde, Bipindi; 3°05'N, 10°25'E; Apr. 1903 • M.P.E. Parren 223 (KRIBI, WAG), About 7 km NE of Ebom. Plot 13, subplot 84, tree 6, coordinates: X = 7, Y = 3 m; 3°07'N, 10°45'E; alt. 500 m; Aug. 1996 • M.P.E. Parren 68 (KRIBI, WAG), About 7 km NE of Ebom. Plot 4, subplot 53, tree 9, coordinates: X = 7.5, Y = 8.5 m; 3°07'N, 10°45'E; alt. 500 m; Aug. 1996 • R.G. Letouzey 15272 (P, YA), Piste Meyo Ntem-Evouzok, 75 Km W Ambam, entre 1er et 3e bras du Ntem; 2°16'48"N, 10°31'12"E; 28 Nov. 1979 • T.L.P. Couvreur 1157 (K, MPU, P, WAG, YA), on road Lolodorf-Bipindi, about half way, near Mbiguiligui village (Mbikiliki); 3°09'41.51"N, 10°31'52.18"E; alt. 250 m; 26 Feb. 2018 • T.L.P. Couvreur 486 (YA), 15 km east from Lélé village; 2°16'17.2"N, 13°17'30.35"E; alt. 578 m; 09 Sep. 2013 – South-West Region • A. Binuyo FHI35564 (FHI, K, WAG), Kumba Distr. Along the path from Pete to Bopo at the right handside of the road in Southern Bakundu Forest Reserve; 4°31'N, 9°22'E; 23 Feb. 1956 • A.H. Gentry 62407 (MO), Banyong, Batanga area, between Awong and Banyu, ca 15 km W of Manyemen. TRANSECT 2; 5°00'N, 9°10'E; alt. 420 m; 02 May. 1988 • B. Sonké 5610 (MO); Meme, Nguti, Sanctuaire Mbanyang Mbo; 5°21'24"N, 9°32'57"E; alt. 239 m; 14 Apr. 2011 • C.F.A. Onochie FHI30860 (FHI, K), S.Bakundu, S. Bakundu F.R., between Bombe Rest House and Mbalange; 4°27'N, 9°28'E; 19 Mar. 1953 • D.W. Thomas 3322 (K, MO, P, YA), North-eastern corner of Korup National Park; near Baro village; 5°16'N, 9°11'E; alt. 200 m; 24 Mar. 1984 • D.W. Thomas 4549 (MO, YA), Takamanda Forest Reserve; 6°14'N, 9°19'E; alt. 170 m; 21 Mar. 1985 • D.W. Thomas 5965 (K, MO, WAG, YA), 30 km W of Kumba on Mbonge Road; 4°31'N, 9°22'E; alt. 50 m; 26 Mar. 1986 • D.W. Thomas 6090 (P, YA); Meme, from the vicinity of Lake Barombi, Kumba; 4°39'N, 9°24'E; alt. 300 m; Apr. 1986 • D.W. Thomas 7499 (MO, P, WAG, YA); Ndian, Between Baro and Ikenge villages, along foot path, in the Korup National Park; 5°15'N, 9°00'E; alt. 250 m; 01 Apr. 1988 • G.K. Gottsberger 210307/12 (ULM, WAG), c. 20 m from Banyang Mbo Research Station; 5°08'N, 9°30'E; 21 Mar. 2007 • J. Dundas FHI13898 (K), Southern Bakundu Forest Reserve; 4°30'N, 9°30'E; 19 Feb. 1946 • J. Nemba 64 (K, MO, P, WAG, YA, YA), Bolo Forest, 5 kms W of Kumba – Mamfe road near Konye; 4°55'N, 9°36'E; alt. 300 m; 25 Mar. 1986 • J. Olorunfemi FHI30561 (FHI, K), Mungo River F.R., Kumba Distr.: Mumbo – Southern Bakossi; 4°50'N, 9°20'E; 09 May. 1951 • M.R. Cheek 9337 (K, YA), Mungo River F.R., Mungo river forest reserve. c. 1 Km East of bridge, Chained road to S; 4°44'N, 9°33'E; alt. 200 m; 24 Oct. 1998 • R.G. Letouzey 13673 (P, YA), entre Babong et Okurikang, 35 km WSW. Mamfe (feuille IGN 1/200 000 Mamfe); 5°37'02.16"N, 9°02'22.7"E; 29 May. 1994 • T.L.P. Couvreur 1013 (MPU, WAG, YA), Bayang Mbo Wildlife Sanctuary, after Mbu river; 5°21'19.19"N, 9°30'01.69"E; alt. 242 m; 26 Mar. 2016 • T.L.P. Couvreur 980 (WAG, YA), on top of hill, near Small Ekombe village, 3 km after Kumba on road to Ekondo Titi town; 4°37'22.48"N, 9°22'37.12"E; alt. 615 m; 13 Jan. 2016 • T.L.P. Couvreur 999 (MPU, WAG, YA), Bayang Mbo Wildlife Sanctuary, after Mbu river; 5°21'04.06"N, 9°30'01.78"E; alt. 251 m; 25 Mar. 2016. Gabon – Ogooué-Ivindo • J. Florence 1005 (P), Station d'Ipassa, 10 km S de Makokou; 0°30'N, 12°45'E; alt. 500 m; 17 Apr. 1978 • N. Hallé 548 (P), Bélinga, mines de fer; 1°08'N, 13°12'E; alt. 700 m; 16 Aug. 1966. Ghana – Ashanti Region • C. Vigne 1611 (K, P), Kwahu Prasu; 6°37'10.2"N, 0°54'27.72"E; Feb. 1929 • H. Abbin GC43343 (K), Tano Ofin Forest Reserve, Tano Ofni F.R; 6°40'N, 2°10'W; 16 Aug. 1972 – Eastern Region • F.R. Irvine 3016 (K), Bunsu; 6°16'21.42"N, 0°27'52.93"E; May. 1938 – Western Region • A.A. Enti FE-2169 (B), Neung Forest Reserve, Takoradi-Tarkwa Road; 5°11'07.08"N, 1°58'23.52"W; May. 1982 • A.A. Enti FH6705 (K, P, WAG), Enchi Distr., Enchi – Nyankamam; 5°49'N, 2°49'W; May. 1957 • M.C. Merello 1385 (MO), Bia National Park, Bia National Forest and Production Reserve. Secondary logging roads west from MIM Timber Company Camp; 6°24'15"N, 3°02'30"W; alt. 140 m; 04 Mar. 1996. Ivory Coast – Adzopé • L. Aké Assi 11501 (G), Forêt d'Abongoua; 6°15'N, 3°32'W; 19 Mar. 1971 – Divo • C. Chatelain 679 (G), Forêt de l'IRCC de Divo; 5°47'N, 5°17'W; 19 Dec. 1990 • L. Aké Assi 8469 (G), Forêt de Mopri; 5°46'10.5"N, 4°56'34.87"W; 22 Jan. 1966 – Tiassalé • L. Aké Assi 4290 (G, P), Forêt d'Amitioro; 5°52'50.13"N, 4°52'46.45"W; 07 May. 1957. Nigeria – Cross River State • B.O. Daramola 641 (MO), Ikom Forest Reserve; 5°58'N, 8°42'E; 06 May. 1995 • J.O. Ariwaodo 447 (FHI, WAG); Ikom District, Efraya; 5°53'N, 8°42'E; 25 Mar. 1977 • M.G. Latilo FHI43924 (K); Ikom District, Cross River North Forest Res., between miles 156 & 157 on Ikom-Namfe road; 5°53'08.13"N, 8°47'51.41"E; 10 May. 1961 • P.A. Talbot 123 (K), Oban; 5°13'23.28"N, 8°33'06.9"E; 1911 • P.A. Talbot s.n (BM); Calabar, Oban; 5°13'23.28"N, 8°33'06.9"E; 1912 • P.P.C. van Meer 1490 (WAG), Ekinta River Forest Reserve, Northern part. Near and between pillar 18 and 19; 4°58'N, 8°35'E; 26 Apr. 1971 • P.P.C. van Meer 1664 (WAG); Ikom District, Cross River North Forest Reserve. Compt 1 and 2. 15 km SE of Ikom; 5°52'N, 8°46'E; 18 May. 1971 • P.P.C. van Meer 1736 (WAG); Ikom District, Cross River North Forest Reserve. Compt 1 and 2. 15 km SE of Ikom; 5°52'N, 8°46'E; 20 May. 1971 • R.W.J. Keay FHI28683 (K, P); Ikom District, Cross River North Forest Res.; 5°50'N, 8°50'E; 19 Jan. 1951 – Nassarawa State • A.P.D. Jones FHI16965 (P), Khaya HF nr. Onda enclave; 8°30'N, 7°48'E; 18 Feb. 1946 – Oyo State • C.F.A. Onochie FHI31539 (K); Ibadan District, c. 14 miles south of Ibadan on the Ijebu-Ode road; 7°11'00.66"N, 3°58'13.92"E; 19 Mar. 1958 • D.P. Stanfield FHI44949 (K, MO); Ibadan District, Gambari Forest Reserve; 7°07'59.88"N, 3°49'59.88"E; alt. 80 m; 11 Mar. 1964 • R.W.J. Keay FHI22812 (K); Ibadan District, Ibadan Forest Reserve, c. 22 miles south of Ibadan. School Enumeration area 1948; 7°03'02.43"N, 3°54'20.56"E; 22 Apr. 1948.

#### ﻿ Uvariodendron citriodorum

Taxon classificationPlantaeMagnolialesAnnonaceae

(Le Thomas) Dagallier & Couvreur, comb. et stat. nov.

urn:lsid:ipni.org:names:77326967-1

Figs 13 , 14

≡ Uvariodendron molundense var. citrata Le Thomas, Fl. Gabon No. 16, 283 (1969). Type. Gabon – Ogooué-Ivindo • N. Hallé 525 (holotype: P! (P00363400), sheet here designated, isotypes: K! (K000198798, K000198799), MO! (MO216993, MO216994), P! (P00363398), WAG! (WAG0065739)), Bélinga, mines de fer, sommet de Belvédère; 1°08'N, 13°12'E; alt. 800 m; 15 Aug. 1966.

##### Description.

Tree to shrub 4–5 m tall, D.B.H. unknown; young branches glabrous, old branches glabrous. Leaves with strong lemon smell when crushed. Petiole 6–10 mm long, 1.5–3.5 mm wide, sparsely pubescent to glabrous. Leaf lamina (183) 250–337 mm long, 72–97 mm wide, length:width ratio 2.3–4.2, elliptic to oblong, coriaceous, base acute to rounded (sometimes minutely decurrent at the very base, and decurrent on young leaves), apex attenuate, surface above glabrous, surface below glabrous; midrib impressed above, raised below, glabrous above, sparsely pubescent to glabrous below; secondary veins 12–18 pairs, weakly brochidodromous, impressed above, raised below; tertiary veins reticulate. Inflorescences borne on branches or axillary, composed of 1 sessile flower. Flowers bisexual, buds globose, sessile, 5–6 mm high, 5–7 mm in diameter, velutinous. Mature flower unknown, measures taken from flower buds or fruits. Bracts 1 to 4, upper bract ca. 5 mm long, ca. 7 mm wide, broadly ovate, semi clasping the pedicel, velutinous outside, glabrous inside. Sepals 3, ca. 7 mm long, ca. 6 mm wide (measures taken from bud), imbricate, velutinous outside, glabrous inside, color unknown. Outer petals 3, length, shape, indumentum and color unknown. Inner petals 3, length, shape, indumentum and color unknown. Stamens number unknown, ca. 1 mm long, ca. 0.2 mm wide, anthers linear, connective prolongation truncate. Carpels 10 to 20, ca. 2 mm long, ca. 0.4 mm wide, sparsely pubescent, free; stigma 0.5 mm long, 0.5 mm wide, pubescent. Fruiting pedicel 10–12 mm long, 2–5 mm in diameter, pubescent to glabrous. Monocarps 5 to 20, 25–40 mm long, 9–20 mm wide, length:width ratio 1.9–2.9, cylindrical, oblong, straight, truncate or rounded at apex, pubescent to glabrate, pale greyish green to red; sessile to shortly stipitate, stipe 0–5 mm long, 3–5 mm wide, pubescent to glabrous. Seeds 9–14 per monocarp, biseriate, 13 to 15 mm long, ca. 11 mm wide, semicircular.

**Figure 13.**
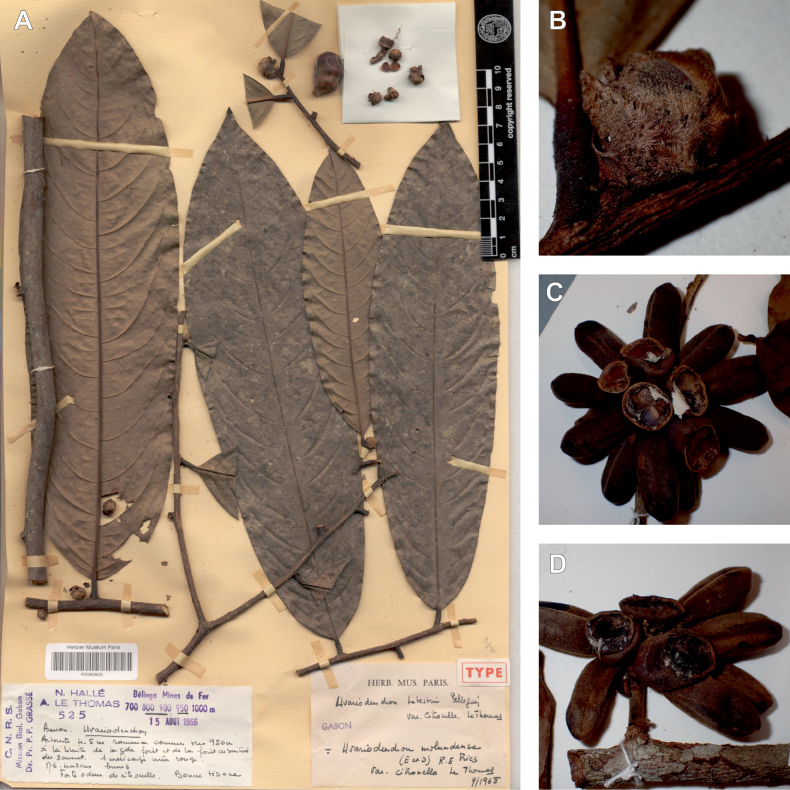
*Uvariodendroncitriodorum* (Le Thomas) Dagallier & Couvreur **A** entire specimen sheet with branch, leaves and flower buds **B** flower bud showing bracts, semi-side view **C** fruit with monocarps, some monocarps transversally cut, top view **D** fruit with monocarps, some monocarps transversally cut, side view. **A, B** Hallé 525 (type) **C, D** Sosef 2219. Photos Léo-Paul Dagallier.

##### Distribution.

Endemic to Lower Guinean Domain of the Guineo-Congolian Region. Known from one area in Gabon: Bélinga, mines de fer, and one locality in Republic of the Congo: Les Saras.

##### Habitat and ecology.

Mature rain forests on rocky soils. Altitude: 800–950 m a.s.l.

##### Phenology.

Fruits collected in July and November.

##### Vernacular names.

Gabon: ‘Bombamba’ in Bakota (Hallé 2896).

##### Etymology.

The specific epithet refers to the strong lemon smell of the plant. The epithet ‘*citrata*’ would prevail as it is the one given by Le Thomas for the variety. However, this specific epithet is already taken by *Uvariopsiscitrata* Couvreur & Niangadouma, from the genus *Uvariopsis*, which is closely related to *Uvariodendron*. Following the recommendation 23.A.3.h from Turland et al. (2018) (“Avoid [the specific epithets] that have been used before in any closely allied genus”), we choose the specific epithet ‘*citriodorum*’.

**Figure 14.**
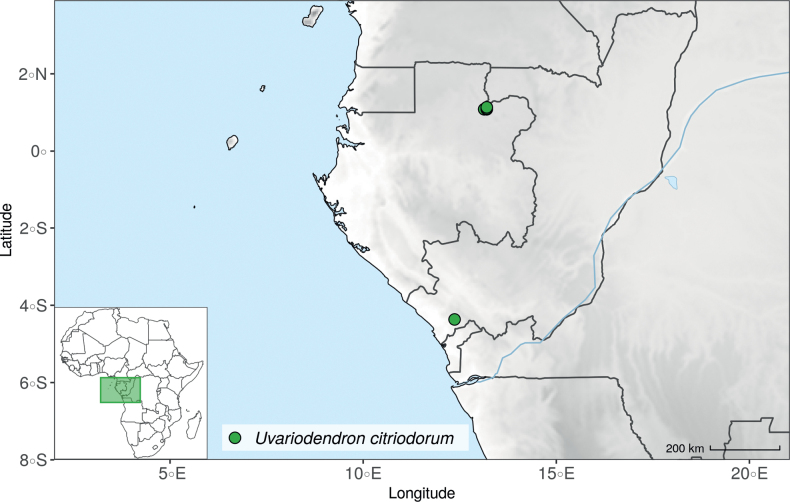
Distribution map of *Uvariodendroncitriodorum*. Shades of grey represent elevation, from white (sea level) to darker grey (higher elevation). The inset shows the extent of the map over Africa.

##### Notes.

This species differs from the other species, and particularly from *Ud.molundense*, in having leaves emitting a strong lemon scent when fresh material is crushed. Apart from this character, this species is morphologically very similar to *Ud.molundense*. However, as *Ud.citriodorum* and *Ud.molundense* are not monophyletic (Fig. 1, Suppl. materials 1, 2), they can’t be considered the same species following the phylogenetic species concept.

##### Preliminary conservation status.

This species is only known from one locality in Gabon and one locality in the Republic of the Congo. The EOO of the species is estimated at 2,256 km^2^ and its AOO at 20 km^2^. The unique occurrence in the Republic of the Congo dates back more than 30 years and the occurrences in Gabon are situated in Bélinga. Although the project of exploitation of the Bélinga iron ore deposits has been canceled, this locality is still threatened by a possible future exploitation of the ore. Following IUCN criterion B, it is thus assigned a preliminary conservation status of Endangered EN B1a(i,ii,iii,iv)+2a(i,ii,iii,iv).

##### Additional specimens examined.

Gabon – Ogooué-Ivindo • M.S.M. Sosef 2219 (BR, K, LBV, MO, WAG), Bélinga, Mines de Fer, 4 km on the road to Mvadi; 1°05'N, 13°12'E; alt. 900 m; 05 Nov. 2005 • N. Hallé 2896 (P), Bélinga; 1°05'N, 13°08'E; alt. 900 m; 29 Oct. 1964 • N. Hallé 3082 (P), Bélinga; 1°05'N, 13°08'E; alt. 950 m; 07 Nov. 1964 • N. Hallé 13 (P), Bélinga, mines de fer, route du Belvédère; 1°05'N, 13°12'E; alt. 950 m; 08 Jul. 1966 • N. Hallé (P), Bélinga, mines de fer, route du pt. B3; 1°06'N, 13°12'E; 25 Jul. 1966 • N. Hallé (K, MO, P), Bélinga, mines de fer; 1°08'N, 13°12'E; alt. 950 m; 16 Jul. 1966. Republic of the Congo – Kouilou • H. de Foresta 1759 (P), Les Saras, piste Cofibois, environs de la plantation Coobama de 1987; 4°22'S, 12°22'E; 25 Nov. 1988.

#### ﻿ Uvariodendron connivens

Taxon classificationPlantaeMagnolialesAnnonaceae

(Benth.) R.E.Fr., Acta Horti Berg. 10: 55 (1930)

Figs 7A–D , 15 , 16

≡ Uvaria connivens Benth., Trans. Linn. Soc. London 23(3): 465 (1862); Uvaconnivens Kuntze, Revis. Gen. Pl. 1: 8. (1891). Type. Equatorial Guinea – Bioko Sur • G. Mann 1159 (lectotype: K! (K000198803); isolectotypes: P! (P00362655), K! (K000198804, K000198805)), Fernando Po; 3°30'N, 8°40'E; 1861.
= Uvaria winkleri Diels, Bot. Jahrb. Syst. 38(3): 240 (1907). Type. Cameroon – South-West Region • H. Winkler 1466 (holotype: B (not found, destroyed?)); Moliwe.
= Uvaria megalantha Diels, Bot. Jahrb. Syst. 39(3–4): 472 (1907). Type. Cameroon – South Region • G.A. Zenker 3204 (holotype: B (not found, destroyed?); lectotype: WAG! (WAG0057972), designated by Couvreur et al (2022); isolectotypes: BM! (BM000636652), E! (E00147958), G! (G00412220), GOET! (GOET005733), HBG! (HBG502487), K! (K000198800), L! (L.1768578), M! (M0107939), P! (P01982908), S! (S07-13392), WU! (WU0025787), Z! (Z-000000876, Z-000000877)), Kamerun. Bipinde; 3°05'N, 10°25'E; 1904.

##### Description.

Tree 3–20 m tall, D.B.H. 2–25 cm; young branches glabrous, old branches glabrous. Petiole 5–21 mm long, 2–6 mm wide, glabrous. Leaf lamina 254–636 mm long, 66–177 mm wide, length:width ratio 2.3–6.2, elliptic to oblong to obovate, coriaceous, base acute to rounded, sometimes truncate or subcordate, apex acute to acuminate, acumen 5–24 mm long; surface above glabrous, surface below glabrous; midrib impressed above, raised below, glabrous above, glabrous below; secondary veins 16–28 pairs, weakly brochidodromous, impressed above, raised below; tertiary veins reticulate. Inflorescences borne on trunk and branches or axillary, composed of 1 flower. Flower pedicel (5) 10–40 mm long, 1.4–4 mm, increasing toward the apex up to 10 mm in diameter, pubescent to glabrous. Flowers bisexual, buds ovoid to globose, pedicellate, 9–22 mm high, 8–30 mm in diameter, pubescent. Bracts 1 to 6, upper bract 4–13 mm long, 6–17 mm wide, ovate, appressed, enclosing the bud, pubescent outside, glabrous inside. Sepals 3, 5–14 mm long, 7–17 mm wide, imbricate to fused at base, puberulent outside, glabrous inside, dull green. Outer petals 3, 14–32 mm long, 12–29 mm wide, length:width ratio 0.95–1.5, broadly ovate to ovate, pubescent to puberulent outside, puberulent to glabrous inside, wine red (cream in immature flowers) outside, wine red (cream in immature flowers) inside. Inner petals 3, 9.5–30 mm long, 8–23 mm wide, length:width ratio 0.9–1.9, broadly ovate to ovate, pubescent to puberulent outside, puberulent to glabrous inside, wine red (cream in immature flowers) outside, wine red (cream in immature flowers) inside. Stamens 1900 to 2500, 2–3.8 mm long, 0.3–0.5 mm wide, anthers linear, connective prolongation truncate. Carpels 5 to 33, 2–6.5 mm long, 0.8–1.5 mm wide, pubescent, free; stigma 0–2 mm long, 1–1.8 mm wide, coiled, pubescent, covered with an exudate at anthesis. Fruiting pedicel 13–31 mm long, 3–8 mm in diameter, glabrous. Monocarps 1 to 10, 22–55 mm long, 17–32 mm wide, length:width ratio 1.3–2, cylindrical to ovoid, longitudinally ridged, sparsely pubescent to glabrous, red to orange when ripe, green when immature; stipe 0–8 mm long, 3–10 mm wide, slightly pubescent to glabrous. Seeds 8–23 per monocarp, biseriate, 13 to 29 mm long, 3–14 mm wide, semicircular.

**Figure 15.**
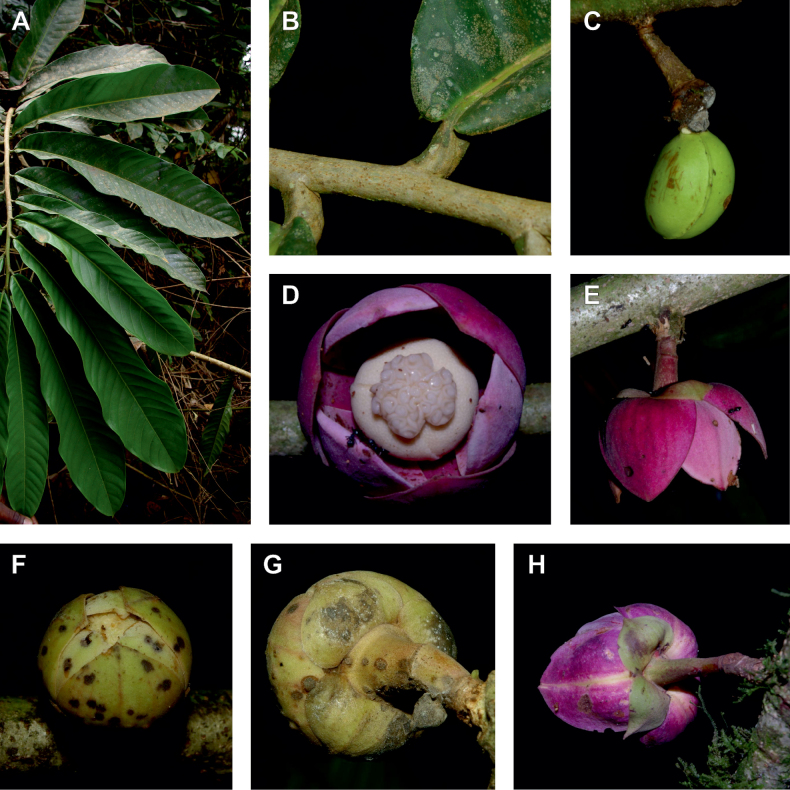
*Uvariodendronconnivens* (Benth.) R.E.Fr. **A** young branch with leaves, upper side **B** detail of petiole and base of leaf, upper side **C** fruiting material with unripe monocarp, side view **D** flower, top view **E** flower, side view **F** flower bud, top view **G** flower bud, bottom view **H** flower, one sepal removed, semi-bottom view. **A, B** Couvreur 383 **C, F, G** Couvreur 620 **D, E** Couvreur 1016 **H** Couvreur 1051. Photos Thomas Couvreur.

**Figure 16.**
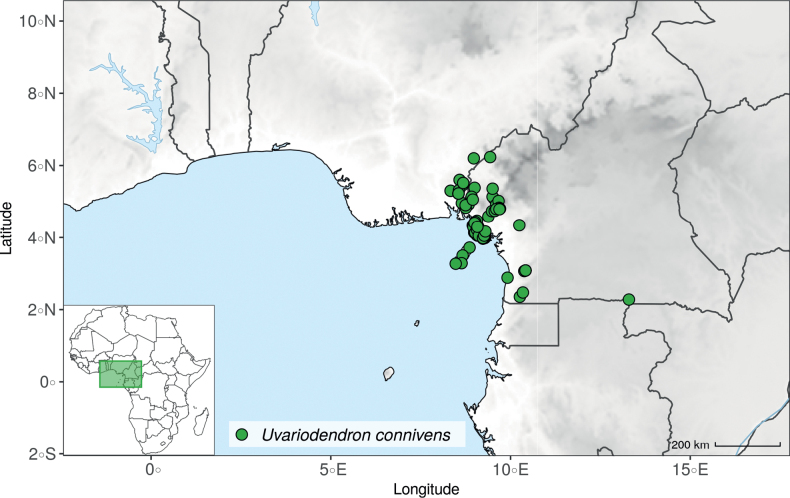
Distribution map of *Uvariodendronconnivens*. Shades of grey represent elevation, from white (sea level) to darker grey (higher elevation). The inset shows the extent of the map over Africa.

##### Distribution.

Endemic to Lower Guinean Domain of the Guineo-Congolian Region: Cameroon, Equatorial Guinea (Bioko Island) and Nigeria.

##### Habitat and ecology.

Lowland and premontane mature and old secondary rain forests, sometimes in swamp forests. Altitude: 50–1000 m a.sl.

##### Phenology.

Flowers and fruits collected all year.

##### Vernacular names.

Cameroon: ‘Ikeinju’ in Bakweri (Mbani 14).

##### Uses.

The young leaves are eaten (Cheek 5180) and the fruits are used for cough and dye (Mbani 14).

##### Notes.

This species is easily differentiated from the other species by its petals being wine red on both sides in mature flowers. Apart from this character, *Ud.connivens* resemble *Ud.calophyllum* and *Ud.fuscum* in having great leaves (more than 25 cm long). It can be distinguished from these species by being glabrous when young and old, and by having a distinct flower pedicel 10–40 mm long, whereas *Ud.calophyllum* and *Ud.fuscum* flowers are subsessile (flower pedicel 10–15 mm maximum in Ud.fuscumvar.magnificum).

##### Preliminary conservation status.

This species has been assessed previously as Near Threatened NT but the assessment needs to be updated (Peguy 1998). Here, the EOO of this species is estimated at 107,423 km^2^ and its AOO at 268 km^2^. Based solely on AOO value, it would qualify for Endangered, but none of the other B2 sub-criterion are met. It is relatively widespread in Cameroon with more than 10 locations, thus no longer qualifies for Near Threatened NT. Following IUCN criterion B, and it is assigned a preliminary updated conservation status of Least Concern LC.

##### Additional specimens examined.

Cameroon – Littoral • T.L.P. Couvreur 620 (MPU, YA), Ebo Wildlife Reserve, Djuma permanent camp. On Djuma-Djuma trail; 4°20'23.59"N, 10°14'41.58"E; alt. 335 m; 14 Feb. 2014 – South Region • G.A. Zenker 2624 (B, BM, G, K, L, M, MA, P, P, WAG), Bipindi; 3°05'N, 10°25'E; 1903 • G.A. Zenker 3401 (BM, G, K), Bipinde; 3°05'N, 10°25'E; 1907 • G.A. Zenker 3487 (BM, G, K), Bipinde; 3°05'N, 10°25'E; 1908 • G.A. Zenker 358 (B, G, M, P, P, U, WAG), Mimfia; 3°04'N, 10°23'E; Sep. 1913 • G.A. Zenker 3845 (BM, K), Bipindi; 3°05'N, 10°25'E; 1909 • J.J. Bos 5412 (P, WAG), 6 km S. of Kribi, 2–4 km E. of Grand Batanga road; 2°53'N, 9°55'E; 26 Sep. 1969 • T.L.P. Couvreur 383 (MPU, WAG, YA); Océan, Campo Ma an National Park, 5 km after main entrance; 2°21'19.48"N, 10°15'33.59"E; alt. 300 m; 15 Feb. 2012 • T.L.P. Couvreur 484 (MPU, YA), 15 km east from Lélé village; 2°16'39.39"N, 13°17'37.19"E; alt. 549 m; 09 Sep. 2013 • T.L.P. Couvreur 706 (MPU, WAG, YA), Campo Ma'an National Park, 11 km on trail from Ebinanemeyong village, on road, 7 km from Nyabessan to Campo town; 2°28'25.42"N, 10°20'39.1"E; 14 Feb. 2015 – South-West Region • B.-A. Nkongmeneck 959 (YA); Fako, Idenau, Mt Cameroun, versabt de Idenao. Feuille IGN 1/200 000 Buea/Douala; 4°12'N, 9°05'E; alt. 300 m; 23 Feb. 1985 • C.F. Tekwe 87 (K, SCA, YA), Isobi, above Isobe; 4°10'N, 9°00'E; alt. 40 m; 10 Jun. 1992 • D. Kenfack 1507 (MO), Mokoko; 4°27'N, 9°04'00.12"E; 24 Apr. 2001 • D. Kenfack 879 (MO); Ndian, Korup National Park, 11 km from Mundemba, along Fabe road; 5°05'03.43"N, 9°32'20.63"E; alt. 9 m; 11 Jul. 1997 • D.W. Thomas 2264 (K, MO, P, WAG, YA); Ndian, Map # NB 32 IV Buea-Douala. South Korup Reserve; 4°55'N, 8°50'E; alt. 50 m; Jul. 1983 • D.W. Thomas 4447 (K, MO, P, YA), forest and meadows on the gently sloping side of Mt Cameroun above small Koto village; 4°18'N, 9°06'E; alt. 550 m; 06 Mar. 1985 • D.W. Thomas 5533 (P, YA), forest between Kindonge and small Ekombe, Southern Bakundu Forest Reserve; 4°35'N, 9°23'E; alt. 200 m; 10 Feb. 1986 • D.W. Thomas 5537 (P, YA), at Bonenza, 2 km N of Limbe-Idenao road; 4°03'N, 9°05'E; alt. 300 m; 10 Feb. 1986 • D.W. Thomas 6928 (P, YA), footpath toward Matene from Mbilishe; 6°14'N, 9°26'E; alt. 400 m; 1987 • D.W. Thomas 7961 (MO, P, YA), Steep hillside south of Esukutang village; 5°23'N, 9°00'E; alt. 300 m; 25 May. 1988 • D.W. Thomas 9875 (K, SCA, YA), West of the Onge River and ridges on ‘Thump Mount'; 4°20'N, 8°57'E; alt. 200 m; 09 Nov. 1993 • F. Nguembock 76 (K); Fako, Mabeta-Moliwe Reserve; 3°58'N, 9°14'E; 10 Mar. 1992 • G. Mann 763 (BM, K), Ambas Bay; 4°01'N, 9°12'E; Feb. 1861 • G.K. Gottsberger 130307/21 (ULM, WAG), c. 300 m from Banyang Mbo Research Station; 5°08'N, 9°30'E; 13 Mar. 2007 • G.P. Tchouto Mbatchou 685 (K, SCA, YA), Bomana secondary forest. Transect OA, Plot OA0Z; 4°15'N, 9°01'E; alt. 200 m; 05 Oct. 1993 • I. von Rege 87 (K, SCA, YA); Fako, Mabeta, 6 km SE Limbe SBL; 3°59'N, 9°17'E; alt. 60 m; 11 Aug. 1993 • J. Bongyu 73 (K); Fako, Mabeta-Moliwe Reserve; 3°58'N, 9°14'E; 06 Apr. 1992 • J. Nemba 550 (MO, P, YA), Secondary growth and old growth forest along Kumba-Mamfe road at mile 14 Lkiliwindi; 4°44'N, 9°29'E; alt. 200 m; 15 Jun. 1987 • J. Nemba 56 (L, P, U, YA), 5 kms west of Kumba-Mamfe road near Konye; 4°55'N, 9°36'E; alt. 300 m; 25 Mar. 1986 • J.-P. Ghogue 1551 (YA), Bimbia Bonadikombo (former Mabeta Moliwe), 18 km SE Limbe; 3°59'36"N, 9°15'43"E; alt. 50 m; 10 Apr. 2003 • J.F. Villiers 2483 (P, YA), 4 km E Bomana, 34 NW limbé; 4°12'55.22"N, 9°06'11.21"E; 14 Dec. 1984 • J.I. Wheatley 194 (K, SCA, YA); Fako, Mabeta-Moliwe TC 10; 4°01'N, 9°16'E; alt. 50 m; 20 Apr. 1992 • J.I. Wheatley 326 (K, SCA, YA); Fako, Mabeta-Moliwe TD 5835 m; 4°01'N, 9°16'E; alt. 100 m; 24 Jun. 1992 • J.J. Wieringa 45 (WAG); Fako, Limbe, Bakingini, forest above ‘mile 11 village'; 4°04'10"N, 9° 03'50"E; alt. 160 m; 20 Jan. 1994 • J.J. Wieringa 5839 (BR, FHO, MO, WAG), Bakingini, at edge of plantation area above Mile 11; 4°04'10.8"N, 9°03'27.6"E; alt. 150 m; 06 Mar. 2007 • J.M. Dalziel 8243 (K), Buea1, Buea to Mayuko; 4°09'N, 9°14'E; 13 Feb. 1927 • J.M. Mbani 14 (K, SCA, YA), Njonji; 4°08'11.04"N, 8°59'22.56"E; alt. 100 m; 13 Feb. 1992 • J.M. Mbani 382 (K, SCA, YA), Ekumbe Mofako, Plot M19; 4°28'N, 9°04'E; 21 May. 1994 • J.P. Watts 245 (K, YA), Moliwe, Makota River watershed.TD 5832 m; 4°00'N, 9°15'E; alt. 100 m; 29 Apr. 1992 • J.P. Watts 394 (K, SCA, YA), c.5 km South East of Moliwe, TF +4000 m; 4°02'N, 9°17'E; alt. 100 m; 09 Jun. 1992 • J.P. Watts 782 (K, SCA, YA), forest to the West of Onge River, about 4 km West of Liwenyi village (c 14 km North of Idenau); 4°23'N, 8°59'E; alt. 260 m; 28 Oct. 1993 • M. Etuge 156 (MA, MO, P, WAG, YA), Bakolle Bakossi, on Kumba – Mamfe road; 5°01'N, 9°40'E; alt. 350 m; 24 May. 1986 • M. Etuge 2390 (K, YA), Max's trail, Nyasoso; 4°49'39.72"N, 9°40'51.96"E; alt. 1100 m; 24 Jun. 1996 • M. Etuge 2396 (K, YA), Max's trail, Nyasoso; 4°49'39.72"N, 9°40'51.96"E; alt. 1100 m; 24 Jun. 1996 • M. Etuge 6506 (YA), Mungo River F.R., Mungo F.R; 4°44'17"N, 9°33'38"E; 22 Feb. 2006 • M.R. Cheek 5180 (K, P, SCA, YA), Liwenyi, Low altitude forest on the West bank of the Onge river above the first set of rapids, that is about 1–2 hours walk inland from Enyenge. Grid ref. and alt. approx. Local names and uses from Clement Offu (Enyenge); 4°17'N, 8°58'E; alt. 50 m; 28 Oct. 1993 • M.R. Cheek 5462 (K, SCA, YA), Low altitude forest above oil palm plantation. Reached after c. 40 minutes walk N then E from Njonji. Hunters path to ‘Lake Njonji'. Little farming, but many gaps and fallen trees at low altitude, c. 150–300 m, crossing and running alongside substantial seasonal stream. At c. 400 m, Hypselodelphyus-Aframomum thicket dominates; 4°08'N, 9°01'E; alt. 300 m; 18 Nov. 1993 • M.R. Cheek 8164 (K, YA); Ndian, Korup National Park, Ekundu Kundu, Transect 10, c. 1100 m; 5°08'N, 8°55'E; alt. 170 m; 25 Apr. 1996 • N. Ndam 1076 (K, SCA), Bonjare, Plot 09; 4°26'N, 9°01'E; alt. 220 m; 30 Apr. 1994 • N. Ndam 1118 (K, SCA), Bonjare, Plot 10; 4°26'N, 9°01'E; alt. 270 m; 01 May. 1994 • N. Ndam 708 (K, YA), Bomana-Koto Rd c 500 m Bearing 305deg towards Onge river 3 hr walk from the rd; 4°13'N, 9°04'E; alt. 400 m; 18 Oct. 1993 • P. Nkeng 37 (K, SCA, YA), Etome; 4°03'N, 9°07'E; alt. 300 m; 02 Mar. 1992 • R.G. Letouzey 15175 (P, YA); Ndian, Rivières Mosongosele et de Ndian depuis Mosongosele jusqu'à l'entrée amont de la mangroce, env. 20 km au SW de Mundemba (feuille IGN 1/200 000 Buea-Douala); 4°49'27.3"N, 8°45'15.72"E; 13 Jun. 1976 – R.G. Letouzey 15177 (MO, P, P, WAG, YA), Au SW de Mosongosele, 20 km WSW Mendumba; 4°54'N, 8°45'E; 14 Jun. 1976 • S. Cable 2329 (K, YA); Ndian, Korup National Park, Ekundu Kundu, path to Esoki about 4 km from Ekundu-Kundu (as on 27^th^); 5°03'16.8"N, 8°56'58.5"E; alt. 350 m; 29 Apr. 1996 • S. Cable 611 (K, SCA, YA), Dikulu, Coastal lowland rain forest along Mangrove stream; 3°59'N, 9°14'E; alt. 50 m; 17 Dec. 1993 • S.N. Ekema 1078 (K, YA), Mokoko Forest Reserve, Boa/Likinge; 4°24'N, 9°00'E; alt. 150 m; 31 May. 1994 • T.C.H. Sunderland 1264 (K, SCA, YA), Mabeta-Moliwe: TB 6000 m; 4°03'N, 9°16'E; alt. 40 m; 22 Apr. 1992 • T.C.H. Sunderland 1536 (K, K, SCA, YA), Nyasoso, Mt Kupe: Max's Trail; 4°48'05.04"N, 9°42'29.16"E; alt. 1600 m; 09 Jul. 1992 • T.D. Maitland 537 (K, P), Buea area, at Balifamba; 4°10'N, 9°18'E; alt. 731 m; 1929 • T.L.P. Couvreur 1016 (MPU, WAG, YA), Bayang Mbo Wildlife Sanctuary, after Mbu river; 5°21'26.28"N, 9°30'05.88"E; alt. 253 m; 26 Mar. 2016 • T.L.P. Couvreur 1051 (MPU, WAG, YA), Mt Cameroon National Park, on the Bomona trail, behind Bomona village, 10 km NW from Idenau; 4°17'48.27"N, 9°04'43.77"E; alt. 690 m; 03 Apr. 2016 • W.G. Gosline 235 (K, YA), Kupe Village, trail to Kupe rock saddle; 4°47'10"N, 9°41'30"E; alt. 950 m; 28 Nov. 1999 • W.J. Baker 294 (K, SCA, YA); Fako, Mabeta, 6 km SE Limbe SBL; 3°59'N, 9°17'E; 10 Aug. 1993 – Unknown major area • W.G. Gosline 209 (K, WAG, YA), Meme Division, Mahole-Bintulu road; 4°47'30"N, 9°36'12"E; alt. 300 m; 24 Nov. 1999. Equatorial Guinea – Bioko Norte • M.G. Carvalho 4220 (K, MA), BIOCO: Malabo – Cupapa, km 22–23, margenes del rio Ejoa, 32NMK9005, 200 m; 3°43'12"N, 8°51'36"E; 06 Jul. 1989 – Bioko Sur • W.R.Q. Luke 11955 (EA, K, MA), Moaba – Moka trail, Biadyi River Camp Pt 140; 3°17'06.4"N, 8°38'17.81"E; alt. 650 m; 16 Mar. 2007 • W.R.Q. Luke 13184 (K, MA), Moraka pt 340 to 342; 3°16'13.8"N, 8°28'29.64"E; alt. 3 m; 28 Jan. 2009. Nigeria – Cross River State • C.F.A. Onochie FHI36080X (K); Calabar, Oban Group Forest Reserve, between Akor and Orem; 5°36'N, 8°35'E; 21 Jan. 1957 • E.U. Ujor FHI31636 (K); Calabar, Ikot Ewa; 4°57'24.84"N, 8°38'44.88"E; 05 Jul. 1952 • H.D. Onyeachusim FHI54055 (K); Calabar, between miles 57–58 Osomba village on Calabar-Mamfe road; 5°27'23.32"N, 8°39'55.34"E; 21 Feb. 1964 • M.C. Ejiofor FHI21898 (K); Calabar, Oban Group Forest Reserve; 5°36'N, 8°35'E; 07 May. 1952 • M.G. Latilo 23 (K); Calabar, Akamkpa Rubber Estate. Calabar River Division; 5°18'N, 8°20'E; 21 Mar. 1959 • M.G. Latilo 32 (K, MO); Calabar, Akamkpa Rubber Estate. Dukwe felling area. Calabar River Division; 5°18'N, 8°20'E; 23 Mar. 1959 • P.A. Talbot 1254 (BM, K), Oban; 5°13'23.28"N, 8°33'06.9"E; 1911 • P.A. Talbot 158 (BM); Calabar, Oban; 5°19'N, 8°34'E; 1911 • P.A. Talbot 402 (BM), Oban; 5°13'23.28"N, 8°33'06.9"E; 1911 • P.A. Talbot 404 (BM, K), Oban; 5°13'23.28"N, 8°33'06.9"E; 1911 • P.A. Talbot 433 (BM), Oban; 5°13'23.28"N, 8°33'06.9"E; 1911 • P.A. Talbot 434 (BM), Oban; 5°13'23.28"N, 8°33'06.9"E; 1911 • P.A. Talbot 82 (BM, K), Oban; 5°13'23.28"N, 8°33'06.9"E; 1911 • P.A. Talbot s.n (BM); Calabar, Oban; 5°13'23.28"N, 8°33'06.9"E; 1912 • P.P.C. van Meer 1412 (WAG); Calabar, Oban Group Forest Reserve, East Block; 5°31'N, 8°41'E; alt. 200 m; 21 Apr. 1971 • P.P.C. van Meer 1430 (U); Calabar, Oban Group Forest Reserve, East Block; 5°31'N, 8°41'E; alt. 200 m; 22 Apr. 1971 • P.P.C. van Meer 1430 (WAG); Calabar, Oban Group Forest Reserve, East Block; 5°31'N, 8°41'E; alt. 200 m; 22 Apr. 1971 • R.W.J. Keay FHI28191 (K); Ikom District, Afi River Forest Reserve, near Aboabam, forest by river Nkem; 6°11'52.16"N, 8°58'40.48"E; 09 Dec. 1950.

#### ﻿ Uvariodendron dzomboense

Taxon classificationPlantaeMagnolialesAnnonaceae

Dagallier, Q.Luke & Couvreur, PhytoKeys 174: 114 (2021)

**Figure 17.**
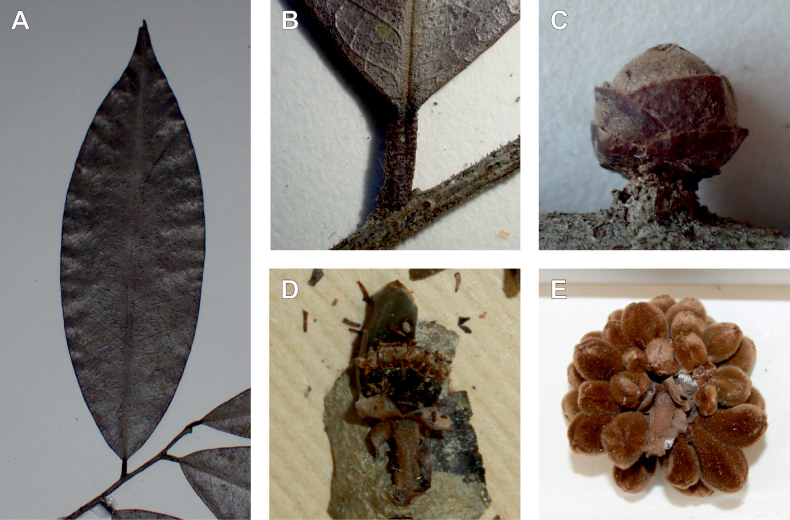
*Uvariodendrondzomboense* Dagallier, Q.Luke & Couvreur **A** leaf, upper side **B** petiole and base of leaf, upper side **C** flower bud showing bracts, side view **D** flower, two outer petals and three outer petals removed **E** fruit with young monocarps. **A–C** Luke 7443 **D, E** Robertson MDE 207. Photos Léo-Paul Dagallier.

**Figure 18.**
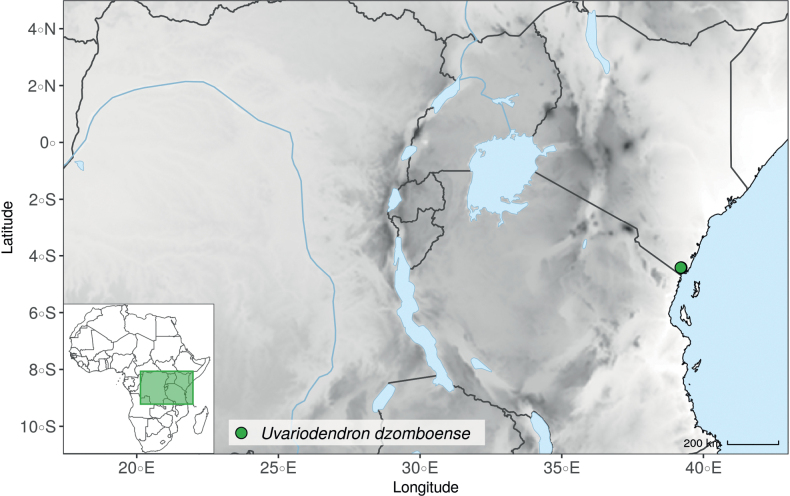
Distribution map of *Uvariodendrondzomboense*. Shades of grey represent elevation, from white (sea level) to darker grey (higher elevation). The inset shows the extent of the map over Africa.

Figs 17 , 18 ; Table 3

##### Type.

Kenya – Coast • S.A. Robertson MDE207 (holotype: K! (no barcode); isotypes: EA!, MO!, WAG! (WAG0065798)), Kwale District – Dzombo Hill; 4°26'S, 39°13'E; alt. 300 m; 07 Feb. 1989.

##### Description.

Tree 4–7 m tall, D.B.H. unknown; young branches sparsely pubescent to glabrous, old branches glabrous; leaf bud ‘eragrostiform’, composed of 5, distichous, longitudinally folded, velutinous scales. Leaves with margin slightly revolute. Petiole 3–4 mm long, 1–1.5 mm wide, sparsely pubescent to glabrous. Leaf lamina 65–132 mm long, 20–45 mm wide, length:width ratio 2.9–3.6, elliptic to narrowly elliptic, coriaceous, base acute to slightly decurrent, apex attenuate, surface above glabrous, surface below sparsely pubescent to glabrous when young, glabrous when old; midrib impressed above, raised below, glabrous above, slightly pubescent to glabrous below; secondary veins 12–13 pairs, weakly brochidodromous, impressed above, raised below; tertiary veins reticulate. Inflorescences borne on trunk and branches, composed of 1 flower. Flower pedicel 8–30 mm long, 2–2.5 mm in diameter, densely pubescent. Flowers bisexual, buds globose, sessile, ca. 4 mm high, ca. 4.5 mm in diameter, sparsely pubescent. Bracts 6 at base of the pedicel in flower bud, generally the uppermost remaining towards the lower half of the pedicel on mature flower, 5–6 mm long, 5–8 mm wide, ovate, pubescent to shortly pubescent outside, glabrous inside. Sepals 3, 5–7 mm long, 4.5–7 mm wide, fused at base, pubescent to shortly pubescent outside, glabrous inside, color unknown. Outer petals 3, ca. 16 mm long, ca. 9 mm wide, length:width ratio ca. 1.8, ovate, shortly velutinous outside, glabrous inside, color unknown. Inner petals 3, ca. 18 mm long, ca. 8 mm wide, length:width ratio ca. 2.3, obovate, shortly velutinous outside, glabrous inside, color unknown. Stamens 700 to 1000, 2 mm long, 0.5 mm wide, anthers linear, connective prolongation truncate. Carpels 50 to 75, ca. 2 mm long, 1–1.5 mm wide, densely pubescent, free; stigma unknown. Fruiting pedicel ca. 14 mm long, ca. 4 mm in diameter, pubescent. Monocarps (unripe?) ca. 35, ca. 15 mm long, ca. 10 mm wide, length:width ratio ca 1.5, ovoid, densely pubescent, golden brown, sessile. Seeds (unripe?) ca. 5 per monocarp, uniseriate, ca. 4.5 mm long, ca. 10 mm wide.

##### Distribution.

Endemic to Somalia-Masai Region. Only known from one locality in Kenya: Dzombo Hill.

##### Habitat and ecology.

Moist semi-deciduous forest. Soil: igneous intrusion. Altitude: 270–300 m a.s.l.

##### Phenology.

Flowers collected in January and June. Fruits collected in February.

##### Notes.

This species differs from the other *Uvariodendron* species by the combination of small (i.e. less than 150 mm long) narrowly elliptic to elliptic leaves and 50–75 densely pubescent carpels. It differs from *Ud.kirkii* by its smaller leaves (132 mm maximum versus 210 mm maximum) and higher number of carpels (50–75 versus 7–20).

##### Preliminary conservation status.

Previous work estimated the EOO and AOO of this species to be less than 6 km^2^, and assigned a preliminary status of Endangered EN B1ab(iii)+2ab(iii) (Dagallier et al. 2021).

##### Additional specimens examined.

Kenya – Coast • W.R.Q. Luke 1654 (EA, K); Kwale District, Dzombo Forest Reserve; 4°25'S, 39°13'E; alt. 270 m; 06 Jan. 1989 • W.R.Q. Luke 2884 (EA, K); Kwale District, Dzombo Forest Reserve; 4°25'S, 39°13'E; alt. 271 m; 04 Oct. 1991 • W.R.Q. Luke 3370 (EA); Kwale District, Dzombo Forest Reserve; 4°25'S, 39°13'E; alt. 272 m; 12 Nov. 1992 • W.R.Q. Luke 7443 (EA); Kwale District, Dzombo; 4°25'S, 39°12'E; alt. 270 m; 28 Jun. 2001.

#### ﻿ Uvariodendron fuscum

Taxon classificationPlantaeMagnolialesAnnonaceae

(Benth.) R.E.Fr., Acta Horti Berg. 10: 61 (1930)

**Figure 19.**
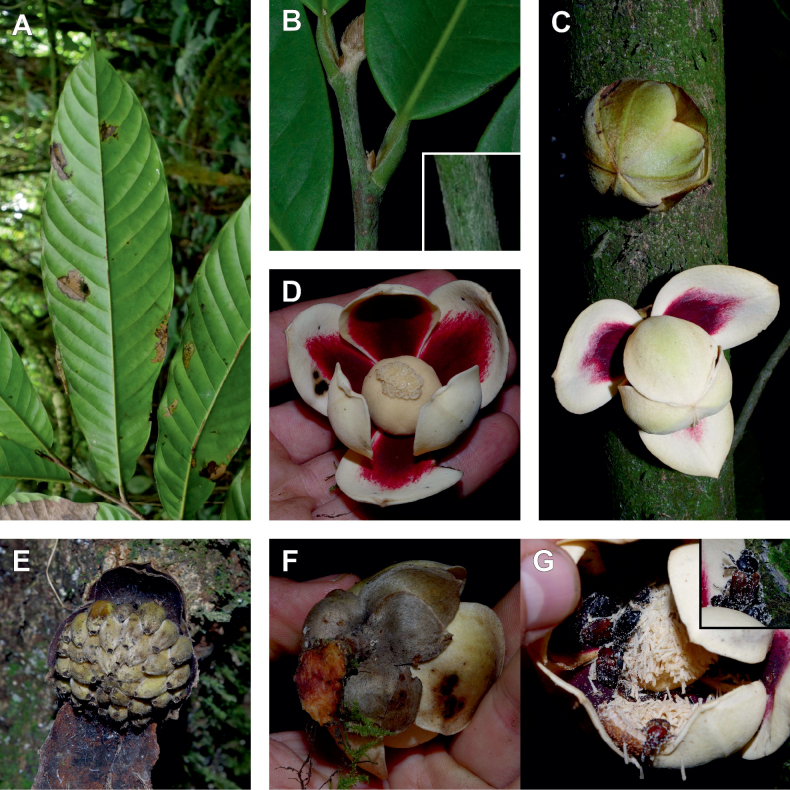
Uvariodendronfuscum(Benth.)R.E.Fr.var.fuscum**A** leaf, lower side **B** apex of young branch with detail of petiole and leaf base, upper side, inset: detail of sparse pubescence on young branch **C** trunk with flower bud (top) and flower (bottom) **D** open flower **E** young fruit with unripe monocarps, one old petal remaining **F** flower semi-bottom view, showing bracts and sepals **G** detail of flower after anthesis, note the falling stamens and pollinator insects, inset: detail of Coleoptera full of pollen grains. **A, E, G** Couvreur 1046 **B, C** Couvreur 1029 **D, F** Couvreur 990. Photos Thomas Couvreur.

**Figure 20.**
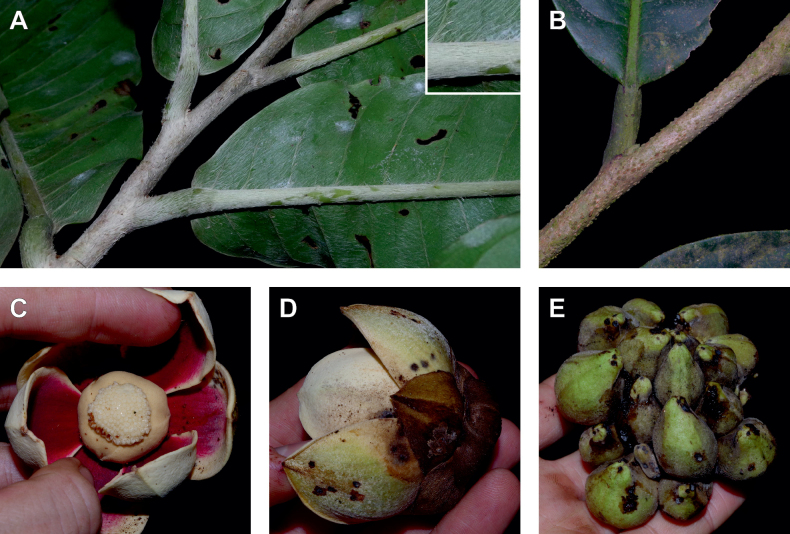
Uvariodendronfuscumvar.giganteum (R.E.Fr.) Dagallier & Couvreur **A** young branch with leaves, lower side, inset: detail of midrib, note the long soft hairs producing **A** whitish appearance **B** young branch, petiole and base of leaf, upper side, note the absence of hairs (fallen off) **C** flower, top view **D** flower, side view showing sepals and bracts **E** young fruit, top view. **A** Couvreur 1229 **B–D** Couvreur 1057. Photos Thomas Couvreur.

**Figure 21.**
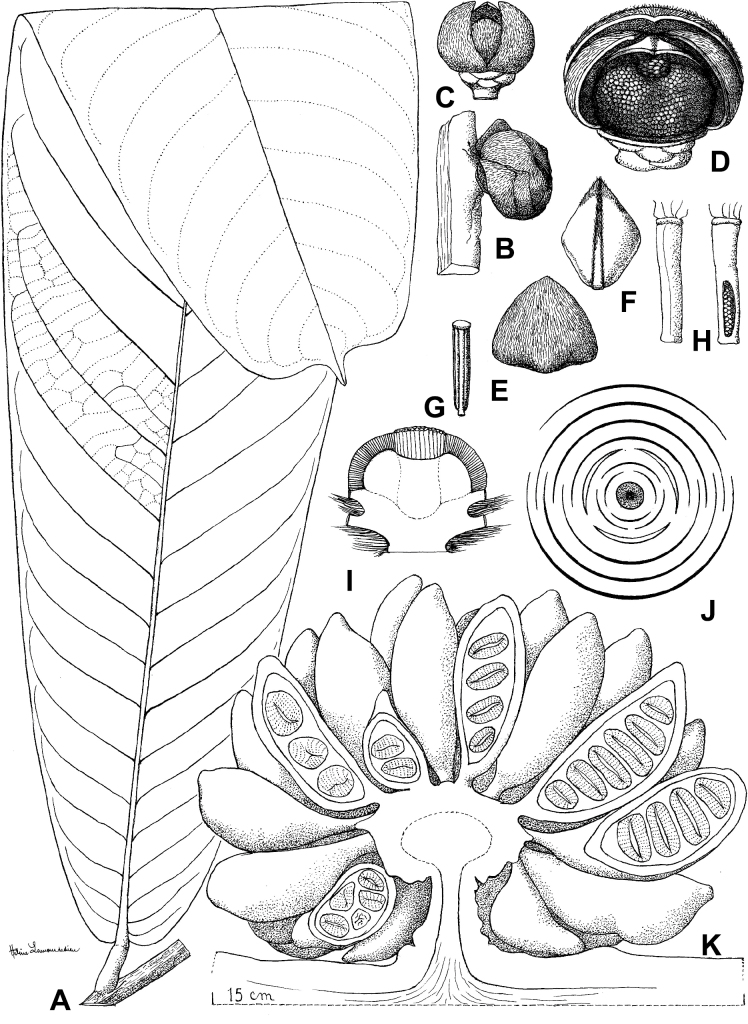
Uvariodendronfuscumvar.giganteum (R.E.Fr.) Dagallier & Couvreur **A** leaf **B** flower bud, side view **C** flower bud, bracts removed, side view **D** detail of flower bud, two sepals, one outer and two inner petals removed **E** outer petal, outer view **F** inner petal, outer view **G** stamen **H** carpels, side view and detail of ovules **I** longitudinal section of receptacle **J** floral diagram **K** fruit, longitudinal sections of monocarps. **A–K** from Hallé 3156 (as *Ud.giganteum*). Drawings by Hélène Lamourdedieu, modified from Le Thomas (1969; pl. 50, p. 279), Publications Scientifiques du Muséum national d’Histoire naturelle, Paris.

**Figure 22.**
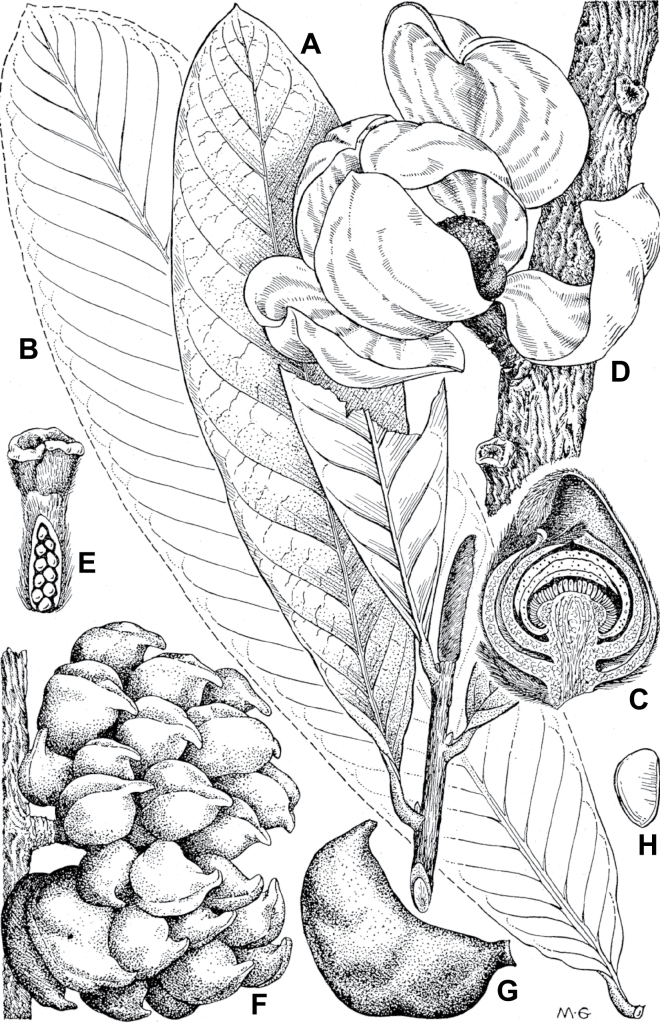
Uvariodendronfuscumvar.magnificum (Verdc.) Dagallier & Couvreur **A** young branch with leaves and apical bud **B** leaf, upper side **C** longitudinal section of flower bud **D** flower, semi-side view **E** carpel with carpel wall partially removed to show arrangement of ovules, side view **F** young fruit, side view **G** monocarp, side view **H** seed, side view. **A–H** from Okodi in Hamilton 696 (as *Ud.magnificum*). Drawings by Mary Griesrson, modified from Verdcourt (1969; fig. 2, p. 517), Kew Bulletin 1969, © Board of Trustees of the Royal Botanic Gardens, Kew.

**Figure 23.**
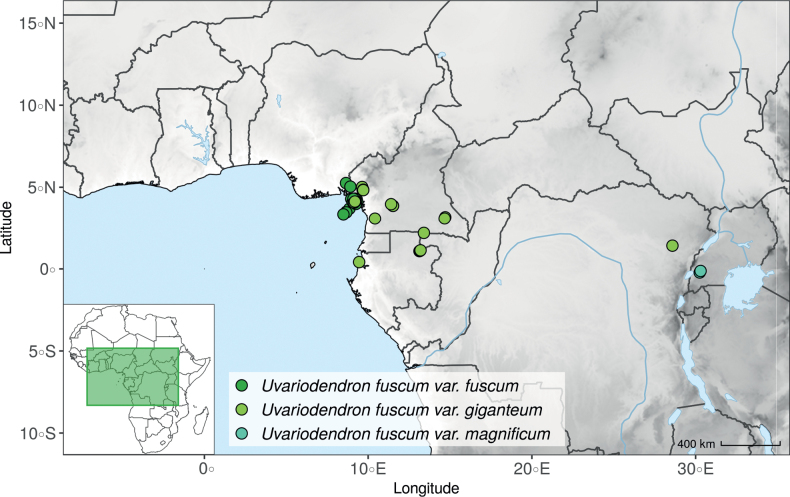
Distribution map of *Uvariodendronfuscum*. Shades of grey represent elevation, from white (sea level) to darker grey (higher elevation). The inset shows the extent of the map over Africa.

Figs 4 , 19 , 20 , 21 , 22 , 23 ; Table 4

≡ Uvaria fusca Benth., Trans. Linn. Soc. London 23(3): 466 (1862); Uvafusca Kuntze, Revis. Gen. Pl. 1: 8 (1891). Type. Equatorial Guinea – Bioko Sur – G. Mann 308 (holotype: K! (K000198801); isotype: P! (P00362657)); 3°30'N, 8°40'E; alt. 396 m; 1860.
= Uvariodendron mirabile R.E.Fr., Acta Horti Berg. 10 : 59 (1930). Type. Cameroon – South-West Region • P.R. Preuss 1378 (lectotype: P! (P00315830), designated by Couvreur et al. (2022), B destroyed), zwischen Victoria und Bimbia; 3°58'58.55"N, 9°15'19.17"E; 15 Mar. 1898.
= Uvaria gigantea Engl.; Uvagigantea Kuntze; Uvariodendrongiganteum (Engl.) R.E.Fr.; concerning Uvariodendronfuscumvar.giganteum (see details under this variety).
= Uvariodendron magnificum Verdc.; syn. nov. concerning Uvariodendronfuscumvar.magnificum (see details under this variety).

##### Description.

Tree 3–15 m tall, D.B.H. 5–35 cm; young branches with long soft hairs producing a whitish appearance quickly falling off to glabrous, old branches glabrous. Petiole 4–35 mm long, 2–8 mm wide, pilose to glabrous. Leaf lamina 160–750 mm long, 43–225 (250) mm wide, length:width ratio (2.1) 2.5–4, narrowly elliptic to elliptic to narrowly obovate, coriaceous, base acute to cuneate to rounded, apex rounded to acuminate, acumen 1–23 mm long; surface above glabrous, surface below pilose to glabrous when young, glabrous when old; midrib impressed above, raised below, glabrous above, pilose to glabrous below; secondary veins 15–33 pairs, weakly brochidodromous, impressed above, raised below; tertiary veins reticulate. Inflorescences borne on trunk and branches, composed of 1–2 (sub)sessile to pedicellate flowers. Flower pedicel 0–15 mm long, 3–6 mm in diameter, velutinous. Flowers bisexual, buds globose, sessile, 6–15 mm high, 6.5–15 mm in diameter, velutinous. Bracts 1 to 6, upper bract 8–35 mm long, 10–50 mm wide, broadly ovate, clasping the pedicel, enclosing the sepals, pubescent outside, glabrous inside. Sepals 3, 11–55 mm long, 13–43 mm wide, ovate, fused at base over 20–50% of their length, velutinous outside, glabrous inside, brown. Outer petals 3, 20–70 mm long, 17–47 mm wide, length:width ratio 1.1–1.7, elliptic to broadly ovate, velutinous outside, glabrous inside, cream to greenish outside, cream with a dark red streak from base up to 75% of the petal length inside. Inner petals 3, 20–53 mm long, 15–38 mm wide, length:width ratio 1.1–2.1, elliptic to broadly obovate to obovate, puberulent outside, glabrous inside, cream outside, dark red to dark purplish red with cream margins inside. Stamens around 2500, 3.5–5 mm long, 0.1–0.5 mm wide, anthers linear, connective prolongation truncate, pale yellow. Carpels 20 to 160, 4–7 mm long, 0.5–2.2 mm wide, velutinous, free; stigma 1–2 mm long, 0.8–1.1 mm wide, coiled, glabrous to velutinous, covered with an exudate at anthesis. Fruiting pedicel 5–18 mm long, 5–8 mm in diameter, pubescent. Monocarps 6 to 80, 20–60 mm long, 11–32 mm wide, length:width ratio 1.7–2.5, cylindrical, curved, acuminate, puberulent, green to brown; sessile to shortly stipitate, stipe 0–4 mm long, 2–5 mm wide, pubescent to glabrate. Seeds 1–16 per monocarp, biseriate, 14 to 23 mm long, 7–11 mm wide, semicircular, orange-brown.

##### Distribution.

Element of the Lower Guinean Domain and Congolia Domain of the Guineo-Congolian Region and Zambezian Region: Cameroon, Democratic Republic of the Congo, Gabon, Equatorial Guinea (Bioko Island), Nigeria, Uganda.

##### Habitat and ecology.

Lowland and submontane to mountain mature or old secondary rain forests. Altitude: 100–1400 m a.s.l.

##### Phenology.

Flowers collected from January to April. Fruits collected from March to April.

##### Vernacular names.

Cameroon: ‘Limboto’ in Bakweri (van Andel 3761), ‘Obom Ossoé’ in Yaoundé (Biholong 279). Gabon: ‘Inkaca’ in Bakota (Hallé 3156).

##### Uses.

The young leaves are boiled and used for soup (Cheek 5145) and the ripe fruits are eaten (van Andel 3761).

##### Notes.

From now on, the name *Ud.fuscum* encompasses the synonyms *Ud.mirabile*, *Ud.giganteum* and *Ud.magnificum*. *Ud.fuscum* consists of a large morphological variation of the leaf and flower size, with var. magnificum having the largest dimensions, followed by var. giganteum and then the type variety being the smallest (Fig. 4, Table 4). Although this variation seems to form a continuum, we recognize three different morphological groups described as varieties (see notes under the varieties). This species resembles *Ud.calophyllum*, *Ud.connivens* and *Ud.usambarense* in having large elliptic to obovate leaves. It differs from *Ud.calophyllum* and *Ud.connivens* in having pilose to glabrous young branches and petioles (vs. tomentose in *Ud.calophyllum* and completely glabrous in *Ud.connivens*). It differs from *Ud.connivens* in having flower pedicels between 0 and 15 mm long (vs. between 10 and 40 mm long) and in having cream petals with dark red streak within the flower (vs. wine red petals both inside and outside).

##### Preliminary conservation status.

This species is widespread, distributed from Nigeria to Uganda. A previous assessment listed it as Near Threatened NT (Cheek and Cable 2000). However, the assessment was made on what is now Ud.fuscumvar.fuscum and thus needs to be updated. Here, the EOO is estimated at 755,899 km^2^ and its AOO at 152 km^2^. Based solely on AOO value, it would qualify for Endangered EN, but none of the other B2 subcriterion are met. Following IUCN criterion B, it is assigned a preliminary conservation updated status of Least Concern LC.

#### ﻿ Uvariodendron fuscum var. fuscum

Taxon classificationPlantaeMagnolialesAnnonaceae

Figs 19 , 23 ; Table 4

##### Description.

Young branches sparsely pubescent to glabrous. Petiole 4–16 mm long, 2–5 mm wide. Leaf lamina 160–450 mm long, 43–118 mm wide, length:width ratio (2.1) 2.8–3.9, Base acute, apex acute to acuminate, surface below glabrate when young, glabrous when old; midrib sparsely pubescent to glabrous below; secondary veins 15–24 pairs. Flower pedicel 0–5 mm long. Bracts 1 to 6, upper bract 10–25 mm long, 14–25 mm wide. Sepals 11–23 mm long, 13–26 mm wide. Outer petals 20–39 mm long, 17–26 mm wide. Inner petals 20–42 mm long, 15–26 mm wide, length:width ratio 1.3–2.1, broadly obovate to obovate. Carpels 20 to 70. Fruiting pedicel ca. 5 mm long. Monocarps (only unripe fruits seen).

##### Distribution.

Endemic to Lower Guinean Domain of the Guineo-Congolian Region: Cameroon, Equatorial Guinea (Bioko Island), Nigeria.

##### Habitat and ecology.

Submontane to mountain mature or old secondary rain forests. Altitude: 800–1400 m a.s.l.

##### Additional specimens examined.

Cameroon – South-West Region • A. Dahl 622 (K), trail north of Likombe village; 4°14'N, 9°11'E; alt. 1060 m; 02 Mar. 1995 • B.-A. Nkongmeneck 891 (YA), Mt Cameroun, flanc d'Ekona Lelu, feuille IGN: 1/200 000 Buea-Douala; 4°16'N, 9°18'E; alt. 1300 m; 14 Jan. 1985 • D.W. Thomas 4469 (K, MO, P, P, YA), forest and meadows on the gently sloping side of Mt Cameroun above small Koto village; 4°18'N, 9°06'E; alt. 550 m; 06 Mar. 1985 • E.W.G. Kalbreyer 41 (K); 4°01'N, 9°12'E; 1877 • G.W.J. Mildbraed 10720 (K), Likomba – Pflangzung, 15–35 km NE of Victoria; 4°06'N, 9°20'E; alt. 50 m; Nov. 1928 • H. Lehmbach 178 (B), Buea; 4°09'N, 9°14'E; alt. 1800 m; 16 Jan. 1898 • H. Lehmbach 57 (B, K), Buea1, Buea; 4°09'N, 9°14'E; alt. 1000 m; 26 Apr. 1897 • J.J. Wieringa 2058 (WAG); Fako, Mt Etinde (=Small Mt Cameroon), near and at summit; 4°05'N, 9°07'E; alt. 1400 m; 29 Jan. 1994 • M. Groves 122 (K, SCA, YA), trail north of Likombe Village; 4°14'N, 9°11'E; alt. 1000 m; 21 Feb. 1995 • M.R. Cheek 5145 (K, SCA, YA), Liwenyi, on the West bank of the Onge river above the first set of rapids, that is about 1–2 hours walk inland from Enyenge; 4°17'N, 8°58'E; alt. 50 m; 27 Oct. 1993 • P. Lane 142 (K, SCA, YA), Mt Kupé, within Permanent Sample Plot on Shrike Trail leading from Nyasoso to summit; 4°50'N, 9°40'E; alt. 1200 m; 20 Jun. 1994 • R.W.J. Keay FHI37485 (FHI, K), Kumba Distr., eastern boundary of Bambuko F.R., about 11 miles SSW. of Musome. Northern slopes of Cameroon Mt; 4°18'18.11'N, 9°11'55.75'E; alt. 1000 m; 01 Feb. 1958 • S. Cable 1353 (K, SCA, YA), path towards grassland and top of Mt Cameroon from Likombe; 4°07'N, 9°11'E; alt. 1060 m; 22 Feb. 1995 • S. Cable 1524 (K, SCA, YA), Upper Boando, logging path that becomes trail to summit; 4°04'N, 9°09'E; alt. 900 m; 14 Mar. 1995 • S. Cable 1626 (K, YA), path to summit of Etinde from Upper Boando; 4°04'N, 9°09'E; alt. 1000 m; 16 Mar. 1995 • S. Cable 2187 (K, YA); Ndian, Korup National Park, Ekundu Kundu, path from Ekundu-Kundu to about 1 km; 5°02'14.24'N, 8°54'58.14'E; alt. 300 m; 26 Apr. 1996 • T.D. Maitland 453 (K), Buea, above upper farm; 4°09'N, 9°14'E; alt. 1219 m; 1924 • T.D. Maitland s.n.10 (K), Cameroon mountain, Buea; 4°09'N, 9°14'E; alt. 975 m; 1930 • T.D. Maitland s.n.9 (K), Cameroon mountain, Buea area; 4°09'N, 9°14'E; alt. 1219 m; 1930 • T.L.P. Couvreur 1026 (WAG, YA), on trail leading to top of Mt Etinde, after Ekonjo village; 4°04'02.27'N, 9°09'10.16'E; alt. 749 m; 01 Apr. 2016 • T.L.P. Couvreur 1029 (MPU, WAG, YA), on trail leading to top of Mt Etinde, after Ekonjo village; 4°04'04.83'N, 9°09'11.43'E; alt. 781 m; 01 Apr. 2016 • T.L.P. Couvreur 1040 (MPU, WAG, YA), Mt Cameroon National Park, Bakinguili trail, above Bakinguili village; 4°05'48.05'N, 9°03'24.71'E; alt. 563 m; 02 Apr. 2016 • T.L.P. Couvreur 1046 (MPU, WAG, YA), Mt Cameroon National Park, on the Bomona trail, behind Bomona village, 10 km NW from Idenau; 4°17'46.46'N, 9°06'06.31'E; alt. 859 m; 03 Apr. 2016 • T.L.P. Couvreur 990 (MPU, WAG, YA), slopes of Mt Cameroon, on the Bokwango trail, near Bokwango village, 4 km south west of Buea; 4°07'25.26'N, 9°11'11.28'E; alt. 1227 m; 23 Mar. 2016 • T.L.P. Couvreur 992 (MPU, WAG, YA), slopes of Mt Cameroon, on the Bokwango trail, near Bokwango village, 4 km south west of Buea; 4°07'27.06'N, 9°10'13.93'E; alt. 1560 m; 23 Mar. 2016. Equatorial Guinea – Bioco (Fernando Poo) • G.W.J. Mildbraed 6428 (B), Pico Basilé, Fernando Poo: Nordseite d. Pics v. Sta. Isabel oberhalb Basilé, Wald über der Kakao-Region 6–800 m, viel Allanblackia, (oberer Tropenwald); 3°41'24.72'N, 8°51'27.72'E; alt. 800 m; 16 Aug. 1911 – Bioko Sur • W.R.Q. Luke 12203 (MA), Moeri: Camp1 to Camp2; 3°28'09.84'N, 8°40'06.96'E; alt. 704 m • W.R.Q. Luke 13279 (K), Hormiga Camp pt 346 to North camp pt 347; 3°20'51'N, 8°29'23.64'E; alt. 800 m; 01 Feb. 2009. Nigeria – Cross River State • K. Schmitt 323 (MO), Cross River National Park. Oban Hills. SW facing slope ca 5 km E of Neghe; 5°15'20“N, 8°38'50"E; 07 Feb. 1995.

#### ﻿ Uvariodendron fuscum var. giganteum

Taxon classificationPlantaeMagnolialesAnnonaceae

(R.E.Fr.) Dagallier & Couvreur, PhytoKeys 207: 423 (2022)

Figs 20 , 21 , 23 ; Table 4

≡ Uvaria gigantea Engl., Notizbl. Königl. Bot. Gart. Berlin 2: 292. 1899 (quoad specimens Zenker 108 and 698); Uvagigantea Kuntze, Deutsche Bot. Monatsschr. xxi. 173 (1903); Uvariodendrongiganteum (Engl.) R.E.Fr., Acta Horti Berg. 10: 62 (1930). Type. Cameroon – Central Region • G.A. Zenker 108 (lectotype: P! (P00362654), designated by Couvreur et al. (2022); isolectotype: COI! (COI00004926); lectotype designed by Fries (1930) as B, but sheet destroyed), Yaoundé, Yaunde; 3°52'N, 11°31'E; alt. 800 m; 1895.

##### Description.

Young branches with long soft hairs producing a whitish appearance quickly falling off. Petiole 8–35 mm long, 3.5–8 mm wide. Leaf lamina 357–676 mm long, 84–225 mm wide, length:width ratio 2.5–4, base acute to rounded, apex acute to acuminate, surface below pilose to glabrous when young, glabrous when old; midrib pilose to glabrous below; secondary veins 22–33 pairs. Flower pedicel 0–7.5 mm long. Bracts 1 to 6, upper bract 16–22 mm long, 20–50 mm wide. Sepals 20–30 mm long, 16–26 mm wide. Outer petals 25–40 mm long, 17–30 mm wide. Inner petals 21–40 mm long, 17–29 mm wide, length:width ratio 1.1–1.5, broadly obovate to obovate. Carpels 50 to 100. Fruiting pedicel 9–15 mm long. Monocarps 6 to 24, 20–50 mm long, 11–25 mm wide, length:width ratio 1.7–3.8, cylindrical, curved, acuminate, slightly constricted between the seeds, puberulent, green to brown. Seeds 8–14 per monocarp, ca. 14 mm long, ca. 7 mm wide.

##### Distribution.

Element of the Lower Guinean Domain and Congolia Domain of the Guineo-Congolian Region: Cameroon, Democratic Republic of the Congo, Gabon.

##### Habitat and ecology.

Lowland to submontane mature or old secondary rain forests, on inundated soils or along streams or rivers. Altitude: 100–1300 m a.s.l.

##### Notes.

Ud.fuscumvar.giganteum differs from the type variety in having young branches and petiole covered with long soft hairs producing a whitish appearance quickly falling off (vs. young branches and petiole sparsely pubescent to glabrous). Compared to the type variety, it has larger leaves (35.7–67.6 cm long and 8.4–22.5 cm wide, vs. 16–45 cm long and 4.3–11.8 cm wide), with generally more secondary veins (22 to 33 vs. 15 to 24), and flowers with generally greater sepals (20–30 mm long vs. 11–23 mm long) and more carpels (50 to 100 vs. 20 to 70) (Fig. 4, Table 4). Most of these characters overlap and without the young branches covered with long soft hairs it can be hard to place some specimens in the var. giganteum with certainty. The specimen Zenker 108, defined to be the type specimen by Fries (1930), was not found in B (lost or destroyed), so we made the duplicate from P as the lectotype and the duplicate from COI as the isolectotype (Couvreur et al 2022).

##### Additional specimens examined.

Cameroon – Central Region • G.A. Zenker 698 (B), Yaoundé, Yaunde; 3°52'N, 11°31'E; alt. 800 m; 04 Feb. 1895 • T.L.P. Couvreur 419 (MPU, WAG, YA), Mont Mbam Minkon, on trail, 5 km from Nkol Nyada village; 3°58'04.91'N, 11°24'08.64'E; alt. 1000 m; 21 Mar. 2013 – East Region • T.L.P. Couvreur 1206 (MPU, WAG, YA), 60 km south of Yokadouma, 30 km after Ngato, 15 km after river. ALPICAM ‘base de vie', then on forestry road starting 4 km before Maséa village; 3°09'55.18'N, 14°42'25.64'E; alt. 587 m; 05 Mar. 2019 • T.L.P. Couvreur 1229 (MPU, WAG, YA), 60 km south of Yokadouma, 30 km after Ngato, 15 km after river. ALPICAM ‘base de vie', then 40 km on forestry road starting 4 km before Maséa village. Coupe 4–4 of UFA 023; 3°05'08.17'N, 14°40'18.2'E; alt. 622 m; 08 Mar. 2019 – South Region • G.A. Zenker 1438 (L), Bipinde, Urwaldgebiet; 3°05'N, 10°25'E; 1898 • M. Biholong 279 (P, YA), près d'Alati. Ancienne piste Alati – Mintom II; 2°11'44.45'N, 13°24'17.61'E; 17 Jan. 1975 – South-West Region • C. Doumenge 473 (L, MO, P), Cameroon. Bakossi Mountains 1–8 Km NNE of Menyum Village; 5°01'N, 9°38'E; alt. 1000 m; 22 May. 1987 • P. Lane 501 (K, SCA, WAG, YA), Mt Kupe Division. Ndum. Forest trail 2 km south from Etube-Tape village; 4°51'N, 9°42'E; alt. 1200 m; 02 Feb. 1995 • T.L.P. Couvreur 1057 (MPU, WAG, YA), Nyasoso village, on max's trail to Mt Kupe; 4°49'27.88'N, 9°41'59.42'E; alt. 1227 m; 05 Apr. 2016 • T.L.P. Couvreur 512 (MPU, YA); Fako, on trail from Ekongo village, located 5 km before the entrance to Limbe, 7 km on secondary road. On flank of Mt Etinde. 100 m in Mont Cameroon National Park; 4°04'19.23'N, 9°08'00.76'E; alt. 959 m; 16 Oct. 2013 • T.R. van Andel 3761 (MO, SCA, U, WAG), Bokwango. Trail to Mann's spring; 4°07'44'N, 9°11'26'E; alt. 1130 m; 21 Jun. 2001. Democratic Republic of the Congo – Orientale • T.B. Hart 910 (WAG); Mambasa, Epulu, Zaire, Zone de Mambasa (Ituri); 1°25'N, 28°35'E; alt. 750 m; 23 Mar. 1989. Gabon – Estuaire • T.-J. Klaine 1690 (P), Environs de Libreville; 0°25'N, 9°27'E; 18 Oct. 1899 – Ogooué-Ivindo • N. Hallé 3156 (P), Bélinga; 1°05'N, 13°08'E; 12 Nov. 1964 • N. Hallé 430 (K, P), Bélinga, mines de fer, le long de la rivière Folley; 1°07'N, 13°11'E; 12 Aug. 1966 • N. Hallé 549 (K, P), Bélinga, mines de fer; 1°08'N, 13°12'E; alt. 700 m; 16 Aug. 1966.

#### ﻿ Uvariodendron fuscum var. magnificum

Taxon classificationPlantaeMagnolialesAnnonaceae

(Verdc.) Dagallier & Couvreur, comb. et stat. nov.

urn:lsid:ipni.org:names:77326968-1

Figs 22 , 23

≡ Uvariodendron magnificum Verdc. syn. nov., Kew Bull. 23(3): 515 (1969). Type. Uganda – Western Province • Okodi in Hamilton 696 (holotype: K! (K000198899, K000198900, K000198896, K000198897, K000198898); isotypes: ENT, MHU! (MHU000022, MHU000023, MHU000024)); Ankole District, Kashoya • Kitomi CFR; 0°13'N, 30°15'E; alt. 1050 m; 15 Jun. 1968.

##### Description.

Young branches with long soft hairs producing a whitish appearance quickly falling off. Petiole 6–20 mm long, ca. 5 mm wide. Leaf lamina 210–750 mm long, 57–215 (250) mm wide, length:width ratio 3–4, base acute to cuneate, apex rounded to acuminate, surface below pilose to glabrous when young, glabrous when old; midrib pilose to glabrous below; secondary veins 23–30 pairs. Flower pedicel 10–15 mm long. Flower not seen, description from Verdcourt (1969). Bracts 5 to 6, upper bract 8–35 mm long, 10–40 mm wide. Sepals 30–55 mm long, 30–43 mm wide. Outer petals 60–70 mm long, 43–47 mm wide. Inner petals 50–53 mm long, 36–38 mm wide, length:width ratio ca. 1.4, elliptic, imbricate at apex. Carpels 150 to 160. Fruiting pedicel 13–18 mm long. Monocarps 10 to 80, 28–60 mm long, 13–32 mm wide, length:width ratio 1.9–2.5, cylindrical, curved, acuminate, pubescent, brownish, fruits not seen, data from Verdcourt (1969). Seeds 1–16 per monocarp, 18 to 23 mm long, 10–11 mm wide.

##### Distribution.

Endemic to Zambezian Region. Known from only one locality in the Western Province in Uganda.

##### Habitat and ecology.

Montane forest. Altitude: around 1050 m a.s.l.

##### Notes.

Ud.fuscumvar.magnificum differs from the type variety in having young branches and petioles covered with long soft white hairs producing a whitish appearance quickly falling off (vs. young branches and petiole sparsely pubescent to glabrous). Compared to the type variety, it has larger leaves (21.–75 cm long and 5.7–21.5 (25) cm wide, vs. 16–45 cm long and 4.3–11.8 cm wide), with more secondary veins (23 to 30 vs. 15 to 24). It differs from the type variety and from the var. giganteum by its flowers having longer pedicels 10–15 mm long (vs. 0–7.5 mm long), larger sepals (30–55 mm long and 30–43 mm wide, vs. 11–30 mm long and 13–26 mm wide), larger petals (50–70 mm long and 36–47 mm wide, vs. 20–40 mm long and 15–30 mm wide) and more carpels (150 to 160 vs. 20 to 100) (Fig. 4, Table 4). This variety is known from two specimens collected more than 50 years ago in the Kasyoha-Kitomi Forest Reserve. Uganda’s forests have been reported to be degraded (Obua et al. 2010) so this variety might be threatened.

##### Additional specimens examined.

Uganda – Western Province • T. Synnott 197 (MHU); Ankole District, North border of Kitomi Forest; 0°07'29.07'N, 30°18'07.11'E; alt. 1070 m; 22 Oct. 1968.

#### ﻿ Uvariodendron gorgonis

Taxon classificationPlantaeMagnolialesAnnonaceae

Verdc., Kew Bull. 23(3): 512 (1969)

Figs 24 , 25 , 26

##### Type.

Kenya – Coast • B. Verdcourt 3940 (holotype: K! (K000198893), sheet here designated; isotype: BR! (BR0000008824325), EA! (EA000002458, EA000002460, EA000002459), K! (K000198894, K000198892, K000198895); Kwale District, Mrima hill (about halfway up road to Lungalunga from Mwambweni); 4°29'06.39'S, 39°15'47.19'E; alt. 182 m; 16 Jan. 1964.

##### Description.

Tree 3–27 m tall, D.B.H. 3–50 cm; young branches pubescent to glabrous, old branches glabrous; plant with lemon smell. Leaf bud ‘eragrostiform’, composed of 6–12, distichous, longitudinally folded, velutinous scales. Petiole 4–18 mm long, 1.5–5 mm wide, sparsely pubescent to glabrous. Leaf lamina 153–410 mm long, 36–127 mm wide, length:width ratio 2.3–4.3, elliptic to oblong to obovate, coriaceous, base generally acute to rounded, and minutely decurrent at the extreme base, apex generally acute to acuminate but can also appear rounded or retuse, acumen 0.5–12 mm long; surface above glabrous, surface below sparsely pubescent to glabrous when young, glabrous when old; midrib impressed above, raised below, glabrous above, sparsely pubescent to glabrous below; secondary veins 13–22 pairs, weakly brochidodromous, impressed above, raised below; tertiary veins reticulate. Inflorescences borne on trunk and branches, composed of 1–3 flowers. Flower pedicel 8–18 mm long, 3–8 mm in diameter, velutinous. Flowers bisexual, buds globose, sessile, 7–15 mm high, 9–14 mm in diameter, velutinous. Bracts 1 to 8, upper bract 3–12 mm long, 9–25 mm wide, broadly ovate, clasping the pedicel, enclosing the bud, velutinous outside, glabrous inside. Sepals 3, 8–13 mm long, 8–24 mm wide, imbricate to fused over up to ca. 30 % of their length, velutinous outside, glabrous inside, clear brown. Outer petals 3, 15–35 mm long, 13–23 mm wide, length:width ratio 1.1–2.2, broadly elliptic to elliptic, velutinous outside, glabrous inside, cream outside, cream with a dark purplish red streak from base up to 75% of the petal length inside. Inner petals 3, 15–40 mm long, 9–16 mm wide, length:width ratio 1.5–2.7, obovate, valvate at apex, velutinous outside, glabrous inside, cream to cream with a dark purplish red streak at base up to 15% of their length outside, dark purplish red with cream apex inside. Stamens 1400 to 1600, 2.5–3 mm long, 0.4–1 mm wide, anthers linear, connective prolongation truncate. Carpels 40 to 80, 3–4.5 mm long, 0.8–1.5 mm wide, pubescent, free; stigma 0.5 mm long, 0.8–1.1 mm wide, coiled, velutinous. Fruiting pedicel 10–20 mm long, 3–5 mm in diameter, velutinous. Monocarps 20 to 60, 24–90 mm long, 4.5–11 mm wide, length:width ratio 5–11, very narrowly cylindrical, torulose to torose, velutinous, greyish green; stipe 5–11 mm long, 1.5–3 mm wide, velutinous. Seeds 3–14 per monocarp, uniseriate, 3 to 9 mm long, 2–7 mm wide, ellipsoid.

**Figure 24.**
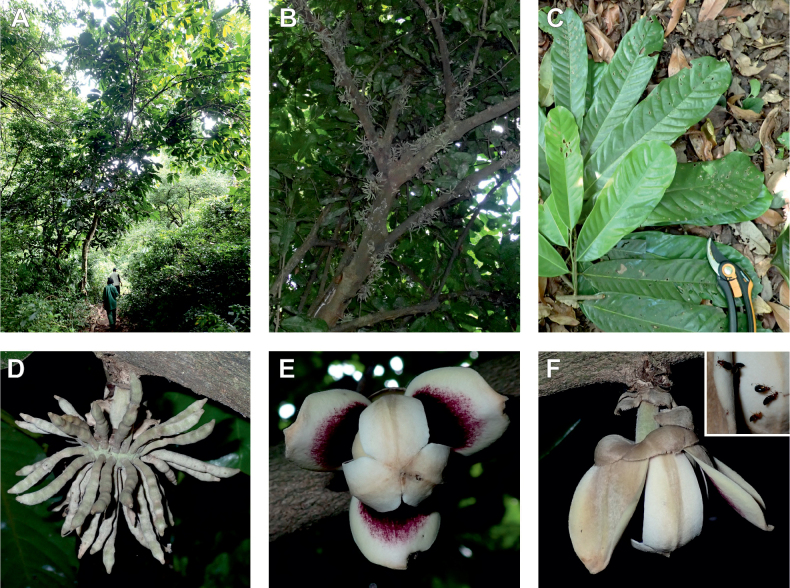
*Uvariodendrongorgonis* Verdc **A** habit **B** trunk and branches, note the young fruits all along the branches and trunk **C** young branches and leaves, upper side **D** young fruit, side view **E** flower, bottom view **F** flower, side view, inset: Coleoptera larvae frequently found in the flower. **A, B** no specimen associated **C** Dagallier 52 **D, E** Dagallier 38. Photos Léo-Paul Dagallier.

##### Distribution.

Endemic to Somalia-Masai Region: Kenya, Mozambique and Tanzania.

##### Habitat and ecology.

Lowland to submontane secondary rain forests or semi-deciduous forest. Soil: coral rag or igneous intrusion. Altitude: 170–950 m a.s.l.

##### Phenology.

Flowers collected from January to February and from July to November. Fruits collected from January to March and from June to November.

##### Vernacular names.

Tanzania: ‘Mtwamu’ (Mbago 1616).

##### Etymology.

Although Verdcourt (1969) did not mention it in the protologue, the specific epithet might come from the Latin *gorgonis* (derived from the Ancient Greek *gorgo*). This refers to the gorgons, three creatures from the Greek mythology, depicted to have hairs made of living snakes. The name of *Ud.gorgonis* might thus come from their fruits formed of long and numerous monocarps, resembling a gorgon’s head.

**Figure 25.**
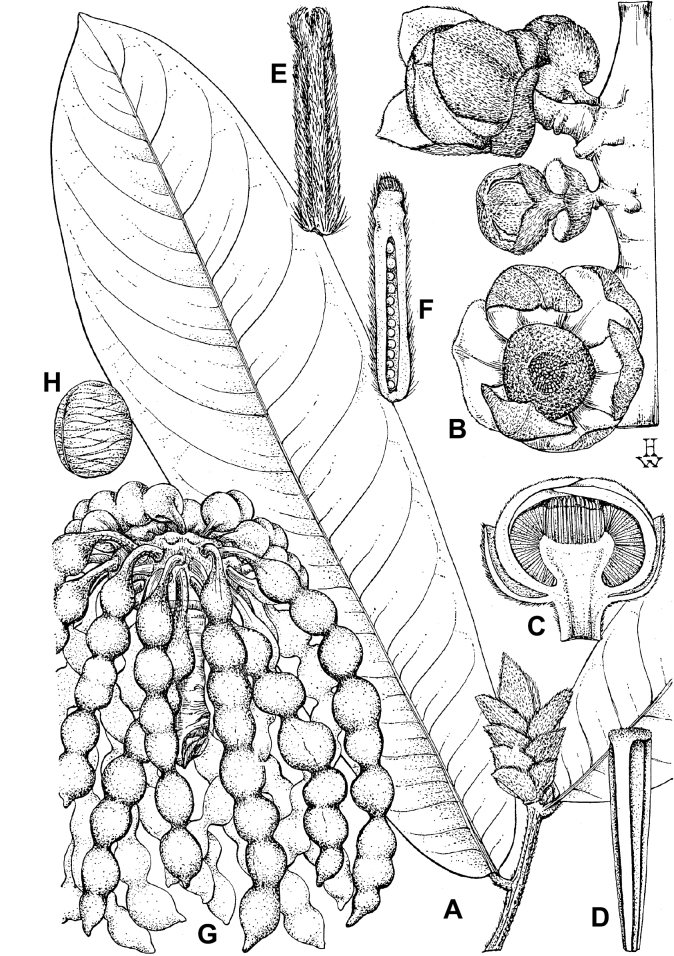
*Uvariodendrongorgonis* Verdc **A** young branch and apical ‘eragrostiform’ bud **B** flower, top view, and flower buds, side view **C** longitudinal section of flower bud **D** stamen, side view **E** carpel, side view **F** longitudinal section of carpel **G** fruit, side view **H** seed, side view. **A** from Verdcourt 1890 **B–G** from Drummond 1954 **H** from Verdcourt 3940 (type). Drawings by Heather Wood, from Verdcourt (1969; fig. 1, p. 514), Kew Bulletin 1969, © Board of Trustees of the Royal Botanic Gardens, Kew.

##### Notes.

This species differs from all other species by its fruits, unique in the genus. The fruits of *Ud.gorgonis* are composed of 20 to 60 monocarps, while most of the *Uvariodendron* species have fruits composed of 1 to 20 monocarps (except *Ud.calophyllum* and Ud.fuscumvar.giganteum that have fruits composed of up to 35 monocarps). Moreover, the monocarps are 5 to 10 times longer than wide (vs. 1.3 to 4.5 times longer than wide in the other species). They are torulose to torose, i.e. very strongly constricted between the seeds. The ovules (and seeds) are uniseriate. The combination of these characters confers the fruits the aspect of a hairy head.

**Figure 26.**
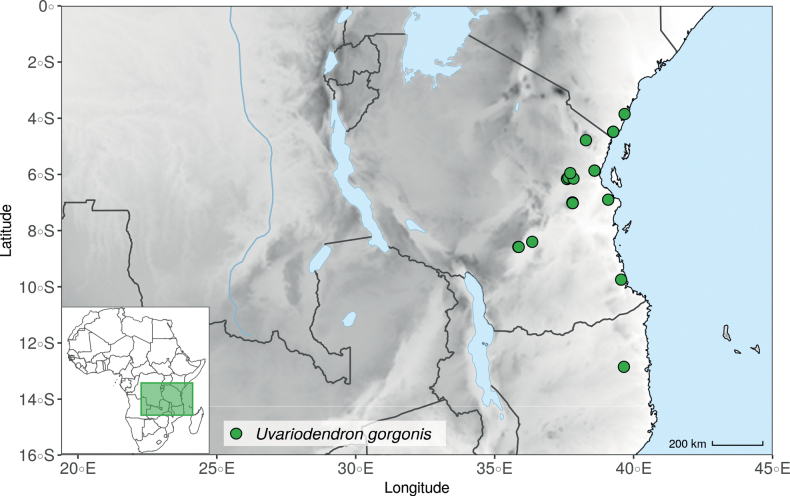
Distribution map of *Uvariodendrongorgonis*. Shades of grey represent elevation, from white (sea level) to darker grey (higher elevation). The inset shows the extent of the map over Africa.

Vegetatively, this species is harder to differentiate as its leaves have dimensions that overlap with most of the other species. However, the bases of the leaves are quite peculiar: they appear generally acute to rounded but a closer look actually detects them as minutely decurrent at the extreme base. Similarly, the apices of the leaves are generally acute to acuminate, but can appear rounded to slightly retuse with a minute acumen.

The fresh leaves of *Ud.gorgonis* also present a citrus smell when crushed, as in *Ud.angustifolium*, *Ud.citriodorum* and *Uvariopsiscitrata*.

##### Preliminary conservation status.

A previous assessment, that needs updating, listed this species as Endangered EN under criteria B2ab(iii), based on its occurrence in eight locations in Tanzania (Eastern Arc Mountains & Coastal Forests CEPF Plant Assessment Project 2009a). Here, the EOO of this species is estimated at 203,044 km^2^ and its AOO at 72 km^2^. It occurs in 11 locations in Tanzania, Kenya and Mozambique. Given its habitat is severely fragmented and there is continuing decline due to habitat loss and degradation (Eastern Arc Mountains & Coastal Forests CEPF Plant Assessment Project 2009a), it qualifies for the same previous status of Endangered EN under criteria B2ab(iii).

##### Additional specimens examined.

Kenya – Coast • B. Verdcourt 1890 (K); Kwale District, Mrima Forest, Mrima Hill; 4°29'06.39'S, 39°15'47.19'E; 06 Sep. 1957 • B. Verdcourt 1911 (B, EA, K); Kwale District, Mrima Forest, Mrima Hill; 4°29'06.39'S, 39°15'47.19'E; 07 Sep. 1951 • J.P.M. Brenan 14601 (EA, K, P); Kilifi District, K7, Kilifi District. Pangani ‘Kaya' Forest; 3°51'S, 39°40'30'E; alt. 170 m; 19 Nov. 1978 • R.B. Faden 70/253 (K); Kwale District, Mrima Hill, about halfway betwee Mgambweni and Lungalunga; 4°29'S, 39°16'E; alt. 70 m; 25 Jun. 1970 • S.A. Robertson MDE78 (K); Kwale District, Mrima Hill; 4°29'S, 39°16'E; alt. 270 m; 04 Feb. 1989. Mozambique – Cabo Delgado • W.R.Q. Luke 9983 (MO), Quirimba NP, Ngura Inselberg; 12°51'S, 39°39'E; alt. 420 m; 10 Dec. 2003. Tanzania – Lindi • F.M. Mbago 2148 (K), Ruawa Forest; 9°44'46.39'S, 39°33'04.77'E; alt. 250 m; 01 Sep. 2001 – Morogoro • A. Pócs 6061B (DSM); Morogoro Rural District, Kimboza Forest Reserve, between Mkuyuni and Matombo; 7°00'S, 37°48'E; alt. 300 m; 19 Nov. 1969 • A. Pócs 6280F (DSM); Morogoro Rural District, Kimboza Forest Reserve; 7°00'S, 37°48'E; alt. 300 m; 05 Nov. 1970 • B.J. Harris 3223 (DSM, EA); Morogoro Rural District, Kimboza Forest Reserve, KFR near Matombo, Uluguru Mts; 7°00'S, 37°48'E; 06 Sep. 1969 • C.D. Mgaza 323 (K), Luzunguru forest reserve; 6°05'S, 37°40'E; 19 Sep. 1959 • F.C. Magogo 2171 (K); Morogoro Rural District, Kimboza Forest Reserve, Kibungo Forest Reserve (Kimboza); 7°00'S, 37°48'E; alt. 600 m; 31 Aug. 1981 • F.M. Mbago 1616 (DSM); Kilombero District, Lower Kihansi Hydropower Project; 8°24'S, 36°21'E; 30 Aug. 1997 • F.M. Mbago 1734 (K), T7: Udzungwa scarp, Upper Kihansi Gorge, forest opposite the spray zone; 8°34'54.35'S, 35°51'05.9'E; alt. 950 m; 15 Jul. 1998 • L.-P.M.J. Dagallier 47 (DSM, MPU, P, WAG); Morogoro Rural District, Kimboza forest; 7°01'16.37'S, 37°48'17.38'E; alt. 278 m; 15 Nov. 2019 • L.-P.M.J. Dagallier 52 (DSM, K, MPU, P, WAG); Morogoro Rural District, Kimboza forest; 7°01'18.37'S, 37°48'31.63'E; alt. 262 m; 15 Nov. 2019 • L.-P.M.J. Dagallier 57 (DSM, MPU, P, WAG); Mvomero District, Turiani village; 6°09'36.37'S, 37°36'17.72'E; alt. 377 m; 18 Nov. 2019 • L.-P.M.J. Dagallier 61 (DSM, MPU, P, WAG); Mvomero District, Turiani village; 6°09'46.37'S, 37°36'12.66'E; alt. 373 m; 18 Nov. 2019 • L.B. Mwasumbi 19144 (MO); Kilombero District, Kihansi gorge; 8°35'S, 35°51'50'E; alt. 800 m; 04 Sep. 1998 • R.B. Drummond 1954 (K); Morogoro Rural District, 6.4 km N of Turiani, Lusunguru Forest Reserve, near Mtibwa sawmill; 6°05'S, 37°41'E; 31 Mar. 1953 • S. Paulo 163 (EA, K); Morogoro Rural District, Kimboza Forest Reserve; 7°00'S, 37°48'E; Nov. 1953 • S.R. Semsei 1498 (K), Mtibwa Forest Reserve; 6°08'56.37'S, 37°50'10.32'E; Nov. 1953 • S.R. Semsei 773 (K); Morogoro Rural District, Kimboza Forest Reserve; 7°00'S, 37°48'E; Jul. 1952 • S.R. Semsei 852 (K); Morogoro Rural District, Mtibwa Forest Reserve; 6°07'S, 37°39'E; Aug. 1952 • T.L.P. Couvreur 69 (DSM, MO, WAG); Kilombero District, Kimboza Forest reserve, 2 km after Kimboza village, 45 km from Morogoro; 7°01'19.37'S, 37°48'16.8'E; alt. 250 m; 25 Nov. 2006 • Y.S. Abeid 2651 (EA, MO); Mvomero District, Kanga Forest Reserve, near Kwa Beku subvillage; 5°57'15'S, 37°43'11.4'E; alt. 500 m; 11 Mar. 2006 – Pwani • L.B. Mwasumbi 113377 (DSM); Kisarawe District, Pugu hills Forest Reserve; 6°54'S, 39°05'E; 28 May. 2002 – Tanga • F.M. Mbago 3756 (DSM); Handeni District, T3: Handeni Kwansisi Mgulwi stream; 5°51'55.38'S, 38°35'39.34'E; alt. 203 m; 16 Aug. 2016 • G.A. Peter 13872 (B, K, WAG); Lushoto District, O. Usambara, Mashewa; 4°47'S, 38°17'E; alt. 400 m; Sep. 1915 • L.-P.M.J. Dagallier 38 (BR, DSM, K, MO, MPU, P, WAG); Handeni District, Kwamsisi village; 5°51'50.38'S, 38°35'31.77'E; alt. 196 m; 13 Nov. 2019.

#### ﻿ Uvariodendron kimbozaense

Taxon classificationPlantaeMagnolialesAnnonaceae

Dagallier & Couvreur sp. nov.

urn:lsid:ipni.org:names:77326969-1

**Figure 27.**
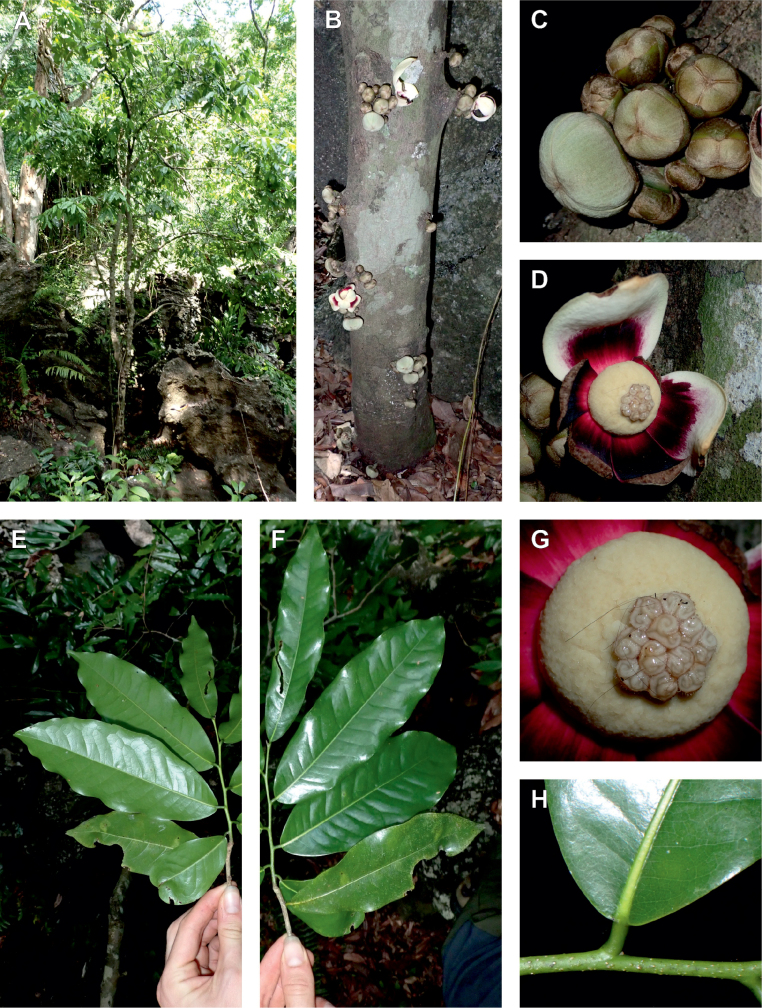
*Uvariodendronkimbozaense* Dagallier & Couvreur **A** habit **B** trunk with borne inflorescences **C** young inflorescence with flower buds **D** flower with one outer petal and the 3 inner petals torn or gnawed **E** young branch with leaves, lower side **F** young branch with leaves, upper side **G** detail of flower receptacle with stamens and stigmas, note the gleaming exudate on the stigmas and the hairs stuck on them **H** base of leaf, upper side, note the slightly raised midrib. **A–H** Dagallier 49 (type). Photos Léo-Paul Dagallier.

**Figure 28.**
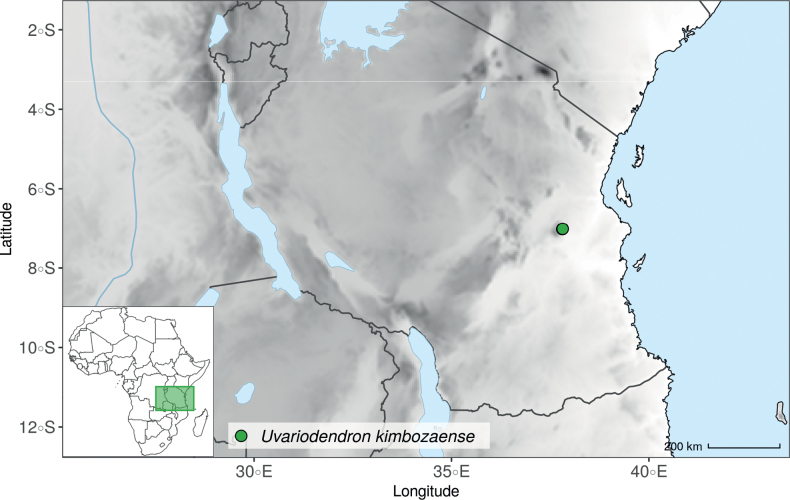
Distribution map of *Uvariodendronkimbozaense*. Shades of grey represent elevation, from white (sea level) to darker grey (higher elevation). The inset shows the extent of the map over Africa.

Figs 3A, C, E, G , 27 , 28 ; Table 2

##### Type.

Tanzania – Morogoro • L.-P.M.J. Dagallier 49 (holotype: MPU! (MPU1379108); isotypes: DSM!, MPU! (MPU1375359), P! (P00948153); also distributed to K and WAG); Morogoro Rural District, Kimboza forest; 7°01'16.37'S, 37°48'35.24'E; alt. 279 m; 15 Nov. 2019.

##### Diagnosis.

*Uvariodendronkimbozaense* resembles *Ud.kirkii* by the elliptic leaves and petal colors, but is distinguished from this species by having slightly greater leaves (140–220 mm, vs. 70–190 mm long in *Ud.kirkii*) with a base acute to rounded (vs. acute to decurrent). Compared to *Ud.kirkii*, *Ud.kimbozaense* also has more bracts on the flower pedicel (1 to 4, vs. 1 maximum), greater (6–12 mm long, vs. 3–6.5 mm long) and imbricate (vs. connivent) sepals, and greater (16–39 mm long, vs. 10–20 mm long), elliptic (vs. ovate), and almost flat petals (with a slight transversal curvature vs. “boat-shaped” petals, with a strong transversal curvature) (Fig. 3, Table 2). *Ud.kimbozaense* is also distinguished from all other *Uvariodendron* species by the leaves having a midrib slightly raised above with a central depression all along the length of the midrib (Figs 3G, 27H).

##### Description.

Tree 5–7 m tall, D.B.H. 15–20 cm; young branches sparsely pubescent to glabrous, old branches glabrous. Petiole 4–5 mm long, 1.5–2 mm wide, glabrous. Leaf lamina 147–215 mm long, 44–68 mm wide, length:width ratio 2.5–3.6, elliptic to oblong, coriaceous, base acute to rounded, apex attenuate, surface above glabrous, surface below glabrous; midrib slightly raised with a central groove all along above, raised below, glabrous above, glabrous below; secondary veins 12–17 pairs, weakly brochidodromous, impressed above, raised below; tertiary veins reticulate. Inflorescences borne on growth of the trunk and on old branches, composed of 1–11 flowers. Flower pedicel 10–14 mm long, 2–3 mm in diameter, pubescent. Flowers bisexual, buds globose to oblate, pedicellate, 5–10 mm high, 7–16 mm in diameter, pubescent. Bracts 1 at base and from 1 to 4 along the pedicel, upper bract 3–6 mm long, 6–14 mm wide, broadly ovate, adpressed, clasping the pedicel, pubescent outside, glabrous inside. Sepals 3, 6–12 mm long, 12–21 mm wide, depressed ovate, imbricate, pubescent outside, glabrous inside, green to brownish-green. Outer petals 3, 16–28 mm long, 13–19 mm wide, length:width ratio 1.2–1.8, broadly elliptic to elliptic, puberulent outside, puberulent at apex to glabrous inside, cream outside, cream with a purple-red to reddish black streak from base up to 50% of the petal length inside. Inner petals 3, 18–39 mm long, 9–18 mm wide, length:width ratio 2–2.2, obovate, slightly transversally curved, puberulent outside, glabrous inside, cream to cream with a slight purplish red streak at base outside, purple-red at base to reddish black toward the apex inside. Stamens 600 to 800, 2–3.5 mm long, 0.5–0.7 mm wide, anthers linear, connective prolongation truncate. Carpels 11 to 16, 4–5 mm long, 1.8–2 mm wide, densely pubescent, free; stigma 1–1.5 mm long, 1.5–2 mm wide, coiled, densely pubescent, covered with an exudate at anthesis. Fruits unknown.

##### Distribution.

Endemic to Somalia-Masai Region. Known from only one locality in Tanzania: the Kimboza Forest Reserve.

##### Habitat and ecology.

Lowland mature rain forest on coral rag and limestone rocks. Altitude: 250–450 m a.s.l.

##### Phenology.

Flowers collected in March and November.

##### Etymology.

The specific epithet comes from Kimboza Forest Reserve (Tanzania), from where the species is endemic.

##### Notes.

*Ud.kimbozaense* has been previously identified as *Ud.kirkii* in all the specimens examined. However, it clearly differs from *Ud.kirkii* by the characters presented in the diagnosis above (see Fig. 3 and Table 2). The leaves have a slightly raised midrib with a central groove all along above (Figs 3G, 27H). It is a character unique in the genus *Uvariodendron*, but it is common in the genus *Crematosperma* (Pirie et al. 2018). Simply raised midribs occur in other genera such as *Isolona*, *Monodora* and *Ophrypetalum* (Couvreur 2009).

##### Preliminary conservation status.

This species is only known from the Kimboza Forest Reserve in Tanzania. This protected area has been threatened by encroachment, logging and invasion by the exotic *Cedrelaodorata* L. (Hall and Rodgers 1986; Patrick 2008). The surface of the Kimboza Forest Reserve is 3.85 km^2^ (https://www.protectedplanet.net/7520), and we thus estimate the EOO and AOO to be less than 4 km^2^. Following IUCN criterion B, this species is assigned a preliminary status of Critically Endangered CR B1ab(iii)+2ab(iii).

##### Additional specimens examined.

Tanzania – Morogoro • C.J. Kayombo 5357 (MO); Morogoro Rural District, Kimboza Forest Reserve; 7°00'43'S, 37°48'50'E; alt. 400 m; 21 Mar. 2006 • T.L.P. Couvreur 71 (DSM, MO, WAG); Kilombero District, Kimboza Forest reserve, 2 km after Kimboza village, 45 km from Morogoro; 7°01'19.37'S, 37°48'16.8'E; alt. 250 m; 25 Nov. 2006 • W.R.Q. Luke 766 (EA), Uluguru Mts nr Ruvu bridge; 7°01'S, 37°49'E; alt. 450 m; 04 Nov. 1987.

#### ﻿ Uvariodendron kirkii

Taxon classificationPlantaeMagnolialesAnnonaceae

Verdc., Kew Bull. 23(3): 518 (1969)

**Figure 29.**
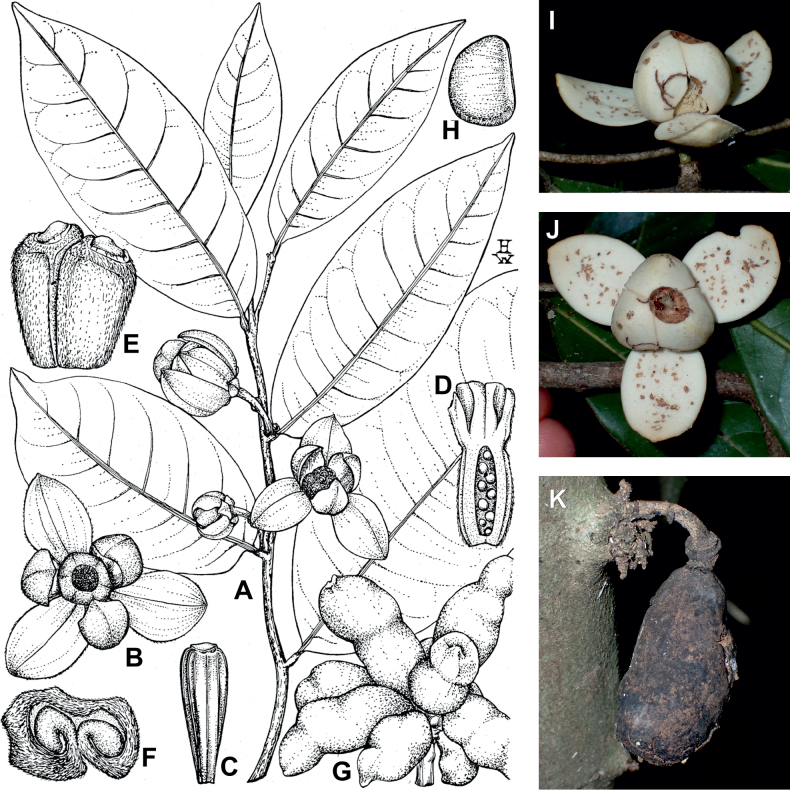
*Uvariodendronkirkii* Verdc **A** flowering branch **B** flower, top view **C** stamen **D** longitudinal section of carpel **E** carpel, semi-side view **F** carpel, top view **G** fruit, side view **H** seed, side view **I** flower, side view **J** flower, top view **K** old fruit with **A** single rotten monocarp. **A** from Verdcourt 3939 (type) **B–F** from Drummond 4029 **G, H** from Drummond 3983 **I, J** Dagallier 23 **K** Dagallier 60. Drawings by Heather Wood, from Verdcourt (1969; fig. 3, p. 519), Kew Bulletin 1969, © Board of Trustees of the Royal Botanic Gardens, Kew. Photos Léo-Paul Dagallier.

**Figure 30.**
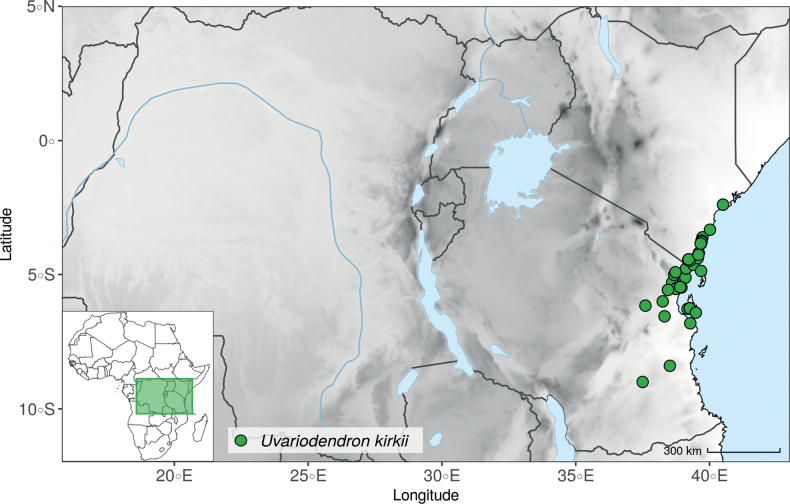
Distribution map of *Uvariodendronkirkii*. Shades of grey represent elevation, from white (sea level) to darker grey (higher elevation). The inset shows the extent of the map over Africa.

Figs 3B, D, F, H , 29 , 30 ; Table 2

##### Type.

Kenya – Coast • B. Verdcourt 3939 (holotype: K! (K000198893), sheet here designated; isotypes: BR! (BR0000008824325), EA!, K! (K000198894, K000198895)); Kwale District, Mrima hill (about halfway up road to Lungalunga from Mwambweni); 4°29'06.39'S, 39°15'47.19'E; alt. 182 m; 16 Jan. 1964.

##### Description.

Shrub to tree 1.5–6 (10–15) m tall, D.B.H. 3–20 cm; young branches sparsely pubescent to glabrous, old branches glabrous. Petiole 2.5–8 mm long, 1–2 mm wide, glabrous. Leaf lamina 70–188 mm long, 22–75 mm wide, length:width ratio 2.1–3.5, elliptic, coriaceous, base acute to decurrent, apex attenuate, surface above glabrous, surface below glabrous; midrib slightly impressed above, raised below, glabrous above, glabrous below; secondary veins 8–16 pairs, weakly brochidodromous, impressed above, raised below; tertiary veins reticulate. Inflorescences mostly axillary or borne on old branches, sometimes borne on trunk, composed of 1–2 flowers. Flower pedicel 5–28 mm long, 1–4 mm in diameter, pubescent. Flowers bisexual, buds globose, pedicellate, 2–5.5 mm high, 1.3–6 mm in diameter, pubescent. Bracts 1 at base and sometimes 1 on the upper half of the pedicel, upper bract 1–5 mm long, 1–8 mm wide, broadly ovate, adpressed, semi-clasping the pedicel, pubescent outside, glabrous inside. Sepals 3, 3–6.5 mm long, 4–8 mm wide, very broadly to broadly ovate, connivent, pubescent outside, glabrous inside, green to brownish-green. Outer petals 3, 10–17 mm long, 6–13 mm wide, length:width ratio 1.2–1.7, broadly ovate to ovate, pubescent to puberulent outside, puberulent to glabrous inside, cream to greenish pale yellow outside, cream to cream with a purple streak from base up to 50% of the petal length inside. Inner petals 3, 12–20 mm long, 7–8 mm wide, length:width ratio 1.3–1.9, obovate, strongly transversally curved (“boat-shape”), valvate at apex, puberulent outside, glabrous inside, white to cream outside, cream to cream with purple streak at base inside. Stamens 1000 to 1200, 1.5–1.8 mm long, 0.2–0.3 mm wide, anthers linear, connective prolongation truncate. Carpels 8 to 16 (20), 2–3 mm long, 1–1.5 mm wide, pubescent, free; stigma 0–1.5 mm long, 1–1.3 mm wide, coiled, pubescent. Fruiting pedicel 7–22 mm long, 2–4 mm in diameter, pubescent. Monocarps 1 to 6, 23–36 mm long, 10–16 mm wide, length:width ratio 1.4–2.4, cylindrical, longitudinally ridged, slightly constricted between the seeds, minutely acuminate, puberulent, green to dull orange to greyish dark blue; sessile to shortly stipitate, stipe 0–3 mm long, 2–5 mm wide, pubescent. Seeds 12–15 per monocarp, biseriate, 11 to 15 mm long, 8–10 mm wide.

##### Distribution.

Endemic to Somalia-Masai Region: Kenya and Tanzania.

##### Habitat and ecology.

Lowland mature or secondary rain forest, dry forest or thicket on coral rag and limestone rocks, or igneous intrusions. Altitude: 10–80 m a.s.l.

##### Phenology.

Flowers and fruits collected all year.

##### Vernacular names.

Tanzania: ‘Kisambaa’ in Mkomboa (Mwangoka 1356), ‘Mdumi’ in Zigua (Tanner 3467).

##### Notes.

This species is quite variable morphologically with leaves from 70 mm to almost 200 mm long. It can be distinguished from the other species with its pedicelate flowers growing mainly on leafy branches and sometimes on the trunk. The pedicel is lower than in *Ud.anisatum* (5–28 mm vs. 15–65 mm long in *Ud.anisatum*). At anthesis, the flowers generally have their outer petals completely spread out and their inner petals connivent at apex (Fig. 29I–J). At anthesis, the inner petals are “boat shaped”, that is with a strong transversal curvature (this character is not detectable on flower buds and on dried specimens). Some of the oldest specimens of this species were collected by Gustav Albert Peter under the name “*Uvariodendroncrassum* (Peter)” (e.g. Peter 4487, 24366 in B). Peter died in 1937, before achieving all the volumes of the Flora von Deutsch-Ostafrika, thus not publishing this species. In parallel, Verdcourt described and named this species in 1969, before seeing Peter’s specimens (see Verdcourt’s note on specimen Peter 24366 in B).

##### Preliminary conservation status.

A previous assessment, which needs updating, listed this species as Vulnerable VU under criteria B1ab(iii) (Eastern Arc Mountains & Coastal Forests CEPF Plant Assessment Project 2009b). Here, the EOO of this species is estimated at 67,203 km^2^ and its AOO at 152 km^2^. It occurs in more than 10 locations in Tanzania and Kenya and is fairly common but its population is severely fragmented and there is continuing decline due to habitat loss (Eastern Arc Mountains & Coastal Forests CEPF Plant Assessment Project 2009a). We thus assign a preliminary updated conservation status of Endangered EN B2ab(iii).

##### Additional specimens examined.

Kenya – Coast • B. Verdcourt 1891 (K); Kwale District, Mrima Forest, Mrima Hill; 4°29'06.39'S, 39°15'47.19'E; 06 Sep. 1957 • B. Verdcourt 5280 (K); Kwale District, Jombo Mt (near bottom); 4°26'08.39'S, 39°12'42.61'E; 11 Apr. 1978 • F.C. Magogo 768 (K); Kwale District, Shimba Hills, Dzombo Mountain; 4°26'09.39'S, 39°12'46.1'E; alt. 305 m; 08 Apr. 1968 • I.R. Dale 3836 (K), Witu Forest Reserve, Gongoni Forest, Witu; 2°23'07'S, 40°30'09'E; Oct. 1937 • J.B. Gillett 19901 (K); Kwale District, Diani forest. Areas NW & NE within 1 km N of turn off from new road for Jadini hotel, W & E of this road. Areas SW & SE within 1.5 km s of this point; 4°19'S, 39°33'E; alt. 12 m; 11 Jul. 1972 • J.B. Gillett 19906 (EA, K); Kwale District, Diani forest. Areas NW & NE within 1 km N of turn off from new road for Jadini hotel, W & E of this road. Areas SW & SE within 1.5 km s of this point; 4°19'S, 39°33'E; alt. 12 m; 11 Jul. 1972 • J.B. Gillett 24009 (B, EA); Kwale District, Kenya: K7: Kwale District Waa Kaya forest near coast N. of Tiwi ruined Mosque; 4°12'S, 39°36'E; alt. 30 m; 30 Dec. 1982 • J.P.M. Brenan 14592 (K); Kilifi District, K7, Kilifi District. Pangani ‘Kaya' Forest; 3°51'S, 39°40'50'E; alt. 170 m; 19 Nov. 1978 • L.J. Lap 112 (MO, WAG); Kilifi District, Kaya Kauma. Mapsheet K198/1. Along the path extending westwards from the Kaya cemetery. Western hill slope forest of Kaya Kauma; 3°37'S, 39°44'E; 22 Dec. 1981 • M.G. Gilbert 4967 (EA, K, WAG), Kwale District. Jombo Hill; 4°28'S, 39°12'E; alt. 300 m; 07 Jan. 1978 • M.G. Gilbert 5334 (K); Kilifi District, Cha Simba rocks on Mariakani – Kilifi Road; 3°44'S, 39°42'E; alt. 200 m; 16 Feb. 1979 • N. Mwadime 204 (EA); Kwale District, Shimoni, mwamba-wanga area; 4°38'30.39'S, 39°24'12.97'E; alt. 2 m; 13 Dec. 2012 • R.B. Drummond 3983 (K); Kwale District, Jardini Beach, 18 miles south of Mombasa, sea level; 4°19'30'S, 39°34'20'E; 26 Aug. 1953 • R.B. Drummond 4029 (K, U); Kwale District, Mwasangombe Forest, 15 miles S.W. of Kwale; alt. 230 m; 27 Aug. 1953 • R.B. Faden 77/397 (K); Kwale District, Kinondo (Ngalani Kaya) Forest; 4°23'30'S, 39°33'30'E; alt. 5 m; 15 Feb. 1977 • R.B. Faden 77/437 (K); Kilifi District, just north of Mwarakaya on Chonyi – Ribe road; 3°47'S, 39°42'E; alt. 140 m; 16 Feb. 1977 • S. Rawlins EAH11275 (K), Witu; 2°23'05.40'S, 40°30'04.68'E; alt. 24 m; Jun. 1957 • S.A. Robertson 5929 (K); Kwale District, Kaya Diani; 4°16'S, 39°35'E; alt. 10 m; 10 Nov. 1989 • S.A. Robertson 6465 (EA); Kilifi District, Watamu, Ashe plot; 3°20'S, 40°01'E; alt. 10 m; 28 May. 1991 • S.A. Robertson 7550 (EA, K, WAG); Kilifi District, Kambe Rocks; 3°51'S, 39°40'E; alt. 120 m; 13 May. 2005 • S.A. Robertson MDE293 (EA, K, WAG), Kwale Distr., Dzombo Hill; 4°26'S, 39°13'E; alt. 400 m; 09 Feb. 1989 • S.A. Robertson MDE46 (K); Kwale District, Mrima hill; 4°29'S, 39°16'E; 03 Feb. 1989. Tanzania – Dar es Salaam • J. Kirk s.n (K), Dar es Salaam; 6°48'57.39'S, 39°16'49.44'E; Mar. 1860 – Kaskazini Pemba (North Pemba) • P.J. Greenway 2681 (K); Micheweni District, Ras Kigomasha; 4°52'S, 39°41'E; 10 Dec. 1930 – Lindi • K.B. Vollesen 3150 (EA), c. 7 km NWN of Kingupina; 8°24'S, 38°31'E; alt. 125 m; 25 Dec. 1975 • R.I. Ludanga 1369 (EA), Selous, Likandage (Utunge); 9°00'S, 37°30'E; 28 Jan. 1972 – Morogoro • L.-P.M.J. Dagallier 60 (DSM, MPU, P, WAG); Mvomero District, Turiani village; 6°09'40.37'S, 37°36'14.48'E; alt. 384 m; 18 Nov. 2019 – Pwani • B.J. Harris 6209 (EA), Approx 6 mls north of Chalinze on road D'Salaam-Chalinze-Segera; 6°33'23.38'S, 38°19'08.14'E; alt. 274 m; 04 Apr. 1972 • G.A. Peter 24366 (B, K), Useguha; Aug. 1918 – Tanga • G.A. Peter 40249 (B), Usaguha: Hale in Pangani; 5°18'S, 38°36'E; alt. 330 m; 14 May. 1926 • G.A. Peter 52280 (B); Usambara, E Usambara; 5°00'45.38'S, 38°40'43.59'E; 05 Aug. 1915 • H.G. Faulkner 11 (B, K); Tanga District, Sawa Creek; 5°07'S, 39°06'E; 17 Feb. 1957 • J. Procter 3592 (K); Pangani District, Msumbugwe Forest Reserve; 5°30'15.38'S, 38°45'23.76'E; Apr. 1967 • L.-P.M.J. Dagallier 23 (DSM, MPU, P, WAG), Kilulu Hill, 50 km North of Tanga; 4°46'21.39'S, 39°07'33.48'E; alt. 247 m; 08 Nov. 2019 • L.-P.M.J. Dagallier 8 (DSM, K, MPU, P, WAG), forest patch near Msangazi river, along the road from Chalinze to Korogwe-Tanga; 5°34'16.38'S, 38°26'47.01'E; alt. 306 m; 06 Nov. 2019 • L.-P.M.J. Dagallier 9 (DSM, MPU, P, WAG), Kilulu Hill, 50 km North of Tanga; 4°46'23.39'S, 39°07'29.07'E; alt. 263 m; 07 Nov. 2019 • M.A. Mwangoka 1356 (MO); Muheza District, Kuze Kibago village. NW part of Kwemnyese public forest patch; 4°54'51'S, 38°43'35'E; alt. 100 m; 01 Jun. 2000 • R.E.S. Tanner 12 (K); Pangani District, Msubugwe Forest; 5°31'59.38'S, 38°43'59.88'E; alt. 100 m; 1955 • R.E.S. Tanner 29 (K); Pangani District, Msubugwe Forest; 5°31'59.38'S, 38°43'59.88'E; alt. 100 m; 1955 • R.E.S. Tanner 3467 (B, K); Pangani District, Mwera, Kwa Besa, Mwanamgaru; 5°29'S, 38°57'E; 28 Mar. 1957 • R.E.S. Tanner 6 (K); Pangani District, Msubugwe Forest; 5°31'59.38'S, 38°43'59.88'E; alt. 100 m; 1955 • T.L.P. Couvreur 34 (DSM, FHO, MO, NHT, WAG); Handeni District, road 3 km before arriving to Kwedikwazu coming from Dar Es Salaam. At Msangasi river; 5°34'21.38'S, 38°26'46.8'E; alt. 300 m; 14 Nov. 2006 • W.D. Hawthorne 776 (K), Nr Langoni village; 5°28'S, 38°54'E; 27 May. 1982 – Zanzibar Central/South • J. Vaughan 2318 (BM), Kufile cave; 6°25'08.39'S, 39°29'15.85'E; 23 Feb. 1936 – Zanzibar West • D. Aplin 1 (K), Zanzibar: Chumbe I; 6°16'44'S, 39°10'37'E; alt. 5 m; Feb. 1988 • J. Vaughan 1689 (K), Kombeni caves; 6°15'39.39'S, 39°15'39.95'E; 22 Nov. 1930 • J. Vaughan 1715 (K), Kombeni caves; 6°15'39.39'S, 39°15'39.95'E; 04 Dec. 1930 • J. Vaughan 1731 (EA, K), Kombeni caves; 6°15'39.2'S, 39°15'39.95"E; Dec. 1930 • J.H. Vaughan 1689 (K); Magharibi (West) District, Kombeni Cave Well; 6°15'S, 39°16'E; 22 Nov. 1930 • P.J. Greenway 2654 (K); Magharibi (West) District, Haitajwa Hill; 6°16'S, 39°16'E; 04 Dec. 1930 – Unknown major area • Botany Students DSM1394 (DSM, K, WAG), Steinbruch Forest Reserve near Maweni, W of Tanga; 5°06'S, 39°01'E; alt. 80 m; 31 Dec. 1969 • G.A. Peter 4487 (B, K, WAG), Useguha; 6°00'S, 38°15'E; 10 Jun. 1914.

#### ﻿ Uvariodendron mbagoi

Taxon classificationPlantaeMagnolialesAnnonaceae

Dagallier & Couvreur, PhytoKeys 174: 109 (2021)

Figs 31 , 32

##### Type.

Tanzania – Tanga • L.-P.M.J. Dagallier 39 (holotype: MPU! (MPU1375316); isotypes: DSM!, MPU! (MPU1375317), P! (P00948145, P00948146); also distributed to K, MO, WAG); Handeni District, Kwedijela forest, ~8 km Kwamsisi village; 5°54'50.38'S, 38°36'12.35'E; alt. 156 m; 13 Nov. 2019.

##### Description.

Tall shrubs to trees 3–6 m tall, D.B.H. 5–10 cm; young branches sparsely pubescent to glabrous, old branches glabrous; slash with strong bergamot smell (the citrusy smell of *Citrusbergamia* Risso). Leaves stiff, greyish green, with margin slightly revolute. Petiole 3–6.5 mm long, 1.2–3 mm wide, sparsely pubescent to glabrous. Leaf lamina 76–157 mm long, 31–59 mm wide, length:width ratio 2.2–3.5, narrowly elliptic to elliptic to obovate, between coriaceous and cartilaginous, base acute to slightly decurrent (sometimes cuneate), apex acute to shortly acuminate, acumen 5–10 mm long; surface above glabrous, surface below sparsely pubescent to glabrous when young, glabrous when old; midrib impressed above, raised below, glabrous above, sparsely pubescent to glabrous when young, glabrous when old below; secondary veins 10–13 pairs, weakly brochidodromous, indistinct to slightly impressed above, (slightly) raised below; tertiary veins reticulate. Inflorescences borne on trunk and old branches, composed of 1–2 (3) sessile flowers. Flower pedicel 0–0.6 mm long, ca 2 mm in diameter, velutinous. Flowers bisexual, buds globose, 5–9 mm high, 5–9 mm in diameter, velutinous, falling off very easily. Only flower buds and old fallen flowers seen. Bracts 2 to 5 at base of the pedicel, upper bract 4–8 mm long, 10–15 mm wide, appressed, enclosing the bud, pubescent outside, glabrous inside. Sepals 3, ca. 7–8 mm long, ca. 7–12 mm wide (measures taken from bud), imbricate, enclosing the petals in bud, velutinous outside, glabrous inside, brown. Outer petals 3, 4.5–5.5 mm long, 4.5–5 mm wide (measures taken from bud), length:width ratio ca. 1, ovate, shortly velutinous outside, glabrous inside, color unknown. Inner petals 3, 3.5–4.5 mm long, 4–4.5 mm wide (measures taken from bud), length:width ratio ca. 1, ovate, glabrous outside, glabrous inside, color unknown. Stamens 400 to 500, mature length unknown, anthers linear, connective prolongation truncate. Carpels 12 to 16, ca. 1.5 mm long, ca. 1 mm wide (measures taken from old flower), velutinous, free; stigma mature length unknown, coiled. Fruiting pedicel 0–6 mm long, ca. 4 mm in diameter, pubescent. Monocarps 1 to 7, 20–50 mm long, 10–12 mm wide, length:width ratio 2–4.5, cylindrical, generally curved, longitudinally ridged and slightly constricted between the seeds, tomentose with regular tufts of high hair density, green-grey; sessile to shortly stipitate, stipe 0–1.5 mm long, ca. 5 mm wide, tomentose. Seeds 4–17 per monocarp, uniseriate to biseriate, 8 to 8.5 mm long, 5.5–6 mm wide, ellipsoid.

**Figure 31.**
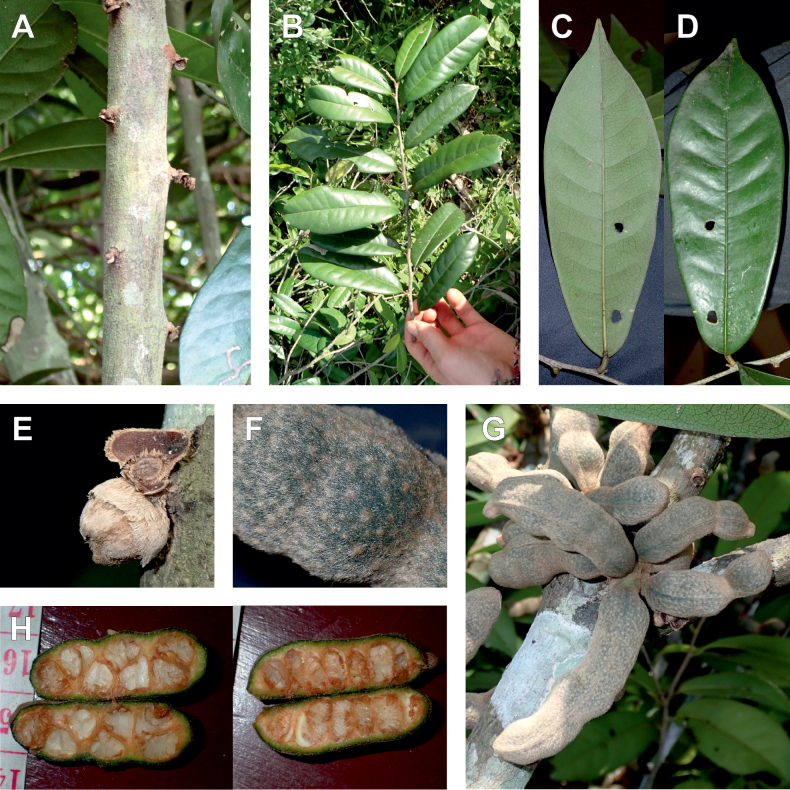
*Uvariodendronmbagoi* Dagallier & Couvreur **A** trunk with old or fallen flowers **B** young branch with leaves, upper side **C** leaf, lower side **D** leaf, upper side **E** bract remaining from **A** fallen flower (top) and flower bud (bottom) **F** detail of **A** moncarp, note the hair pattern **G** fruit, semi-top view **H** tangential (left) and longitudinal (right) cuts of **A** monocarp. **A, C** Dagallier 40 **B, D–H** Dagallier 39 (type). Photos Léo-Paul Dagallier.

##### Distribution.

Endemic to Somalia-Masai Region: Tanzania.

##### Habitat and ecology.

Lowland rain forest or dry forest. Soil: coral rag. Altitude: 90–340 m a.s.l.

##### Phenology.

Flowers collected from August to December. Fruits collected from August to November.

##### Vernacular names.

Tanzania: ‘Mkenene’ in Chizigua (Bloesch s.n.; Couvreur 3; Dagallier 39, 40).

##### Uses.

The bark is used as spice for tea or meat meals (Dagallier 39).

##### Notes.

This species differs from the other *Uvariodendron* species by the strong bergamot scent of crushed fresh and dry leaves and bark, by having stiff greyish-green leaves with slightly revolute margins, and by its globose flower buds easily falling off and its tomentose fruits having regular tufts of higher hair density.

**Figure 32.**
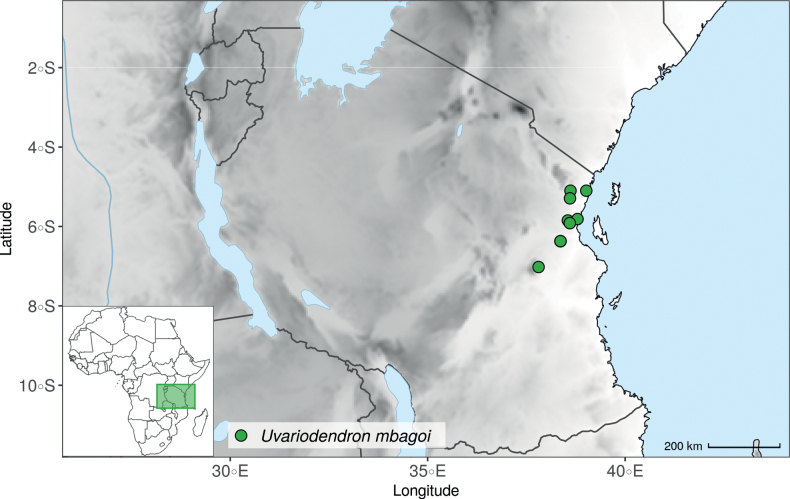
Distribution map of *Uvariodendronmbagoi*. Shades of grey represent elevation, from white (sea level) to darker grey (higher elevation). The inset shows the extent of the map over Africa.

##### Preliminary conservation status.

Following IUCN criterion B, this species has been previously assigned a preliminary status of EN B1ab(i,ii,iii,iv)+2ab(i,ii,iii,iv) (Dagallier et al. 2021).

##### Additional specimens examined.

Tanzania – Morogoro • L.-P.M.J. Dagallier 50 (DSM, K, MPU, P, WAG); Morogoro Rural District, Kimboza forest; 7°01'18.38“S, 37°48'32.13"E; alt. 267 m; 15 Nov. 2019 – Pwani • L.-P.M.J. Dagallier 1 (DSM, K, MPU, P, WAG), Msata Hill, 30 km North of Chalinze; 6°22'17.78“S, 38°21'49.97"E; alt. 317 m; 06 Nov. 2019 • L.-P.M.J. Dagallier 3 (WAG), Msata Hill, 30 km North of Chalinze; 6°22'17.09“S, 38°21'50.23"E; alt. 328 m; 06 Nov. 2019 • L.-P.M.J. Dagallier 6 (WAG), Msata Hill, 30 km North of Chalinze; 6°22'17.93“S, 38°21'49.94"E; alt. 323 m; 06 Nov. 2019 • T.L.P. Couvreur 3 (DSM, WAG); Bagamoyo District, Mazizi hill, on road between Chilinze and Wami River; 6°22'14.4“S, 38°21'51"E; alt. 100 m; 09 Nov. 2006 • U. Bloesch s.n (WAG), Kwedijela Coastal Forest, T3; 5°55'S, 38°36'E; 18 Sep. 2004 – Tanga • C.M. Kisena 3039 (MO); Handeni District, T3; Collected from Kwedivikilo sacred forest near Manga Village; 5°06'S, 38°37'E; 17 Nov. 1997 • F.M. Mbago 3323 (DSM, K); Handeni District, Kwedijela forest, 8 km Kwamsisi village; 5°51'S, 38°33'E; 07 Oct. 2004 • G.A. Peter 52283 (B, K, WAG); Pangani District, Useguha. Inseln des Pangani bei Hale; 5°17'34.8“S, 38°36'14.06"E; alt. 340 m; 31 Jan. 1915 • L.-P.M.J. Dagallier 40 (DSM, K, MO, MPU, P, WAG); Handeni District, Kwedijela forest, ~8 km Kwamsisi village; 5°54'50.77“S, 38°36'13.27"E; alt. 155 m; 13 Nov. 2019 • T.C.E. Congdon 532 (K); Pangani District, Mkwaja Ranch; 5°48'50.76“S, 38°47'40.92"E; alt. 90 m; 04 Dec. 1998 • W.D. Hawthorne 1420A (K), Mkulumuzi river, karst river valley, Steinbruch reserve; 5°06'S, 39°01'00.12»E; 12 Aug. 1982.

#### ﻿ Uvariodendron molundense

Taxon classificationPlantaeMagnolialesAnnonaceae

(Diels) R.E.Fr., Acta Horti Berg. 10: 61 (1930)

Figs 33 , 34 , 35

≡ Uvaria molundensis Diels, Bot. Jahrb. Syst. 53(3–5): 435 (1915). Type. Cameroon – East Region • G.W.J. Mildbraed 4373 (holotype: B! (B 10 0153118)), Südkameruner Waldgebiet: Bezirk Molundu, ‘Bange Busch’, unbewohnter Urwald zwischen Lokomo, Bumba und Bange; 2°50'N, 15°15'E; 29 Jan. 1911.
= Uvaria letestui Pellegr., Bull. Mus. Natl. Hist. Nat. xxvi. 658 (1920); Uvariodendronletestui (Pellegr.) R.E.Fr., Acta Horti Berg. 10: 60 (1930). Type. Gabon – Nyanga • G.M.P.C. Le Testu 1234 (holotype: P! (P00315833), sheet designated by Couvreur et al (2022); isotypes: BM! (BM000554071, BM000554072), P! (P00315835, P00315837)), Tchibanga; 2°50'S, 11°00'E; Nov. 1907.
= Uvaria mayumbensis Exell, J. Bot. 64(Suppl. 1): 3 (1926); Uvariodendronmayumbense (Exell) R.E.Fr., Acta Horti Berg. 10: 57 (1930). Type. Angola – Cabinda • J. Gossweiler 6159 (holotype: BM! (BM000554073)), Mayombe Pango Mariga [Maringa?]; 4°55'S, 12°25'E; 17 Jan. 1916.
= Uvaria mannii Hutch. & Dalziel, Fl. W. Trop. Afr. I. 50 (1927); Kew Bull 150 (1927). Type. Equatorial Guinea – Bioko Norte • G. Mann 257 (holotype: K! (K000198802); isotype: P! (P00315831)); 3°40'N, 8°47'E; 1860.

##### Description.

Tree to shrub 1.2–10 m tall, D.B.H. 1.5–20 cm; young branches sparsely pubescent to glabrous, old branches glabrous. Petiole 4–17 mm long, 1.5–5 mm wide, sparsely pubescent to glabrous. Leaf lamina (180) 200–460 mm long, 51–160 mm wide, length:width ratio 2.2–4.8, elliptic to oblong to obovate, coriaceous, base acute to rounded (sometimes minutely decurrent at the very base), apex attenuate to acuminate, acumen 2–38 mm long; surface above glabrous, surface below sparsely pubescent to glabrous when young, glabrous when old; midrib impressed above, raised below, glabrous above, sparsely pubescent to glabrous below; secondary veins 12–22 pairs, brochidodromous to weakly brochidodromous, impressed above, raised below; tertiary veins reticulate. Inflorescences borne on trunk and branches or axillary, composed of 1 flower. Flower pedicel 2–14 mm long, 1–4 mm in diameter, pubescent. Flowers bisexual, buds globose, sessile, 3.5–9 mm high, 4–14 mm in diameter, velutinous. Bracts 1 to 6, upper bract 3–8 mm long, 3–10 mm wide, broadly ovate, clasping the pedicel, velutinous outside, glabrous inside. Sepals 3, 5–9 (14) mm long, 5–10 mm wide, imbricate (sometime fused at base), velutinous outside, glabrous inside, green to brown. Outer petals 3, 12–30 mm long, 9–22.5 mm wide, length:width ratio 1.1–2.5, elliptic, pubescent outside, pubescent at apex to glabrous inside, light yellow to cream outside, purple with cream margins inside. Inner petals 3, 11–27 mm long, 8–20 mm wide, length:width ratio 1.2–2.4, obovate, pubescent outside, glabrous inside, light yellow to cream outside, purple with cream margins inside. Stamens 1000 to 1200, 0.8–2.5 mm long, 0.1–1 mm wide, anthers linear, connective prolongation truncate. Carpels 8 to 40, 1–5 mm long, 0.5–2 mm wide, pubescent, free; stigma 0–2 mm long, 1–1.5 mm wide, coiled, densely pubescent, covered with an exudate at anthesis. Fruiting pedicel 3–15 mm long, 2.5–6 mm in diameter, pubescent to glabrous. Monocarps 2 to 10, 21–60 mm long, 9–30 mm wide, length:width ratio 1.3–3.4, cylindrical, generally straight, truncate or rounded at apex, pubescent to glabrate, greyish green to orange; sessile to shortly stipitate, stipe 0–4 mm long, 2–4 mm wide, pubescent to glabrate. Seeds 3–10 per monocarp, biseriate, 12 to 14 mm long, 7–10 mm wide, semicircular, pale brown to pinkish brown.

**Figure 33.**
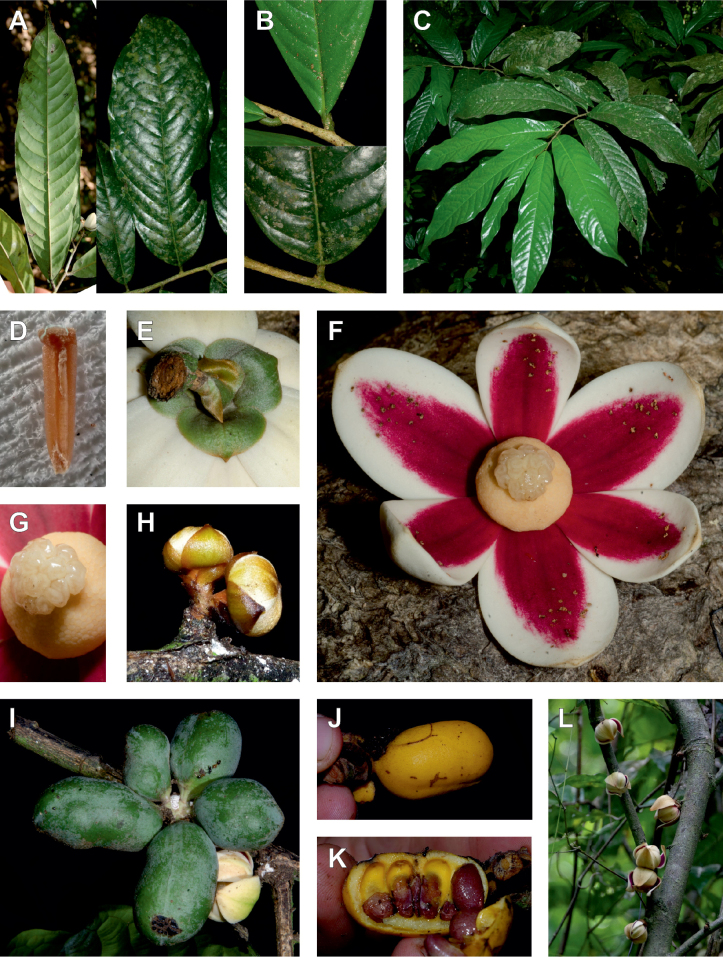
*Uvariodendronmolundense* (Diels) R.E.Fr. **A** leaves, lower side (left) and upper side (right), note the morphological variation **B** leaf bases, upper side, note the morphological variation **C** young branch with leaves **D** stamen, front view **E** detail of flower showing pedicel bracts and sepals, bottom view, not the imbrication of sepals **F** flower, top view **G** detail of flower showing stamens and stigmas, note the gleaming exudate on the stigmas **H** flower buds, side view **I** young fruit with unripe monocarps, top view **J** ripe monocarp, side view **K** monocarp, transversally open **L** branches with borne flowers. **A** (left) **E–H** Bidault 2222 **A** (right) Couvreur 655 **B** (top) Couvreur 542 **B** (bottom) **C** Bidault 4269 **D** Couvreur 1195 **I** Bidault 2248 **J, K** Couvreur 932 **L** Couvreur 1172. Photos **A** (left) **B** (bottom) **C, E–H, I** Ehoarn Bidault **A** (right) **B** (top) **J–L** Thomas Couvreur **D** Léo-Paul Dagallier.

##### Distribution.

Element of the Lower Guinean Domain and Congolia Domain of the Guineo-Congolian Region: Cameroon, Democratic Republic of the Congo, Gabon, Equatorial Guinea (Bioko Island), Republic of the Congo.

**Figure 34.**
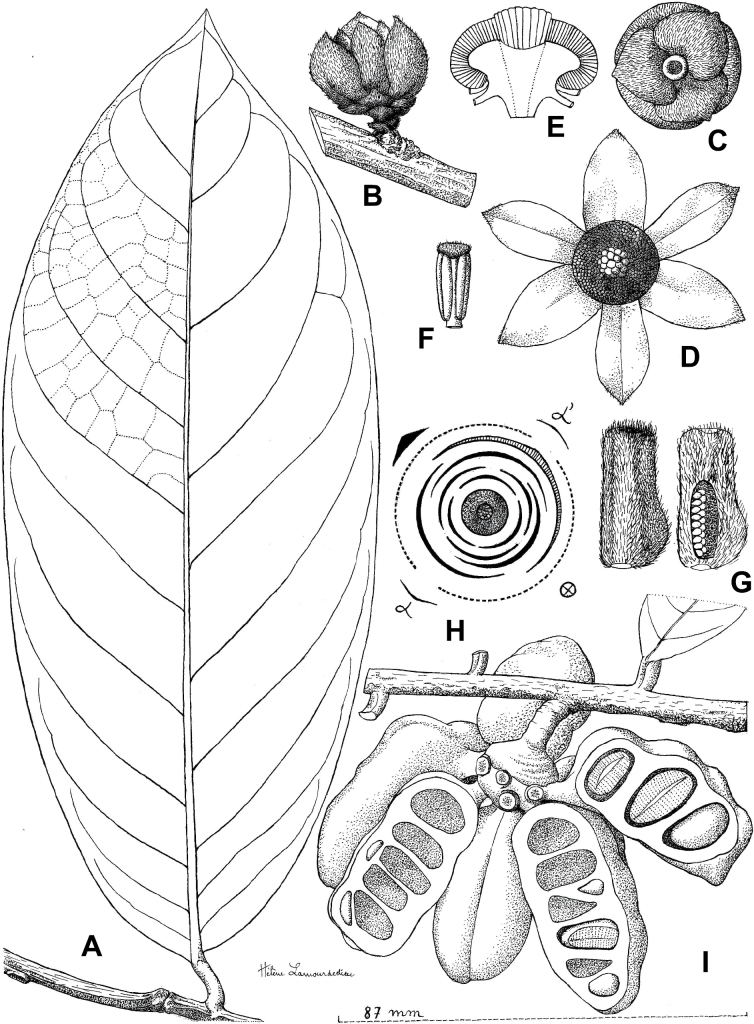
*Uvariodendronmolundense* (Diels) R.E.Fr. **A** leaf **B** flower on branch, side view **C** flower, bottom view **D** flower, petals open, top view **E** longitudinal section of receptacle **F** stamen, front view **G** carpel, side view and detail of ovules **H** floral diagram **I** fruit, longitudinal sections of monocarps **A–C, E–G** from Le Testu 9649 **D** from Le Testu 8437 **H, I** from Hallé 3264. Drawings by Hélène Lamourdedieu, from Le Thomas (1969; pl. 51, p. 281) Publications Scientifiques du Muséum national d’Histoire naturelle, Paris.

##### Habitat and ecology.

Lowland and premontane mature and old secondary rain forests. Altitude: 0–1000 m a.s.l.

##### Phenology.

Flowers and fruits collected all year.

##### Notes.

This species presents a great morphological variability in terms of leaf size and shape. Vegetatively it resembles *Ud.connivens* and *Ud.fuscum* (especially var. fuscum), although it generally has smaller leaves than these two species. In flower, it differs in having a short pedicel 2–14 mm long (vs. a long pedicel 10–40 mm long in *Ud.connivens*), imbricate sepals (vs. fused at base in *Ud.fuscum*), smaller petals (11–30 mm long vs. 20–70 in *Ud.fuscum*), and fewer carpels (eight to 40 vs. 20 to 160 in *Ud.fuscum*).

**Figure 35.**
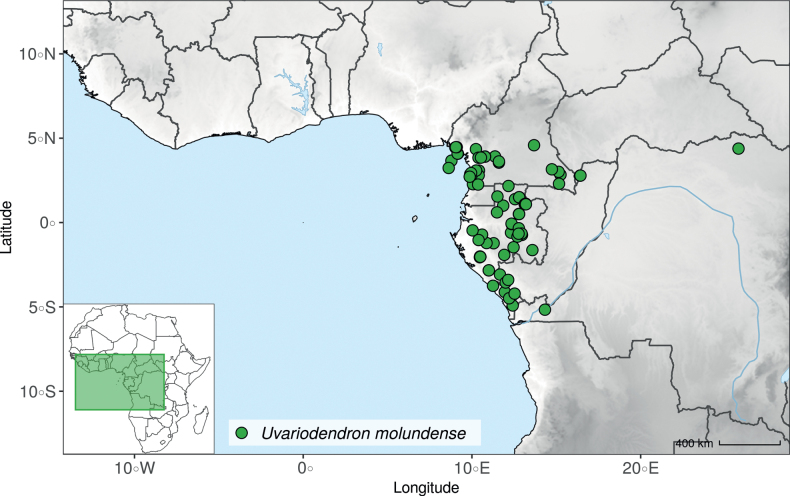
Distribution map of *Uvariodendronmolundense*. Shades of grey represent elevation, from white (sea level) to darker grey (higher elevation). The inset shows the extent of the map over Africa.

##### Conservation status.

This species has been assessed as Least Concern LC (Botanic Gardens Conservation International and IUCN SSC Global Tree Specialist Group 2019a).

##### Additional specimens examined.

Cameroon – Central Region • D. Dang 643 (P, YA), colline ‘ZOABISSIM' dans l'apellation locale, 4 km envirion N du village EKEKAM; 3°55'N, 11°22'E; alt. 1085 m; 09 Mar. 1978 • F. Tadjouteu 566 (K, YA), Ndanan 1, Mefou National park, 18 km from MFOU; 3°33'N, 11°36'02.47'E; alt. 700 m; 22 Mar. 2004 • M.R. Cheek 11839 (YA), Ndanan 1, Eastern part of the park, West of Ndangan 1; 3°37'19'N, 11°36'03'E; alt. 710 m; 18 Mar. 2004 – East Region • J.F. Villiers 625 (P, YA), Camp CFA Bango I, bord de la riv. Bango 36 km SE Bateka Malen, village situé à 21 km N de Molundou (Feuille IGN Molundou 1/200.000); 2°17'49.56'N, 15°09'41.81'E; 08 Nov. 1971 • R.G. Letouzey 3117 (P, YA), Bertoua, Kap; 4°35'N, 13°40'50'E; 23 Feb. 1960 • R.G. Letouzey 5142 (P, YA), a 30 km au NE de Bangé (km75 route Yokadouma – Molundu) feuille IGN 1/200 000. Yokadouma; 3°00'25.8'N, 15°08'13.99'E; 25 May. 1963 • T.L.P. Couvreur 1195 (MPU, WAG, YA), 60 km south of Yokadouma, 30 km after Ngato, 15 km after river. ALPICAM ‘base de vie', then on forestry road starting 4 km before Maséa village; 3°09'19'N, 14°44'16.87'E; alt. 613 m; 04 Mar. 2019 – Littoral • T.L.P. Couvreur 1172 (MPU, P, WAG, YA), Mapubi, 30 km before Edea on Yaoundé-Edea road. On forestry road, 5 km direction to Sanaga river; 3°50'39.26'N, 10°23'22.88'E; alt. 164 m; 28 Feb. 2018 • T.L.P. Couvreur 613 (MPU, YA), Ebo Wildlife Reserve, Djuma permanent camp. On Djashaka trail; 4°21'06.18'N, 10°14'12.1'E; alt. 378 m; 13 Feb. 2014 • T.L.P. Couvreur 652 (WAG, YA), Mambe Massif, above Boga village, 100 km along road from Yaoundé to Edea; 3°54'36.26'N, 10°46'31.86'E; alt. 637 m; 19 Jun. 2014 • T.L.P. Couvreur 655 (MPU, WAG, YA), Mambe Massif, above Boga village, 100 km along road from Yaoundé to Edea; 3°54'29.55'N, 10°46'20.29'E; alt. 657 m; 19 Jun. 2014 • T.L.P. Couvreur 656 (WAG, YA), Mambe Massif, above Boga village, 100 km along road from Yaoundé to Edea; 3°54'24.79'N, 10°46'17.82'E; alt. 653 m; 20 Jun. 2014 • W.J.J.O. de Wilde 1420 (K, MO, P, WAG), 40 km NW. of Eséka, W. of Yaoundé; 3°51'N, 10°32'E; 12 Dec. 1963 – South Region • B.-A. Nkongmeneck 800 (YA); Océan, environs de Mvini, à 34 km E de Campo. Feuille IGN 1/200 000 Kribi-Nyabessa; 2°22'18.12'N, 10°06'15.48'E; 24 Oct. 1984 • G.A. Zenker 3511 (BM, K, L, P), Kamerun, Bipinde; 3°05'N, 10°25'E; 1908 • G.P. Tchouto Mbatchou T8X70 (WAG), Campo Ma'an area, Bibabimvoto, Dipikar island, in the Campo area along transect T8; 2°16'16'N, 10°03'35'E; alt. 60 m; 26 Aug. 2000 • G.W.J. Mildbraed 5936 (B, K), S. Cameroon: Kribi; 2°48'N, 10°24'E; 1911 • J.J. Bos 3259 (P, WAG, YA), About 13 km from Kribi, Ebolowa road; 2°51'N, 10°00'E; 13 Nov. 1968 • J.J. Bos 5474 (BR, MO, P, WAG, YA), S. bank of Lobé river, SE. of Gr. Batanga ferry; 2°52'N, 9°54'E; 11 Oct. 1969 • J.J. Bos 6521 (WAG), 6 km N. of km 46 Kribi – Lolodorf; 3°05'N, 10°15'E; 12 Mar. 1970 • J.J. Bos 7075 (P, WAG), 5 km N. of km 7 Kribi – Ebolowa road, behind Pygmee village; 2°56'N, 9°57'E; 10 Jul. 1970 • M.E. Elad 1270 (KRIBI, WAG), Campo-Ma'an area, Bibabimvoto, along transect T4; 2°15'03'N, 10°21'51'E; alt. 40 m; 01 Feb. 2000 • T.R. van Andel 4228 (KRIBI, U, WAG, YA), Campo Ma'an area, Boussebeliga. Trail to Bimvoa chute; 2°43'N, 9°52'E; alt. 40 m; 26 Oct. 2001 – South-West Region • B. Sonké 1226 (K, SCA); Ndian, Dikome; 4°27'N, 9°01'E; 05 May. 1994 • B.N. Khayota 537 (K, SCA, YA), Upper Boando, footpath to south of village; 4°05'N, 9°10'E; alt. 650 m; 14 Mar. 1995 • D. Kenfack 1509 (MO), Mokoko, at the center base forest; 4°27'N, 9°04'00.12'E; 25 Apr. 2001 • G.P. Tchouto Mbatchou 136 (K, SCA, YA), Mapanja; 4°05'N, 9°09'E; alt. 1040 m; 20 Apr. 1992 • G.P. Tchouto Mbatchou 611 (K, SCA, YA), Mokoko, forest above Bonja village; 4°28'N, 9°06'E; alt. 240 m; 23 Mar. 1993 • M. Akogo 234 (K, SCA, YA), Ekumbe Mofako, Mokoko Forest Reserve, Ekumbe-Mofako; 4°28'N, 9°03'E; alt. 140 m; 21 Apr. 1994. Central African Republic – Sangha-Mbaéré • D.J. Harris 5112 (E, MO), Kongana research camp, 25 km SE of Bayanga; 2°47'N, 16°25'E; 08 Jun. 1994 • D.J. Harris 5504 (E, MO), Kongana camp, 22 km SE of Bayanga; 2°47'N, 16°26'E; 11 Feb. 1996. Democratic Republic of the Congo – Bas-Congo • P. Compère 1112 (K), vallée de la Quinongo. Territ: Songololo. Province: Léopoldville; 5°09'57.14'S, 14°19'37.82'E; 21 Dec. 1959 – Orientale • P. Gérard 5604 (K, WAG); Ango, Digba, Foret de Akare entre rivière Bili et Ase; 4°23'N, 25°48'E; 05 Nov. 1963. Equatorial Guinea – Bioko Sur • W.R.Q. Luke 11889 (EA), Moaba. Pt 140; 3°14'06'N, 8°37'22'E; alt. 8 m; 15 Mar. 2007. Gabon – Haut-Ogooué • G.M.P.C. Le Testu 9649 (P); 1°38'S, 13°35'E; 30 Oct. 1930 – Moyen-Ogooué • T.L.P. Couvreur 928 (LBV, WAG, YA), 27 km after Lambaréné, on road to Bifoum (N1), then around 20 km on road to Lake Azingo; 0°27'52.7'N, 10°02'18.45'E; alt. 95 m; 24 Nov. 2015 • T.L.P. Couvreur 932 (LBV, WAG, YA), 33 km south of Lambaréné, then 18 km on dirt road just after Mimongo village, in Morel petrol consession; 1°02'10.10'S, 10°23'39.95'E; alt. 185 m; 25 Nov. 2015 – Ngounié • G.M.P.C. Le Testu 5762 (BM, P), Mbigou, Mboumi riverside; 1°55'S, 11°55'E; 18 Nov. 1925 • G.V. Dauby 574 (LBV, MO), Est du Parc National de Waka, à ± 5 km au Sud de la rivière Mayi; 1°13'36.11'S, 11°17'09.31'E; alt. 604 m; 17 Feb. 2008 • J.C. Arends 450 (LBV, WAG), Waka River; 1°13'S, 10°52'E; alt. 330 m; 25 Nov. 1984 • O.L.S. Lachenaud 1444 (BRLU, LBV, MO), Mabounié, piste du nord-est; 0°43'02'N, 10°36'05'E; 17 Nov. 2013 • P.J.M. Maas 10207 (LBV, WAG), side roads of road Pény-Mouila, in CBG concession; 2°00'45.10'S, 10°28'52.7'E; alt. 115 m; 10 Nov. 2011 – Nyanga • E. Bidault 4269 (BR, BRLU, LBV, MO, P, WAG), Entre Ndendé et le Congo, au Sud de Nzinga, bords de la rivière Douvono; 3°04'56.11'S, 11°39'34.31'E; alt. 154 m; 04 Apr. 2018 • G.M.P.C. Le Testu 1211 (BM), Tchibanga area, Nganda; 2°50'S, 11°00'E; 31 Oct. 1907 – Ogooué-Ivindo • A. Moungazi 1631 (LBV, MO, P, WAG), Ivindo National Park, route Langoué, rivière Niadou; 0°15'N, 12°25'E; 05 May. 2004 • A. Moungazi 223 (P), Makokou, 10 km S de Makoukou; 0°30'30.82'N, 12°47'45.59'E; alt. 500 m • E. Bidault 2222 (BRLU, LBV, MO), concession forestière Rougier-Ivindo; 0°05'34'N, 12°21'15'E; alt. 283 m; 27 Oct. 2015 • E. Bidault 2248 (BR, BRLU, LBV, MO, MO, P, WAG), concession forestière Rougier-Ivindo; 0°04'05'N, 12°21'15'E; alt. 460 m; 28 Oct. 2015 • J. Florence 930 (P), Station d'Ipassa, 10 Km S de Makokou; 0°30'N, 12°47'E; alt. 500 m; 14 Apr. 1978 • N. Hallé 3264 (L, P, U), Bélinga; 1°05'N, 13°08'E; 19 Nov. 1964 • N. Hallé 3332 (P), Bélinga; 1°05'N, 13°08'E; 25 Nov. 1964 • N. Hallé 431 (MO, P, WAG), Bélinga, mines de fer, le long de la rivière Folley; 1°07'N, 13°11'E; 12 Aug. 1966 • N. Hallé 451 (MO, P), Bélinga, mines de fer, route du Belvédère; 1°05'N, 13°12'E; alt. 900 m; 12 Aug. 1966 • T.L.P. Couvreur 564 (YA), along main trail departing from behind the herbarium at the Research station of Ipassa, in Ivindo National Park; 0°30'13.77'N, 12°47'36.4'E; alt. 519 m; 11 Nov. 2013 – Ogooué-Lolo • A.M. Louis 648 (WAG), ± 70 km N.W. of Lastoursville, along rd. to Achouka, old forest along Lolo R. near Wagny settlement; 0°36'N, 12°19'E; alt. 250 m; 13 Nov. 1983 • F.J. Breteler 13331 (WAG), near Bambidie, E of Lastoursville; 0°44'N, 12°58'E; alt. 300 m; 16 Oct. 1994 • G.M.P.C. Le Testu 49 (P), region de Lastoursville, Lastoursville; 0°50'N, 12°42'E; 1934 • G.M.P.C. Le Testu 8437 (BM, P), region de Lastoursville, Nzocou; 1°28'S, 12°28'E; 12 Oct. 1930 • J.J. Wieringa 6223 (LBV, MO, WAG), c.55 km N of Lastoursville, CEB forestry concession ‘Milolé' (UFA2-UFG2-lot2), foothills of Ngota Mountain; 0°20'24'N, 12°46'48'E; alt. 330 m; 28 Jan. 2008 • J.J. Wieringa 6263 (LBV, MO, WAG), c.30 km ENE of Lastoursville, 15 km on forestry road from Bambidie to Akieni; 0°39'11.4'N, 12°56'36.6'E; alt. 320 m; 29 Jan. 2008 • T.L.P. Couvreur 1110 (LBV, WAG), Lastoursville, in Precious Woods conssesion (CEB). 30 km east of Bambidie village, 55 km from Lastoursville; 0°39'46.04'N, 12°45'55.58'E; 07 Jun. 2016 – Ogooué-Maritime • M.S.M. Sosef 2270 (LBV, WAG), Doudou Mountains National Parc, c. 5 km S of Camp Peny (CBG); 2°03'30'S, 10°28'12'E; alt. 100 m; 14 Nov. 2005 • T.L.P. Couvreur 539 (MPU, YA), 5 km on loggers road from Peny (CGB) village; 2°01'55.10'S, 10°29'59.48'E; alt. 188 m; 07 Nov. 2013 • T.L.P. Couvreur 542 (MPU, YA), 5 km on loggers road from Peny (CGB) village; 2°01'53.10'S, 10°29'45.29'E; alt. 200 m; 07 Nov. 2013 – Woleu-Ntem • G.M.P.C. Le Testu 9064 (P), region de Minvoul, Minvoul; 2°10'N, 12°10'E; Mar. 1933 • L. Ngok Banak 1570 (LBV, LBV, MO, P, WAG), Minkébé National Park, southern inselberg area; 1°23'10.2'N, 12°33'17.4'E; alt. 620 m; 02 May. 2003 • M.S.M. Sosef 1894 (WAG), forestry concession Bordamur, c. 40 km NE of Mitzic; 1°00'06'N, 11°51'12'E; alt. 500 m; 06 Feb. 2003 • MINKébé Series AM60 (WAG), Minkébé area, at 2000 m from the camp; 1°30'N, 12°48'E; 25 Mar. 1990 • MINKébé Series V150 (WAG), Minkébé area, 10 x 10 m inventory plot V, placed 3.5–13.5 m north at 200–210 m on transect A; 1°30'N, 12°48'E; 04 Mar. 1990 • MINKébé Series W15 (WAG), Minkébé area, near base camp number 1; 1°29'N, 12°49'E; 08 Feb. 1990 • MINKébé Series W403 (WAG), Minkébé area, 800 m on transect B (to the south); 1°30'N, 12°48'E; 20 May. 1990 • T.L.P. Couvreur 853 (LBV, WAG, YA), Oyem, on road to Mbolonzok (off main road to Mongono and Equatorial Guinea); 1°32'39.18'N, 11°30'43.04'E; alt. 660 m; 13 Nov. 2015 • T.L.P. Couvreur 876 (LBV, WAG, YA), on road from Mitzic to Lalara (N2), just after the bridge over the Lara, c.500 m in forest; 0°36'12.83'N, 11°29'11.53'E; alt. 580 m; 15 Nov. 2015. Republic of the Congo – Kouilou • C. Farron 4882 (P), Pointe Noire, route du chantier de Boungolo (Pointe-Noire); 4°07'S, 11°57'E; 31 Jan. 1966 – Mianga N'Bissy 183 (P), Moyen Congo, Mayombe central, vers km 57 chemin de fer Pointe Noire – Brazzaville; 4°29'27'S, 12°12'15'E; Sep. 1947 • P. Sita 3759 (P), route du nouveau campement Maamar sur 18 km après l'ancien; 3°45'S, 11°15'E; 09 May. 1974 • T.L.P. Couvreur 789 (IEC, WAG), 30 km on Dolisie-Mvouti road, just behind the telephone antenna; 4°12'49.12'S, 12°31'44.71'E; alt. 710 m; 21 Sep. 2015 – Niari • P. Cabalion 153 (P), 3 km O Banga Route Vounda-Banda; 3°33'18.97'S, 11°59'56.48"E; 06 Feb. 1976 • P. Sita 3995 (P), Niari-ouest, region Banda, Vallé de la Ngouanga; 3°24'S, 12°09'E; 05 Nov. 1975.

#### ﻿ Uvariodendron mossambicense

Taxon classificationPlantaeMagnolialesAnnonaceae

Robson ex Dagallier & Couvreur sp. nov.

urn:lsid:ipni.org:names:77326970-1

**Figure 36.**
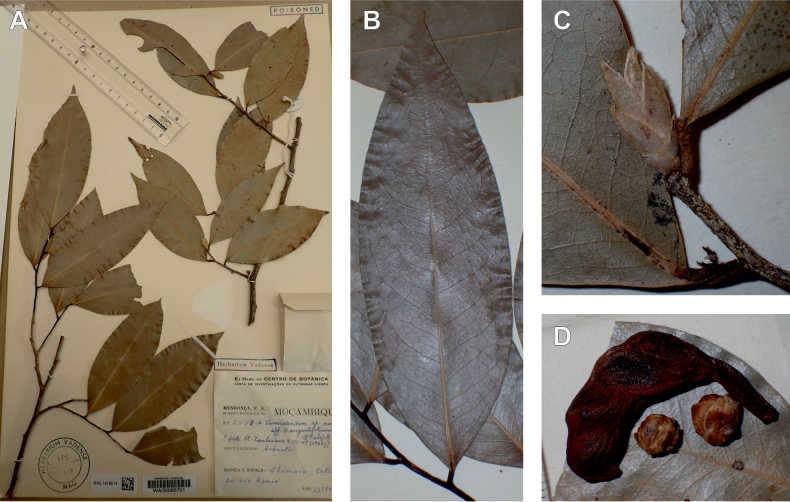
*Uvariodendronmossambicense* Robson ex Dagallier & Couvreur **A** entire specimen sheet with young branch and leaves **B** leaf, top view **C** ‘eragrostiform’ apical bud **D** monocarp and flower buds. **A–D** Mendonça 2558A (type). Photos Léo-Paul Dagallier.

**Figure 37.**
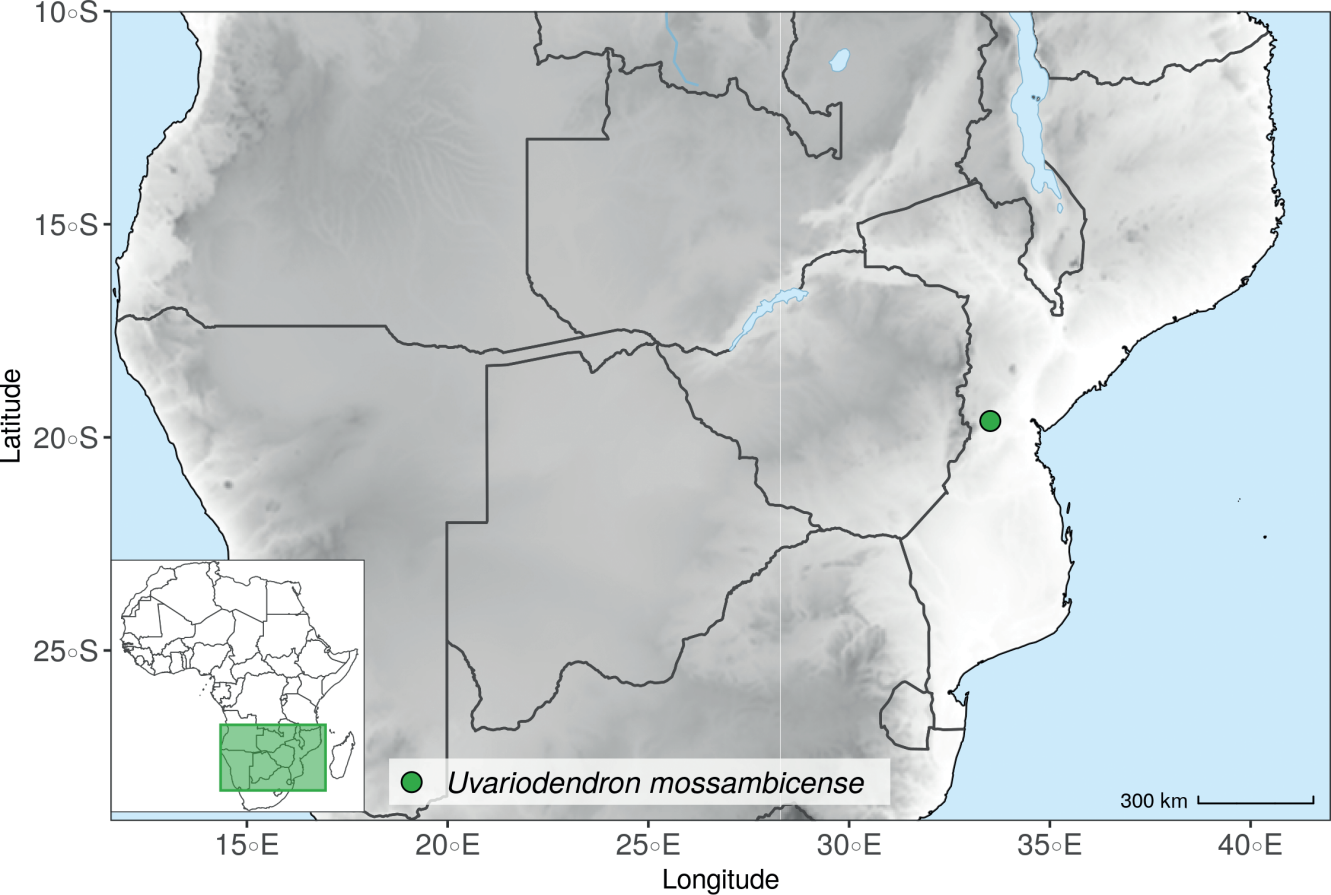
Distribution map of *Uvariodendronmossambicense*. Shades of grey represent elevation, from white (sea level) to darker grey (higher elevation). The inset shows the extent of the map over Africa.

Figs 36 , 37 ; Table 3

##### Type.

Mozambique – Manica • F. de A. Mendonça 2558A (holotype: WAG! (WAG.1418614); isotypes: COI! (COI00085396), LISC), Chimoio, Cataratas do Rio Revué; 19°37'S, 33°31'E; 23 Oct. 1944.

##### Diagnosis.

*Ud.mossambicense* closely resembles *Ud.dzomboense* by its narrowly elliptic and small (less than 140 mm long) leaves with acute to slightly decurrent base. However, *Ud.mossambicense* has flower buds ca. 6 mm in diameter (vs ca. 4 mm in *Ud.dzomboense*), covered by 2–5 velutinous bracts ca. 4 mm long (vs. 6 sparsely pubescent bracts 5–6 mm long). *Ud.mossambicense* has ca. 5 carpels (vs. 50–75 in *Ud.dzomboense*) and monocarps with stipe ca. 12 mm long (vs. sessile monocarps) (Table 3). *Ud.mossambicense* can be differentiated from all the other species by the combination of the following characters: narrowly elliptic leaves less than 140 mm long with acute to slightly decurrent base, flowers with ca. 5 carpels and monocarps with stipe ca. 12 mm long.

##### Description.

Probably a small tree but height unknown, D.B.H. unknown; young branches pubescent to glabrous, old branches glabrous; leaf bud ‘eragrostiform’, composed of 7, distichous, longitudinally folded, velutinous scales. Petiole 3–5 mm long, ca. 1.5 mm wide, glabrous. Leaf lamina 80–135 mm long, 30–50 mm wide, length:width ratio ca. 3, narrowly elliptic, coriaceous, base acute to slightly decurrent, apex attenuate, surface above glabrous, surface below glabrous; midrib impressed above, raised below, glabrous above, glabrous below; secondary veins 10–16 pairs, brochidodromous, impressed above, raised below; tertiary veins reticulate. Inflorescences borne on branches, composed of 1 flower. Flowers bisexual, buds globose, sessile, ca. 6 mm high, ca. 6.5 mm in diameter, velutinous. Only flower bud seen. Bracts 2 to 5, upper bract ca. 4 mm long, ca. 7 mm wide, broadly ovate, appressed, half-enclosing the flower bud, velutinous outside. Sepals 3, 4–5 mm long, ca. 6 mm wide (measures taken from bud), broadly ovate, fused at base on almost 50 % of their length, forming a 3-lobed cupule, velutinous outside, glabrous inside. Outer petals 3, ca. 5 mm long, ca. 5 mm wide (measures taken from bud), length:width ratio ovate, velutinous outside. Inner petals 3, length, shape, indumentum and color unknown. Stamens numerous, length unknown, anthers linear. Carpels ca. 5, glabrous, free; stigma unknown, pubescent. Fruiting pedicel unknown. Monocarp 1 seen, ca. 37 mm long, ca. 9 mm wide, length:width ratio ca. 4.1, cylindrical, curved, slightly acuminate, glabrous, (monocarp found in the pocket of the specimen); stipitate, stipe ca. 12 mm long, ca. 3 mm wide, glabrate. Seeds 4 per monocarp, uniseriate, size and shape unknown.

##### Distribution.

Endemic to Somalia-Masai Region. Only known from one locality in Mozambique: Chimoio, near the falls of Revué river.

##### Habitat and ecology.

Unknown. Altitude around 100 m a.s.l.

##### Phenology.

Flowers and fruits collected in October.

##### Etymology.

The specific epithet comes from the country where the single specimen of this species was found.

##### Preliminary conservation status.

This species is known from a single location, not situated in a protected area. Its EOO and AOO are thus estimated lower than 4 km^2^. Although the botanical exploration of the country is continuously updated (Hyde et al. 2021), this species is known from a single specimen collected more than 70 years ago. It is thus possible that this species is extinct. Following IUCN criterion B, we assigned this species a preliminary conservation status of Critically Endangered CR B1ab(iv)+2ab(iv).

#### ﻿ Uvariodendron occidentale

Taxon classificationPlantaeMagnolialesAnnonaceae

Le Thomas, Adansonia sér. 2, 7: 251 (1967)

Figs 38 , 39

##### Type.

Ivory Coast – Oumé • A. Aubréville 4140 (holotype: P! (P00315840), sheet here designated; isotype P! (P00315843)), Oume, région d'Oumé; 6°23'N, 5°25'W; 28 Feb. 1957.

##### Description.

Tree 5–10 m tall, D.B.H. up to 15 cm; young branches glabrous, old branches glabrous. Petiole 5–10 mm long, 1–3 mm wide, sparsely pubescent to glabrous. Leaf lamina 170–350 mm long, 55–95 mm wide, length:width ratio 2.5–3.7, elliptic to obovate, coriaceous, base acute to decurrent, apex acuminate, acumen 7–21 mm long; surface above glabrous, surface below sparsely pubescent to glabrous when young, glabrous when old; midrib impressed above, raised below, glabrous above, glabrous below; secondary veins 10–18 pairs, weakly brochidodromous, impressed above, raised below; tertiary veins reticulate. Inflorescences borne on trunk, composed of 1 flower. Flower pedicel 6–16 mm long, 1–2 mm in diameter, pubescent. Flowers bisexual, buds globose, pedicellate, 4–6 mm high, 7–8 mm in diameter, pubescent. Bracts 1 to 3, upper bract 2.5–4 mm long, 4.5–5 mm wide, ovate, semi-clasping to clasping the pedicel, pubescent outside, pubescent inside. Sepals 3, 3–5 mm long, 2.5–6 mm wide, depressed ovate to broadly triangular, fused at base in a ring, pubescent outside, glabrous inside, color unknown. Outer petals 3, 15–25 mm long, 10–17 mm wide, length:width ratio 1.4–1.6, elliptic, pubescent outside, glabrous inside, yellow with purple marks on the margins outside, yellow with purple marks inside. Inner petals 3, 10–21 mm long, 6–10 mm wide, length:width ratio obovate, pubescent outside, glabrous inside, color unknown. Stamens 600 to 800, 2–2.5 mm long, 0.4–0.5 mm wide, anthers linear, connective prolongation truncate and puberulent. Carpels 20 to 40, 3–4 mm long, 1 mm wide, pubescent, free; stigma 0.5–1 mm long, 1 mm wide, coiled, pubescent. Fruiting pedicel 15–22 mm long, 2–3.5 mm in diameter, pubescent to glabrous. Monocarps 6 to 11, 17–50 mm long, 7–20 mm wide, length:width ratio 1.6–2.7, cylindrical, curved, longitudinally ridged, acuminate, pubescent to glabrate, color unknown; stipitate, stipe 5–12 mm long, 1–2.5 mm wide, puberulent. Seeds 9–14 per monocarp, biseriate, 7 to 5 mm long, ca. 5 mm wide.

**Figure 38.**
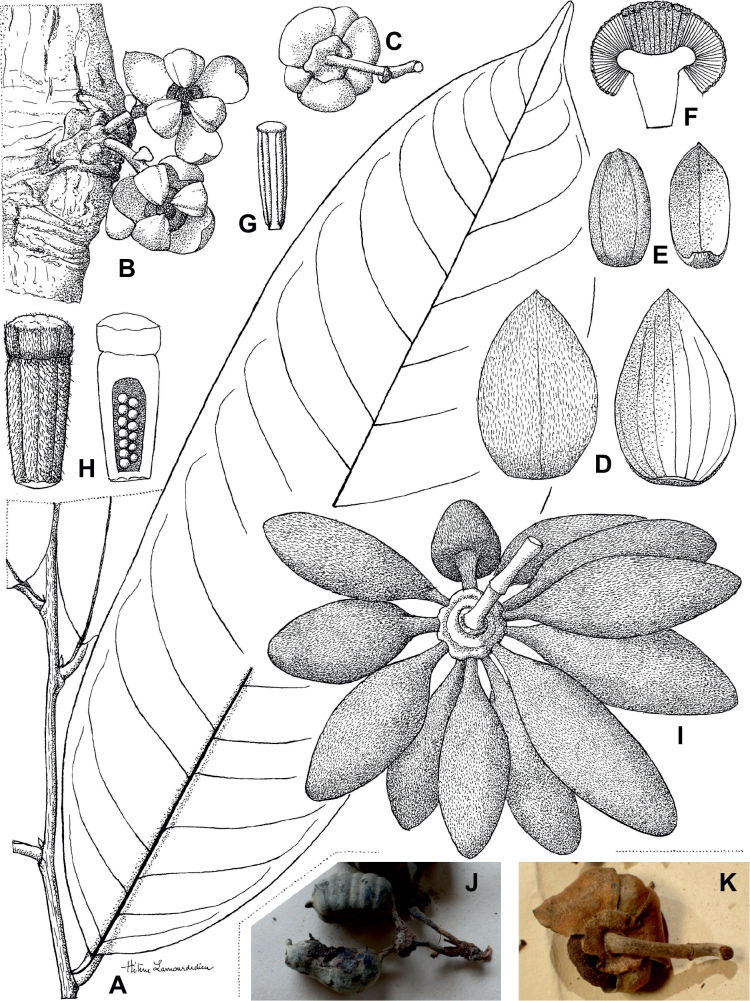
*Uvariodendronoccidentale* Le Thomas **A** leaf **B** inflorescence **C** flower, bottom view **D** outer petals, outside (left) and inside (right) views **E** inner petals, outside (left) and inside (right) views **F** longitudinal section of the receptacle **G** stamen, front view **H** carpel and longitudinal section **I** fruit, bottom view **J** fruit, side view **K** flower, one outer and one inner petals removed, bottom view. **A–I** from Aubréville 4140 (type) **J** Kouamé 1487 **K** Aubréville 4140. Drawings by Hélène Lamourdedieu, modified from Le Thomas (1967; pl. 1 p. 250), Publications Scientifiques du Muséum national d’Histoire naturelle. Photos Léo-Paul Dagallier.

##### Distribution.

Element of the Upper Guinean Domain and Lower Guinean Domain of the Guineo-Congolian Region: Ghana, Ivory Coast, Nigeria.

##### Habitat and ecology.

Lowland mature rain forest. Altitude: 155–250 m a.s.l.

##### Phenology.

Flowers collected from October to February. Fruits collected from January to April.

##### Vernacular names.

Ivory Coast: ‘Michiti à grandes feuilles’ (Aubréville 4140).

##### Etymology.

A. Le Thomas initially named this species *Ud.occidentalis* (Le Thomas 1967). However, *Uvariodendron* is neutral as -*dendron* -tree- is neutral in Greek (Article 62.2.(c) in (Turland et al. 2018), the termination of the adjective *occidental* -western- should be -*e* and not -*is.* In accordance with Articles 23.5, 32.2 and 60.1 (Turland et al. 2018), it is thus corrected here as *Ud.occidentale*.

**Figure 39.**
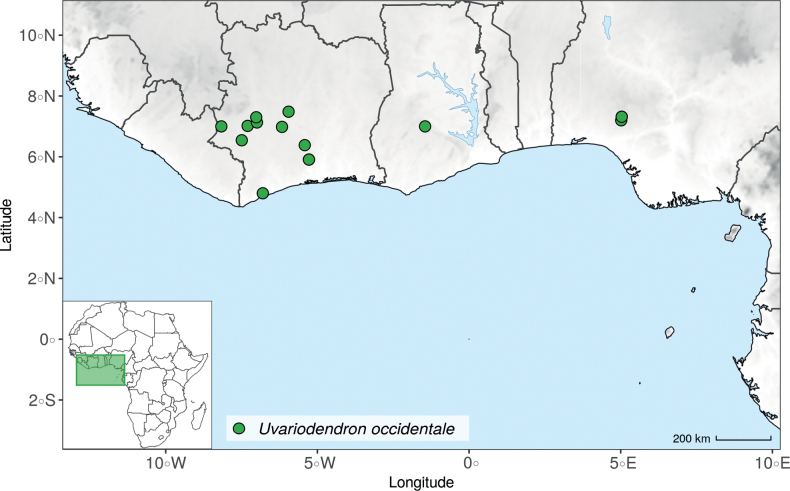
Distribution map of *Uvariodendronoccidentale*. Shades of grey represent elevation, from white (sea level) to darker grey (higher elevation). The inset shows the extent of the map over Africa.

##### Notes.

This species resembles *Ud.fuscum* and *Ud.molundense* but can be differentiated in having leaf bases acute to decurrent (vs. acute to rounded), flower slender flower pedicel 6–16 mm long and 1–2 mm wide (vs. flower subsessile in *Ud.fuscum* and flower pedicel rather short and thick 2–14 mm long and 2–4 mm wide in *Ud.molundense*), and sepals 3–5 mm long fused at base forming a ring (vs. 11–55 mm long and fused in *Ud.fuscum* and 5–9 mm long and free in *Ud.molundense*).

##### Preliminary conservation status.

A previous assessment, that needs updating, listed this species as Vulnerable VU under criteria A1c (Hawthorne 1998). Here its EOO is estimated at 223,596 km^2^ and AOO at 52 km^2^. It would qualify for Endangered EN based solely on AOO, but none of the other sub-criteria are met. Given it occurs in less than 20 locations and given its low AOO, it is likely to qualify for a threatened category in the future. Following IUCN criterion B, it is thus assigned a preliminary updated conservation status of Near Threatened NT.

##### Additional specimens examined.

Ghana – Ashanti Region • J.E. Andoh 4171 (K, P), Ofin Headwaters Reserve; 7°00'N, 1°27'W; Apr. 1936. Ivory Coast – Bouaflé • C.C.H. Jongkind 4349 (WAG), Parc National de la Marahoue. Near south border; 6°59'N, 6°10'W; alt. 250 m; 11 Feb. 1998 – Daloa • F.N. Kouamé 1443 (CSRS, G), F.C. du Haut-Sassandra, Nord. forêt dégradée, relevé FNK14; 7°01'N, 7°18'W; 04 Apr. 1995 • F.N. Kouamé 1486 (CSRS, G), F.C. du Haut-Sassandra, Centre. forêt dégradée de pente, relevé FNK14, entre layons 10 et 11; 7°00'N, 8°10'W; 13 Apr. 1995 • F.N. Kouamé 1487 (G), F.C. du Haut-Sassandra, Centre, forêt dégradée, relevé FNK14; 7°07'45.48'N, 6°59'29.4'W; 13 Apr. 1995 – Divo • L. Aké Assi 4633 (P), Divo, Foret Classee de, Forêt de Divo; 5°54'30.6'N, 5°16'41.52'W; alt. 155 m; 11 Jun. 1958 • L. Aké Assi 6629 (G), Divo, Foret Classee de, Forêt de Divo; 5°54'30.6'N, 5°16'41.52'W; alt. 155 m; Nov. 1962 – Guiglo • A. Aubréville 1224 (P), Giglo, Debokun; 6°32'37.26'N, 7°29'36.58'W; 18 Apr. 1932 • A. Bakayoko 21 (G), Guiglo, Zaipobly, forêt sur colline; 7°29'N, 5°57'W; 27 Jan. 2001 – San-Pédro • L. Aké Assi 17192 (G), route de Tabou, forêt près de Para; 4°48'N, 6°48'W; 28 Dec. 1985 – Unknown major area • F.N. Kouamé 1623 (CSRS, G), Daloa F.C. du Haut-Sassandra, Nord. forêt très dégradée sur cuirasse, relevé FNK23; 7°18'N, 7°01'W; 07 Feb. 1995 – Unknown collector 1780 (P (P01982836)), Bords de l'Agnéby, Mudjika; Feb. 1933. Nigeria – Ondo State • F. Anakwense FHI19701 (K); Akure, Akure Forest Reserve; 7°19'N, 5°02'E; 11 Dec. 1950 • K. Obeng-Darko 182 (K); Akure, Owena Forest Reserve, Owena; 7°12'N, 5°01'E; 20 Feb. 1946 • R.W.J. Keay FHI24604 (K); Akure, Akure Forest Reserve, Block 6C Akure FR; 7°19'N, 5°02'E; 28 Oct. 1948.

#### ﻿ Uvariodendron pilosicarpum

Taxon classificationPlantaeMagnolialesAnnonaceae

Dagallier & Couvreur sp. nov.

urn:lsid:ipni.org:names:77326971-1

Figs 40 , 41

##### Type.

Gabon – Nyanga • J.L.C.H. van Valkenburg 2540 (holotype: WAG! (WAG.1418718); isotypes: BR! (BR0000009320413), E, K, LBV, MO, P! (P01956020), WAG! (WAG.1418719, WAG.1418720)), concession SFN; 2°39'34.2'S, 10°27'03'E; alt. 200 m; 01 Nov. 2003.

##### Diagnosis.

*Ud.pilosicarpum* resembles *Ud.molundense* and *Ud.citriodorum* but can be differentiated in having the longest leaves smaller than 25 cm (vs. longest leaves greater than 25 cm long), obovate leaves (vs. elliptic to oblong to obovate leaves), with base decurrent (vs. base rounded to convex) and monocarps ellipsoid, curved, acuminate and densely pubescent (vs. monocarps cylindrical, straight and pubescent to glabrate).

##### Description.

Tree 4–8 m tall, D.B.H. ca. 8 cm; young branches sparsely pubescent to glabrous, old branches glabrous. Petiole 4–7 mm long, 1.5–2.5 mm wide, sparsely pubescent to glabrous. Leaf lamina 178–243 mm long, 60–87 mm wide, length:width ratio 2.3–3.6, obovate, coriaceous, base decurrent, apex acuminate, acumen 13–21 mm long; surface above glabrous, surface below sparsely pubescent to glabrous when young, glabrous when old; midrib impressed above, raised below, glabrous above, glabrous below; secondary veins 12–19 pairs, weakly brochidodromous, impressed above, raised below; tertiary veins reticulate. Inflorescences borne on trunk and branches, composed of 1 flower. Flower pedicel unknown. Flowers bisexual, buds unknown. Mature flower unknown, measures taken from fruits. Bracts 1, ca. 10 mm long, ca. 12 mm wide, broadly ovate, semi clasping the pedicel, velutinous outside, glabrous inside. Sepals 3, 9–10 mm long, 12.5–18 mm wide (measures taken from young fruits), imbricate, pubescent outside, glabrous inside, color unknown. Outer petals unknown. Inner petals unknown. Stamens unknown. Carpels ca. 30, free. Fruiting pedicel 8–13 mm long, 3.5–5.5 mm in diameter, pubescent to sparsely pubescent. Monocarps 6 to 30, 23–32 mm long, 10–15 mm wide, length:width ratio 2.2–2.3, ellipsoid, curved, acuminate at apex, densely velutinous, bright green with brown indumentum to glaucous with silvery indumentum, with resinous fragrance when cut; sessile to shortly stipitate, stipe 0–2 mm long, 2–3 mm wide, densely pubescent. Seeds unknown.

**Figure 40.**
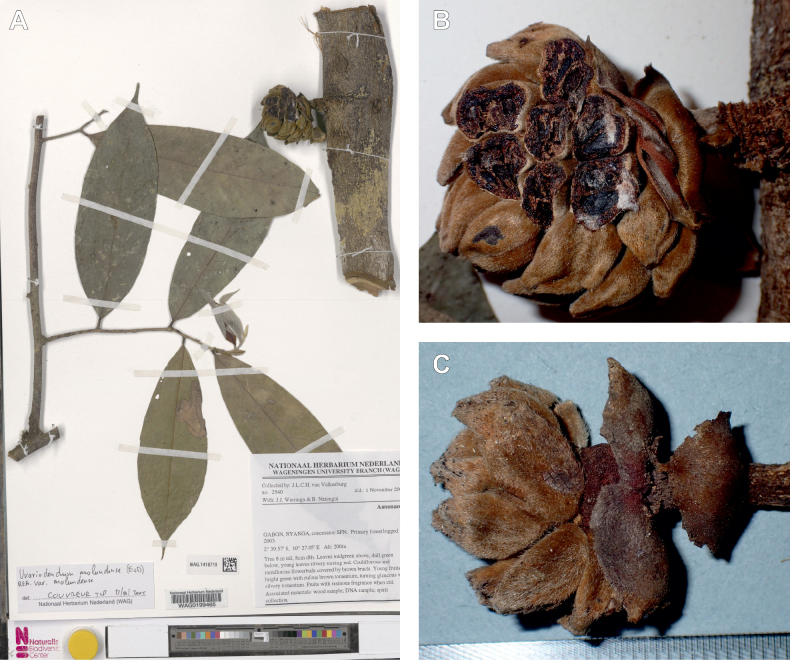
*Uvariodendronpilosicarpum* Dagallier & Couvreur **A** full specimen sheet with young branch and leaves and fruit **B** fruit with some monocarps transversally cut **C** young fruit with remaining sepals and bract. **A, B** van Valkenburg 2540 (type) **C** Sosef 2261. Photos **A** Naturalis (https://data.biodiversitydata.nl/naturalis/specimen/WAG.1418718) **B, C** Léo-Paul Dagallier.

##### Distribution.

Endemic to Lower Guinean Domain of the Guineo-Congolian Region: Gabon.

##### Habitat and ecology.

Lowland mature or secondary rain forest. Altitude: 100–200 m a.s.l.

##### Phenology.

Flowers and fruits collected in November.

##### Etymology.

The specific epithet refers to the densely pubescent monocarps, characteristic of this species.

##### Preliminary conservation status.

This species is known from only two occurrences in the Monts Doudou National Park in Gabon. The AOO is estimated at 8 km^2^. Based on criterion B it would qualify for Endangered EN B1(a)+2(a), but no other subcriteria (b or c) is met. The outlook for this species relies on the future of this protected area and its distribution depends on the effects of human activities or random events in an uncertain future. Following IUCN criterion D, it is thus assigned a preliminary conservation status of Vulnerable VU D2.

**Figure 41.**
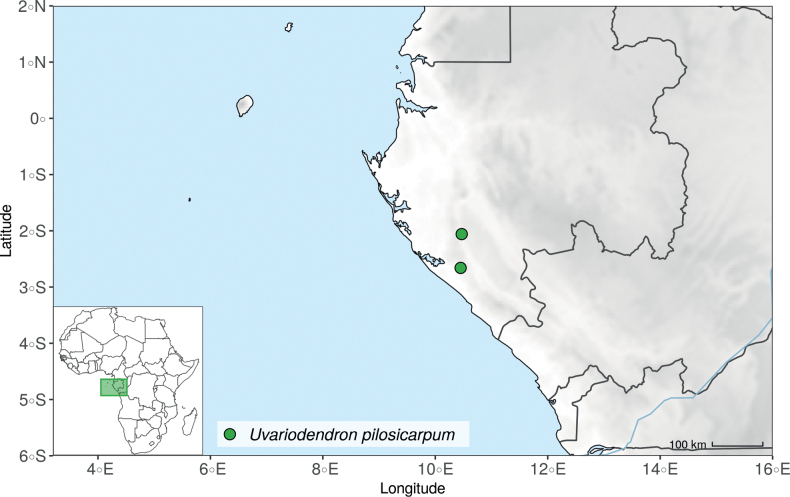
Distribution map of *Uvariodendronpilosicarpum*. Shades of grey represent elevation, from white (sea level) to darker grey (higher elevation). The inset shows the extent of the map over Africa.

##### Additional specimen examined.

Gabon – Ogooué-Maritime • M.S.M. Sosef 2261 (BR, K, LBV, MO, WAG), Doudou Mountains National Parc, c. 5 km S of Camp Peny (CBG); 2°03'30'S, 10°28'12'E; alt. 100 m; 14 Nov. 2005.

#### ﻿ Uvariodendron pycnophyllum

Taxon classificationPlantaeMagnolialesAnnonaceae

(Diels) R.E.Fr., Acta Horti Berg. 10: 58 (1930)

Figs 42 , 43

≡ Uvaria pycnophylla Diels, Bot. Jahrb. Syst. 53(3–5): 434 (1915). Type. Tanzania – Tanga • Brönnle 1876 (lectotype, here designated: FHO! (00060171); sheet from B lost or destroyed); Lushoto District, Amani; 5°06'S, 38°38'E; 20 Jul. 1908.

##### Description.

Tree 3–10 m tall, D.B.H. 20–30 cm; young branches glabrous, old branches glabrous; bark of trunk and branches reddish, external thin layers peeling off. Petiole 8–11 mm long, 1.7–3 mm wide, glabrous. Leaf lamina 158–282 mm long, 43–83 mm wide, length:width ratio 3–4.5, elliptic to oblong, coriaceous, base acute to slightly decurrent, apex attenuate to acuminate, acumen 7–13 mm long; surface above glabrous, surface below sparsely pubescent to glabrous when young, glabrous when old; midrib impressed above, raised below, glabrous above, glabrous below; secondary veins 11–17 pairs, weakly brochidodromous, impressed above, raised below; tertiary veins reticulate. Inflorescences borne on old branches or axillary, composed of 1 (sub)sessile flowers. Flower pedicel 0–10 mm long, ca. 4 mm in diameter, pubescent. Flowers bisexual, buds globose to ovoid, sessile, 7–10 mm high, 7.5–12 mm in diameter, pubescent. Bracts 2 to 6, up to 9 enclosing the bud, upper bract 10–13 mm long, 16–20 mm wide, depressed ovate to broadly ovate, adpressed, clasping the pedicel and sepals, pubescent outside, glabrous inside. Sepals 3, 10–12 mm long, ca 10 mm wide, fused at base, velutinous outside, glabrous inside, brown outside, yellow with red streak at base inside. Outer petals 3, 20–36 mm long, 16–19 mm wide, length:width ratio 1.1–2, elliptic to broadly obovate, free (valvate in bud), velutinous outside, glabrous inside, brownish to reddish-yellow outside, yellow with a pinkish-red streak from base up to 80% of the petal length inside. Inner petals 3, 20–31 mm long, ca. 15 mm wide, length:width ratio 1.9–2.1, obovate, valvate at apex (imbricate in bud), puberulent outside, glabrous inside, light yellow to cream, sometimes with a slight pinkish-red streak outside, pinkish-red to dark purplish-red with yellow margins inside. Stamens 1000 to 1200, ca. 3 mm long, ca. 0.4 mm wide, anthers linear, connective truncate, yellow. Carpels 30 to 40, 4.5 mm long, 1.2 mm wide, velutinous, free; stigma 1.5 mm long, 1.2 mm wide, coiled, velutinous, covered with an exudate at anthesis. Fruiting pedicel 0–5 mm long, 3.5 mm in diameter, velutinous. Monocarps 3 to 7, 19–35 mm long, 14–22 mm wide, length:width ratio ca 1.6–2.2, ovoid to cylindrical, pubescent, red with brown pubescence, only unripe fruits seen; sessile, stipe 0–1 mm long, 2.2 mm wide, puberulent. Seeds ca. 4 per monocarp, uniseriate, unknown.

**Figure 42.**
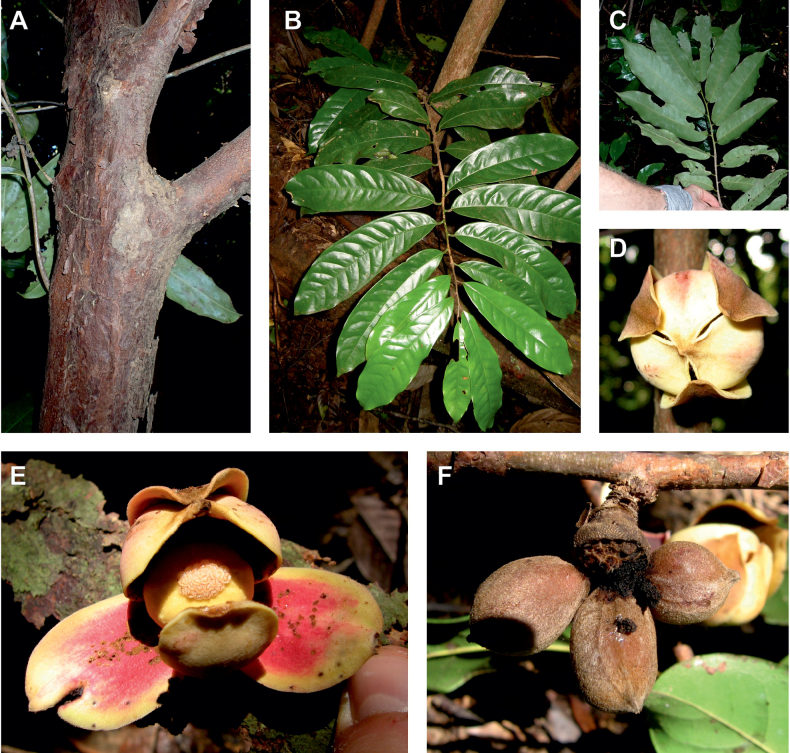
*Uvariodendronpycnophyllum* (Diels) R.E.Fr. **A** trunk, note the reddish peeling bark **B** young branch with leaves, upper side **C** young branch with leaves, lower side **D** young flower, top view **E** semi-open flower, top view **F** fruit, side view. **A** Dagallier 33 **B, D–F** Couvreur 21 **C** Dagallier 37. Photos **A, C** Léo-Paul Dagallier **B, D–F** Thomas Couvreur.

##### Distribution.

Endemic to Somalia-Masai Region: Tanzania.

##### Habitat and ecology.

Lowland and premontane mature rain forest. Soil: rocky or sandy soil (sandstone). Altitude: 400–1025 m a.s.l.

##### Phenology.

Flowers collected in November. Fruits collected in April, May and November.

##### Notes.

This species can be differentiated from other species by its bark being reddish, with external thin layers peeling off. The specimen Brönnle 1876 in B, defined as the type by Fries (1930) was not found. We thus define the specimen Brönnle 1876 in FHO as the lectotype.

**Figure 43.**
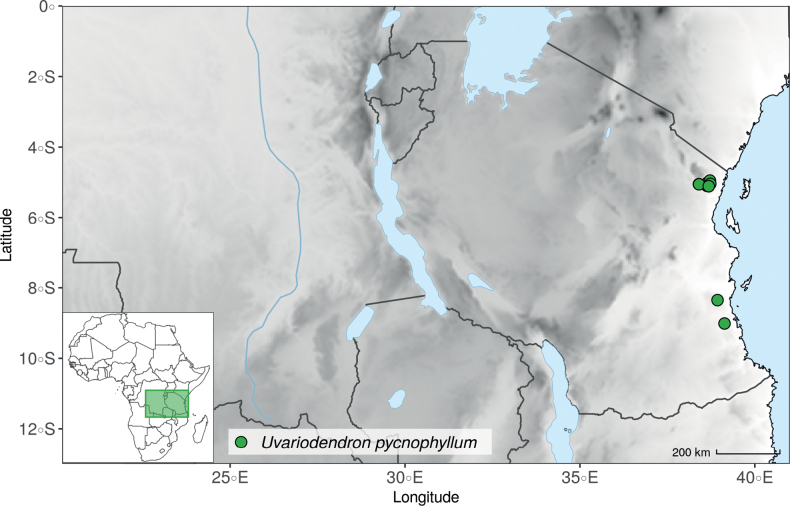
Distribution map of *Uvariodendronpycnophyllum*. Shades of grey represent elevation, from white (sea level) to darker grey (higher elevation). The inset shows the extent of the map over Africa.

##### Preliminary conservation status.

A previous assessment, that needs updating, listed this species as Endangered EN under criteria B1ab(iii) (Eastern Arc Mountains & Coastal Forests CEPF Plant Assessment Project 2009c). Here the EOO of is estimated at 9,926 km^2^ and AOO at 32 km^2^. It is known from 12 occurrences in Tanzania, mostly situated in the East Usambara Mountains. Only two of the occurrences of this species are within protected areas, namely the Mtai Forest Reserve and the Kiwengoma Forest Reserve. Most of the remaining occurrences are less than 5 km from other protected areas (e.g. Amani Forest Reserve, Nilo Forest Reserve), and it is thus possible that this species occurs in these protected areas. However, this would need to be verified, and given the uncertainty of the occurrence of this species in protected areas other than Mtai and Kiwengoma Forest Reserves, the number of locations and area of occupancy is likely to decrease in the future. Following IUCN criterion B, it is thus assigned a preliminary updated conservation status of Endangered EN 2ab(ii,iv).

##### Additional specimens examined.

Tanzania – Lindi • C.J. Kayombo 4558 (MO); Kilwa District, T8. NE end of Mbarawala Plateau, above valley running SE. Evergreen mixed dry rocky forest. Transect #3; 9°00'47'S, 39°07'59'E; alt. 280 m; 04 Nov. 2003 – Pwani – Frontier-Tanzania Coastal Forest Res.Prog 700 (K, MO); Rufiji District, Kiwengoma Forest. Northern edge of the Matumbi Highlands; 8°21'S, 38°56'E; alt. 365 m; 31 Jan. 1990 – Tanga • D.M. Johnson 1947 (DSM); Muheza District, along road Bombani-Kisiwani, ca 3 km from Kisiwani; 5°01'S, 38°38'24'E; alt. 400 m; 05 Jun. 1996 • F.M. Mbago 3366 (DSM); Muheza District, 3 km from Kisiwani to Bombani along the road; 5°06'38.38'S, 38°40'49.08'E; alt. 150 m; 18 May. 2005 • L.-P.M.J. Dagallier 33 (BR, DSM, K, MO, MPU, P, WAG); Muheza District, along the road to Amani Nature Reserve; 5°06'04.38'S, 38°40'30.44'E; alt. 359 m; 11 Nov. 2019 • L.-P.M.J. Dagallier 37 (DSM, K, MO, MPU, P, WAG); Muheza District, along the road to Amani Nature Reserve; 5°06'07.38'S, 38°40'16.12'E; alt. 378 m; 12 Nov. 2019 • L.B. Mwasumbi 112474 (DSM); Muheza District, T3: Kwamngumi Forest Reserve; 4°57'S, 38°43'E; 12 Mar. 1999 – Langheinrich FHDS2889 (B); Korogwe District, W Usambara Mts., Bungu; 5°03'S, 38°24'E; Jul. 1912 • M.A. Mwangoka 26 (MO); Korogwe District, (T3) West Usambara Mountains, riverine forest patch in Kieti Village; 5°03'21'S, 38°24'17'E; alt. 1025 m; 31 May. 2002 • M.A. Mwangoka 6168 (MO); Muheza District, East Usambaras mountains, Kiwanda village Forest (Mpangamanyoka forest). W part of the forest; 5°02'32'S, 38°43'38'E; alt. 310 m; 05 Apr. 2009 • T.L.P. Couvreur 21 (DSM, K, MO, WAG); Muheza District, on road 200 m before arriving to Kisiwani village; 5°06'28.8'S, 38°40'55.2"E; alt. 300 m; 12 Nov. 2006.

#### ﻿ Uvariodendron schmidtii

Taxon classificationPlantaeMagnolialesAnnonaceae

Q.Luke, Dagallier & Couvreur, PhytoKeys 174: 116 (2021)

Figs 44 , 45

##### Type.

Kenya – Coast • W.R.Q. Luke 3087 (holotype: EA! (EA000008814), isotypes: K!, MO, US); Kwale District, Shimba hills, Longo-Magandi; 4°14'S, 39°25'E; alt. 380 m; 20 Apr. 1992.

##### Description.

Tree 10–12 m tall, D.B.H. unknown; young branches sparsely pubescent to glabrate, old branches glabrous; leaf bud ‘eragrostiform’, composed of 5–7, ca. 10 mm long, 10 mm wide, distichous, longitudinally folded, velutinous scales. Petiole 4.5–7 mm long, 1.5–2 mm wide, sparsely puberulent to glabrate. Leaf lamina 159–188 mm long, 49–71 mm wide, length:width ratio 2.4–3.3, narrowly elliptic to elliptic, coriaceous, base acute to decurrent, apex attenuate to acuminate, acumen 16–20 mm long; surface above glabrous, surface below sparsely pubescent to glabrate when young, glabrous when old; midrib impressed above, raised below, glabrous above, pubescent to glabrous when young, glabrous when old below; secondary veins 10–14 pairs, brochidodromous to weakly brochidodromous, impressed above, raised below; tertiary veins reticulate. Inflorescences borne on trunk and branches, composed of 1–2 flowers. Flower pedicel 10–15 mm long, 2.5 mm in diameter, densely velutinous. Flowers bisexual, buds globose, subsessile to pedicellate, ca. 6 mm high, ca. 7 mm in diameter, pubescent. Bracts 1 to 2 between the lower 20–70% of the length of the pedicel, upper bract ca. 5 mm long, ca. 10 mm wide, depressed ovate, adpressed, semi-clasping the pedicel, velutinous outside, glabrous inside. Sepals 3, 5.5–7 mm long, 7–9 mm wide, fused at base for more than 50% of the length, forming a ring around the fruit pedicel, densely velutinous to velutinous outside, glabrous inside, brown outside, green inside. Outer petals 3, 11–12 mm long, 9–11 mm wide, length:width ratio 1.1–1.3, broadly obovate, velutinous to densely velutinous outside, glabrous inside, brown outside, cream with a bright reddish-pink streak at base inside. Inner petals 3, ca. 10 mm long, 8–9 mm wide, length:width ratio 1.1–1.3, broadly obovate, connivent at apex over ca. 50 % of the petal length, densely velutinous outside, glabrous inside, orangish-brown with cream margins and a bright reddish-pink streak at base outside, cream with a bright reddish-pink streak at base inside. Stamens 500 to 700, length and shape unknown. Carpels ca. 7, ca. 1.5 mm long, ca. 1 mm wide, velutinous, free; stigma coiled. Fruiting pedicel ca. 16 mm long, ca. 2.5 mm in diameter, pubescent. Monocarps 3 to 5, ca. 32 mm long, ca. 20 mm wide, length:width ratio ca. 1.6, rounded to ellipsoid, longitudinally ridged, sparsely pubescent, green turning orange; sessile, stipe ca. 0 mm long. Seeds unknown.

**Figure 44.**
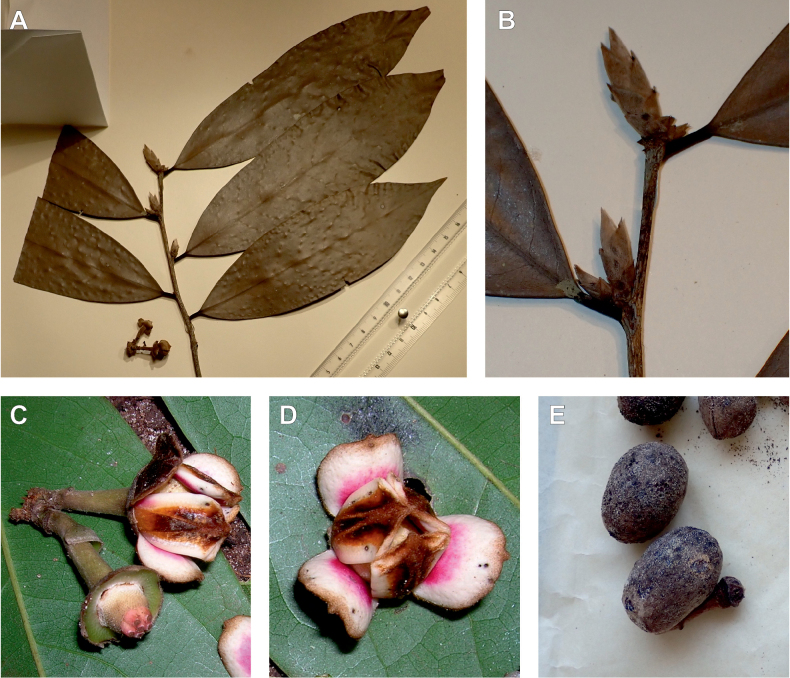
*Uvariodendronschmidtii* Q.Luke, Dagallier & Couvreur **A** branch with leaves, upper side **B** detail of branch apex, note the ‘eragrostiform’ buds **C** two-flowered inflorescence, side view, bottom flower has no petals, note the sepals fused in **A** cup-shaped calyx **D** flower, top view **E** loose monocarps, side view. **A, B, E** Luke 4717 **C, D** unknown material. Photos **A, B, E** Léo-Paul Dagallier **C, D** Quentin Luke.

##### Distribution.

Endemic to Somalia-Masai Region. Only known from one locality in Kenya: the Shimba Hills.

##### Habitat and ecology.

Lowland rain forest. Soil: deep red. Altitude: 380–450 m a.s.l.

##### Phenology.

Flowers collected in September and October. Fruits collected in April.

##### Notes.

This species differs from other *Uvariodendron* species by its flowers that are small (petals < 13 mm long), velutinous, on a 10–15 mm long pedicel, with fused sepals forming a ring around the fruit pedicel, and fewer than 10 carpels.

**Figure 45.**
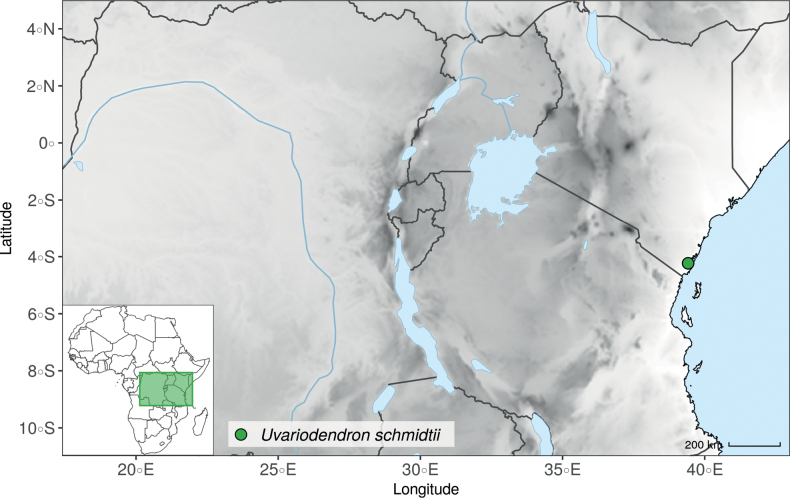
Distribution map of *Uvariodendronschmidtii*. Shades of grey represent elevation, from white (sea level) to darker grey (higher elevation). The inset shows the extent of the map over Africa.

##### Preliminary conservation status.

Following IUCN criterion D, this species has been assigned a preliminary status of Vulnerable VU (Dagallier et al. 2021).

##### Additional specimens examined.

Kenya – Coast • S.A. Robertson 7556 (EA, K, WAG); Kwale District, Shimba Hills NR, Longomandi forest; 4°14'S, 39°25'E; alt. 450 m; 04 Jun. 2005 • W.R.Q. Luke 2919 (EA); Kwale District, Shimba hills, Longo-Magandi; 4°14'S, 39°25'E; alt. 390 m; 15 Oct. 1991 • W.R.Q. Luke 4717 (EA, P); Kwale District, Shimba hills, Longo-Magandi; 4°14'S, 39°25'E; alt. 380 m; 12 Sep. 1997.

#### ﻿ Uvariodendron usambarense

Taxon classificationPlantaeMagnolialesAnnonaceae

R.E.Fr., Acta Horti Berg. 10: 65 (1930)

Figs 46 , 47

##### Type.

Tanzania – Tanga • G. Scheffler 226 (lectotype, here designated: EA! (EA000002471); isolectotypes: A! (A00039714), BM! (BM000554074), E! (E00704857, E00704858), EA! (EA000002470), P! (P00363394); B sheet lost or destroyed), E Usambara Mts., Derema; 5°05'S, 38°38'E; 26 Mar. 1900.

##### Description.

Tree 8–30 m tall, D.B.H. 17–30 cm; young branches pubescent to glabrous, old branches glabrous; the bark is dark on young individuals, peels on old individuals letting appear a pinkish-grey layer. Petiole 5–15 mm long, 1.5–6 mm wide, pubescent to glabrous. Leaf lamina 283–564 mm long, 79–180 mm wide, length:width ratio 2.4–3.9, obovate to oblong to elliptic, coriaceous, base rounded to subcordate, apex acuminate, acumen 5–18 mm long; surface above glabrous, surface below sparsely pubescent to glabrous when young, glabrous when old; midrib impressed above, raised below, glabrous above, sparsely pubescent to glabrous below; secondary veins 18–30 pairs, weakly brochidodromous, impressed above, raised below; tertiary veins reticulate. Inflorescences borne on trunk and old branches, composed of 1 flower. Flower pedicel unknown. Flowers bisexual, buds ovoid, sessile, ca. 10 mm high, ca. 6 mm in diameter, pubescent. Only old rotten flower seen. Bracts unknown, 1 to 3 bracts scars observed on fruiting pedicel. Sepals 3, length, shape and indumentum unknown. Outer petals 3, ca. 20–30 (?) mm long, ca. 10–20 (?) mm wide (measures taken on old and rotten flower), length:width ratio ovate, greenish yellow to brown with dark red streak from base up to 50% of the petal length inside. Inner petals 3, length, shape, indumentum and color unknown. Stamens unknown. Carpels unknown. Fruiting pedicel 12–22 mm long, 4–8 mm in diameter, pubescent to glabrous. Monocarps 1 to 17, 50–89 mm long, 18–27 mm wide, length:width ratio 1.8–3.6, cylindrical, pubescent to sparsely pubescent, pale yellowish green (unripe ?); stipe 1–5 mm long, 6–10 mm wide, pubescent to glabrate. Seeds 16–25 per monocarp, biseriate, 20 to 25 mm long, 12–14 mm wide.

**Figure 46.**
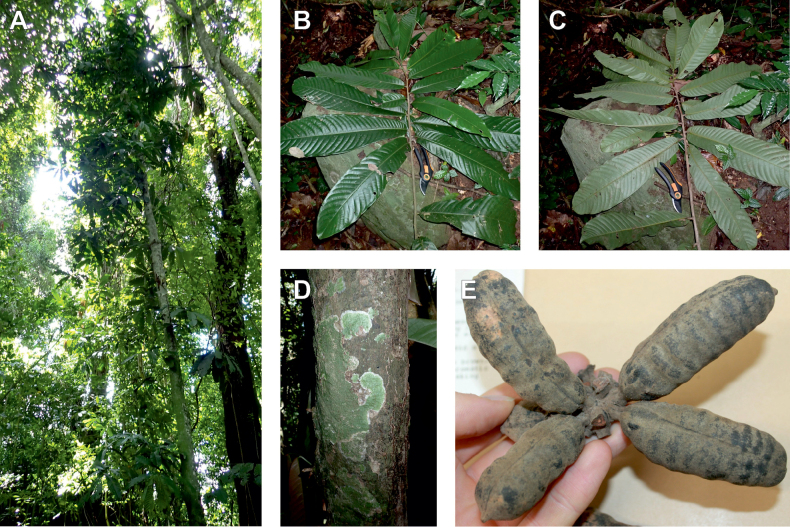
*Uvariodendronusambarense* R.E.Fr. **A** habit **B** branch with leaves, upper side **C** branch with leaves, lower side **D** trunk **E** dried fruit, top view. **A, D** specimen not collected **B, C** Dagallier 34 **E** Lovett 816. Photos Léo-Paul Dagallier.

##### Distribution.

Endemic to Somalia-Masai Region. Tanzania: Usambaras mountains and Udzungwa mountains.

##### Habitat and ecology.

Submontane to montane rain forest. Altitude: 961–1000 m a.s.l.

##### Phenology.

Flowers collected in August and November. Fruits collected from March to November.

##### Vernacular names.

Tanzania: ‘Mkenene’ in Kinguru (Greenway 8628, Swynnerton 1110), ‘Mkomboa’ in Kisamba (Mwangoka 1593), ‘Ntambileni’ in Kinguru (Mwangoka 3272).

##### Notes.

This species resembles *Ud.calophyllum*, *Ud.connivens*, *Ud.fuscum* and *Ud.molundense* in having great obovate to elliptic to oblong leaves (28–57 cm), but can be differentiated in having rounded to subcordate leaf bases (vs. acute to rounded), and a dark bark when young, peeling off when old, letting appear a pinkish-grey layer.

**Figure 47.**
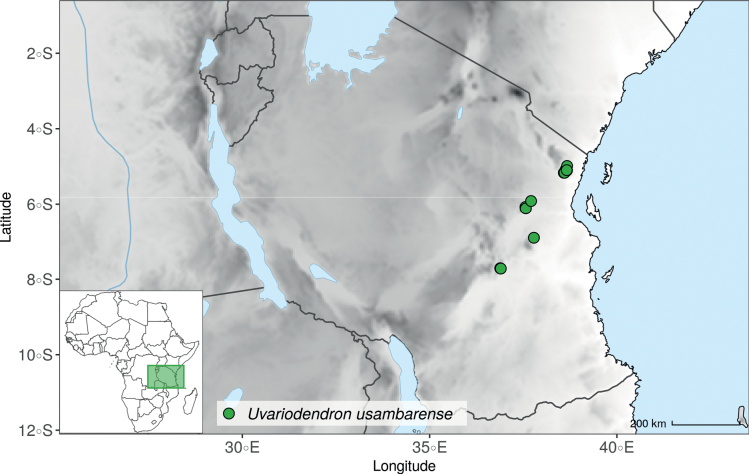
Distribution map of *Uvariodendronusambarense*. Shades of grey represent elevation, from white (sea level) to darker grey (higher elevation). The inset shows the extent of the map over Africa.

##### Preliminary conservation status.

A previous assessment, which needs updating, listed this species as Endangered EN under criteria B1ab(iii) (Eastern Arc Mountains & Coastal Forests CEPF Plant Assessment Project 2009d). Here the EOO is estimated at 13,513 km^2^ and AOO at 44 km^2^. It is known from 14 occurrences in Tanzania, situated in East Usambara Mountains, Uluguru Mountains and Udzungwa Mountains. Following IUCN criterion B, it would be assigned a preliminary conservation status of Endangered EN based solely on AOO, but no other subcriteria is met (b or c). Most of these occurrences are situated in protected areas such as the Udzungwa Mountains National Park and the Amani Nature Reserve. The future of this species relies on the future of these protected areas. It is thus assigned a preliminary updated conservation status of Near Threatened NT.

##### Additional specimens examined.

Tanzania – Morogoro • A.R. Marshall 1100 (K), Msolwa – PSP9. Udzungwa National Park. udzungwa Mountains; 7°42'30.36'S, 36°53'06.73'E; alt. 1124 m; 05 Aug. 2007 • B.J. Harris 4610 (DSM, K); Morogoro Rural District, Uluguru Mtns. near Kinole; 6°54'S, 37°47'E; alt. 900 m; 30 Apr. 1970 • C.F.M. Swynnerton 1110 (K); Morogoro Rural District, S Nguru Forest Reserve, Manyangu Forest, Liwale River; 6°07'S, 37°34'E; Feb. 1920 • M.A. Mwangoka 3272 (MA, MO); Morogoro Rural District, Nguru Mountains, Nguru South Forest Reserve, along valley between Mafuta and Ubili villages; 6°04'34'S, 37°33'33'E; alt. 555 m; 12 Aug. 2004 • P.J. Greenway 8628 (BM, K); Morogoro Rural District, S Nguru Forest Reserve, Manyangu Forest, Liwale River; 6°07'S, 37°34'E; alt. 760 m; 21 Aug. 1951 • W.R.Q. Luke 8737 (EA, K, NHT), Pt 303, Camp 244; 7°43'S, 36°54'E; alt. 900 m; 06 Jun. 2002 • Y.S. Abeid 2666 (EA, MO); Mvomero District, Kanga Forest Reserve, Submontane Forest, Work Unit 3, Base Camp; 5°55'S, 37°42'28'E; alt. 1140 m; 23 Mar. 2006 – Tanga • A.L. Borhidi 82031 (DSM, K); Lushoto District, Amani-Sigi Forest Reserve at the Sigi headwaters S of Amani near Kwamkoro sawmills; 5°10'S, 38°36'E; alt. 900 m; 19 Feb. 1982 • F.M. Mbago 3707 (DSM); Lushoto District, Amani, above Kisiwani village; 5°06'53.38'S, 38°39'16.51'E; alt. 961 m; 16 Oct. 2015 • I. Rajabu Hizza 185 (MO); Muheza District, T3. Amani Nature Reserve, phenology transect P-T 2, Kisiwani; 5°06'S, 38°40'E; alt. 400 m; 17 Nov. 1998 • J.C. Lovett 816 (DSM, K, MO); Muheza District, East Usambara mountains, Kwamkoro Forest Reserve. Area currently being logged by Sikh sawmills; 5°10'S, 38°35'E; alt. 950 m; 11 Jun. 1986 • L.-P.M.J. Dagallier 34 (BR, DSM, K, MO, MPU, P, WAG); Muheza District, Amani Nature Reserve; 5°06'31.38'S, 38°39'45.44'E; alt. 687 m; 12 Nov. 2019 • L.B. Mwasumbi 112429 (DSM), Kwamkoro N. R; 5°10'S, 38°36'E; 08 Nov. 1998 • M.A. Mwangoka 1593 (MO); Muheza District, Kwezitu public forest along path to peak between Mkalamo and Gonja Subvillages; 4°59'17'S, 38°40'12'E; alt. 825 m; 04 Aug. 2000 • T.L.P. Couvreur 20 (DSM, MO, WAG); Muheza District, c. 1 km straight up from Kisiwani village (just before Amani Nature Reserve); 5°05'55.2'S, 38°39'40.2"E; alt. 1000 m; 11 Nov. 2006.

#### ﻿ Uvariopsis

Taxon classificationPlantaeMagnolialesAnnonaceae

Engl., Notizbl. Königl. Bot. Gart. Berlin 2: 298 (1899)

= Tetrastemma Diels, Bot. Jahrb. Syst. 38(3): 241 (1906).
= Thonnera De Wild., Ann. Mus. Congo Belge, Bot. sér. 5, 3(1): 86 (1909).

##### Type species.

*Uvariopsiszenkeri* Engl.

##### Synoptic characters.

Shrubs to trees with flowers invariably exhibiting two sepals, in combination with at least one of the two following characters (and generally with both of them): flowers unisexual (plant monoecious) and flowers exhibiting one whorl of four free or basally fused petals.

##### Description.

Shrub to tree 1.5–30 m tall, D.B.H 1.5–39 cm; young branches pubescent to glabrous, old branches sparsely pubescent to glabrous. Petiole 1–9 mm long, 1–6 mm wide, pubescent to glabrous. Leaf lamina 73–615 mm long, 22–165 mm wide, length:width ratio 2.1–5, elliptic to oblong to obovate, papyraceous to coriaceous, base acute to decurrent to rounded to subcordate, apex acute to acuminate, acumen 0.5–30 mm long, surface above glabrous, surface below sparsely pubescent at base to glabrous when young, glabrous when old; midrib impressed above, raised below, glabrous above, sparsely pubescent to glabrous below; secondary veins 5–26 pairs, brochidodromous to weakly brochidodromous, impressed above, raised below; tertiary veins reticulate. Flowers unisexual or bisexual, male and female flowers similar or dimorphic, on same individuals (plant monoecious). Flower buds globose to long conical. Inflorescences borne in clumps of variable density on trunk, axillary or terminal, composed of 1 to 50 flowers. Peduncle inconspicuous to 2 mm long. Flower pedicel 0–430 mm long, 0.5–3 mm in diameter, pubescent to glabrous. Bracts 1 to 4, upper bract 0.5–4 mm long, 0.5–5 mm wide, triangular to broadly ovate, pubescent to sparsely pubescent outside, glabrous inside. Sepals 2, 0.7–18 mm long, 1–12 mm wide, triangular to broadly ovate, free to fused, pubescent to glabrous outside, glabrous inside, yellowish green to brown to dark red. Petals 3 to 4, 2.5–46 mm long, 1.5–22 mm wide, length:width ratio 0.9–5, elliptic to ovate, pubescent to glabrous outside, glabrous and soft to verrucose inside, cream to brown to dark red outside, cream to brown to dark red inside. Stamens 100 to 900, 0.1–2 mm long, 0.1–0.5 mm wide, anthers linear, connective prolongation truncate or absent. Carpels 3 to 280, 1–5 mm long, 0.5–3 mm wide, pubescent to glabrous, free; stigma 0.1–0.6 mm long, 0.2–1 mm wide, flat to coiled to globose, glabrous. Fruiting pedicel 2–400 mm long, 1–7 mm in diameter, pubescent to glabrous. Monocarps, 1–25, 10–85 mm long, 4.5–55 mm wide, length:width ratio 1.1–3.7, cylindrical, smooth to verrucose, straight to constricted between the seeds, pubescent to glabrous, dull olive green to red to brown, sessile to stipitate; stipe 0–14 mm long, 1–6 mm wide, pubescent to glabrous. Seeds 2–16 per monocarp, uniseriate to biseriate, 8–25 mm long, 5–15 mm wide, ellipsoid to semicircular.

### ﻿Key to *Uvariopsis* species

**Table:** 

1	Largest leaves with lamina < 18 cm long, leaves with base acute to decurrent, flowers with petals < 16 mm long	**2**
–	Largest leaves with lamina > 18 cm long, leaves with base acute or rounded or subcordate or decurrent, flowers with petals ≥ or < 16 mm long	**6**
2	Leaves with base invariably decurrent, flowers bisexual with 3 to 6 carpels (Tanzania)	***Up.bisexualis***
–	Leaves with base acute to decurrent, flowers unisexual with more than 13 carpels	**3**
3	Young branches and petioles densely to sparsely pubescent, petals ovate to triangular, fused at base over ca. 50% of their length (Cameroon)	***Up.zenkeri***
–	Young branches glabrous or very sparsely pubescent, petals broadly ovate to ovate, free at base	**4**
4	Flowers with 13 to 20 carpels, fruits composed of 1 to 3 monocarps, not constricted between the seeds and pubescent to sparsely pubescent (West Africa)	***Up.oligocarpa***
–	Flowers with 20 to 40 carpels, fruits composed of 1 to 15 monocarps, strongly constricted between the seeds and glabrate to glabrous (Central and East Africa)	***Up.congensis* (5)**
5	Shrub to tree 2–6 m tall, leaves elliptic to obovate with a length:width ratio between 2.1 and 3.1 (Central Africa)	**Up.congensisvar.congensis**
–	Shrub to tree 7–15 m tall, leaves narrowly elliptic with a length:width ratio between 3 and 4 (Central and East Africa)	**Up.congensisvar.angustifolia**
6	Larger leaves > 31 cm long, leaves with base rounded to subcordate or cordate	**7**
–	Larger leaves ≤ 31 cm long, leaves with base acute to decurrent, or acute to rounded to subcordate	**10**
7	Leaves emitting a strong lemon scent when crushed, flowering pedicel ≤ 2 mm long (Central Africa)	***Up.citrata***
–	Leaves not emitting lemon scent when crushed, flowering pedicel > 2 mm long	**8**
8	Female and male flowers with flowering pedicel ≤ 8 mm, petals narrowly ovate to linear, always free (Central Africa)	***Up.bakeriana***
–	Female flowers with pedicel ≥ 20 mm, petals ovate to narrowly ovate, free to fused	**9**
9	Largest leaves with lamina ≤ 38 cm long, female flowers with sepals ≥ 6 mm long, female and male flowers with petals pinkish to dark red inside and outside (Cameroon)	***Up.submontana***
–	Largest leaves with lamina > 38 cm long, female flowers with sepals ≤ 5 mm long, female and male flowers with petals cream to brownish outside and cream to pinkish inside (Central Africa)	***Up.korupensis***
10	Petals fused and female flowers with pedicel ≥ 8 cm	**11**
–	Petals free and female flower with pedicels < 20 cm, or petals fused and female flowers with pedicels ≤ 6.5 cm	**12**
11	Flowers with 3 petals, ≤ 40 carpels, monocarps stipitate (stipe 3–4 mm long) sparsely pubescent to glabrous with ca. 4 longitudinal ridges (Central Africa)	***Up.congolana***
–	Flowers with 4 petals, ≥ 50 carpels, monocarps sessile pubescent and verrucose (Central Africa)	***Up.pedunculosa***
12	Leaf base acute to decurrent	**13**
–	Leaf base acute and minutely cordate or acute to rounded to subcordate	**14**
13	Flowering pedicel ≤ 10 mm, ca. 20 carpels (Cameroon)	***Up.etugeana***
–	Flowering pedicel ≥ 14 mm, > 30 carpels (Tanzania)	***Up.lovettiana***
14	Leaf base acute and minutely cordate, inflorescence with peduncle ca. 2 mm long and 2 mm wide (Cameroon)	***Up.dicaprio***
–	Leaf base acute to rounded to subcordate, inflorescence with peduncle inconspicuous	**15**
15	Young branches and petiole tomentose to shortly tomentose (Central Africa)	***Up.solheidii* (16)**
–	Young branches and petiole pubescent to glabrous	**17**
16	Male flowers with petals 7–10 mm long, with a l:w ratio of ca. 3; female flowers with pedicel 63–198 mm long and petals 9–17 mm long with a l:w ratio > 1.8; petals curved outward at anthesis (Central Africa)	**Up.solheidiivar.solheidii**
–	Male flowers with petals 5–7 mm long, with a l:w ratio of ca. 2, female flowers with pedicel 20–110 mm long and petals 7.5–10 mm long with a l:w ratio > 1.8; petals straight at anthesis (Gabon)	**Up.solheidiivar.letestui**
17	Flowers borne in clumps of 5 to 20 flowers on thickenings of the trunk, ≥ 100 carpels (Central Africa)	***Up.dioica***
–	Flowers borne in inflorescences of 1 to 2 flowers on the trunk or axillary or terminal, ≤ 50 carpels	**18**
18	Petals ovate with a l:w ratio ≥ 1.8, brownish red outside, cream inside; male flowers with pedicel ≤ 4 mm; female flower pedicel ≥ 70 mm (Angola)	***Up.noldeae***
–	Petals broadly ovate with a l:w ratio ≤ 1.4, pale greenish to cream outside, cream with purplish base inside; male flowers with pedicel ≥ 5 mm; female flower pedicel ≤ 65 mm (West Africa)	***Up.guineensis* (19)**
19	Flowers with petals fused at base over 30–50% of their length and 20 to 50 carpels; male flowers with petals 8–17 mm long and 9–17 mm wide (West Africa)	**Up.guineensisvar.guineensis**
–	Flowers with petals free and 10 to 20 carpels; male flowers with petals 7–10 mm long and 5–9 mm wide (Ghana)	**Up.guineensisvar.globiflora**

### ﻿Species descriptions

#### ﻿ Uvariopsis bakeriana

Taxon classificationPlantaeMagnolialesAnnonaceae

(Hutch. & Daltz.) Robyns & Ghesq., Ann. Soc. Sci. Bruxelles, Ser. B liii. 320 (1933)

**Figure 48.**
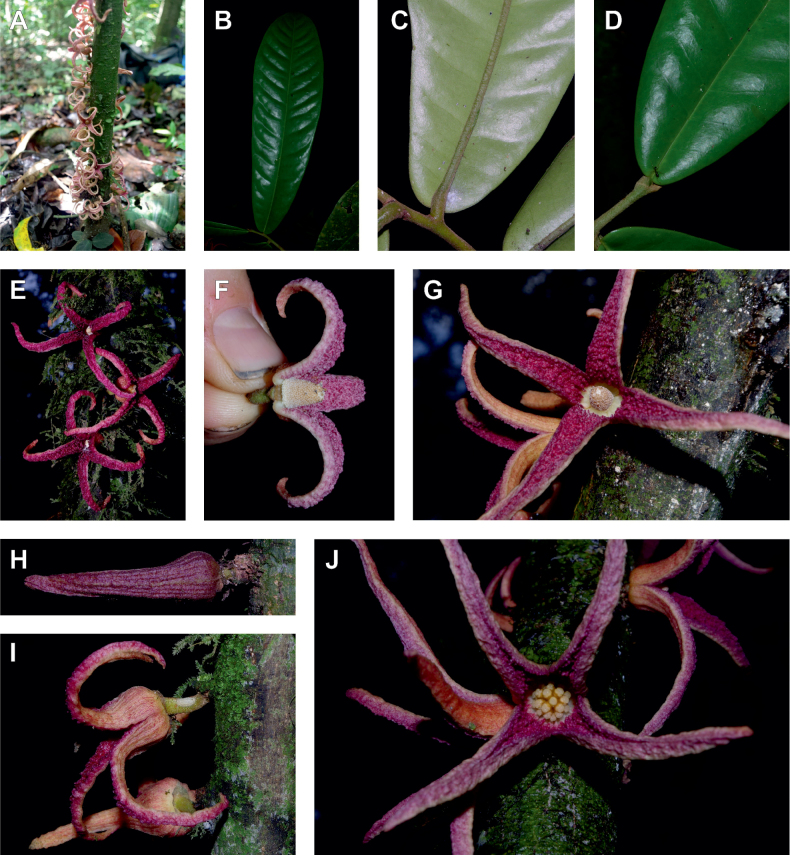
*Uvariopsisbakeriana* (Hutch. & Daltz.) Robyns & Ghesq **A** trunk with flowers **B** leaf, upper side **C** leaf base, lower side **D** leaf base, upper side **E** flowers borne on trunk **F** male flower, one petal removed, side view **G** male flower, top view **H** flower bud, side view **I** flower, side view **J** female flower, top view. **A–D, F, H** Couvreur 1000 **E, I** Couvreur 1045 **G, J** Couvreur 1015. Photos Thomas Couvreur.

**Figure 49.**
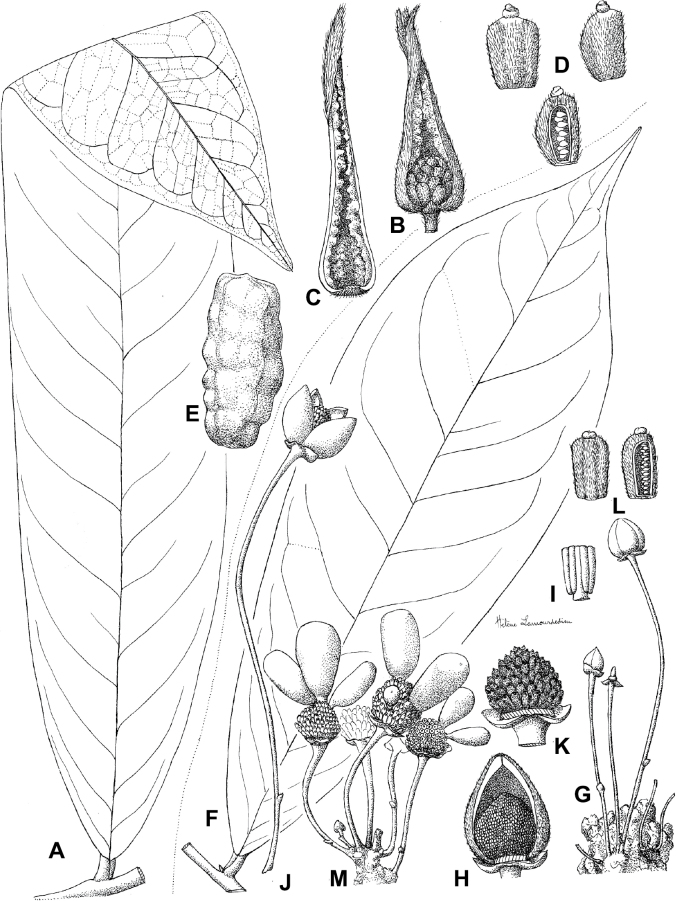
*Uvariopsisbakeriana* (Hutch. & Daltz.) Robyns & Ghesq **A** leaf, top view **B** detail of female flower, two petals removed **C** petal, inner view **D** carpel, side view, front view and detail of ovules **E** monocarp *Uvariopsisdioica* (Diels) Robyns & Ghesq **F** leaf, top view **G** male flowering pedicels and flowers **H** detail of male flower, two petals removed **I** stamen **J** female flowering pedicel **K** detail of female flower, all four petals removed **L** carpel, front view and detail of ovules **M** fruits. **A–E** from Brenan 9409 **F–L** from Keay 28066 **M** from Letouzey 4230. Drawings by Hélène Lamourdedieu, Publications Scientifiques du Muséum national d’Histoire naturelle, Paris.

**Figure 50.**
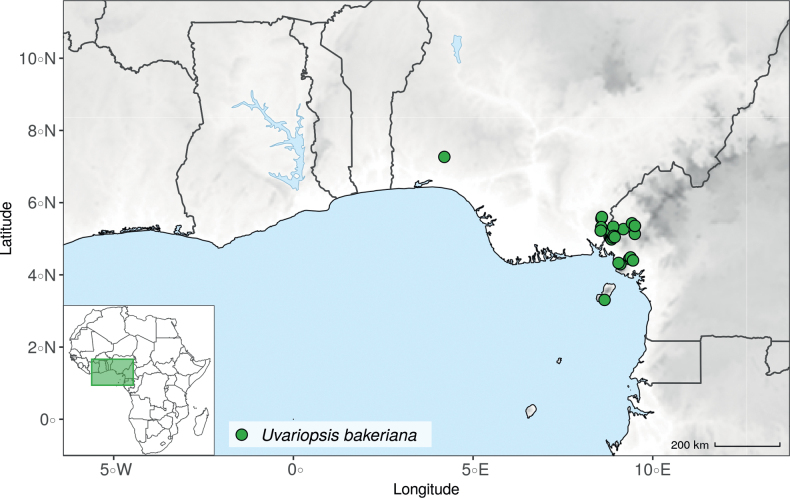
Distribution map of *Uvariopsisbakeriana*. Shades of grey represent elevation, from white (sea level) to darker grey (higher elevation). The inset shows the extent of the map over Africa.

Figs 48 , 49A–E , 50 ; Table 5

≡ Tetrastemma bakerianum Hutch. & Dalziel, Kew Bull. 1927: 153 (1927) & Fl. W. Trop. Afr. I. 57 (1927). Type. Nigeria – Cross River State • P.A. Talbot 1517 (holotype: K! (K000199043); isotype: BM! (BM000554076)); Calabar, Oban; 5°19'N, 8°34'E; 1912.

##### Description.

Tree 3–7 m tall, D.B.H 1.8–8 cm; young branches pubescent to puberulent, old branches sparsely pubescent to glabrous. Petiole 2–6 mm long, 2–4 mm wide, pubescent to glabrous. Leaf lamina 150–340 mm long, 40–90 mm wide, length:width ratio (2.27) 3.3–5, oblong to obovate, coriaceous, base rounded to subcordate, apex acuminate, acumen 6–32 mm long, surface above glabrous, surface below glabrous; midrib impressed above, raised below, glabrous above, sparsely pubescent to glabrous below; secondary veins 10–26 pairs, brochidodromous to weakly brochidodromous, impressed above, raised below; tertiary veins reticulate. Flowers unisexual, male and female flowers similar, on same individuals (plant monoecious). Flower buds long conical. Male and female inflorescences borne on trunk, composed of 1 to 2 flowers. Peduncle inconspicuous. Flower pedicel 2–8 mm long, 0.5–2 mm in diameter, pubescent. Bracts 1 (2) at base of the pedicel, 1–2 mm long, 1–4 mm wide, triangular, pubescent outside, glabrous inside. Sepals 2, 1–3.5 mm long, 1.5–3 mm wide, triangular, free, pubescent outside, glabrous inside, brown. Petals 4, 24–46 mm long, 3–8 mm wide, length:width ratio 4–10, narrowly ovate to linear, valvate, pubescent to sparsely pubescent outside, glabrous and highly verrucose inside, light red to dull yellowish brown outside, bright pink to dark pinkish red inside. Male flowers: stamens 400 to 600, 0.1–0.6 mm long, 0.1–0.3 mm wide, anthers linear, connective prolongation truncate or absent. Female flowers: carpels 15 to 40, 1.5–2.5 mm long, 1–1.5 mm wide, densely pubescent, free; stigma coiled. Fruiting pedicel 5–12 mm long, 2–2.5 mm in diameter, pubescent. Monocarps, 2–5, 21–50 mm long, 15–23 mm wide, length:width ratio 1.2–3.5, cylindrical, wrinkled, pubescent to glabrous, bright red, sessile to shortly stipitate; stipe up to 4 mm long, 1–3 mm wide, pubescent. Seeds 6–16 per monocarp, biseriate, 15–22 mm long, 8–12 mm wide, ellipsoid.

##### Distribution.

Endemic to Lower Guinean Domain of the Guineo-Congolian Region: Cameroon, Equatorial Guinea (Bioko island), Nigeria.

##### Habitat and ecology.

Lowland mature or old secondary rain forests. Soil: generally sandy. Altitude: 50–800 m asl.

##### Phenology.

Flowers collected from January to April. Fruits collected from March to June.

##### Notes.

This species resembles *Up.citrata*, *Up.korupensis* and *Up.submontana* in having large obovate leaves (up to 35 cm long), with rounded to subcordate bases. *Up.bakeriana* differs in having short flower pedicel (2–8 mm, vs. 0–2 mm in *Up.citrata*, 6–70 mm in *Up.korupensis* and 25–60 mm in *Up.submontana*), and with its petals being narrowly ovate to linear, with a length:width ratio comprised between 4 and 10 (vs. ca. less than 5 in *Up.citrata* and *Up.submontana*), free (vs. fused in *Up.korupensis*), and bright pinkish to deep red petals (vs. cream to brownish in *Up.citrata* and *Up.korupensis*) (Table 5).

**Table 5.** The ‘large leaves group’: morphological comparison between *Uvariopsisbakeriana*, *Uvariopsiscitrata*, *Uvariopsiskorupensis* and *Uvariopsissubmontana*. In bold: character useful to differentiate the species. l:w = length:width.

	Lamina	Flower pedicel	Sepals	Petals	Altitudinal range
*Uvariopsisbakeriana*	150–340 mm long	2–8 mm long	Male and female flowers: 1–3.5 mm long, 1.5–3 mm wide, **free**	Male and female flowers: **l:w ratio 4–10**, narrowly ovate to linear, free, valvate, **bright pink to dark pinkish red**	50–800 m above sea level
*Uvariopsiscitrata*	312–500 mm long, **strong lemon** scent when crushed	**0–2 mm long**	Male and female flowers: 9–15 mm long, 4–6 mm wide, basely fused	Male and female flowers: l:w ratio ca. 3.5, ovate, **greenish yellow**	60–300 m above sea level
*Uvariopsiskorupensis*	280–615 mm long	6–70 mm long	Male flowers: **1–5 (7.5) mm long**, 2–6.5 mm wide, basely fused	Male flowers: l:w ratio 2.5–7, narrowly ovate, fused at base	90–160 m above sea level
			Female flowers: **3–5 mm long, 4–5 mm wide**, basely fused	Female flowers: **petals more than 3 times longer than the sepals**, l:w ratio 7–12, narrowly ovate, fused at base, **cream to pinkish**	
*Uvariopsissubmontana*	160–380 mm long	24–60 mm long	Male flowers: **5–11 mm long, 6–12 mm wide**, basely fused	Male flowers: l:w ratio 1.8–4.7, ovate to narrowly ovate, free to fused at base over 30% of their length	**900–1300 m** above sea level
			Female flowers: **6–8 mm long, 6–9 mm wide**, basely fused	Female flowers: **petals less than 3 times longer than the sepals**, l:w ratio 5–9, narrowly ovate, fused at base, ovate to narrowly ovate, free to fused at base over 30% of their length, **cream to pinkish**	

##### Conservation status.

This species has been assessed as Least Concern LC (Cosiaux et al. 2019b). It is known from several occurrences in western Cameroon, Bioko Island (Equatorial Guinea) and eastern Nigeria, and from only one occurrence in western Nigeria. Here we estimate its EOO at 68,842 km^2^ and its AOO at 92 km^2^.

##### Additional specimens examined.

Cameroon – Littoral • D.W. Thomas 4300 (K, MO), Korup National Park. Forest along footpath from Ndian River at PAMOL field 69 and transect P; 5°01'N, 8°50'E; alt. 50 m; 24 Jan. 1985 – South-West Region • C. Farron 7297 (P), South Bakundu Forest, Kendongi; 4°26'30.63'N, 9°20'43.88'E; 14 May. 1970 • C.F.A. Onochie 9305 (K), South Bakundu Forest; 4°24'06.26'N, 9°26'32.16'E; 12 Mar. 1948 • D.W. Thomas 1086 (K); Ndian, Korup Forest Reserve, Korup Reserve, path between transect P&Q; 4°59'N, 8°51'E; Mar. 1979 • D.W. Thomas 3210 (K, MO), forest in the Korup National Park; 5°03'N, 8°48'E; alt. 50 m; 28 Feb. 1984 • D.W. Thomas 3336 (MO), North-eastern corner of Korup National Park; near Baro village; 5°16'N, 9°11'E; alt. 200 m; 24 Mar. 1984 • D.W. Thomas 5606 (MO), along transect P, southern end of Korup National Park; 5°11'N, 8°51'E; alt. 50 m; 16 Feb. 1986 • D.W. Thomas 7835 (MO), Ajaman, near northern edge of Korup National Park; 5°20'N, 8°54'E; alt. 200 m; 22 May. 1988 • G.K. Gottsberger 100307/11 (ULM, WAG), c. 100 m from Banyang Mbo Research Station; 5°08'N, 9°30'E; 10 Mar. 2007 • J.P.M. Brenan 9305 (K), South Bakundu Forest; 4°29'N, 9°23'E; 12 Mar. 1948 • J.P.M. Brenan 9409 (BM, K), Banga, S. Bakundu F.R; 4°24'N, 9°27'E; 13 Mar. 1948 • M.R. Cheek 7234 (K, SCA, YA); Ndian, Mundemba, Korup National Park, nature trail near suspension bridge.; 4°59'N, 8°51'E; alt. 50 m; 01 Feb. 1995 • R.E. Gereau 5195 (MA, MO, WAG); Ndian, 2–2.5 km S of Six Cup Garri Creek; 5°02'N, 8°53'E; alt. 100 m; 06 Mar. 1993 • R.G. Letouzey 13841 (P), entre Etinkem et Nfaitok, 10 km N Nguti; 5°25'39.72'N, 9°25'14.26'E; 15 Jun. 1975 • S. Cable 2150 (K, YA); Ndian, Korup National Park, Ekundu Kundu, path to Ekon 1, 5 km from Ekundu-Kundu; 5°04'50.32'N, 8°54'29.81'E; alt. 300 m; 25 Apr. 1996 • S. Cable 2256 (K, YA); Ndian, Korup National Park, path from Ekundu Kundu to Faba, about 3 km; 5°02'53.41'N, 8°56'25.12'E; alt. 300 m; 27 Apr. 1996 • T.L.P. Couvreur 1000 (MPU, WAG, YA), Bayang Mbo Wildlife Sanctuary, after Mbu river; 5°21'09.69'N, 9°30'09.23'E; alt. 259 m; 25 Mar. 2016 • T.L.P. Couvreur 1015 (MPU, WAG, YA), Bayang Mbo Wildlife Sanctuary, after Mbu river; 5°21'22.04'N, 9°30'05.8'E; alt. 250 m; 26 Mar. 2016 • T.L.P. Couvreur 1045 (MPU, WAG, YA), Mount Cameroon National Park, on the Bomona trail, behind Bomona village, 10 km NW from Idenau; 4°17'41.96'N, 9°05'53.93'E; alt. 856 m; 03 Apr. 2016 • W. Mukete 6 (K, MO, SCA, YA), Kosse, along the path to the Reserve; 4°19'48.72'N, 9°02'52.08'E; 24 Apr. 1996. Equatorial Guinea – Bioko Sur • W.R.Q. Luke 11907 (K, MA), Moaba. Pt 140-141; 3°18'20.6'N, 8°39'33.8'E; alt. 8 m; 15 Mar. 2007. Nigeria – Cross River State • C.F.A. Onochie FHI36140X (K); Calabar, Oban Group Forest Reserve, Orem; 5°36'N, 8°35'E; 23 Jan. 1957 • J. Ntui 944 (MO), Oban Hills; 5°33'30'N, 8°34'12'E; alt. 600 m; 19 Dec. 1955 • M.C. Ejiofor FHI21870 (K); Calabar, Oban Group Forest Reserve; 5°36'N, 8°35'E; 24 Apr. 1952 • P.A. Talbot 1516 (BM); Calabar, Oban; 5°19'N, 8°34'E; 1912 • P.A. Talbot 446 (BM), Oban; 5°13'23.28'N, 8°33'06.9'E; 1909 • P.W. Richards 5166 (K, K, P); Calabar, Oban; 5°13'27.54'N, 8°33'10.42'E; alt. 244 m; 13 Mar. 1955 – Ogun State • A.P.D. Jones FHI17275 (P), c. 1/2 ml W. of Oaho Enclave, E.B. 34 Line 7. Road trace of U.A.C; 7°16'N, 4°12'E; 05 Apr. 1946.

#### ﻿ Uvariopsis bisexualis

Taxon classificationPlantaeMagnolialesAnnonaceae

Verdc., Kew Bull. 41(2): 289 (1986)

**Figure 51.**
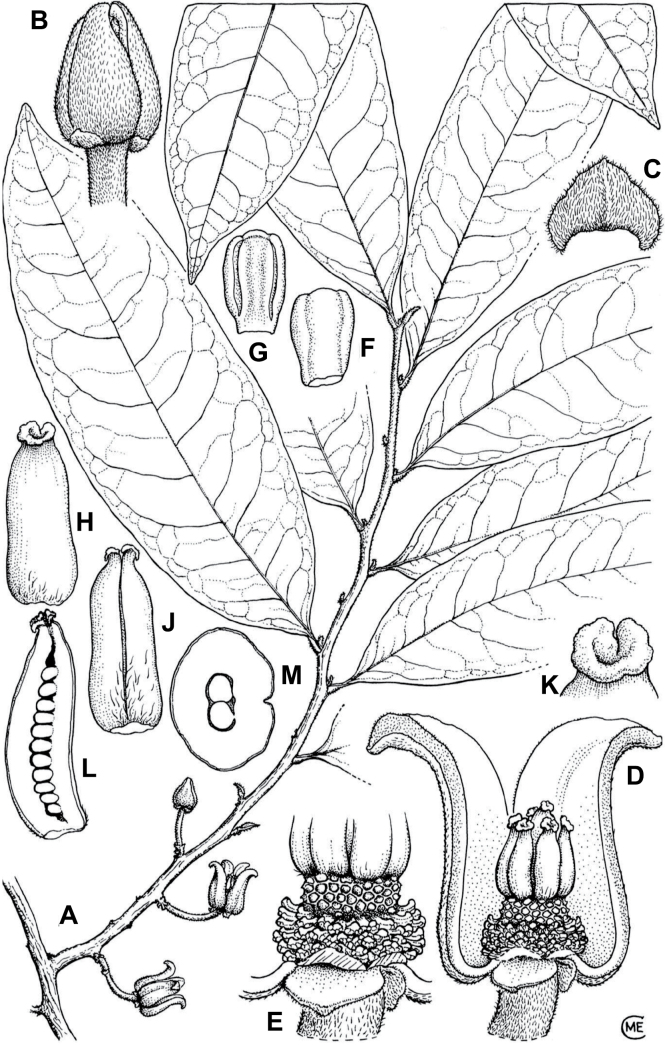
*Uvariopsisbisexualis* Verdc **A** flowering branch **B** flower bud, side view **C** sepal, outer view **D** flower, two petals removed, side view **E** detail of receptacle, some stamens fallen, side view **F** stamen, upper view **G** stamen, lower view **H** carpel, outer view **J** carpel, inner view **K** stigma **L** longitudinal section of carpel **M** transversal section of ovary. **A–M** from Lovett 233 (type). Drawings by Maureen Church, from Verdcourt (1986; fig. 3, p. 292), Kew Bulletin 1969, © Board of Trustees of the Royal Botanic Gardens, Kew.

**Figure 52.**
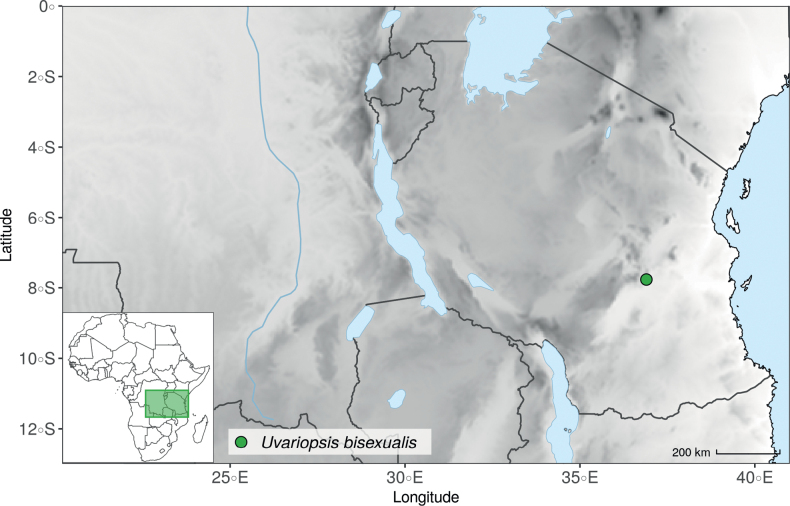
Distribution map of *Uvariopsisbisexualis*. Shades of grey represent elevation, from white (sea level) to darker grey (higher elevation). The inset shows the extent of the map over Africa.

Figs 51 , 52 ; Table 6

##### Type.

Tanzania – Iringa • J.C. Lovett 233 (holotype: K! (K000199040)); Iringa Rural District, Udzungwa Mts., Sanje; 7°46'S, 36°54'E; alt. 1480 m; 02 Dec. 1983.

##### Description.

Tree ca. 10 m tall, D.B.H unknown; young branches pubescent, old branches glabrous. Petiole 3.5–5 mm long, ca. 2 mm wide, sparsely pubescent to glabrous. Leaf lamina 135–152 mm long, 37–48 mm wide, length:width ratio ca. 3, elliptic to oblong, coriaceous, base decurrent, apex attenuate, surface above glabrous, surface below sparsely pubescent to glabrous when young, glabrous when old; midrib impressed above, raised below, glabrous above, sparsely pubescent to glabrous below; secondary veins ca. 12 pairs, weakly brochidodromous, impressed above, raised below; tertiary veins reticulate. Flowers bisexual. Flower buds ovoid to conical. Inflorescences borne on branches, composed of 1 flower. Peduncle inconspicuous. Flower pedicel 12–20 mm long, ca. 1 mm in diameter, pubescent. Bracts 1 at base to towards the lower half of the pedicel, ca. 1 mm long, ca. 1 mm wide. Sepals 2, 1.6–2.5 mm long, 2.2–2.5 mm wide, broadly ovate, free, pubescent outside, glabrous inside, color unknown. Petals 4, 12–14 mm long, 4–8 mm wide, length:width ratio 3–3.5, narrowly ovate, sparsely pubescent outside, glabrous inside, cream outside, cream with red base inside. Stamens numerous (exact number unknown), 0.5–0.7 mm long, ca. 0.5 mm wide, anthers linear, connective prolongation truncate or absent. Carpels 3 to 6, 2–4 mm long, 1–1.4 mm wide, glabrate at base to glabrous, free, ovules 22–24 per ovary, biseriate. Fruits unknown.

##### Distribution.

Endemic to Somalia-Masai Region. Known from only one locality in Tanzania: Sanje in the Udzungwa mountains.

##### Habitat and ecology.

Montane rain forests. Altitude ca. 1480 m asl.

##### Phenology.

Flowers collected in December.

##### Notes.

This species resembles *Up.congensis*, *Up.oligocarpa* and *Up.zenkeri* in having elliptic leaves generally less than 16 cm long with decurrent base. It differs from all the other *Uvariopsis* in having bisexual flowers and less than 6 carpels (vs. more than 10 carpels) (Table 6).

**Table 6.** Morphological comparison between *Uvariopsisbisexualis*, *Uvariopsiscongensis*, *Uvariopsisoligocarpa* and *Uvariopsiszenkeri*. In bold: character useful to differentiate the species.

	Young branches indumentum	Flowers	Carpels number	Fruits
*Uvariopsisbisexualis*	pubescent	**bisexual**	3 to 6	unknown
*Uvariopsiscongensis*	sparsely pubescent to glabrous	unisexual	20 to 40	1 to 15 monocarps, cylindrical, smooth to lumpy, **strongly constricted between the seeds**, **glabrate to glabrous**, green to **orange to red** when ripe (**black when dry**), sessile to stipitate with stipe 0–7 mm long
*Uvariopsisoligocarpa*	glabrous	unisexual	**13 to 20**	**1–3 monocarps**, cylindrical, smooth, **pubescent to sparsely pubescent**, green to **yellow to orange** when ripe (**orange-brown when dry**), sessile to very shortly stipitate with stipe 0–1 mm long
*Uvariopsiszenkeri*	**densely pubescent to pubescent**	unisexual	13 to 22	**1–3 monocarps**, cylindrical, slightly veined and constricted between the seed when dried, **tomentose**, brown, subsessile with stipe ca. 1 mm long

##### Conservation status.

This species has been assessed as Endangered EN under criteria B1ab(iii) (Eastern Arc Mountains & Coastal Forests CEPF Plant Assessment Project 2009e). It is known from a single specimen collected more than 30 years ago in the Udzungwa Mountains National Park in Tanzania.

#### ﻿ Uvariopsis citrata

Taxon classificationPlantaeMagnolialesAnnonaceae

Couvreur & Niang., PhytoKeys 68: 3 (2016)

**Figure 53.**
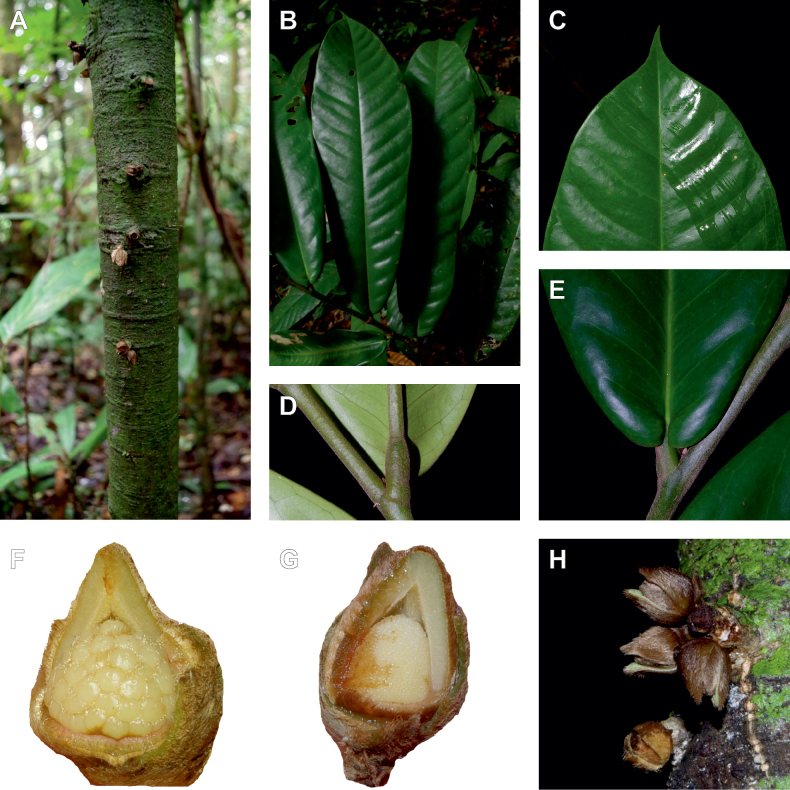
*Uvariopsiscitrata* Couvreur & Niang **A** trunk with flower buds **B** leaves, upper side **C** leaf base, lower side **D** lear apex, upper side **E** leaf base, upper side **F** female flower bud, two petals removed, side view **G** male flower bud, one petal removed, side view **H** flower buds on trunk. **A–E, H** Couvreur 1143 (type) **F, G** Couvreur 1126. Photos Thomas Couvreur.

**Figure 54.**
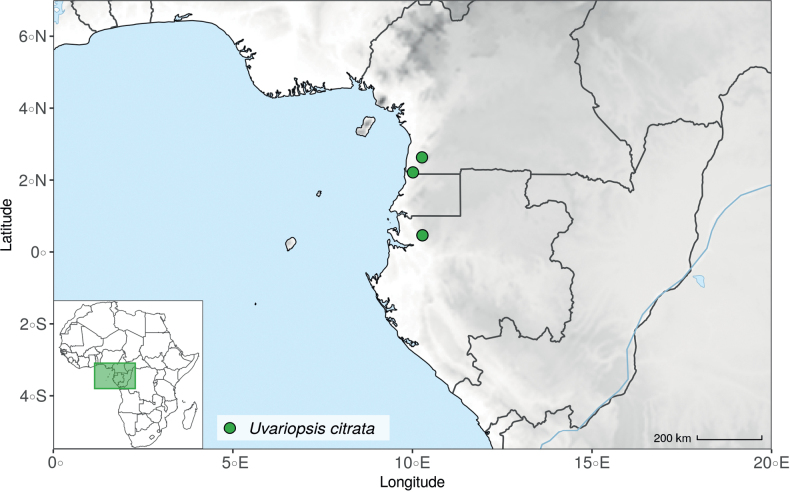
Distribution map of *Uvariopsiscitrata*. Shades of grey represent elevation, from white (sea level) to darker grey (higher elevation). The inset shows the extent of the map over Africa.

Figs 53 , 54 ; Table 5

##### Type.

Gabon – Estuaire • T.L.P. Couvreur 1143 (holotype: WAG! (no barcode); isotypes: LBV, P!), Monts de Cristal National Park, Mbé sector, 800 m from Kinguélé ANPN camp, near bridge; 0°27'50.18'N, 10°16'42.82'E; 14 Jun. 2016.

##### Description.

Tree 4–10 m tall, D.B.H 3–10 cm; young branches pubescent, old branches glabrous. Leaves strong lemon scent when crushed. Petiole 4–8 mm long, 3–5 mm wide, pubescent to glabrous. Leaf lamina 312–500 mm long, 88–120 mm wide, length:width ratio 3.5–4.3, elliptic to obovate, coriaceous, base subcordate, apex acuminate, acumen 20–30 mm long, surface above glabrous, surface below glabrous; midrib impressed above, raised below, glabrous above, sparsely pubescent to glabrous below; secondary veins 17–19 pairs, brochidodromous to weakly brochidodromous, impressed above, raised below; tertiary veins reticulate. Flowers unisexual, male and female flowers similar, on same individuals (plant monoecious). Flower buds ovoid to conical. Male and female inflorescences borne on trunk, sparsely spaced mostly towards the lower half of the trunk, composed of 1 to 2 sessile flowers. Peduncle inconspicuous. Flower pedicel 0–2 mm long, 1–2 mm in diameter, densely pubescent. Bracts up to 3 at base, upper bract 1–2 mm long, ca. 4 mm wide, broadly ovate, pubescent outside, glabrous inside. Sepals 2, 9–15 mm long, 4–6 mm wide, narrowly ovate, basally fused, enclosing the petals in bud, densely pubescent outside, densely pubescent to glabrous toward base inside, brown. Petals 4, 7–15 mm long, 5–8 mm wide, length:width ratio ca. 3.5, ovate, pubescent outside, glabrous inside, brownish to greenish yellow outside, greenish yellow inside. Male flowers: stamens number unknown, ca. 0.5 mm long, anthers linear, connective prolongation truncate. Female flowers: carpels 60, 4–5 mm long, ca. 0.5 mm wide, densely pubescent, free; stigma coiled. Fruits unknown.

##### Distribution.

Endemic to Lower Guinean Domain of the Guineo-Congolian Region: Cameroon, Gabon.

##### Habitat and ecology.

Lowland mature or old secondary rain forests, near streams or in periodically inundated forest. Altitude: 60–300 m asl.

##### Phenology.

Flowers collected from March to June.

##### Uses.

Young leaves are used to wrap fish while cooking to give it an aromatic taste (Letouzey 9017).

##### Notes.

This species resembles *Up.bakeriana*, *Up.korupensis* and *Up.submontana* in having large obovate leaves (more than 31 cm long), with rounded to subcordate bases. *Up.citrata* is unique within the genus by its leaves emitting a strong lemon scent when crushed (Table 5). It also differs from all the other *Uvariopsis* species (except *Up.zenkeri*) in having subsessile flowers, with pedicel less than 2 mm. It differs from *Up.zenkeri* in having larger leaves (31–50 cm vs. less than 16 cm in *Up.zenkeri*).

##### Conservation status.

This species is known from one locality in Gabon situated in the Monts de Cristal National Park, and two localities in Cameroon both situated in the Campo-Ma’an National Park. It has been previously assessed as Data Deficient based on the Gabonese population (Cosiaux et al. 2019c). Here, its EOO is estimated at 3,392 km^2^ and its AOO at 12 km^2^. Given it is close to a road, the future of the Gabonese population is uncertain (Couvreur and Niangadouma 2016). Following IUCN criterion B it is thus assigned an updated preliminary conservation status of Endangered EN B1ab(iii)+2ab(iii).

##### Additional specimens examined.

Cameroon – South Region • G.P. Tchouto Mbatchou 2869 (KRIBI, WAG, YA), Campo-Ma'an area, Bibabimvoto; 2°12'48'N, 10°00'51'E; alt. 60 m; 13 May. 2000 • R.G. Letouzey 9017 (P), 15 km au SSE de Zingui (soit à 50 km au SE de Kribi), feuille IGN 1/200000 Nyabessan; 2°37'52.24'N, 10°15'58.54'E; 14 Mar. 1968. Gabon – Estuaire • T.L.P. Couvreur 1126 (LBV, WAG), Monts de Cristal National Park, 800 m from Kinguélé ANPN camp, near bridge; 0°27'50.18'N, 10°16'42.82'E; 11 Jun. 2016.

#### ﻿ Uvariopsis congensis

Taxon classificationPlantaeMagnolialesAnnonaceae

Robyns & Ghesq., Ann. Soc. Sci. Bruxelles, Ser. B liii. 317 (1933)

**Figure 55.**
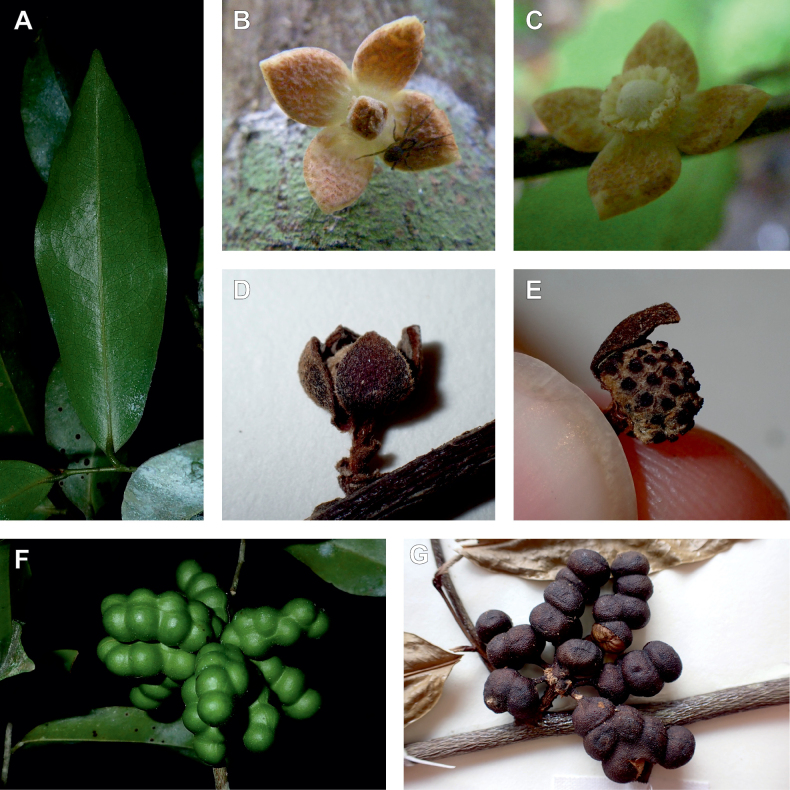
*Uvariopsiscongensis* Robyns & Ghesq **A** leaf, lower side **B** female flower, all carpels fallen, top view **C** male flower, stamens on the top of the receptacle fallen, semi-top view **D** male flower, dry, side view **E** female flower, three petals removed, side view **F** fruit, fresh, top view **G** fruit, dry, side view. **A, F** Texier 2307 **B, C, G** Lachenaud 1384 **D** Wieringa 660 **E** Tisserant 1363. Photos **A, F** Nicolas Texier (CC BY-NC-ND 3.0) **B, C** Olivier Lachenaud (CC BY-NC-ND 3.0) **D, E, G** Léo-Paul Dagallier.

**Figure 56.**
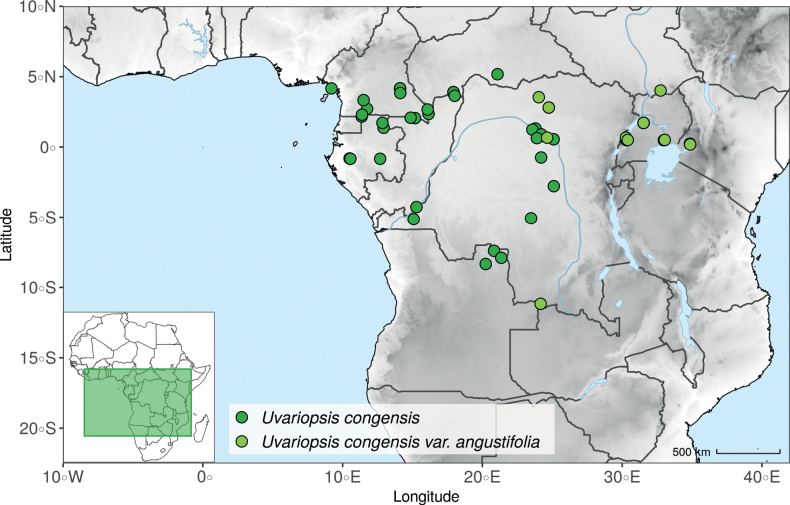
Distribution map of *Uvariopsiscongensis*. Shades of grey represent elevation, from white (sea level) to darker grey (higher elevation). The inset shows the extent of the map over Africa.

Figs 55 , 56 , 85G–M ; Table 6

##### Type.

Democratic Republic of the Congo – Kasai-Oriental • E. van der Kerkhoven s.n (lectotype, here designated: BR! (BR0000008824318)); Lusambo, Saint-Trudon [=Mombelaye]; 5°04'00.23'S, 23°28'59.88'E; alt. 475 m; 16 Aug. 1913.

##### Description.

Shrub to tree 2–15 m tall, D.B.H 3–39 cm; young branches sparsely pubescent to glabrous, old branches glabrous. Petiole 2–5 mm long, 1–2.5 mm wide, glabrous. Leaf lamina 73–177 mm long, 22–77 mm wide, length:width ratio 2.1–4, elliptic to narrowly elliptic to obovate, papyraceous to coriaceous, base acute to decurrent, apex attenuate to acuminate, acumen 5–18 mm long, surface above glabrous, surface below glabrous; midrib impressed above, raised below, glabrous above, glabrous below; secondary veins 7–12 pairs, brochidodromous to weakly brochidodromous, impressed above, raised below; tertiary veins reticulate. Flowers unisexual, male and female flowers similar, on same individuals (plant monoecious). Flower buds globose. Male and female inflorescences borne on old branches or axillary, composed of 1 to 2 flowers. Peduncle inconspicuous. Flower pedicel 3–11 mm long, 0.5–1 mm in diameter, pubescent to glabrous. Bracts 1 to 4 at base and one towards the lower half of pedicel, upper bract ca. 1 mm long, ca. 1 mm wide, triangular, pubescent outside, glabrous inside. Sepals 2, 0.7–1.5 mm long, 1.5–2 mm wide, broadly ovate, free, pubescent outside, glabrous inside, brown. Petals 4, 2.5–8 mm long, 1.5–5 mm wide, length:width ratio 1–1.7, ovate, free, pubescent to glabrate outside, glabrous inside, cream to pale orange outside, cream to yellow inside. Male flowers: stamens 300 to 400, ca. 0.5 mm long, ca. 0.2 mm wide, anthers linear, connective prolongation truncate or absent. Female flowers: carpels 20 to 40, 1.5–2 mm long, 0.5–1 mm wide, pubescent, free; stigma 0.2–0.5 mm long, ca. 0.5 mm wide, globose to flat. Fruiting pedicel 5–15 mm long, 1–3 mm in diameter, pubescent to glabrous. Monocarps, 1–15, 13–45 mm long, 7–19 mm wide, length:width ratio 1.3–3.1, cylindrical, smooth to lumpy, strongly constricted between the seeds, apex rounded to slightly acuminate, glabrate to glabrous, green to orange to red when ripe (black when dry), sessile to stipitate; stipe 0–7 mm long, 1–3 mm wide, glabrous. Seeds 2–11 per monocarp, uniseriate to biseriate, 9.5–14 mm long, 7–10 mm wide, ellipsoid.

##### Distribution.

Element of Lower Guinean Domain and Congolia Domain of the Guineo-Congolian Region and Zambezian Region: Angola, Cameroon, Central African Republic, Democratic Republic of the Congo, Gabon, Kenya, Republic of the Congo, Uganda.

##### Habitat and ecology.

Lowland and premontane mature or secondary rain forests, often in periodically inundated forest. Altitude: 18–1000 m asl.

##### Phenology.

Flowers and fruits collected all year.

##### Vernacular names.

Central African Republic: ‘Ikuta’ (Tisserant 1504) and ‘Molo-Nzange’ (Tisserant 2136) in Lissongo.

##### Notes.

This species resembles *Up.oligocarpa* and *Up.zenkeri* in having elliptic leaves less than 18 cm long at maximum, with a decurrent base. It differs from *Up.oligocarpa* and *Up.zenkeri* in having more carpels (20 to 40 vs. 13 to 20 in *Up.oligocarpa* and 13 to 22 in *Up.zenkeri*) and glabrous monocarps strongly constricted between the seeds (vs. pubescent and not constricted in *Up.oligocarpa* and tomentose and slightly constricted in *Up.zenkeri*) (Table 6).

##### Conservation status.

This species is widespread, distributed from Cameroon to Kenya. It has been previously assessed as Least Concern LC (Harvey-Brown 2019a).

#### ﻿ Uvariopsis congensis var. angustifolia

Taxon classificationPlantaeMagnolialesAnnonaceae

Dagallier & Couvreur var. nov.

urn:lsid:ipni.org:names:77326972-1

##### Type.

South Sudan – Unknown major area • I. Friis 735 (holotype: K! (no barcode)), Talanga; 4°01'N, 32°45'E; alt. 1300 m; 07 Dec. 1980.

##### Diagnosis.

Up.congensisvar.angustifolia differs from the type variety in being trees 7–15 m high (vs. shrubs to trees 2–6 m high) and having narrowly elliptic leaves with a length:width ratio between 3 and 4 (vs. elliptic to obovate leaves with a length:width ratio between 2.1 and 3.1).

##### Description.

Tree 7–15 m tall. Leaf lamina 73–166 mm long, 22–46 mm wide, length:width ratio 3–4, narrowly elliptic.

##### Distribution.

Element of the Congolia Domain of the Guineo-Congolian Region and the Somalia-Masai Region: Democratic Republic of the Congo, Kenya, Uganda.

##### Habitat and ecology.

Lowland and premontane mature or secondary rain forests. Altitude: 470–1000 m asl.

##### Notes.

The variety Uvariopsiscongensisvar.angustifolia was created to acknowledge the clear morphological variation exhibited by several specimens (mainly from East Africa), being taller trees and having narrower leaves than the other specimens (mainly from Central Africa), although this morphotype is not monophyletic (see Fig. 1, Suppl. materials 1, 2).

##### Additional specimens examined.

Democratic Republic of the Congo – Orientale • J.-P.A. Lebrun 2463 (P), Likati Uele; 3°32'25'N, 24°02'11'E; Mar. 1931 • J.-P.A. Lebrun 2619 (P); Buta, Buta-Uele; 2°48'16.32'N, 24°44'59.68'E; Mar. 1931 • J.L.P. Louis 9562 (K); Isangi, Yalulia, à 20 km à l'Est de Yangambi; 0°40'N, 24°38'E; alt. 470 m; 30 May. 1938. Kenya – Nyanza • B. Verdcourt 1695 (K); North Kavirondo, Kakamega Forest Station, By roadside leading to Yala River from Junction of main road with road leading to Forest House section. Kakamega Forest; 0°14'N, 34°51'E; 10 Dec. 1956 • R.B. Faden 70/34 (EA, K); North Kavirondo, Kakamega Forest, Kibiri Block, S. side of Yala River; 0°11'N, 34°52'E; alt. 1550 m; 21 Jan. 1970. Uganda – Buganda • B.T. Styles 236 (K); Mengo, Mabira C.F.R., coupe 71; 0°29'03.48'N, 32°59'05.64'E; alt. 1100 m; 23 Nov. 1962 – Central • R.A. Dümmer 5591 (K), Mulange; 0°30'28.76'N, 33°02'39.79'E; Oct. 1922 – Western Province • B.T. Styles 122 (K); Masindi District, Budongo Forest Reserve, Waibira Block Compt. 19; 1°43'N, 31°32'E; alt. 1097 m; 04 Oct. 1962 • C.M. Harris 167 (K); Masindi District, Budongo Forest Reserve; 1°43'N, 31°32'E; Nov. 1932 • D.L.N. Hafashimana 13 (K); Masindi District, Budongo Forest Reserve, Kaniyo – Pabidi beat; 1°43'N, 31°32'E; alt. 1010 m; Feb. 1996 • H.A. Lindeman 534 (K); Masindi District, Budongo Forest Reserve; 1°43'N, 31°32'E; Mar. 1939 • J.G. Myers 13647 (K); Masindi District, Budongo Forest Reserve; 1°43'N, 31°32'E; 20 Dec. 1940 • M.T. Dawe 484 (K); Toro, Toro at Isungu; 0°28'59.88'N, 30°19'00.12'E; alt. 1500 m; 06 Sep. 1905 • S. Paulo 581 (K); Kabarole, Fort Portal Forest Reserve; 0°40'N, 30°16'E; 18 Jul. 1960 • W.J. Eggeling 2291 (EA, K); Masindi District, Budongo Forest Reserve; 1°43'N, 31°32'E; Nov. 1935 • W.J. Eggeling 3154 (K); Toro, Kibale Forest; 0°30'N, 30°24'E; Aug. 1936 • W.J. Eggeling 3357 (K); Masindi District, Budongo Forest Reserve; 1°43'N, 31°32'E; Jun. 1937. Zambia – North-Western • F. White 3334 (BM, K, MO); Mwinilunga, west of Kalene Hill Mission; 11°10'02.24'S, 24°09'54.84'E; 22 Sep. 1952.

#### ﻿ Uvariopsis congensis var. congensis

Taxon classificationPlantaeMagnolialesAnnonaceae

##### Description.

Shrub to tree 2–6 m tall. Leaf lamina 88–177 mm long, 30–77 mm wide, length:width ratio 2.1–3.1 (3.6), elliptic to obovate.

##### Distribution.

Element of the Lower Guinean Domain and Congolia Domain of the Guineo-Congolian Region and the Zambezian Region: Angola, Cameroon, Central African Republic, Democratic Republic of the Congo, Gabon, Republic of the Congo.

##### Habitat and ecology.

Lowland and premontane mature or old secondary rain forests, often in periodically inundated forest. Altitude: 18–1000 m asl.

##### Additional specimens examined.

Angola – Lunda Norte • I.A. Darbyshire 759 (K), Lovue River near Capaia village; 8°20'17'S, 20°14'25'E; alt. 960 m; 30 Mar. 2013 • J. Gossweiler 13802a (K), Sector fitocologico de Nordeste de Lunda, circunscrição de Chitato. Dundo, Luachimo; 7°24'S, 20°50'E; alt. 700 m; 05 Nov. 1946 • J. Gossweiler 14071 (B, P); 7°54'S, 21°21'E; alt. 700 m; 12 Jun. 1948. Cameroon – Central Region • W.J.J.O. de Wilde 1914 (P, WAG), C. 30 km South of M'Balmayo; 3°20'N, 11°30'E; 13 Feb. 1964 – East Region • D.J. Harris 1512 (K, MO, P), West bank of Sangha River; 2°21'N, 16°09'E; alt. 350 m; 01 Nov. 1988 • F.J. Breteler 2812 (K, P, WAG), Near Bimba, bank of Doumé river, 40 km SW. of Batouri; 4°10'N, 14°07'E; alt. 580 m; 15 Apr. 1962 • J.F. Villiers 683 (P), 2 km N du confluent Ngoko-Malapa, 5 km E de Moloundou; 2°03'17.9'N, 15°11'01.31'E; 21 Apr. 1971 • R.G. Letouzey 10641 (P), Bordure de la Sangha au Sud de Lidjombo (près ile Libongo) à 110 km au N. de Ouesso (feuilles IGN 1/200.000 Moloundou); 2°40'N, 16°05'E; 10 Apr. 1971 • R.G. Letouzey 12086 (K, P, WAG), près Ndongo, à 45 km WNW de Moloundou (Feuille IGN 1/200.000 SOUANKE); 2°05'N, 14°52'E; 15 Mar. 1973 • R.G. Letouzey 4755 (K, P), Rives de la Doumé près Bimba 40 km SW de Batouri – feuille IGN 1/200.000 Batouri-Berberati; 4°10'32.91'N, 14°06'32.96'E; 15 Apr. 1962 • R.G. Letouzey 5491 (K, P), a 11 km au SSW de Koso (village situé à 60 km au SSW de Batouri); 3°50'23.13'N, 14°07'33.62'E; 25 Jul. 1963 – South Region • R.G. Letouzey 10024 (P), Rives du Ntem près du confluent de la Kye, 16 km ESE d'Ambam; 2°18'02.17'N, 11°23'15.84'E; 07 Feb. 1970 • R.G. Letouzey 9915 (P), Bord rivière Nlobo, près Ngomebae, 70 km ESE d'Ebolowa sur route Mvangan; 2°43'N, 11°45'E; 24 Jan. 1970 – South-West Region • A. Staudt 556 (K), Johann – Albrechtshöhe, Johann – Albrechtshohe, Kumba area; 4°10'N, 9°12'E; 1896. Central African Republic – Basse-Kotto • C. Tisserant 1363 (P), Galerie Riv. Moku Sikumdu 10 km N.O. Alindas; 5°10'24.36'N, 21°05'07.74'E; 19 Nov. 1927 – Lobaye • Équipe C. Tisserant 1504 (BM, BR, K, P); Mbaïki, Boukoko; 3°54'N, 17°56'E; 15 Jun. 1949 • Équipe C. Tisserant 2136 (BR, P); Mbaïki, Boukoko; 3°54'N, 17°56'E; 16 Jun. 1951 • Équipe C. Tisserant 2198 (BM, BR, P); Mbaïki, Boukoko; 3°54'N, 17°56'E; 17 Aug. 1951 • Équipe C. Tisserant 2241 (BM, BR, K, P, P); Mbaïki, Boukoko; 3°54'N, 17°56'E; 13 Sep. 1951 • F.J. Badré 239 (B, P); Mbaïki, Mbaiki, Bord de la Lobaye – Forêt 30 km S de Mbaïki; 3°38'25.5'N, 18°00'57.02'E; 06 Nov. 1968. Democratic Republic of the Congo – Bas-Congo • F.H.E.A.W. Robyns 233 (BR); Madimba, Kisantu, Station Rwé droite, Inkisi; 5°08'S, 15°05'E; 10 Jul. 1925 • J. Gillet 22 (BR (BR0000014588372)); Madimba; 1899 – Maniema • C.E.N. Ewango 2664 (MO), Territoire de Kailo, PArc National de la Lomami. Localité Katopa (Camp); 2°46'52'S, 25°06'34'E; alt. 458 m; 21 Mar. 2015 – Orientale • J. Bokdam 3388 (KIS, WAG); Kisangani, 10 km W of Kisangani, near Lindi river; 0°33'N, 25°06'E; 19 Nov. 1971 • J.-P.A. Lebrun 2487 (P); Buta, Buta-Uele; 2°48'16.32'N, 24°44'59.68'E; Mar. 1931 • J.L.P. Louis 13423 (EA, K, MO, P); Isangi, Bassao (île), Un peu en aval de Liléko; 0°54'N, 24°12'E; alt. 470 m; 26 Jan. 1938 • M.D.J. Laurent 1618 (BR); Banalia, Bomaneh; 1°18'N, 23°47'E; 04 Mar. 1906 • R.G.A. Germain 4757 (K), à l'W de Basoko, embouchure de la Lombo; 1°14'14.83'N, 23°34'46.46'E; Feb. 1949 • R.G.A. Germain 4804 (P), Yankeleli, près d'Isangi; 0°44'31'N, 24°12'15'E; Apr. 1949 • R.G.A. Germain 50 (B, K, P); Isangi, village de Yafolo, rives gauche en amont d'Isangi; 0°46'46'N, 24°16'13'E; alt. 470 m; 21 Dec. 1939 • R.G.A. Germain 8207 (K, M); Isangi, île Esali III (en aval d'Isangi); 0°53'34.91'N, 24°13'08.13'E; Feb. 1953 • R.G.A. Germain 8769 (K); Isangi, Yabwesu-Ogeto (Bas Lomami); 0°38'N, 23°54'E; 05 Apr. 1956 – Unknown major area • M.D.J. Laurent s.n (BR); 1°19'N, 23°48'E; 04 Mar. 1906. Gabon – Moyen-Ogooué • T.O.B.E.B. Stévart 4523 (LBV, MO, P), Mabounié, along the Ngounié River; 0°49'59'N, 10°33'23'E; alt. 18 m; 11 May. 2012 – Ngounié • O.L.S. Lachenaud 1384 (BR, BRLU, LBV, MO, P, WAG), Mabounié, rive gauche de la Ngounié juste en aval du débarcadère; 0°48'40'N, 10°29'57'E; alt. 24 m; 15 Nov. 2013 • Ogooué-Lolo • F.J. Breteler 6647 (LBV, P, WAG), 4 km SW of Lastoursville, right side Ogooué R; 0°50'N, 12°41'E; 25 Sep. 1970 – Woleu-Ntem • G.M.P.C. Le Testu 9027 (BM, P), region de Bitam, Meyo kyè; 2°10'N, 11°22'E; Mar. 1933 • J.J. Wieringa 660 (WAG), c. 85 km N of Makokou, Minkébé district, Nsye valley; 1°22'N, 12°56'E; alt. 505 m; 27 Feb. 1990 • MINKébé Series W626 (K, LBV, MAKOK, MO, P, WAG), Minkébé area, river Nouna; 1°43'N, 12°51'E; 16 Dec. 1990. Republic of the Congo – Pool • A.J.B. Chevalier 4176 (P), Brazzaville; 4°16'S, 15°17'E; Jul. 1902.

#### ﻿ Uvariopsis congolana

Taxon classificationPlantaeMagnolialesAnnonaceae

(De Wild.) Fries, Ark. Bot. ser. 2, 3: 42 (1953)

Figs 57 , 58

≡ Thonnera congolana De Wild., Ann. Mus. Congo Belge, Bot. sér. 5, 3(1): 86 (1909). Type. Democratic Republic of the Congo – Equateur • F. Thonner 100 (holotype: BR! (BR0000008824394, BR0000008824202), sheet designated in Couvreur et al (2022); isotypes BR! (BR0000008824219)), Libako près Ngali (Mongala); 2°39'N, 21°30'E; alt. 450 m; 22 Sep. 1896.

##### Description.

Tree 1.5–10 m tall, D.B.H ca. 5 cm; young branches pubescent to glabrous, blackish when dry, old branches glabrous. Petiole 2–5 mm long, 1.5–4 mm wide, pubescent to glabrous. Leaf lamina 163–300 mm long, 44–110 mm wide, length:width ratio 2.4–4, (elliptic) oblong to obovate, papyraceous to coriaceous, base acute to rounded, apex attenuate to acuminate, acumen 2.5–22 mm long, surface above glabrous, surface below sparsely pubescent at base to glabrous when young, glabrous when old; midrib impressed above, raised below, glabrous above, sparsely pubescent to glabrous below; secondary veins 8–17 pairs, brochidodromous to weakly brochidodromous, impressed above, raised below; tertiary veins reticulate. Flowers unisexual, male and female flowers dimorphic, on same individuals (plant monoecious). Flower buds conical to pyramidal. Male inflorescences borne on trunk mainly at ground level, sometimes up to 1 m, composed of 1 to 3 flowers, clumped with other male or female inflorescences. Peduncle inconspicuous. Flower pedicel 95–200 mm long, 1–2 mm in diameter, sparsely pubescent. Bracts 1 to 3 from base towards the lower half of the pedicel, upper bract 1–3 mm long, 1–4 mm wide, depressed ovate, adpressed, semi clasping the pedicel, pubescent outside, glabrous inside. Sepals 2, 2–6.5 mm long, 2–10 mm wide, depressed ovate, free to basally fused, pubescent outside, glabrous inside, color unknown. Petals 3, 11–20 mm long, 9–22 mm wide, length:width ratio 0.8–2, broadly ovate to ovate, fused at base over ca. 30% of the petal length, pubescent to glabrate outside, glabrous inside, pinkish outside. Stamens numerous (exact number unknown), ca. 0.5 mm long, ca. 0.5 mm wide, anthers linear, connective prolongation truncate or absent. Female inflorescences borne on trunk mainly at ground level, sometimes up to 1 m, composed of 1 to 3 flowers, clumped with other male or female inflorescences. Flower pedicel 200–430 mm long, 1–2 mm in diameter, sparsely pubescent. Bracts 1 to 3 from base towards the lower half of the pedicel, upper bract 1–3 mm long, 1–4 mm wide, depressed ovate, adpressed, semi clasping the pedicel, pubescent outside, glabrous inside. Sepals 2, 2–6.5 mm long, 2–10 mm wide, depressed ovate, free to basally fused, pubescent outside, glabrous inside, color unknown. Petals 3, 11–20 mm long, 9–22 mm wide, length:width ratio 0.8–2, broadly ovate to ovate, fused at base over ca. 30% of the petal length, pubescent to glabrate outside, glabrous inside, pinkish outside. Carpels 20 to 40, 2–3.5 mm long, 0.6–1.5 mm wide, densely pubescent, free; stigma ca. 0.5 mm long, ca. 1 mm wide. Fruiting pedicel 200–400 mm long, 1–3 mm in diameter, sparsely pubescent to glabrous. Monocarps, 1–5, 50–80 mm long, 12–32 mm wide, length:width ratio 2.5–3.7, cylindrical, with ca. 4 longitudinal ridge, sparsely pubescent to glabrous, reddish, stipitate; stipe 3–4 mm long, 3–4 mm wide, sparsely pubescent to glabrous. Seeds (2) 6–16 per monocarp, uniseriate to biseriate, ca. 13 mm long, ca. 11 mm wide, ellipsoid, with a longitudinal ridge.

**Figure 57.**
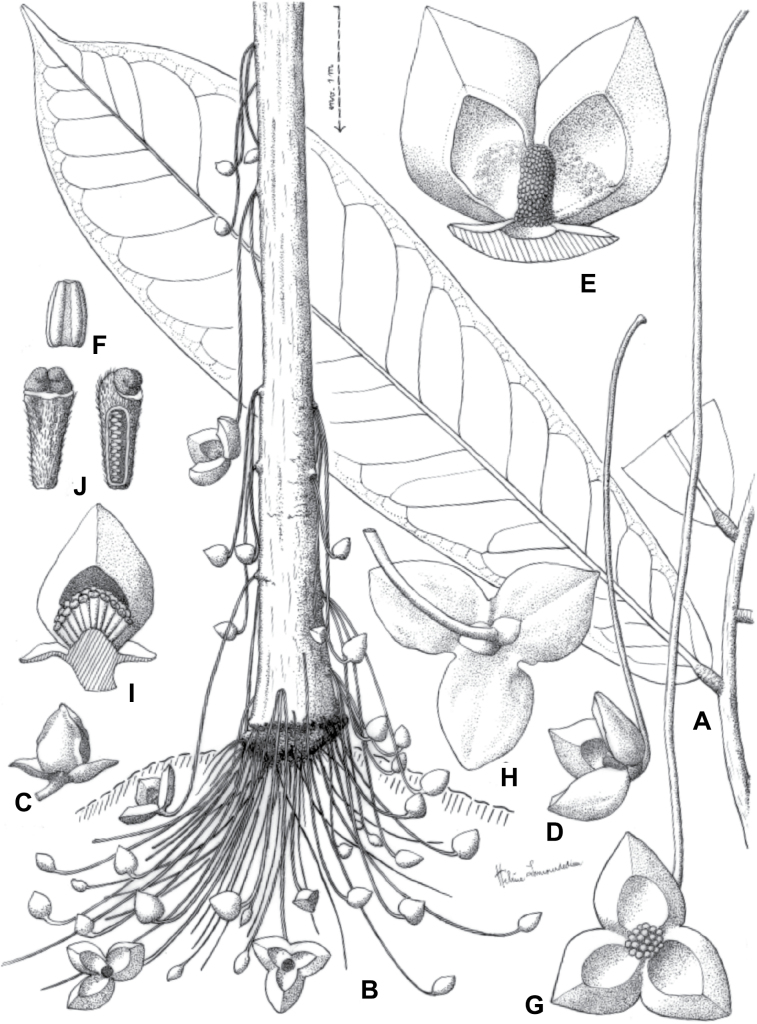
*Uvariopsiscongolana* (De Wild.) Fries **A** leaves, lower side **B** trunk with long flowering pedicels **C** flower bud, side view **D** male flower, side view, note the long pedicel **E** detail of male flower, two petals removed, side view **F** stamen **G** female flower, top view, note the long pedicel **H** female flower, bottom view, note the two sepals **I** longitudinal section of female flower **J** carpel, front view and detail of ovules **A, G** from Hallé 3039 **B–F, H–J** from Hallé 2817. Drawings by Hélène Lamourdedieu, from Le Thomas (1969; pl. 55, p. 305), Publications Scientifiques du Muséum national d’Histoire naturelle, Paris.

##### Distribution.

Element of the Lower Guinean Domain and Congolia Domain of the Guineo-Congolian Region: Democratic Republic of the Congo, Gabon, Republic of the Congo.

**Figure 58.**
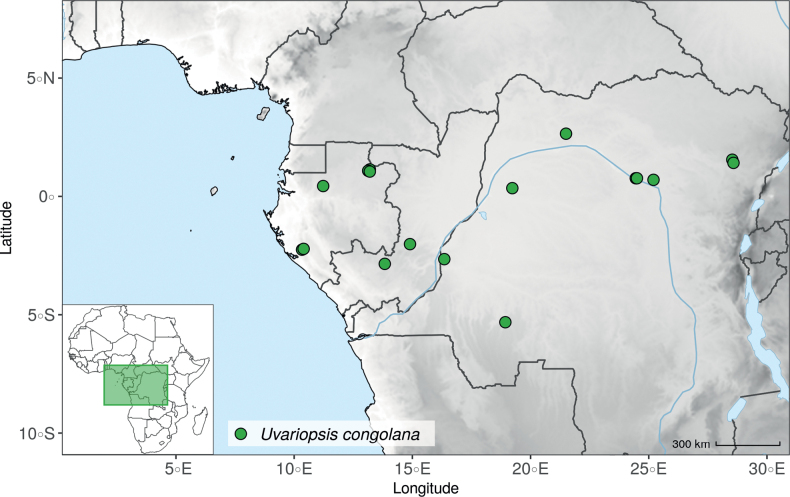
Distribution map of *Uvariopsiscongolana*. Shades of grey represent elevation, from white (sea level) to darker grey (higher elevation). The inset shows the extent of the map over Africa.

##### Habitat and ecology.

Lowland or premontane mature or old secondary rain forest, sometimes in swamp. Altitude: 200–1000 m asl.

##### Phenology.

Flowers collected in March and from August to December. Fruits collected in April, June and December.

##### Vernacular names.

Democratic Republic of the Congo: ‘Asweswe’ in Kimanga (Bokdam 3733), ‘Akobibi’ in Kibila (Ewango 617), ‘Tatakubisa’ (Hart 672).

##### Notes.

Without any flower, this species resemble *Up.dioica*, *Up.guineensis*, *Up.pedunculosa* and *Up.solheidii* in having oblong to obovate leaves, with acute to rounded base and attenuate to acuminate apex. However, when flowering, *Up.congolana* differs from all the other *Uvariopsis* species with flowers with 2 sepals and 3 petals (vs. 2 sepals and 4 petals in other *Uvariopsis* species). Its female flower pedicels and fruiting pedicels are longer comprised between 20 and 40 cm, which is longer than in the other *Uvariopsis* species (except *Up.pedunculosa* that has pedicels that can reach 33 cm). *Up.congolana* differs from *Up.pedunculosa* in having less 20 to 40 carpels (vs. 50–140 in *Up.pedunculosa*), and monocarps marked with longitudinal ridges (vs. monocarps verrucose in *Up.pedunculosa*).

##### Conservation status.

This species has been previously assessed as Least Concern LC (Harvey-Brown 2019b).

##### Additional specimens examined.

Democratic Republic of the Congo – Bandundu • A. Flamigni 10273 (K, M); Mushie, Bolobo; 2°39'03.16'S, 16°21'04.32'E; Mar. 1951 • R. Devred 2881 (K), Kwango, Kiyaka; 5°19'S, 18°56'E; 13 Mar. 1956 – Equateur • C.M. Evrard 5027 (K); Bolomba, Djoa; 0°20'53.88'N, 19°13'44.04'E; alt. 327 m; 15 Oct. 1958 – Orientale • C. Domis 3345 (K); Isangi, Yangambi; 0°46'N, 24°27'E; alt. 470 m; 09 Jan. 1952 • J. Bokdam 3733 (KIS, MO, WAG); Banalia, 20 km along road from Kisangani to Bengamisa; 0°42'N, 25°12'E; 12 Dec. 1972 • J.L.P. Louis 5762 (B, K); Isangi, Yangambi, Isolowe réserve flore; 0°46'N, 24°30'E; 12 Aug. 1937 • T.B. Hart 672 (MO); Mambasa, Epulu; 1°25'N, 28°35'E; alt. 750 m; 14 Aug. 1986 – Unknown major area • C.E.N. Ewango 617 (WAG), Epulu. Zone de Mambasa, Ituri Forest. Afarama; 1°33'N, 28°32'E; alt. 800 m. Gabon – Ogooué-Ivindo • A. Moungazi 234 (P), Bélinga; 1°08'27.49'N, 13°12'03.12'E; alt. 850 m • N. Hallé 2817 (K, P, P, U, WAG), Bélinga; 1°05'N, 13°08'E; alt. 850 m; 27 Oct. 1964 • N. Hallé 3039 (P), Bélinga; 1°05'N, 13°08'E; 06 Nov. 1964 • N. Hallé; A. Le Thomas 64 (K, P), Bélinga, mines de fer, route de Massaka; 1°03'N, 13°12'E; 17 Jul. 1966 – Ogooué-Maritime • J.C. Arends 696 (LBV, MO, P, WAG), Doudou Mountains; 2°15'S, 10°20'E; alt. 670 m; 08 Dec. 1984 • M.S.M. Sosef 1161 (LBV, MO, P, WAG), Monts Doudou, à ± 40 km au Nord-Ouest de Doussala, autour du campement II; 2°13'S, 10°24'E; alt. 425 m; 08 Apr. 2000 – Woleu-Ntem • M.E. Leal 2335 (MO, WAG), Crystal Mountains National Park. Mt. Mekie; 0°26'16'N, 11°13'38'E; alt. 951 m; 20 Nov. 2008. Republic of the Congo – Lékoumou • P. Sita 3209 (P), District de Zanaga, forêt de Mouoni; 2°51'S, 13°50'E; 08 Dec. 1971 – Plateaux • P. Sita 3351 (WAG), région de N'Gouala (Fort-Soufflay); 2°01'S, 14°54'E; 05 Jun. 1972.

#### ﻿ Uvariopsis dicaprio

Taxon classificationPlantaeMagnolialesAnnonaceae

Cheek & Gosline, PeerJ 9(e12614): 8 (2022)

Figs 59 , 60

##### Type.

Cameroon – Littoral • L. MacKinnon 51 (holotype: K! (K001381842); isotypes MO, YA); Yabassi, Ebo Forest, Dicam Trail 2000 m from Bekop camp; 4°20'44'N, 10°24'33'E; alt. 849 m; 25 Mar. 2008.

##### Description.

Tree 3–4 m tall, D.B.H 1.8–2.5 cm; young branches glabrous, old branches glabrous. Petiole 4–5 mm long, 1.9–2.1 mm wide, glabrous. Leaf lamina 177–230 mm long, 64–79 mm wide, length:width ratio 2.7–3, obovate, base acute, minutely cordate, apex acuminate, acumen 0.5–1.3 mm long, surface above glabrous, surface below glabrous; midrib impressed above, raised below, glabrous above, glabrous below; secondary veins 5–9 pairs, brochidodromous, impressed above, raised below; tertiary veins reticulate. Flowers unisexual. Flower buds narrowly ovoid to pyramidal. Male inflorescences borne on trunk from base towards 2.5–3 m, composed of 3 to 7 flowers. Peduncle ca. 2 mm long, ca. 2 mm in diameter, glabrous, bearing radiating 1-flowered partial peduncles 0.5–2 mm long, 0.9–1.2 mm wide. Flower pedicel 18–25 mm long, ca. 1 mm in diameter, glabrous. Bracts 0 to 2, upper bract ca. 1 mm long, ca. 1 mm wide, ovate, sparsely pubescent outside, glabrous inside. Sepals 2, 1–1.5 mm long, 2.1–2.5 mm wide, depressed ovate, glabrous outside, glabrous inside, green to pale brown. Petals 4, 14–16 mm long, 5.5–9 mm wide, length:width ratio 1.7–2.6, ovate to oblong, free, sparsely pubescent outside, glabrous inside, light yellow-green (black when dry) outside, light yellow-green with dark red base inside. Stamens ca. 0.5 mm long, ca. 0.1 mm wide, anthers linear, connective prolongation truncate. Female inflorescences unknown. Fruits unknown.

**Figure 59.**
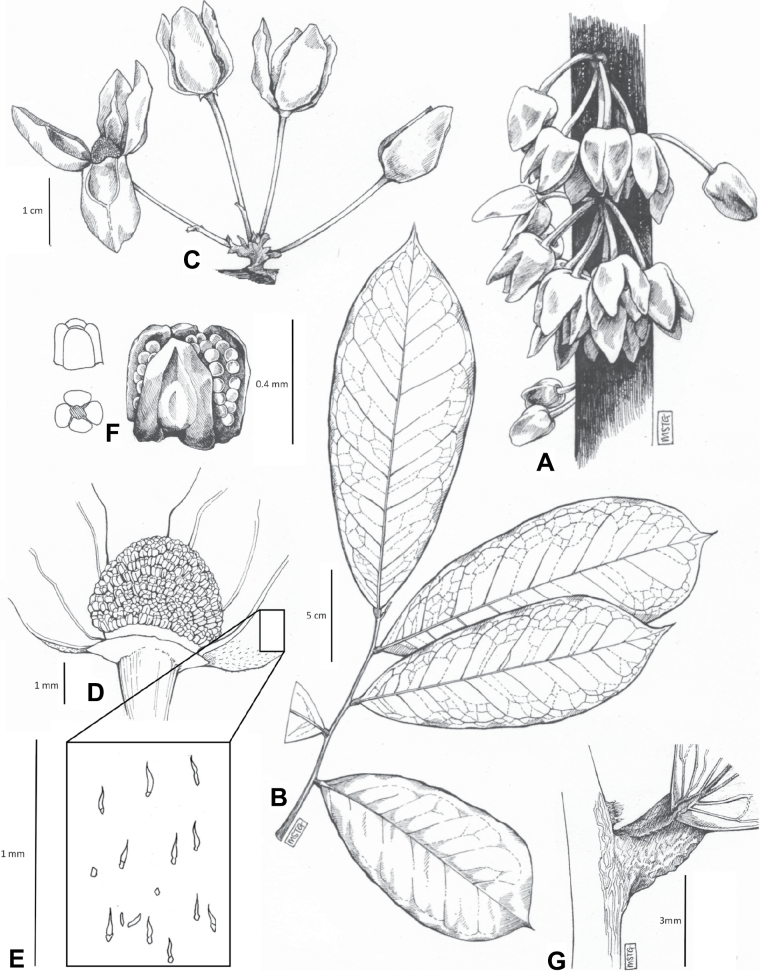
*Uvariopsisdicaprio* Cheek & Gosline **A** inforescences on the trunk **B** young branch with leaves **C** inflorescence, side view **D** detail of male flower, two petals removed **E** detail of petal outer surface **G** detail of petiole and base of leaf. **A–G** from MacKinnon 51 (type). Drawings by Meg Griffiths, from Gosline et al. (2022; fig. 3, p. 5), PeerJ 2022 (CC BY 4.0).

##### Distribution.

Endemic to Lower Guinean Domain of the Guineo-Congolian Region. Known from only one location in Cameroon: the Ebo Forest Reserve.

##### Habitat and ecology.

Submontane mature rain forests. Altitude ca. 280–1000 m asl.

##### Phenology.

Flowers collected in March.

##### Notes.

*Up.dicaprio* resembles *Up.solheidii* in having obovate leaves with acute to minutely cordate leaf base and acuminate apex. It differs from *Up.solheidii* in having young branches and petioles glabrous (vs. tomentose), greater petals (14–16 mm long and 5.5–9 mm wide, vs. 5–10 mm long and 2.5–3.5 mm wide) and petals light yellow-green outside (vs. brown to light red outside). *Up.dicaprio* also differs from all the other *Uvariopsis* species in having and inflorescence peduncle ca. 2 mm long and 2 mm wide (vs. inconspicuous).

**Figure 60.**
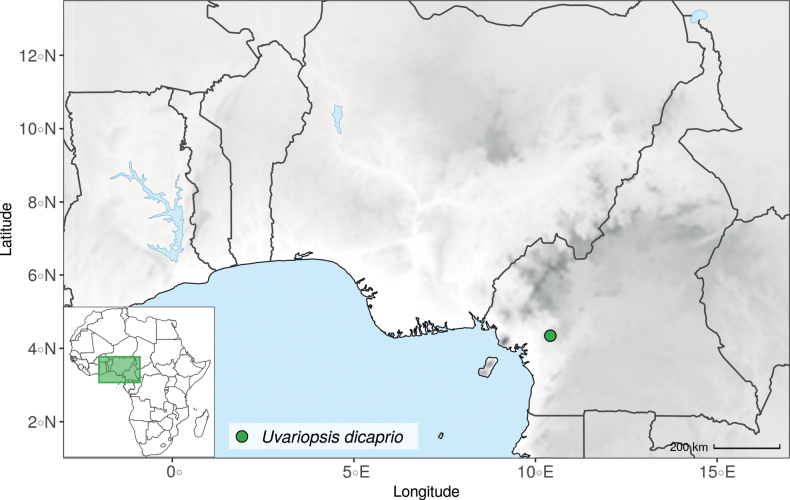
Distribution map of *Uvariopsisdicaprio*. Shades of grey represent elevation, from white (sea level) to darker grey (higher elevation). The inset shows the extent of the map over Africa.

##### Preliminary conservation status.

This species is known from a single specimen collected in the Ebo National Park, and has previously been preliminarily assessed as Critically Endangered, CR B1+2ab(iii), D (Gosline et al. 2022).

#### ﻿ Uvariopsis dioica

Taxon classificationPlantaeMagnolialesAnnonaceae

(Diels) Robyns & Ghesq., Ann. Soc. Sci. Bruxelles, Ser. B liii. 321 (1933)

**Figure 61.**
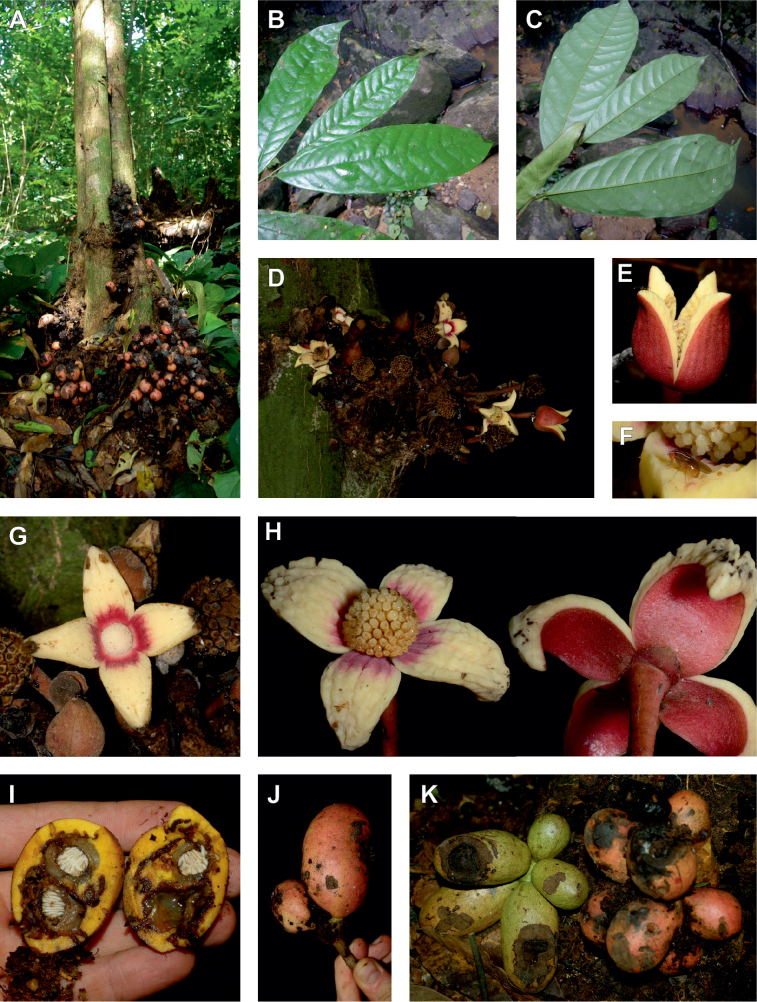
*Uvariopsisdioica* (Diels) Robyns & Ghesq. Fries **A** trunk with infrutescences **B** leaves, upper side **C** leaves, lower side **D** clump of inflorescences on the trunk **E** detail of semi-open female flower, side view **F** fly on petal of femal flower **G** male flower, top view **H** detail of female flower, top view (left) and bottom view (right) **I** transversal cut of fruit **J** detail of fruit, side view **K** fruits mature (right) and unripe (left), top view. **A, I–K** Couvreur 654 **B, C** Lachenaud 2064 **D, G** Stevart 4792A **E, F, H** Bidault 1558. Photos **A, I–K** Thomas Couvreur **B, C** Olivier Lachenaud (CC BY-NC-ND 3.0) **D, G** Tariq Stévart (CC BY-NC-ND 3.0) **E, F, H** Ehoarn Bidault (CC BY-NC-ND 3.0).

**Figure 62.**
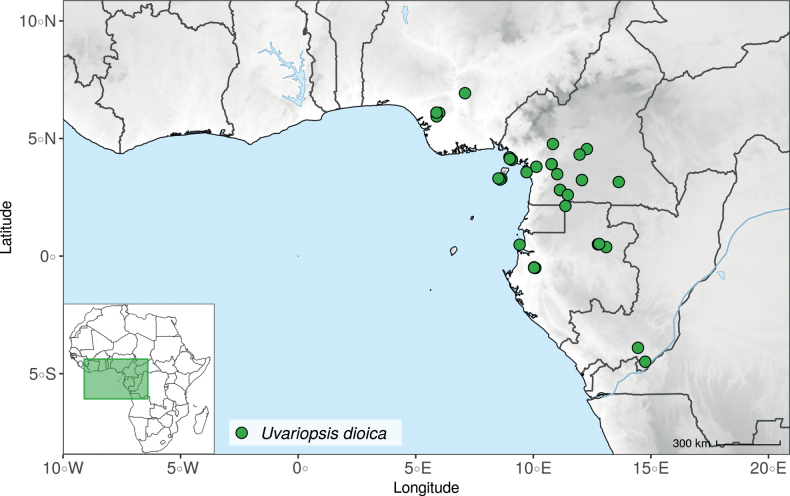
Distribution map of *Uvariopsisdioica*. Shades of grey represent elevation, from white (sea level) to darker grey (higher elevation). The inset shows the extent of the map over Africa.

Figs 49F–M , 61 , 62

≡ Tetrastemma dioicum Diels, Bot. Jahrb. Syst. 38(3): 241 (1906). Type. Cameroon – Littoral • H.J.P. Winkler 909 (lectotype: B! (B 10 0153121), lectotype designated in Couvreur et al (2022), the specimen H.J.P. Winkler 908, cited in the protologue, was not found), Edea; 3°47'50“N, 10°07'50"E; Nov. 1904.
= Tetrastemma sessiliflorum Mildbr. & Diels syn. nov., Bot. Jahrb. Syst. 53(3–5): 440 (1915); ≡ Uvariopsissessiliflora (Mildbr. & Diels) Robyns & Ghesq., Ann. Soc. Sci. Bruxelles, Ser. B liii. 322 (1933). Type. Cameroon – East Region • G.W.J. Mildbraed 5239 (holotype: B! (B 10 0153123); isotypes: BR! (BR0000008824233), HBG! (HBG502485)); Haut-Nyong, Lomié; 3°09'N, 13°38'E; 1911.

##### Description.

Tree to shrub 3.6–20 m tall, D.B.H 8–40 cm; young branches pubescent to glabrous, old branches glabrous. Petiole 1.9–5 mm long, 1–2.5 mm wide, pubescent to glabrous. Leaf lamina 111–245 mm long, 38–92 mm wide, length:width ratio 2.5–3.7, elliptic to obovate, base acute to rounded, apex acuminate, acumen 2–30 mm long, surface above glabrous, surface below glabrous; midrib impressed above, raised below, glabrous above, glabrous below; secondary veins 8–14 pairs, brochidodromous, impressed above, raised below; tertiary veins reticulate. Flowers unisexual, male and female flowers dimorphic, on same individuals (plant monoecious). Flower buds ovoid to conical, rarely globose. Male inflorescences borne in clumps, on thickenings of the trunk, mainly between the base and the lower 3 m of the trunk (but up to ca. 5 m on large specimens), composed of 5 to 20 flowers. Peduncle inconspicuous. Flower pedicel (4) 9–55 mm long, 1–1.5 mm in diameter, pubescent to glabrate. Bracts 1 at base and 1 towards the lower half of the pedicel, upper bract ca. 1 mm long, ca. 1 mm wide, broadly ovate, adpressed, semi clasping the pedicel, pubescent outside, glabrous inside. Sepals 2, 1.5–3 mm long, 2.5–5 mm wide, circular to broadly ovate, sparsely pubescent outside, glabrous inside, purplish brown. Petals 4, 6–17 mm long, 3.5–9.5 mm wide, length:width ratio 1.6–2.5, ovate to elliptic, free, valvate, pubescent to glabrous outside, glabrous inside, red to brownish red outside, yellowish cream to reddish at base inside. Stamens ca. 300, 0.2–0.5 mm long, 0.1–0.5 mm wide, anthers linear, connective prolongation truncate. Female inflorescences fewer in number than the male inflorescences, borne in clumps, on thickenings of the trunk, between the base and the lower 25–50 (150 ?) cm of the trunk, composed of 1 to 10 flowers. Flower pedicel (0) 20–77 (128) mm long, 1–2.5 mm in diameter, pubescent to glabrate. Bracts 1 to 3 at base and 1 to 2 towards the lower half of the pedicel, upper bract 1–3 mm long, 1–3 mm wide, broadly ovate, adpressed, semi clasping the pedicel, pubescent outside, glabrous inside. Sepals 2, 2–5 mm long, 4–11 mm wide, circular to broadly ovate, fused at base, sparsely pubescent outside, glabrous inside, purplish brown. Petals 4, 10–25 mm long, 6–17 mm wide, length:width ratio 1.2–2.5, ovate to elliptic, free, valvate (rarely fused at base over 1–3 mm), pubescent to glabrous outside, glabrous inside, red to brownish red outside, yellowish cream to reddish at base inside. Carpels 100 to 280, 1.5–3.5 mm long, 0.7–1.5 mm wide, pubescent, free; stigma 0–0.6 mm long, 0.5–1 mm wide, coiled. Fruiting pedicel 33–97 mm long, 1–3.5 mm in diameter, sparsely pubescent to glabrous. Monocarps, 1–5, 17–85 mm long, 10–50 mm wide, length:width ratio 1.2–2.1, ellipsoid to cylindrical, smooth, not ridged, sparsely pubescent to glabrous, pale brownish grey to yellow to red, sessile to shortly stipitate; stipe up to 3 mm long, 4–5 mm wide, glabrate. Seeds 5–8 per monocarp, biseriate, 15–22 mm long, ca. 15 mm wide, ellipsoid, in a translucid slimy pulp.

##### Distribution.

Endemic to Lower Guinean Domain of the Guineo-Congolian Region: Cameroon, Equatorial Guinea (Bioko island), Gabon, Nigeria, Republic of the Congo.

##### Habitat and ecology.

Lowland mature or old secondary rain forests. Altitude: 10–980 m asl.

##### Phenology.

Flowers collected from January to June and from October to December. Fruits collected from January to July and from October to November.

##### Notes.

*Up.dioica* resembles *Up.guineensis*, *Up.pedunculosa* and *Up.solheidii* in having elliptic to obovate leaves, with acute to rounded base and attenuate to acuminate apex. It differs from these species in having flowers borne in clumps of 5 to 20 flowers on thickenings of the trunk, lower than 3 m high (vs. inflorescences of 1 or 2 flowers). *Up.dioica* has 100 to 240 carpels, whereas all the other *Uvariopsis* species have less than 100 carpels. Only *Up.korupensis* and *Up.pedunculosa* can have up to 120 and 140 carpels, respectively, but *Up.dioica* has smaller leaves than *Up.korupensis* (11–25 cm long vs. 28–62 cm long) and smaller flower pedicels than *Up.pedunculosa* (20–77 mm long vs. 80–325 mm). Here we make *Ud.sessiliflora* synonym of *Ud.dioica* based on our phylogenetic analyses (Fig. 1, Suppl. materials 1, 2). The specimen Mildbraed 5239, type of *Ud.sessiliflora*, has sessile female flowers. It represents an extreme in the variation of the flower pedicel, which might explain why it remained the sole representative of this name. Note that the authors of *Ud.sessiliflora* (as *Tetrastemmasessiliflorum*) also already mentioned that the carpels of Mildbraed 5239 were similar to those of *Ud.dioica* (Mildbraed and Diels 1915).

##### Preliminary conservation status.

The EOO of this species is estimated at 397,039 km^2^ and its AOO at 128 km^2^. Based solely on AOO, it would qualify for Endangered EN under the B2 criterion, but no other subcriterion (a, b or c) is met. Following IUCN criterion B, it is thus assigned a preliminary conservation status of Least Concern LC.

##### Additional specimens examined.

Cameroon – Central Region • G.W.J. Mildbraed 8260 (K), Übergangs – und Kampfgebiet gegen die Savanne an der Nord-grenze der Hylaea südlich des Sanaga zwischen Jaunde und Dengdeng unweit der vereinigung von Lom (Sanaga) und Djerem. Etwa 115 km N O Jaunde; 4°33'04.96'N, 12°16'43.94'E; Mar. 1914 • H. Jacques-Félix 2493 (P), Ndiki; 4°46'N, 10°50'E; Nov. 1938 • P.R.J. Bamps 1458 (P), Résèrve forestière de Makak; 3°29'N, 11°01'E; 14 Dec. 1967 • R.G. Letouzey 12290 (K, P), Mambe près de Boga, 30 km N. Eseka. (feuille IGN 1/200 000 Edea); 3°53'58.29'N, 10°46'58.4'E; 08 Dec. 1973 • R.G. Letouzey 9541 (MO, P, WAG, YA), Versant septentrional des Monts Mfiki au Sud de Ndo, 25 km NNE d'Esse; 4°19'N, 11°58'E; alt. 983 m; 09 Nov. 1969 – Littoral • R.G. Letouzey 12580 (P), Sude de Ngola (8 km Est de l'embouchure de la Sanaga); 3°33'55'N, 9°42'46'E; 05 Jan. 1974 • T.L.P. Couvreur 654 (WAG, YA), Mambe Massif, above Boga village, 100 km along road from Yaoundé to Edea; 3°54'35.85'N, 10°46'25.97'E; alt. 686 m; 19 Jun. 2014 • T.L.P. Couvreur 659 (WAG, YA), Mambe Massif, above Boga village, 100 km along road from Yaoundé to Edea; 3°54'26.07'N, 10°46'21.27'E; alt. 654 m; 20 Jun. 2014 – South Region • J.J.F.E. de Wilde 7754A (K, P, WAG), Roughly between the village of N'Kolandom and Station du Cacaoyer de N'Koemvone; 2°48'N, 11°09'E; 27 Nov. 1974 • J.J.F.E. de Wilde 8270A (BR, MO, P, PRE, WAG), Station du Cacaoyer de N'koemvone, S. of Ebolowa, 14 km on the road to Ambam. On bank of the Seng river; 2°49'N, 11°08'E; alt. 500 m; 05 Jun. 1975 • J.J.F.E. de Wilde 8709 (BR, MO, P, PRE, WAG, YA), Station du Cacaoyer de N'koemvone, S. of Ebolowa, 14 km on the road to Ambam. On bank of the Seng River; 2°49'N, 11°08'E; alt. 550 m; 12 Dec. 1975 • R.G. Letouzey 4219 (K, P), Nkomo, près Ngoassé au S; de la rivière Lobo – feuille IGN 1/200000 Akonolinga; 3°14'N, 12°04'E; 13 Feb. 1962 • R.G. Letouzey 4230 (P), Nkomo, près Ngoassé au S; de la rivière Lobo – feuille IGN 1/200000 Akonolinga; 3°14'N, 12°04'E; 14 Feb. 1962 • R.G. Letouzey 9934 (K, P), près de la rivière Mboro, près Mevous, 50 km SE d'Ebolowa sur piste d'Evindissi; 2°36'N, 11°28'E; 30 Jan. 1970 – South-West Region • G.W.J. Mildbraed 10647 (K), Bibundi-Pflanzung, Westlich des Kamerunberges; 4°12'N, 8°59'E; Nov. 1928 • J.F. Villiers 2429 (P), Pente SW Mt Cameroun, NE Bakingili, 20 km WNW Limbé; 4°05'22.16'N, 9°05'21.91'E; alt. 800 m; 09 Dec. 1984 • J.J. Wieringa 2029 (BR, E, FHO, K, MO, SCA, WAG); Fako, Limbe, W of Njonji Lake; 4°08'N, 9°01'E; alt. 150 m; 27 Jan. 1994 • M.R. Cheek 5482 (K, SCA, YA), Low altitude forest above oil palm plantation. Reached after c. 40 minutes walk N then E from Njonji. Hunters path to ‘Lake Njonji'; 4°08'N, 9°01'E; alt. 300 m; 18 Nov. 1993 • M.R. Cheek 5501 (K, MO, SCA, YA), Low altitude forest above oil palm plantation. Reached after c. 40 minutes walk N then E from Njonji. Hunters path to ‘Lake Njonji'; 4°08'N, 9°00'E; alt. 5 m; 19 Nov. 1993. Equatorial Guinea – Bioko Sur • W.R.Q. Luke 11970 (EA, MA), Moaba – Moka Trail Pt 139 – Pt 138; 3°17'26.92'N, 8°38'29.83'E; alt. 650 m; 16 Mar. 2007 • W.R.Q. Luke 13050 (MA), Ureka pt 330 to Moraka pt 339; 3°15'19.08'N, 8°35'04.56'E; alt. 141 m • W.R.Q. Luke 13113 (EA, MA, MO), Badja E Trail pt 338; 3°18'04.68'N, 8°31'01.2'E; alt. 700 m; 21 Jan. 2009. Gabon – Moyen-Ogooué • E. Bidault 1558 (BR, BRLU, LBV, MO, P, WAG), Est du lac Azingo, à 30 km au Nord-Ouest de Lambaréné; 0°30'32.42'N, 10°05'08.01'E; alt. 48 m; 08 Jun. 2014 • O.L.S. Lachenaud 2064 (MO), piste du lac Azingo, ± 10 km au NE du lac et 30 km au NW de Lambréné; 0°28'41'N, 10°01'59'E; alt. 20 m; 25 Oct. 2014 • T.L.P. Couvreur 926 (LBV, WAG, YA), 27 km after Lambaréné, on road to Bifoum (N1), then around 20 km on road to Lake Azingo; 0°29'38.73'N, 10°01'55.24'E; alt. 23 m; 24 Nov. 2015 • T.O.B.E.B. Stévart 4792A (P, WAG), Okala, North of Libreville. Terrain de Montigny à Okala (Libreville); 0°29'N, 9°25'E; alt. 10 m; 12 Feb. 2014 – Ogooué-Ivindo • A. Hladik 1472 (BRUNOY, P, P, US), Ipassa, au croisement des layons J et VII; 0°30'N, 12°48'E; alt. 490 m; 29 Oct. 1971 • J.M. Reitsma 3507 (LBV, MO, WAG), Primary rain forest, near village Ekobakoba, 50 km SE of Makokou; inventory; 0°23'N, 13°06'E; 21 May. 1987 • L.J. Dorr 4248 (LBV, MO), Station I.R.E.T. (M'Passa Field Station). 10 km S de Makokou sur la riviere Ivindo; 0°30'N, 12°45'E; alt. 500 m; 13 May. 1985 • L.J. Dorr 4275 (LBV, MO, P), Station I.R.E.T. (M'Passa Field Station), 10 km S de Makokou sur la riviere Ivindo; 0°30'N, 12°45'E; alt. 500 m; 14 May. 1985 • M.S.M. Sosef 2210 (BR, LBV, MO, WAG), Ipassa Reserve, IRET Research Station, SW of Makokou; 0°31'N, 12°48'E; alt. 350 m; 04 Nov. 2005 – Woleu-Ntem • A.M. Louis 4098 (BR, LBV, MO, WAG), 25 km NE of Bitam, Nsimy; 2°08'N, 11°22'E; 02 May. 1995. Nigeria – Edo State • A.F. Ross 2/6 (K); Benin, Sapoba Forest Reserve; 6°06'N, 5°53'E; 1934 • B.O. Daramola FHI72315 (K, MO), Iyekoriowon. Forest Reserve. In P.S.P. 82 Ugo; 6°05'19.05'N, 6°00'04.46'E; 04 Oct. 1973 • R.D. Meikle 628 (K, P), Usonigbe Forest Reserve near Sapoba; 5°56'17.52'N, 5°53'20'E; 16 Nov. 1944 • R.W.J. Keay FHI28066 (K, K, P); Benin, Sapoba Forest Reserve, Compte.9,S.S.I; 6°06'N, 5°53'E; 03 Nov. 1950 – Kogi State • M.G. Latilo FHI47768 (K); Kabba, Southern Adoru Forest Reserve, Igala, bank of river Owe near Adoru; 6°55'38.49'N, 7°05'38.55'E; 01 Jul. 1963. Republic of the Congo – Pool • J. Koechlin 4001 (P), Bangou, Forêt de; 3°54'S, 14°27'E; Dec. 1956 • P. Sita 3264 (P), Forêt de Bangou, Est de M'Passa; 4°29'34'S, 14°45'28'E; 18 Dec. 1971.

#### ﻿ Uvariopsis etugeana

Taxon classificationPlantaeMagnolialesAnnonaceae

Dagallier & Couvreur, PhytoKeys 207: 423 (2022)

Figs 63 , 64

##### Type.

Cameroon – North-West Region • R.G. Letouzey 13414 (holotype: P! (P01982826)), Rive droite de la Metchum, près Obang (18 km S of Wum); 6°15'N, 10°01'59.99"E; alt. 600 m; 03 Dec. 1974.

##### Description.

Tree 3–6 m tall, D.B.H unknown; young branches slightly pubescent, old branches glabrous. Leaves with minute pelucid punctuations. Petiole 3.5–4 mm long, 2.5–3.5 mm wide, glabrous. Leaf lamina 190–235 mm long, 70–85 mm wide, length:width ratio ca. 2.7, elliptic, coriaceous, base acute to slightly decurrent, apex attenuate to acuminate, acumen ca. 10 mm long, surface above glabrous, surface below glabrous; midrib impressed above, raised below, glabrous above, glabrous below; secondary veins 8–10 pairs, brochidodromous, impressed above, raised below; tertiary veins reticulate. Flowers unisexual, male and female flowers dimorphic, on same individuals (plant monoecious). Flower buds ovoid to pyramidal. Male inflorescences borne on trunk, composed of 1 to 2 flowers. Peduncle inconspicuous. Flower pedicel 8–10 mm long, 1–1.5 mm in diameter, glabrate to glabrous. Bracts 1 at base and 1 towards the middle or lower half of the pedicel, upper bract 1–1.5 mm long, 1–2.5 mm wide, broadly ovate, adpressed, semi clasping the pedicel, pubescent outside, glabrous inside. Sepals 2, ca. 2 mm long, ca. 4 mm wide, broadly ovate, basally fused, sparsely pubescent to glabrous outside, glabrous inside, color unknown. Petals 4, 6–11.5 mm long, 3.5–7 mm wide, length:width ratio 1.6–2.5, elliptic to ovate, sparsely pubescent to glabrous outside, glabrous inside, color unknown. Stamens numerous (exact number unknown), 0.5–1 mm long, ca. 0.2 mm wide, anthers linear, connective prolongation truncate. Female inflorescences borne on trunk, composed of 1 to 2 flowers. Flower pedicel ca. 4 mm long, ca. 2 mm in diameter, glabrate to glabrous. Bracts 1 at base and 1 towards the middle or lower half of the pedicel, upper bract ca. 1.5 mm long, ca. 2.5 mm wide, broadly ovate, adpressed, semi clasping the pedicel, pubescent outside, glabrous inside. Sepals 2, 1–1.5 mm long, 2–3.5 mm wide, broadly ovate, basally fused, sparsely pubescent to glabrous outside, glabrous inside, color unknown. Petals 4, ca. 14 mm long, ca. 8 mm wide, length:width ratio 1.6–2.5, elliptic, glabrous outside, glabrous inside, color unknown. Stamens ca. 5, ca. 1 mm long, sterile, seen on one female flower. Carpels ca. 20, ca. 3 mm long, ca. 1 mm wide, glabrate at base to glabrous, free; stigma coiled. Fruits unknown.

**Figure 63.**
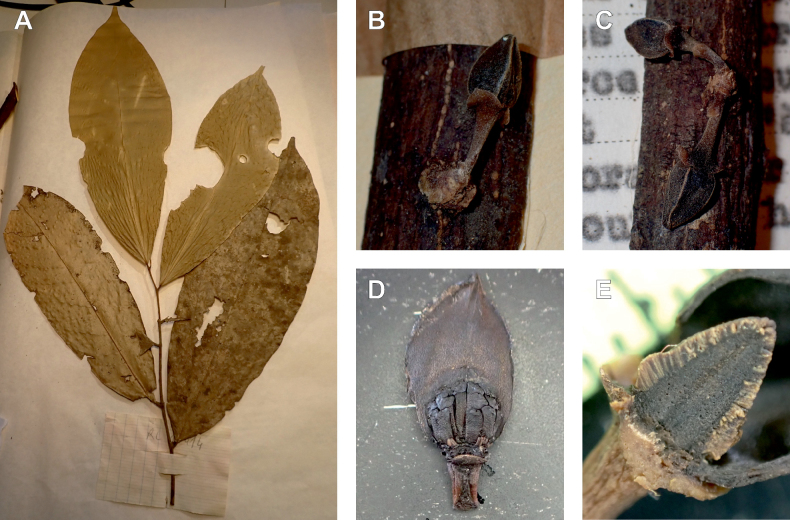
*Uvariopsisetugeana* Dagallier & Couvreur **A** branch with leaves **B** flower, semi-bottom view **C** inflorescence with two flowers **D** female flower, transversal cut, three petals removed **E** male flower, transversal cut of the receptacle. **A–C** Letouzey 13414 **D, E** Thomas 4544. Photos Léo-Paul Dagallier.

##### Distribution.

Endemic to Lower Guinean Domain of the Guineo-Congolian Region. Known from only two localities in the North-West and South-West Regions in Cameron.

**Figure 64.**
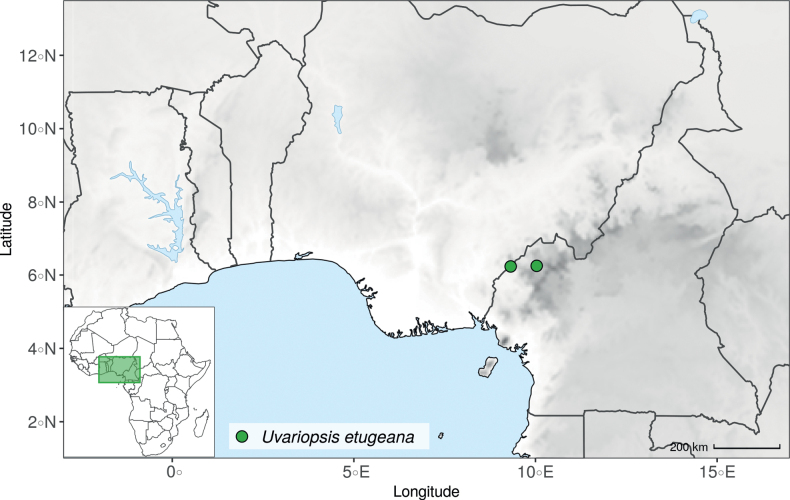
Distribution map of *Uvariopsisetugeana*. Shades of grey represent elevation, from white (sea level) to darker grey (higher elevation). The inset shows the extent of the map over Africa.

##### Habitat and ecology.

Lowland mature rain forests or semi-deciduous forests. 170–600 m asl.

##### Phenology.

Flowers collected in March and December.

##### Notes.

*Up.etugeana* resembles *Up.pedunculosa* in the shape of the leaves, and *Up.solheidii* in the shape of its flowers. However, *Up.etugeana* has a short flowering pedicel (4–10 mm vs. 14–325 mm in *Up.pedunculosa* and 9–198 mm in *Up.solheidii*) and petals which are glabrous outside (vs. pubescent in both *Up.pedunculosa* and *Up.solheidii*).

##### Preliminary conservation status.

This species is known from only two specimens from two different locations in western Cameroon. Its EOO is estimated less than 1,000 km^2^ and its AOO is estimated at 8 km^2^. The two specimens were collected more than 30 years ago, and only one occurs in a protected area, in the Takamanda National Park. This species is thus likely to have undergone, or to undergo in the future, a decline in its AOO. Following IUCN criterion B, it is thus assigned a preliminary conservation status of Endangered EN B1ab(i,ii)+2ab(i,ii).

##### Additional specimens examined.

Cameroon – South-West Region • D.W. Thomas 4544 (MO), Takamanda Forest Reserve; 6°14'N, 9°19'E; alt. 170 m; 21 Mar. 1985.

#### ﻿ Uvariopsis guineensis

Taxon classificationPlantaeMagnolialesAnnonaceae

Keay, Kew Bull. 7(2): 152 (1952)

Figs 65 , 66

≡ Uvaria spectabilis A.Chev., Explor. Bot. Afrique Occ. Franc. i. 7 (1920). Type. Ivory Coast – Danané • A.J.B. Chevalier 21305 (holotype: P! (P00362614), sheet here designated; isotype: P! (P00362612)), Haut Cavally, Pays des Byolas, entre Danané et Goutokouma; 7°16'N, 8°09'W; 25 Apr. 1909.
= Uvariopsis globiflora Keay; syn. nov. concerning Uvariopsisguineensisvar.globiflora (see details under this variety).

##### Description.

Tree 2.5–9 m tall, D.B.H 4–12 cm; young branches sparsely pubescent to glabrous, old branches glabrous. Petiole 1.5–5 mm long, 1.5–3.5 mm wide, sparsely pubescent to glabrous. Leaf lamina 110–310 mm long, 45–108 mm wide, length:width ratio 2.3–4.2, elliptic to oblong to obovate, coriaceous, base acute to rounded, apex attenuate to acuminate, acumen 9–30 mm long, surface above glabrous, surface below glabrous; midrib impressed above, raised below, glabrous above, sparsely pubescent to glabrous below; secondary veins 7–14 pairs, brochidodromous, impressed above, raised below; tertiary veins reticulate. Flowers unisexual, male and female flowers dimorphic, on same individuals (plant monoecious). Flower buds globose to oblate. Male inflorescences borne on trunk, axillary or terminal, composed of 1 flower. Peduncle inconspicuous. Flower pedicel 5–16 mm long, 1–2 mm in diameter, sparsely pubescent. Bracts 1 at base and 1 towards the middle or lower half of the pedicel, upper bract 1–3.5 mm long, 1–3.5 mm wide, broadly ovate, adpressed, semi-clasping the pedicel, pubescent outside, glabrous inside. Sepals 2, 1–3 mm long, 2–7 mm wide, broadly ovate, free to basally fused, pubescent to sparsely pubescent outside, glabrous inside, yellowish green. Petals 4, 7–17 mm long, 5–17 mm wide, length:width ratio 0.9–1.4, broadly ovate, free to fused at base over 30–50% of their length, pubescent to sparsely pubescent outside, glabrous inside, pale greenish to cream outside, greenish cream to cream with purplish base inside. Stamens number unknown, 0.3–1 mm long, 0.2–0.3 mm wide, anthers linear, connective prolongation truncate. Female inflorescences borne on trunk, axillary or terminal, composed of 1 flower. Flower pedicel 10–65 mm long, 1–2 mm in diameter, sparsely pubescent. Bracts 1 to 3 at base and 1 towards the middle or lower half of the pedicel, upper bract 1–3.5 mm long, 1–3.5 mm wide, broadly ovate, adpressed, semi clasping the pedicel, pubescent outside, glabrous inside. Sepals 2, 1–6 mm long, 2–10 mm wide, broadly ovate, free to basally fused, sparsely pubescent to pubescent outside, glabrous inside, yellowish green. Petals 4, 9–24 mm long, 7–22 mm wide, length:width ratio 0.9–1.4, broadly ovate, free to fused at base over 30–50% of their length, pubescent to sparsely pubescent outside, glabrous inside, pale greenish to cream outside, greenish cream to cream with purplish base inside. Carpels 10 to 50, 2–5 mm long, 0.6–1.1 mm wide, pubescent to velutinous, free; stigma ca. 0.5 mm long, 0.5–0.8 mm wide, coiled. Fruiting pedicel 17–69 mm long, 1.5–4.5 mm in diameter, sparsely pubescent to glabrous. Monocarps, 2–10, 20–70 (90) mm long, 12–25 mm wide, length:width ratio 1.7–3.3 (measures taken from both dried fruits and their associated specimen label, note that monocarps seem to sink when drying), cylindrical, smooth, straight to slightly curved, with a longitudinal ridge, sparsely pubescent to glabrous, dull olive green to red to brown, sessile to shortly stipitate; stipe up to 3 mm long, 2–6 mm wide, pubescent to glabrous. Seeds 2–22 per monocarp, biseriate, 12–17 mm long, 10–13 mm wide, in a yellow pulp with strong aniseed scent.

**Figure 65.**
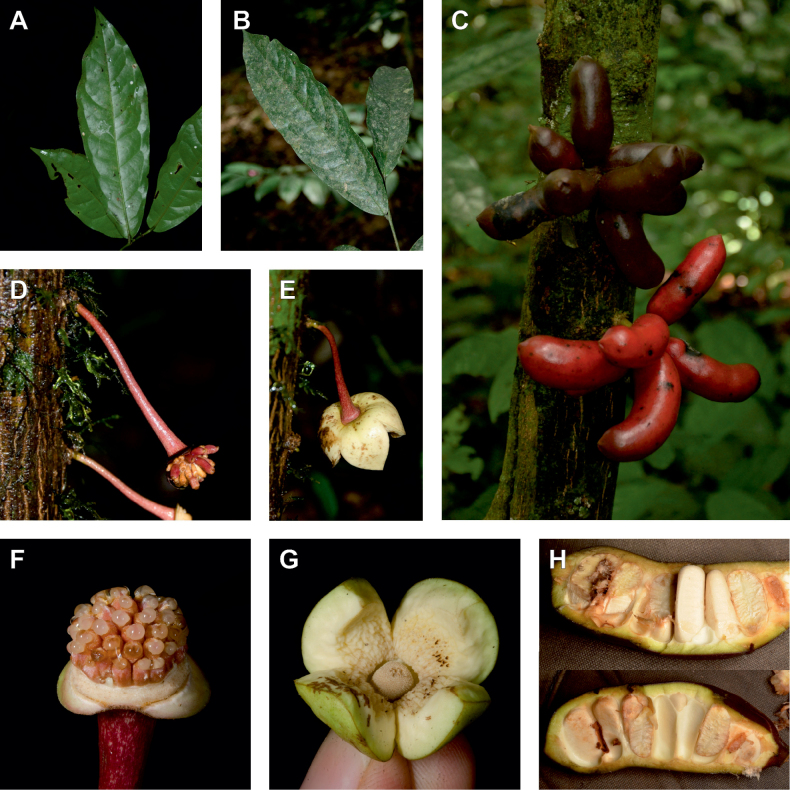
*Uvariopsisguineensis* Keay **A** leaf, lower side **B** leaf, upper side **C** fruits on trunk **D** old female flower, petals fallen, side view **E** flower, semi-bottom view **F** detail of female flower, petals removed, side view **G** male flower, top view **H** monocarp, longitudinal cut. **A, B, D–F** Bidault 4798 **C, H** Bidault 632 **G** Koivogui 250. Photos **A–G** Ehoarn Bidault (CC BY-NC-ND 3.0)

##### Distribution.

Endemic to Upper Guinean Domain of the Guineo-Congolian Region: Ghana, Guinea, Ivory Coast, Liberia, Sierra Leone.

##### Habitat and ecology.

Lowland mature or secondary rain forests. Altitude: 100–950 m asl.

##### Phenology.

Flowers collected from March to May and from September to December. Fruits collected from March to July and in September.

##### Notes.

*Up.guineensis* resembles *Up.dioica*, *Up.pedunculosa*, and *Up.solheidii* in having elliptic to oblong to obovate leaves, with acute to rounded base and attenuate to acuminate apex. Young branches and petioles of *Up.guineensis* are sparsely pubescent to glabrous (vs. pubescent to glabrous in *Up.dioica* and *Up.pedunculosa*, and tomentose to shortly tomentose in *Up.solhdeidii*). *Up.guineensis* has less than 50 carpels (vs. more than 50 in *Up.dioica* and *Up.pedunculosa*), and its monocarps are smooth with a single longitudinal ridge (vs. with longitudinal and transversal ridges in *Up.solheidii* and verrucose in *Up.pedunculosa*). Here we make the names *Ud.guineensis* and *Ud.globiflora* synonyms. They have traditionally been discriminated based on the position of their flowers and the fusion of the petals, with *Ud.globiflora* having axillary or terminal flowers and free petals and *Ud.guineensis* having flowers borne on trunk (cauliflory) and fused petals (Keay 1952). The reliability of the position of the flowers is a character that has been previously discussed in the genus *Uvariodendron* and has been concluded as being an unreliable character (Le Thomas 1967, 1969). Keay himself, who described *Ud.globiflora*, already noticed that “it is […] possible that cauliflory may occur in *Ud.globiflora*” (Keay 1952). Indeed, among the specimens we observed, we found specimens with flowers both on trunk and axillary (e.g. notes on Morton A 4247, identified as *Ud.globiflora*, say “flowers on trunks, branches and amongst leaves”), or with characters of both *Ud.globiflora* and *Ud.guineensis* (e.g. Schmidt 2070, identified as *Ud.globiflora*, has both axillary flowers and fused petals), rendering their placement in one or the other species equivocal. Hawthorne & Jongkind (Hawthorne and Jongkind 2006) also underlined this and already treated the names *Ud.guineensis* and *Ud.globiflora* as synonyms. Our results further support this, as specimens named as *Ud.guineensis* and *Ud.globiflora* form a monphyletic group with strong support and subtended by a relatively long branch in our phylogenetic analyses (Fig. 1, Suppl. materials 1, 2). To account for a morphotype with free petals, we make *Ud.globiflora* a variety of *Ud.guineensis* as Ud.guineensisvar.globiflora.

**Figure 66.**
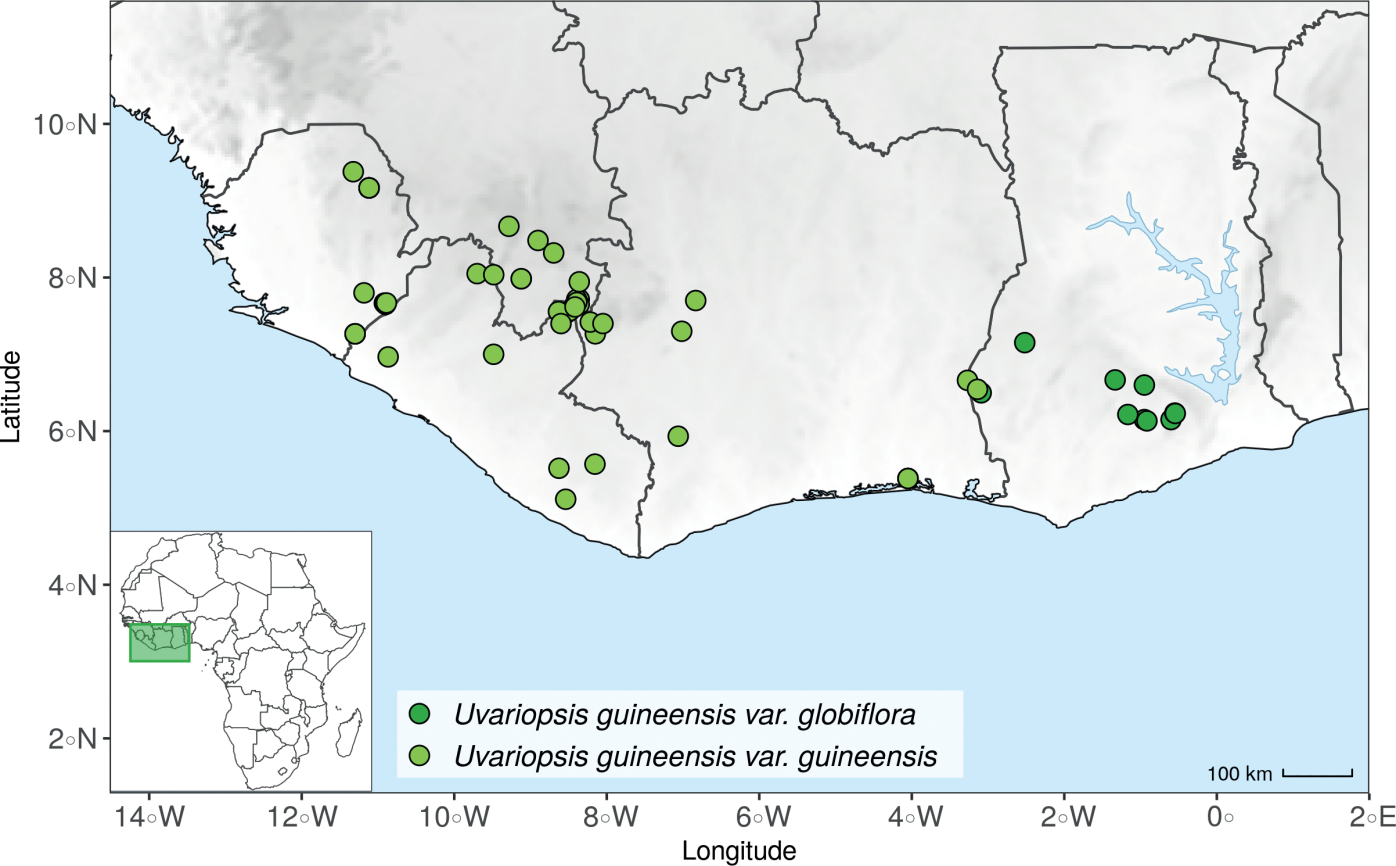
Distribution map of *Uvariopsisguineensis*. Shades of grey represent elevation, from white (sea level) to darker grey (higher elevation). The inset shows the extent of the map over Africa.

##### Conservation status.

This species is widespread in West Africa, from Sierra Leone to Ghana. It has been assessed as Least Concern LC (Harvey-Brown 2019c).

#### ﻿ Uvariopsis guineensis var. globiflora

Taxon classificationPlantaeMagnolialesAnnonaceae

(Keay) Dagallier & Couvreur, comb. et stat. nov.

urn:lsid:ipni.org:names:77326973-1

≡ Uvariopsis globiflora Keay syn. nov., Kew Bull. 7(2): 152 (1952). Type. Ghana – Eastern Region – C. Vigne FH1877 (holotype: K! (K000041224), sheet here designated; isotypes: K! (K000041225, K000041226)), Amentia; 6°13'N, 1°10'W; alt. 160 m; Mar. 1930.

##### Description.

Male inflorescences axillary or terminal (more rarely borne on trunk). Petals 7–10 mm long, 5–9 mm wide, length:width ratio 1.1–1.4, broadly ovate, free. Female inflorescences axillary or terminal (rarely borne on trunk). Sepals 1–3 mm long, 2–4 mm wide. Petals 10–22 mm long, 9–17 mm wide, length:width ratio 1.1–1.4, broadly ovate, free. Female flowers: carpels 10 to 20, 2–4 mm long, 1–1.1 mm wide.

##### Distribution.

Endemic to Upper Guinean Domain of the Guineo-Congolian Region: Ghana.

##### Habitat and ecology.

Lowland mature or secondary rain forests. Altitude: 160–800 m asl.

##### Notes.

Up.guineensisvar.globiflora differs from the type variety in having free petals (vs. fused at base over 30–50% of their length) and fewer carpels (10–20 carpels vs. 20–50 carpels). The male flowers have also smaller petals (7–10 mm long and 5–9 mm wide vs. 8–17 mm long and 9–17 mm wide).

##### Additional specimens examined.

Ghana – Ashanti Region • J.E. Andoh 5096 (P), Bobiri Forest Reserve; 6°40'N, 1°20'W; Jan. 1948 • J.E. Andoh FH4246 (K, P), South Fomangsu Reserve; 6°36'N, 0°57'E; Oct. 1936 – Brong-Ahafo Region • A.A. Enti FE1220 (K, MO), Asukese F.R; 7°09'07'N, 2°31'07'W; 02 Mar. 1973 – Eastern Region • A.A. Enti GC37451 (K), Atewa Range F.R; 6°09'N, 0°36'E; 14 Dec. 1967 • A.A. Enti R698 (BR, MO), Aiyaola Forest Reserve, Kade; 6°09'N, 0°57'E; 03 May. 1972 • C.C.H. Jongkind 1809 (MA, MO, WAG), Atewa Range Forest Reserve, along Old Geological Survey road; 6°14'06'N, 0°33'E; alt. 400 m; 26 Oct. 1994 • D.K. Harder 3325 (MO, WAG), Asiakwa District: Sagyimase Village; Atewa Range (Forest Reserve); between 6–8 km NW of intersection of Accra-Kumasi Road at Sagyimase along forest access road; 6°13'48'N, 0°32'42'E; alt. 810 m; 04 Jul. 1995 • J.K. Morton A4247 (K, WAG), Kade Agric. Res. Station; 6°08'N, 0°55'E; 04 May. 1961 – Western Region • A. Foggie 4456 (K), Bia National Park, Bia F. Resrve; 6°29'55'N, 3°05'24"W; Apr. 1937.

#### ﻿ Uvariopsis guineensis var. guineensis

Taxon classificationPlantaeMagnolialesAnnonaceae

##### Description.

Male inflorescences borne on trunk, axillary or terminal. Petals 8–17 mm long, 9–17 mm wide, length:width ratio 0.9–1.2, broadly ovate, fused over 30–50 % of their length. Female inflorescences borne on trunk, axillary or terminal. Sepals 3–6 mm long, 5–10 mm wide. Petals 9–24 mm long, 7–22 mm wide, length:width ratio 0.9–1.3, broadly ovate, fused at base over 30–50% of their length. Carpels 20–50, 2.1–5 mm long, 0.6–1 mm wide.

##### Distribution.

Endemic to Upper Guinean Domain of the Guineo-Congolian Region: Ghana, Guinea, Ivory Coast, Liberia, Sierra Leone.

##### Habitat and ecology.

Endemic to Upper Guinean Domain of the Guineo-Congolian Region: Lowland mature or secondary rain forests. Altitude: 100–950 m asl.

##### Additional specimens examined.

Ghana – Western Region • H.H. Schmidt 2070 (MO), Bia National Park and Production Reserve. From the town of Bankasa, PArk Guard Camp 13, along foot trail to Park Guard Camp 11; 6°32'40'N, 3°08'20'W; alt. 180 m; 05 Mar. 1996. Guinea – Nzérékoré • A.J.B. Chevalier 20797 (K, P); Macenta, Pays des Koniankéi, Fassakoïdou; 8°40'N, 9°17'W; 24 Feb. 1909 • C.C.H. Jongkind 10660 (WAG), Forêt Classée de Mt Yonon, not far from the Diane River; 7°58'58.8'N, 9°07'16.8'W; alt. 480 m; 07 May. 2011 • C.C.H. Jongkind 10774 (BR, MO, P, WAG), Forêt Classée de Mt Yonon, not far from the Diane River; 7°59'01.8'N, 9°07'17.4'W; alt. 445 m; 12 May. 2011 • C.C.H. Jongkind 11343 (MO, WAG), Nimba mountains. East side of main ridge; 7°38'56.4'N, 8°21'40.8'W; alt. 793 m; 30 Jun. 2012 • C.C.H. Jongkind 7767 (BR, K, P, WAG), Nimba Mountains, banks of Zié River close to reserve boundary; 7°42'58.2'N, 8°21'39.6'W; alt. 540 m; 21 Jun. 2007 • D. Bilivogui 4 (MO, P, WAG); Lola, Guinée Forestière, Nimba Mountains, SMFG iron ore mine concession. Gouan River valley, at its lowest point inside the reserve; 7°42'46'N, 8°23'40'W; alt. 535 m; 28 Sep. 2011 • E. Bidault 4798 (BRLU, MO, P, SERG), Monts Nimba, autour du camp Seringbara 2; 7°37'43.5'N, 8°25'39.72'W; alt. 1020 m; 09 Oct. 2019 • E. Bidault 632 (MO, P); Lola, Nimba Mountain, Guinea, Monts Nimba, site du patrimoine mondial, forêt de Gbié au nord de la grande savane; 7°56'38.37'N, 8°21'38'W; alt. 773 m; 26 Jun. 2012 • J.-G. Adam 3826 (WAG), Nzérékoré. Jomou; 24 Feb. 1949 • Nimba Botanic Team JR1272 (WAG), Nimba Mountains, plot JRFM01, vallée de Gba; 7°40'34.8'N, 8°23'30'W; alt. 947 m; 05 Dec. 2007 • P.K. Haba 107 (K), Béro Mountains, Le sommet du Mont Thon dans les Monts Béro. Proche de la ville de Boola; 8°19'12'N, 8°41'47'W; alt. 1099 m; 03 Dec. 2007 • R.A.A. Schnell 3618 (P), Mt Nimba., Blanmbaya; 7°37'N, 8°25'W; Sep. 1947 • X.M. van der Burgt 1300 (K, WAG); Macenta, Guinée Forestière. Macenta + Beyla préfectures. Simandou Range. South of Pic de Fon, 1.4 km south and 900 m west of Fokou hill; 8°28'55'N, 8°54'05'W; alt. 855 m; 15 Sep. 2008. Ivory Coast – Abengourou • C. Versteegh 619 (U, WAG), Surroundings of Niablé, 30 km east of Abengourou; 6°39'40.63'N, 3°16'11.42'W; 31 Jul. 1969 – Abidjan • J. de Koning 4587 (WAG), Experimental Station ORSTOM, Adiopodoumé. Seedlings, seed source Banco Forest; 5°23'N, 4°03'W; 30 Oct. 1974 • J. de Koning 4905 (WAG), CULTA. Experimental Station ORSTOM, Adiopodoumé. Seedlings, seed source Banco Forest; 5°23'N, 4°03'W; 29 Nov. 1974 • J. de Koning 5170 (MO, WAG), Abidjan, Banco Forest; 5°23'N, 4°03'W; 16 Jan. 1975 • J. de Koning 5504 (BR, MO, WAG), Banco Forest; 5°23'N, 4°03'W; 08 Mar. 1975 – Daloa • L.P.G.A. Nusbaumer 36 (G), F.C. Scio., Pinhou, Lobykro à 5 Km, Bloc 28, Parcelle 141 de la Sodefor; 7°42'N, 6°50'W; 10 Sep. 2001 – Danané • L. Aké Assi 8823 (G), Forêt de Tiapleu 7.4197, -8.2116; 7°25'10.92'N, 8°12'57.6'W; 30 Apr. 1966 • L. Aké Assi 9131 (G), Momi Mont, Foret Classee du, Forêt de Mont Momi; 7°24'N, 8°03'W; 28 Oct. 1966 – Soubré • A.J.B. Chevalier 19224 (P), entre le moyen Sassandra et le moyen Cavally; 5°55'59.75'N, 7°03'46.35'W; 06 Jul. 1907 – Vavoua • F.N. Kouamé 1445 (CSRS, G), F.C. du Haut-Sassandra, Nord. forêt dégradée, relevé FNK14; 7°18'N, 7°01'W; 04 Apr. 1995. Liberia – Bong • D.H. Linder 580 (K), Gbanga; 7°00'N, 9°29'W; 12 Sep. 1926 – Grand Gedeh • C.C.H. Jongkind 6667 (WAG), along Zwedru – Harper road, south of Tiama Town; 5°34'12.6'N, 8°09'16.2'W; alt. 260 m; 07 Jun. 2005 – Lofa • C.C.H. Jongkind 6691 (WAG), North Lorma National Forest; 8°03'N, 9°42'W; 19 Nov. 2005 • C.C.H. Jongkind 9478 (WAG), near Ziggida, rappids in Jèbèh River; 8°02'10.8'N, 9°28'55.8'W; alt. 455 m; 12 Feb. 2010 – Montserrado • J.J.F.E. de Wilde 3811 (MO, WAG), between Bomi hills and Lofa river, c. 15 km N.N.W. of Bomi hills; 6°58'N, 10°52'W; 15 Apr. 1962 – Nimba • J.-G. Adam 20713 (IFAN, K, P, UPS), Nimba; 7°32'N, 8°32'W; alt. 450 m; 23 Jan. 1965 • J.-G. Adam 26362 (MO), Granfield Mt Nimba; 7°34'N, 8°29'W; 17 Oct. 1971 • Nimba Botanic Team NS74 (WAG), Valley between Mt Gangra and Mt Yuelliton, plot SNFR01; 7°33'22.2'N, 8°38'W; alt. 700 m; 14 Jan. 2009 • W.J. Harley 1556 (K), Bilimu; 7°24'N, 8°36'W; 06 Jan. 1948 – Sino • B. Senterre 7066 (MO), Dugbe hummingbird site (ca. 20 km South of Sapo National Park, ca. 50 km East of Greenville), North of Money camp; 5°06'47'N, 8°32'18'W; alt. 107 m; 27 Mar. 2014 • C.C.H. Jongkind 8898 (WAG), Babooni road not too far from main road; 5°31'N, 8°37'36'W; alt. 140 m; 09 Mar. 2009. Sierra Leone – Eastern Province • D. Small 101 (K); Kenema, Dambayei Valley. Kenema, Kambui Hills F.R; 7°48'N, 11°11'W; 25 May. 1951 • J. Momoh 37 (K, SL, WAG), Gola National Park, central block. 600 m N of Malimbe camp; 7°40'07'N, 10°53'22.7'W; alt. 320 m; 24 Oct. 2013 – Northern Province • J.-G. Adam 23345 (MO); Koinadugu, Loma Mountains, Kabala Mt Loma – Kamia; 9°22'37.15'N, 11°19'26.51'W; 26 Jan. 1966 • P. Jaeger 7488 (P); Koinadugu, Loma Mountains, Loma; 9°10'N, 11°07'W; 16 Sep. 1964 • P. Jaeger 7489 (G); Koinadugu, Loma Mountains, W Loma; 9°10'N, 11°07'W; alt. 300 m; 16 Sep. 1964 • P. Jaeger 8041 (G, K, P); Koinadugu, Mt Loma; 9°10'N, 11°07'W; alt. 600 m; 13 Oct. 1964 • P. Jaeger 8756 (K); Koinadugu, Mt Loma; 9°10'N, 11°07'W; alt. 800 m; 29 Dec. 1965 • P. Jaeger 9093 (K, P); Koinadugu, Mt Loma; 9°10'N, 11°07'W; 26 Jan. 1966 • P. Jaeger 9159 (G, K, P); Koinadugu, Mt W Loma; 9°10'N, 11°07'W; alt. 600 m; 29 Jan. 1966 – Southern Province • A.H. Unwin 51 (K); Pujehun, Gola Forest; 7°16'N, 11°18'W; 1909 • D. Small 593 (K, K, P); Pujehun, Gola forest; 7°40'N, 10°55'W; 08 Apr. 1952 • D. Small 655 (K); Pujehun, Gola Forest; 7°16'N, 11°18'W; 1952 • D. Small 676 (K, P); Pujehun, Gola forest, Bagbe Line 20n B.III; 7°16'N, 11°18'W; 19 May. 1952 • D. Small 678 (K); Pujehun, Gola forest, Bagbe Line 20n B.III; 7°16'N, 11°18'W; 15 May. 1952 • H.D. Jordan 2068 (B, K, P); Pujehun, Gola Forest, mile 44 (Tunkia); 7°39'N, 10°54'W; 14 May. 1955.

#### ﻿ Uvariopsis korupensis

Taxon classificationPlantaeMagnolialesAnnonaceae

Gereau & Kenfack, Adansonia sér. 3, 22(1): 41 (2000)

**Figure 67.**
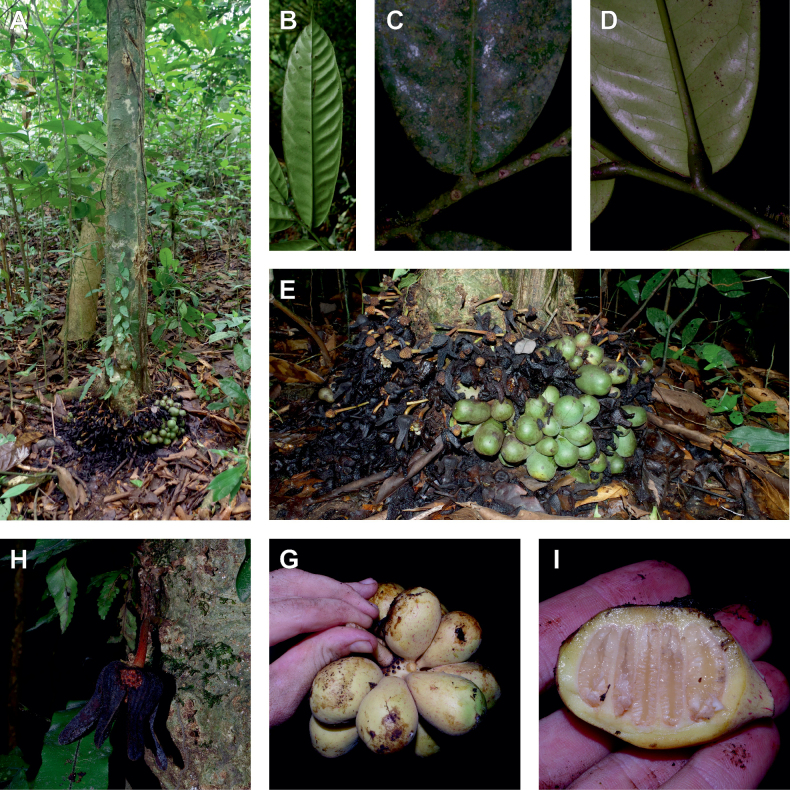
*Uvariopsiskorupensis* Gereau & Kenfack **A** trunk **B** leaf, lower side **C** leaf base, upper side **D** leaf base, lower side **E** clumps of flowers and fruits at the base of the trunk **F** flower, side view **G** fruit, side view **H** monocarp, longitudinal cut. **A–I** Couvreur 1052. Photos Thomas Couvreur.

**Figure 68.**
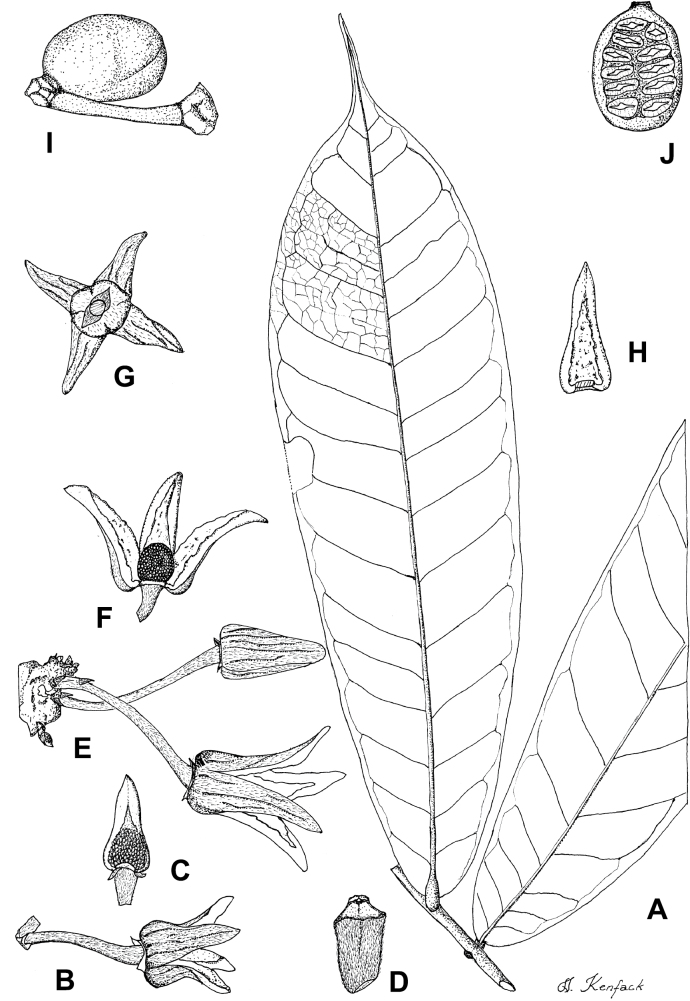
*Uvariopsiskorupensis* Gereau & Kenfack **A** branch with leaves **B** female flower, side view **C** female flower, three petals removed, side view **D** carpel **E** male flower and male flower bud, side view **F** male flower, one petal removed, side view **G** male flower, bottom view **H** petal of male flower, inner view **I** fruit, with **A** single monocarp **J** longitudinal section of monocarp. **A** from Kenfack 1146 **B–K** from fresh material. Scale bars: 1 cm (**A–C, E–K**); 0.5 cm (**D**). Drawings by David Kenfack, from “Le genre *Uvariopsis* (Annonaceae) en Afrique tropicale, avec la description d’une espèce nouvelle du Cameroun” Adansonia 22:1, fig. 1, p. 42 (Gereau and Kenfack 2000), Publications Scientifiques du Muséum national d’Histoire naturelle, Paris.

**Figure 69.**
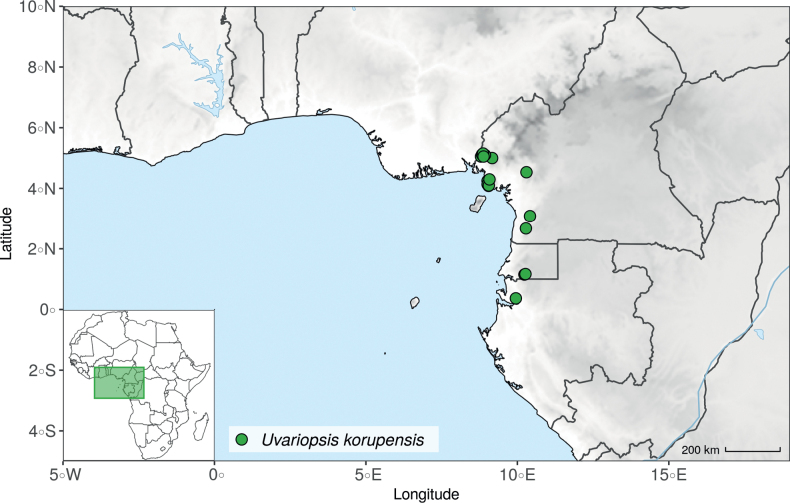
Distribution map of *Uvariopsiskorupensis*. Shades of grey represent elevation, from white (sea level) to darker grey (higher elevation). The inset shows the extent of the map over Africa.

Figs 67 , 68 , 69 ; Table 5

##### Type.

Cameroon – South-West Region • D. Kenfack 1026 (holotype: YA! (YA0003173); isotypes: MO! (MO-022919), P! (P01817719), WAG! (WAG0358388)); Ndian, Korup National Park, Chimpanzee Camp; 5°04'N, 8°52'E; alt. 160 m; 03 Feb. 1998.

##### Description.

Tree 3–15 m tall, D.B.H 5–14 cm; young branches pubescent to glabrous, old branches glabrous. Petiole 2–7 mm long, 3–6 mm wide, pubescent to glabrous. Leaf lamina 280–615 mm long, 83–165 mm wide, length:width ratio 2.9–4.3, obovate, coriaceous, base rounded to cordate, apex acuminate, acumen 18–32 mm long, surface above glabrous, surface below glabrous; midrib impressed above, raised below, glabrous above, glabrous below; secondary veins 13–26 pairs, brochidodromous, impressed above, raised below; tertiary veins reticulate. Flowers unisexual, male and female flowers dimorphic, on same individuals (plant monoecious, but individuals with only staminate flowers were also observed). Flower buds conical. Male inflorescences borne on thickenings of the trunk, mainly at the base of the trunk, towards up to 3.8 m, composed of 2 to 3 flowers. Peduncle inconspicuous. Flower pedicel 6–70 mm long, 1–2 mm in diameter, pubescent to sparsely pubescent. Bracts 1 to 3 at base and one towards the lower 10% of pedicel, upper bract 1–3.5 mm long, 1.5–5 mm wide, broadly ovate, adpressed, semi-clasping the pedicel, pubescent outside, glabrous inside. Sepals 2, 1–5 (7.5) mm long, 2–6.5 mm wide, broadly ovate, basally fused, pubescent outside, glabrous inside, dull yellowish-brown. Petals 4, 10–38 mm long, 4–10 mm wide, length:width ratio 2.5–7, narrowly ovate, fused at base, pubescent outside, glabrous and verrucose inside, cream to brownish outside, cream to pinkish inside. Stamens numerous (exact number unknown), 0.5–0.8 mm long, 0.1–0.3 mm wide, anthers linear, connective prolongation truncate. Female inflorescences borne on thickenings of the trunk, mainly at the base of the trunk, towards up to 3.8 m, composed of 2 to 3 flowers. Flower pedicel 20–50 mm long, 1.5–2.5 mm in diameter, pubescent to sparsely pubescent. Bracts 1 to 3 at base and one towards the lower 10% of pedicel, upper bract 1–3.5 mm long, 1.5–5 mm wide, broadly ovate, adpressed, semi clasping the pedicel, pubescent outside, glabrous inside. Sepals 2, 3–5 mm long, 4–5 mm wide, broadly ovate, basally fused, pubescent outside, glabrous inside, dull tan. Petals 4, 15–35 mm long, 7–12 mm wide, length:width ratio 2.2–5, narrowly ovate, fused at base, pubescent outside, glabrous and verrucose inside, cream to brownish outside, cream to pinkish inside. Carpels 25 to 120, 1.5–2.5 mm long, 0.5–1 mm wide, velutinous, free; stigma ca. 0.6 mm long, 0.8–1.3 mm wide, truncate pyramidal. Fruiting pedicel 45–90 mm long, ca. 3 mm in diameter, glabrous. Monocarps, 5–9, 29–45 mm long, 18–30 mm wide, length:width ratio 1.4–1.9, ellipsoid to cylindrical, smooth, glabrous, clear orangish yellow, sessile to shortly stipitate; stipe up to 5 mm long, ca. 2 mm wide, glabrous. Seeds 8–14 per monocarp, biseriate, 10–22 mm long, 5–14 mm wide, ellipsoid to oblong.

##### Distribution.

Endemic to Lower Guinean Domain of the Guineo-Congolian Region: Cameroon, Equatorial Guinea, Gabon.

##### Habitat and ecology.

Lowland mature or secondary rain forests. Soil: sandy, rocky, volcanic. Altitude 90–160 m asl.

##### Phenology.

Flowers collected from January to March, in April and from July to November. Fruits collected from February to April and in October.

##### Notes.

This species resembles *Up.bakeriana*, *Up.citrata* and *Up.submontana* in having large obovate leaves (from 28 to 62 cm long), with rounded to cordate bases. *Up.korupensis* differs from *Up.bakeriana* and *Up.citrata* in having longer flower pedicel (6–70 mm vs. less than 8 mm), and differs from *Up.bakeriana* in having fused and cream petals (vs. free and pinkish red). It is harder to differentiate *Up.korupensis* and *Up.submontana*. *Up.korupensis* has generally larger leaves than *Up.submontana* (28–62 cm vs. 16–38 cm). The female flowers of *Up.korupensis* have smaller sepals (3–5 mm long vs. 6–8 mm long in *Up.submontana*), and generally longer petals (15–35 mm long, vs. 15–17 mm long), which results in petals more than 3 times longer than the sepals (vs. petals less than 3 times longer than the sepals in *Up.submontana*). Petals of *Up.korupensis* are cream to brownish outside and cream to pinkish inside whereas petals of *Up.submontana* are pinkish to dark red inside and outside (Table 5).

##### Conservation status.

This species has been assessed as Endangered EN under criteria B2ab(iii) (Cheek 2014a).

##### Additional specimens examined.

Cameroon – Littoral • D. Kenfack 1620 (MO), Nkam, Yingui. Ekem River bank; 4°32'N, 10°18'E; alt. 500 m; 05 Mar. 2002 – South Region • G.A. Zenker 3971 (BM, E, G, G, K), Bipinde; 3°05'N, 10°25'E; 1911 • G.P. Tchouto Mbatchou BIFAX25 (WAG), Campo Ma'an area, Bifa, in the National Park; 2°40'28'N, 10°16'54'E; alt. 60 m; 13 Oct. 2001 – South-West Region • A.H. Gentry 62456 (MO), Banyong, Batanga area, between Awong and Banyu, ca 15 km W of Manyemen. TRANSECT 3; 5°00'N, 9°10'E; alt. 420 m; 03 May. 1988 • D.W. Thomas 3182 (L, MO, P); Ndian, Cameroon. South-West, forest in the Korup National Park; 5°03'N, 8°48'E; alt. 50 m; 28 Feb. 1984 • D.W. Thomas 631 (K); Ndian, Korup National Park, transect R; 5°06'N, 8°55'50'E; Jul. 1979 • G.P. Tchouto Mbatchou 675 (K, SCA, YA), Bomana forest. Transect OA, Plot OA0X; 4°15'N, 9°01'E; alt. 200 m; 05 Oct. 1993 • J. Nning 284 (K, MO, SCA, YA); Fako, Bakingili, along a hill`slope to drink gari Camp above the 6^th^ plot; 4°05'N, 9°03'E; alt. 480 m; 16 Feb. 1997 • M.R. Cheek 5486 (K, SCA, YA), Low altitude forest above oil palm plantation. Reached after c. 40 minutes walk N then E from Njonji. Hunters path to ‘Lake Njonji'; 4°08'N, 9°01'E; alt. 300 m; 19 Nov. 1993 • M.R. Cheek 8258 (K, YA); Ndian, Korup National Park, Ekundu Kundu, Transect 9, c. 7.5 cm; 5°07'N, 8°50'E; alt. 170 m; 28 Apr. 1996 • M.R. Cheek 8815 (K, YA); Ndian, Korup National Park, path from village of Ekundu Kundu to top of Mt Juahan; 5°09'N, 8°52'E; alt. 550 m; 09 Jan. 1998 • R.E. Gereau 5192 (MO, P, WAG), Ndian Division; NW of Mundemba and W of Fabe Village, less than 1 km NE of confluence of Six Cup Garri Creek with Ndian (Mana) River; 5°03'N, 8°53'E; alt. 90 m; 05 Mar. 1993 • T.L.P. Couvreur 1039 (MPU, WAG, YA), Mount Cameroon National Park, Bakinguili trail, above Bakinguili village; 4°05'47.97'N, 9°03'24.72'E; alt. 563 m; 02 Apr. 2016 • T.L.P. Couvreur 1052 (MPU, WAG, YA), Mount Cameroon National Park, on the Bomona trail, behind Bomona village, 10 km NW from Idenau; 4°17'48.99'N, 9°04'43.07"E; alt. 688 m; 03 Apr. 2016. Equatorial Guinea – Rio Muni, Litoral • C.M. Wilks 1784 (LBV, WAG), Monts de Cristal. 11 km ENE d'Okuamkos; 1°09'N, 10°14'E; 10 Aug. 1988 – Unknown major area • C.M. Wilks 1818 (LBV, P, P, WAG), Monts de Cristal. 15 km d'Okuamkos; 1°10'N, 10°16'E; 23 Aug. 1988. Gabon – Estuaire • A.M. Louis 1863 (BR, K, LBV, MO, WAG), Andem, Kougouleu-Kango ± 10 km, 2 km N; 0°22'N, 9°57'E; 09 Oct. 1985.

#### ﻿ Uvariopsis lovettiana

Taxon classificationPlantaeMagnolialesAnnonaceae

Couvreur & Q.Luke, Blumea 55(1): 70 (2010)

Figs 70 , 71 , 72

##### Type.

Tanzania – Morogoro • T.L.P. Couvreur 97b (holotype: WAG! (WAG0361229), sheet here designated; isotypes: DSM, MO, WAG! (WAG0361230, WAG0361231)); Kilombero District, E Udzungwa National Park. In forest S of Mwanihana hill. c. 2 km S of last camping site on Mwanihana trail; 7°48'35.36'S, 36°49'26.4'E; alt. 1400 m; 30 Nov. 2006.

##### Description.

Tree 3–30 m tall, D.B.H 3.5–50 cm; young branches sparsely pubescent to glabrous, old branches glabrous. Petiole 4–9 mm long, 1.2–1.5 mm wide, sparsely pubescent to glabrous. Leaf lamina 150–280 mm long, 45–72 mm wide, length:width ratio 2.5–4, elliptic, coriaceous, base acute to decurrent, apex acute to acuminate, acumen 5–25 mm long, surface above glabrous, surface below sparsely pubescent to glabrous when young, glabrous when old; midrib impressed above, raised below, glabrous above, glabrous below; secondary veins 9–16 pairs, weakly brochidodromous, impressed above, raised below; tertiary veins reticulate. Flowers unisexual, male and female flowers similar, on same individuals (plant monoecious). Flower buds globose. Male and female inflorescences axillary, composed of 1 flower. Peduncle inconspicuous. Flower pedicel 14–50 mm long, 0.7–3 mm in diameter, sparsely pubescent. Bracts 1 to 2 at base, upper bract 0.5–3 mm long, 0.5–3 mm wide, broadly ovate, puberulent outside, glabrous inside. Sepals 2, 1–4 mm long, 2–5 mm wide, depressed to broadly ovate, basally fused, pubescent to glabrous outside, glabrous inside, green. Petals 4, 6–15 mm long, 5–8 mm wide, length:width ratio 1.4–1.7, ovate, free, sparsely pubescent to glabrous outside, glabrous inside, light green outside, white inside. Male flowers: stamens ca. 160, 1.1–2 mm long, 0.3–0.5 mm wide, anthers linear, connective prolongation truncate. Female flowers: stamens 1–5, ca. 1 mm long, minute, fertile. Carpels 32 to 100, 2–3.5 mm long, 1–3 mm wide, densely pubescent, free; stigma ca 0.5 mm long, 0.2–1 mm wide. Fruiting pedicel 14–42 mm long, 1–2.5 mm in diameter, glabrous to sparsely pubescent. Monocarps, 2–7, 13.5–37 mm long, 4.5–17 mm wide, length:width ratio 1.1–3, cylindrical, smooth to lumpy, strongly constricted between the seeds, apex rounded to slightly acuminate, sparsely pubescent to glabrous, glossy yellow to red, stipitate; stipe 2.5–14 mm long, 1.5–3 mm wide, sparsely pubescent to glabrous. Seeds 2–6 per monocarp, uniseriate, 13.5–15 mm long, ca. 10 mm wide, ellipsoid, testa smooth, papery, peeling off.

**Figure 70.**
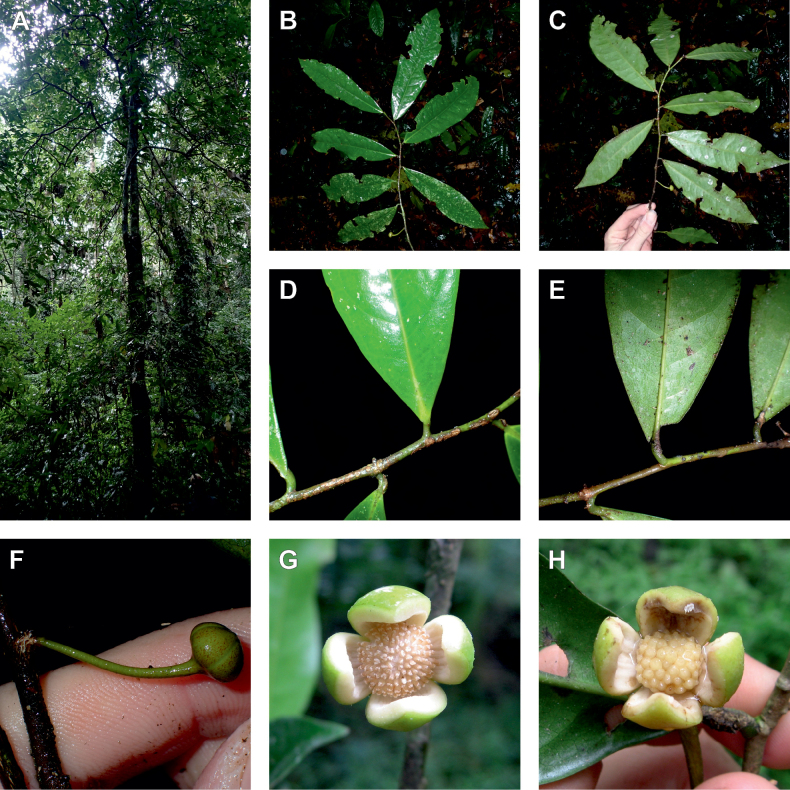
*Uvariopsislovettiana* Couvreur & Q.Luke **A** habit **B** branch with leaves, upper side **C** branch with leaves, lower side **D** leaf base, upper side **E** leaf base, lower side **F** flower bud, side view **G** male flower, top view **H** female flower, top view. **A–F** Dagallier 66 **G, H** Couvreur 97b (type). Photos **A–F** Léo-Paul Dagallier **G, H** Thomas Couvreur.

##### Distribution.

Endemic to Somalia-Masai Region: Tanzania.

##### Habitat and ecology.

Submontane mature rain forests. Altitude: 900–1700 m asl.

##### Phenology.

Flowers collected in January, February, May, July, August and from October to December. Fruits collected in February and from June to November.

##### Notes.

*Up.lovettiana* resemble *Up.congensis* in having elliptic leaves with acute to decurrent base and attenuate to acuminate apex, and cylindrical monocarps strongly constricted between the seeds. However, *Up.lovettiana* has generally larger leaves than *Up.congensis* (15–28 cm long vs. 7–17.7 cm long), longer flower pedicels (14–50 mm long vs. 3–11 mm long), and larger petals (6–15 mm long and 5–8 mm wide vs. 2.5–8 mm long and 1.5–5 mm wide).

**Figure 71.**
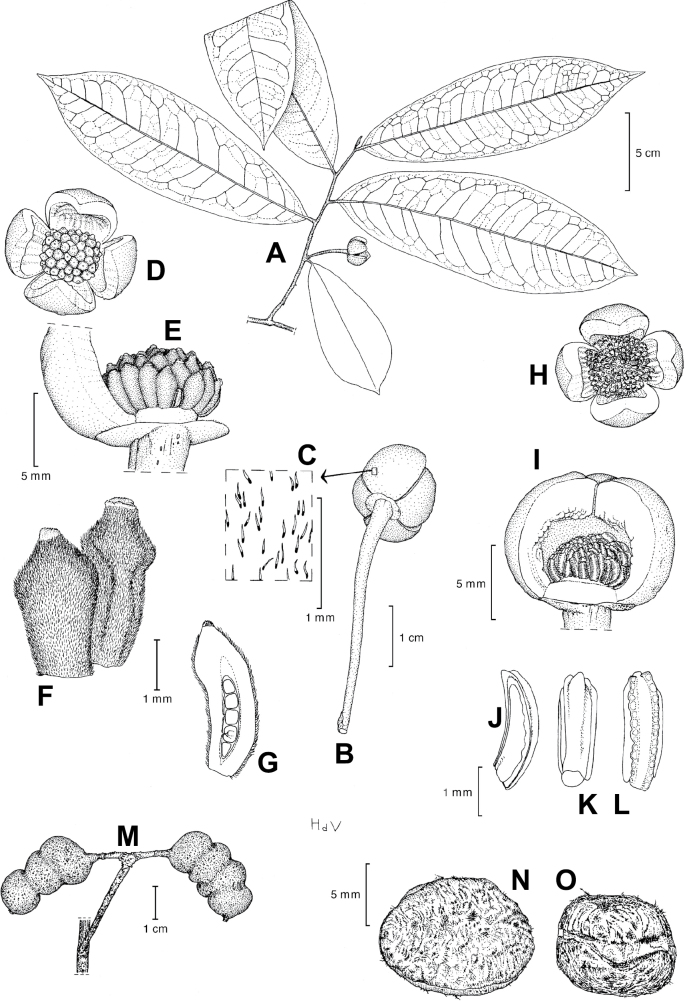
*Uvariopsislovettiana* Couvreur & Q.Luke **A** flowering branch **B** male flower, semi-bottom view **C** detail of outer petal indumentum **D** female flower, top view **E** detail of female flower, three petals removed, side view **F** carpels **G** longitudinal section of carpel **H** male flower, top view **I** male flower, one petal removed, side view **J** stamen, lateral view **K** stamen, back view **L** stamen, front view **M** fruit **N** seed, top view **O** seed, side view. **A–L** from Couvreur 97b (type) **M–O** from Luke 10369. Drawing by Hans de Vries, from “A new species of *Uvariopsis* (Annonaceae), endemic to the Eastern Arc Mountains of Tanzania” Blumea 55:1, fig. 1, p. 69 (Couvreur and Luke 2010), with permission from Blumea.

##### Preliminary conservation status.

This species is endemic from Tanzania. It has previously been assessed preliminarily as Near Threatened based on IUCN criterion B by Couvreur and Luke (2010). Since then, several specimens have been found south-west of the Udzungwa Mountains and in the Morogoro region. Its EOO is thus estimated at 20,766 km^2^ and its AOO is estimated at 68 km^2^. It would fall under the Endangered EN category based on AOO, but no other subcriteria (a, b or c) is met. Following IUCN criterion B, we thus assigned a preliminary conservation status of Least Concern LC.

**Figure 72.**
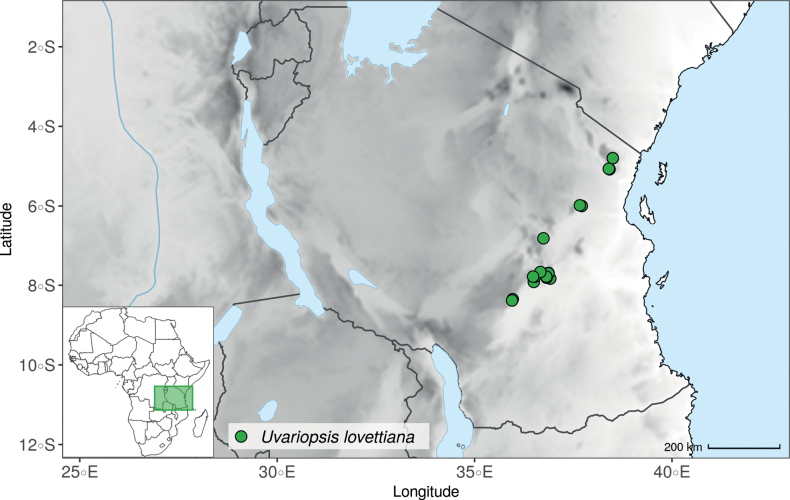
Distribution map of *Uvariopsislovettiana*. Shades of grey represent elevation, from white (sea level) to darker grey (higher elevation). The inset shows the extent of the map over Africa.

##### Additional specimens examined.

Tanzania – Iringa • C. Frimodt-Møller TZ95 (DSM, K), Luhega Forest Reserve, Iringa District, T7; 8°21'S, 35°58'E; alt. 1600 m; 20 Jan. 1997 • C.E. Bracebridge 102 (MO); Kilolo District, Kilombero Nature Reserve, Udekwa Village; 7°48'06'S, 36°30'21'E; alt. 1415 m; 22 Oct. 2009 • D.W. Thomas 3921 (K, MO); Iringa Rural District, Mwanihana Forest Reserve, above Sanje village.Forest on steep slope with small streams and swamps, and patches of elfin forest on ridge top; 7°50'S, 36°55'E; alt. 1400 m; 10 Oct. 1984 • L. Festo 2014 (MO); Kilolo District, Udzungwa Mountains National park, Udzungwa forest, c. 10 km from Msolwa village at source of Msolwa stream; 7°42'11'S, 36°52'23'E; alt. 1350 m; 24 Oct. 2005 • M.A. Mwangoka 8670 (MO); Kilolo District, Uzungwa Scarp Forest Reserve, Idegenda area, E part of Idegenda village; 8°23'S, 35°57'E; alt. 1650 m; 04 Dec. 2012 • W.A. Rodgers 2252 (DSM, K); Iringa Rural District, Udekwa village, forest blowk to East. West of Kilombero F.R; 7°55'S, 36°30'E; alt. 1676 m; Oct. 1982 • W.R.Q. Luke 10369 (K, MO); Kilolo District, Ndundulu Forest reserve, Camp 589 – Camp 590; 7°47'S, 36°29'E; alt. 1440 m; 06 Sep. 2004 • W.R.Q. Luke 6722 (MO); Iringa Rural District, Udzungwa Mountain NP Mt Luhombero Pt 129-131; 7°47'S, 36°32'E; alt. 1440 m; 27 Sep. 2000 • W.R.Q. Luke 7730 (EA, K, NHT), Pt 211; 7°47'S, 36°49'E; alt. 1080 m; 23 Sep. 2001 • W.R.Q. Luke 9195 (EA, K), Pt 370-371; 7°40'S, 36°40'E; alt. 1780 m; 16 Oct. 2002 – Morogoro • A.R. Marshall 1116 (K), Gologolo – PSP10. Uddzungwa Nationa Park. Udzungwa Mountains; 7°41'31.36'S, 36°52'21.02'E; alt. 1772 m; 07 Aug. 2007 • A.R. Marshall 2010 (K); Kilombero District, Ndundulu – Kipunji Plot 1. Kilombero Nature Reserve. udzungwa Mountains; 7°48'17.36'S, 36°30'09.69'E; alt. 1425 m; 30 Jul. 2010 • A.R. Marshall 2114 (K), Udzungwa Forest, Kipunji; 7°48'14.36'S, 36°30'10.01'E; 28 May. 2011 • M.A. Mwangoka 3973 (MO); Mvomero District, Kanga Forest Reserve, Kwamndolwa area; 6°00'S, 37°43'E; 29 Jun. 2005 • M.A. Mwangoka 7178 (MO); Kilosa District, Lumuma Ward, Lunezi Village, Mianzini forest between Lunenzi and Manyomis subvillage; 6°49'18'S, 36°44'36'E; alt. 1530 m; 15 Dec. 2010 • Y.S. Abeid 2625 (MO); Mvomero District, Kanga Forest Resrve, Work Unit 1, North East Transect, 500 m from centre point; 5°59'S, 37°40'E; alt. 900 m; 21 Feb. 2006 – Tanga • A.L. Borhidi 82177 (DSM); Lushoto District, SE corner of Mazumbai University Forest Reserve with ‘Lundgren's plot'; 4°48'S, 38°30'E; alt. 1400 m; 24 Feb. 1982 • B.J. Harris 6257 (DSM), West Usambara Mountains, Mwazumbai; 4°48'S, 38°30'E; alt. 1450 m; 06 Apr. 1972 • C.J. Kayombo 1406 (MO); Korogwe District, T3. Ambangulu Tea Estate, Forest Reserve 5 km W of Ambangulu Tea Factory above Estate road to Makunga; 5°04'48'S, 38°25'05'E; alt. 1300 m; 03 Nov. 1998 • L.-P.M.J. Dagallier 64 (DSM, MPU, WAG); Korogwe District, East Usambaras, Ambangulu, top of the mountain above the tea plantations; 5°04'08.38'S, 38°24'30.45'E; alt. 1316 m; 20 Nov. 2019 • L.-P.M.J. Dagallier 66 (DSM, K, MO, MPU, P, WAG); Korogwe District, East Usambaras, Ambangulu, top of the mountain above the tea plantations; 5°03'57.38'S, 38°23'59.43'E; alt. 1208 m; 20 Nov. 2019.

#### ﻿ Uvariopsis noldeae

Taxon classificationPlantaeMagnolialesAnnonaceae

Exell & Mendonça, Bol. Soc. Brot. sér. 2, 25: 101 (1951)

Figs 73 , 74

##### Type.

Angola – Malanje • I. von Nolde 576 (holotype: BM! (BM000554081)), Quela; 9°16'06.17'S, 17°04'12.72'E; alt. 1200 m; Dec. 1938.

##### Description.

Tree height and D.B.H. unknown; young branches sparsely pubescent to glabrous, old branches glabrous. Petiole 1–2.5 mm long, ca. 1.5 mm wide, sparsely pubescent to glabrous. Leaf lamina 110–204 mm long, 40–60 mm wide, length:width ratio ca. 3.4, elliptic to obovate, coriaceous, base rounded, apex acuminate, acumen ca. 21 mm long, surface above glabrous, surface below glabrate to glabrous when young, glabrous when old; midrib impressed above, raised below, glabrous above, glabrous below; secondary veins 11–12 pairs, weakly brochidodromous, impressed above, raised below; tertiary veins reticulate. Flowers unisexual, male and female flowers dimorphic, on same individuals (plant monoecious). Flower buds ovoid to conical. Male inflorescences borne on trunk, composed of 1 flower. Peduncle inconspicuous. Flower pedicel 2–4 mm long, ca. 0.5 mm in diameter, pubescent. Bracts 1 to 2 at base, upper bract 0.5–1 mm long, 0.5–1 mm wide, broadly ovate, pubescent outside, glabrous inside. Sepals 2, 1–2 mm long, ca. 2 mm wide, broadly ovate, pubescent outside, glabrous inside. Petals 4, 6.5–10 mm long, 2–3.5 mm wide, length:width ratio 2.8–3.2, ovate, free, pubescent outside, glabrous inside, brownish red outside, cream inside. Stamens numerous (exact number unknown), 0.3–0.6 mm long, 0.1–0.4 mm wide, anthers linear, connective prolongation truncate. Female inflorescences borne on trunk, composed of 1 flower. Flower pedicel 70–83 mm long, ca. 1 mm in diameter, sparsely pubescent. Bracts 1 to 2 at base, upper bract ca. 1.5 mm long, ca. 1.5 mm wide, broadly ovate, pubescent outside, glabrous inside. Sepals 2, 3–4 mm long, ca. 4 mm wide, broadly ovate, pubescent outside, glabrous inside, color unknown. Petals 4, 14–16 mm long, 5–8 mm wide, length:width ratio 1.8–3.2, ovate, free, pubescent outside, glabrous inside, brownish red outside, cream inside. Carpels 40 to 45, ca. 2 mm long, ca. 1 mm wide, velutinous, free, ovules ca. 20 per ovary, biseriate; stigma flat. Fruits unknown.

**Figure 73.**
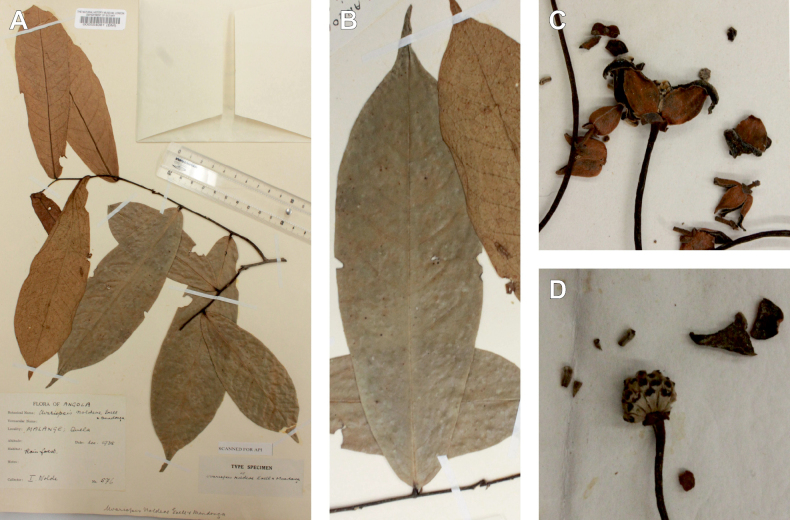
*Uvariopsisnoldeae* Exell & Mendonça **A** full specimen sheet with branch with leaves **B** leaf, upper side **C** flowers, side view **D** detail of female flower, petals removed, side view. **A–D** Nolde 576 (type). Photos Léo-Paul Dagallier.

##### Distribution.

Endemic to Zambezian Region. Known from only one locality in Angola: Quela in Malange region.

##### Habitat and ecology.

Montane rain forests. Altitude ca. 1700 m asl.

##### Phenology.

Flowers collected in December.

##### Notes.

*Up.noldeae* closely resembles *Up.solheidii* in having similar leaves and flowers. However, *Up.noldeae* differs from *Up.solheidii* in having young branches and petioles sparsely pubescent to glabrous (vs. tomentose to shortly tomentose) and leaf midrib glabrous below (vs. tomentose to glabrous below). *Up.noldeae* has very dimorphic flowers with pedicels of male flowers 2–4 mm long (vs. 9–30 mm long in *Up.solheidii*) and pedicels of female flowers 70–83 mm long (vs. 30–198 mm long in *Up.solheidii*).

**Figure 74.**
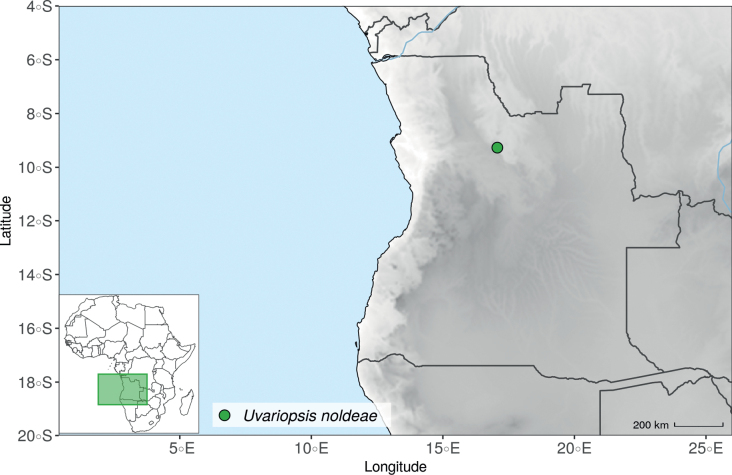
Distribution map of *Uvariopsisnoldeae*. Shades of grey represent elevation, from white (sea level) to darker grey (higher elevation). The inset shows the extent of the map over Africa.

##### Conservation status.

This species is known from a single specimen from Angola, collected in 1938, outside any protected area. Angola is one of the least botanically explored countries (Sosef et al. 2017), with very few Annonaceae collected since the beginning of the civil war in 1975 (see https://bio-dem.surge.sh/, Zizka et al. 2021). It has been previously been assessed as Data Deficient DD (Cosiaux et al. 2019d).

#### ﻿ Uvariopsis oligocarpa

Taxon classificationPlantaeMagnolialesAnnonaceae

Dagallier & Couvreur sp. nov.

urn:lsid:ipni.org:names:77326974-1

**Figure 75.**
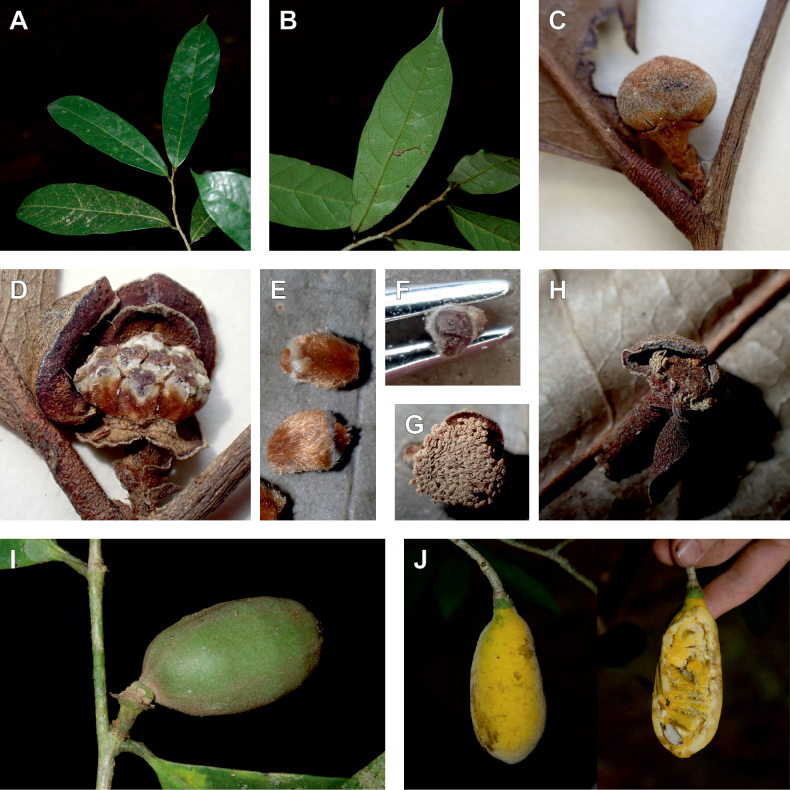
*Uvariopsisoligocarpa* Dagallier & Couvreur **A** leaves, upper side **B** leaf, lower side **C** flower bud, side view **D** female flower bud, two petals removed, side view **E** carpels, side view **F** carpel, top view **G** receptacle of male flower with stamens, top view **H** male flower, one petal removed and one petal falling down, side view **I** fruit with single monocarp, side view, note the hairs on the monocarp **J** mature fruit, entire (left), longitudinal section (right), side view. **A, B, J** Koivogui 98 **C** de Wilde 3656 **D, F** Merello 1380 **H** de Koning 2657 **I** Koivogui 244 **E, G** van der Laan 640. Photos **A, B, I, J** Ehoarn Bidault (CC BY-NC-ND 3.0) **C–H** Léo-Paul Dagallier.

**Figure 76.**
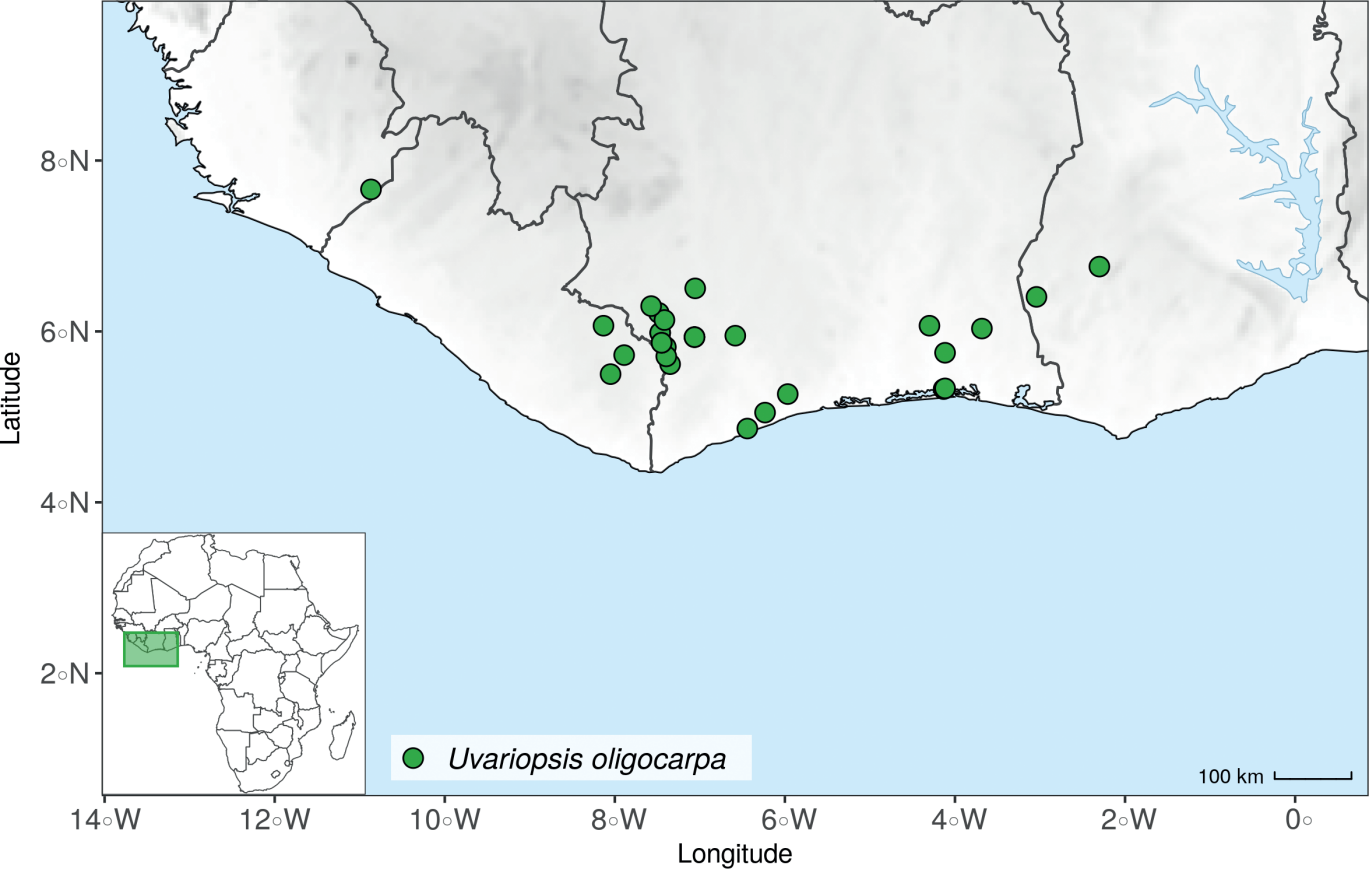
Distribution map of *Uvariopsisoligocarpa*. Shades of grey represent elevation, from white (sea level) to darker grey (higher elevation). The inset shows the extent of the map over Africa.

Figs 75 , 76 , Table 6

##### Type.

Ghana – Western Region • M.C. Merello 1380 (holotype: MO! (MO3055200); isotype: WAG! (WAG.1570507)), Bia National Forest and Production Reserve. Secondary logging roads west from MIM Timber Company Camp; 6°24'15'N, 3°02'30'W; alt. 140 m; 04 Mar. 1996.

##### Diagnosis.

*Up.oligocarpa* resembles *Up.bisexualis*, *Up.congensis* and *Up.zenkeri* when sterile, having elliptic leaves generally less than 15 cm long with decurrent base, but it differs clearly when fertile. *Up.oligocarpa* has unisexual flowers (vs. bisexual flowers in *Up.bisexualis*) and less carpels (13 to 20 vs. 20 to 40 in *Up.congensis*), which results in generally 1 to 3 monocarps reaching maturity on the ripe fruit (vs. up to 15 mature monocarps in *Up.congensis*). The monocarps of *Up.oligocarpa* are pubescent to sparsely pubescent (vs. glabrate to glabrous in *Up.congensis* and tomentose in *Up.zenkeri*), not constricted between the seeds (vs. strongly to slightly constricted between the seeds in *Up.congensis* and *Up.zenkeri*), yellow to orange when ripe and brown to dark brown when dry (vs. green to red when ripe and black when dry in *Up.congensis*), and sessile to very shortly stipitate with stipes shorter than 1 mm (vs. sessile to stipitate with stipes between 0 and 7 mm in *Up.congensis*). In short, *Up.oligocarpa* can be distinguished from all the other *Uvariopsis* species by the combination of the following characters: plant with glabrous branches and elliptic leaves less than 150 mm, unisexual flowers, female flowers with less than 21 carpels, fruits composed of 1–3 monocarps reaching maturity, monocarps pubescent to sparsely pubescent and not constricted between the seeds (Table 6).

##### Description.

Tree 2–3 m tall, D.B.H unknown; young branches glabrous, old branches glabrous. Petiole 4–6 mm long, 1.7–2.5 mm wide, glabrous. Leaf lamina 90–149 mm long, 32–53 mm wide, length:width ratio 2.2–4, elliptic, papyraceous to coriaceous, base acute to decurrent, apex acuminate, acumen 10–21 mm long, surface above glabrous, surface below glabrous; midrib slightly impressed above, raised below, glabrous above, glabrous below; secondary veins 5–11 pairs, brochidodromous to weakly brochidodromous, impressed above, raised below; tertiary veins reticulate. Flowers unisexual, male and female flowers similar, on same individuals (plant monoecious). Flower buds globose. Male and female inflorescences axillary or sometimes borne on branches, composed of 1 flower. Peduncle inconspicuous. Flower pedicel 2–7 (20) mm long, 1–1.5 mm in diameter, pubescent. Bracts 1 to 3 at base, upper bract 0.5–3 mm long, 0.5–2 mm wide, broadly ovate, pubescent outside, glabrous inside. Sepals 2, 0.7–2.5 mm long, 2–3 mm wide, depressed ovate, free to basally fused, pubescent outside, glabrous inside, color unknown. Petals 4, 4–6.5 (10) mm long, 2.5–6 (10) mm wide, length:width ratio 1–1.4, broadly ovate, free, pubescent outside, glabrous inside, yellowish cream to yellow outside. Male flowers: stamens 300 to 400, 0.5–1.6 mm long, 0.1–0.3 mm wide, anthers linear, connective prolongation truncate. Carpels ca. 20, ca. 1 mm long, ca. 0.5 mm wide, free, stunted, sterile (seen on only one male flower). Female flowers: carpels 13 to 20, 1–2 mm long, 0.5–1.1 mm wide, velutinous, free; stigma ca. 0.1 mm long, ca. 0.5 mm wide, coiled, glabrous. Fruiting pedicel 4–10 mm long, 1–3 mm in diameter, glabrous. Monocarps, 1–3, 25–60 mm long, 11–35 mm wide, length:width ratio 1.7–2.7, cylindrical, smooth, pubescent to sparsely pubescent, green ripening yellow to orange (orange-brown when dry), sessile to very shortly stipitate; stipe 0–1 mm long, 2–3 mm wide, pubescent. Seeds 8–13 per monocarp, biseriate, 8.5–14 mm long, 7–8 mm wide, ellipsoid to semicircular, in an orange pulp.

##### Distribution.

Endemic to Upper Guinean Domain of the Guineo-Congolian Region: Ghana, Ivory Coast, Liberia, Sierra Leone.

##### Habitat and ecology.

Lowland mature or secondary forest. Altitude: 140–285 m asl.

##### Phenology.

Flowers collected from February to June and in November. Fruits collected from January to March and from June to November.

##### Etymology.

The specific epithet refers to the low number of monocarps reaching maturity in this species.

##### Preliminary conservation status.

This species is distributed in West Africa, from Sierra Leone to Ghana. Its EOO is estimated at 147,428 km^2^ and its AOO at 96 km^2^. Based on AOO, it would qualify for Endangered EN under B2, the other criteria (a, b, c) are not met. It is thus assigned a preliminary conservation status of Least Concern LC.

##### Additional specimens examined.

Ghana – Brong-Ahafo Region • J.B. Hall 44553 (K), Desiri F.R; 6°45'30.2'N, 2°18'06.66'W; 20 Jun. 1973. Ivory Coast – Abidjan • F.M. van der Laan 640 (U, WAG), Centre Orstom Adiopodonmé, 17 km Dabou rd. from Abidjan. Jardin Botanique; 5°19'30'N, 4°08'W; 29 Jun. 1983 • J.J. Bos 10345 (WAG), Centre Orstom Adiopodonmé, 17 km Dabou road from Abidjan, Jardin Botanique; 5°20'N, 4°07'W; 16 Jun. 1978 – Adzopé • L. Aké Assi 12872 (G), Forêt de la Besso; 6°02'N, 3°41'W; 01 Apr. 1975 – Agboville • A.J.B. Chevalier 16721 (P), Bouroukrou, chemin de fer km 92;6°04'N, 4°18'W; 20 Jan. 1907 • L. Bernardi 8623 (G, K, P), in regione Yapo-Nord, 60–70 km ad septentrionem Abidjan; 5°45'N, 4°07'W; 14 Mar. 1962 – Guiglo • A. Bakayoko 140 (G, P, WAG), Zagné; 6°13'N, 7°29'W; 23 May. 2002 • A.J.B. Chevalier 19308 (P), Keeta, Bassin du moyen Cavally. Pays des Oubi: village de Ke'eta et environs; 5°59'N, 7°28'W; 09 Jul. 1907 • A.J.B. Chevalier 19347 (P), Keeta, Bassin du moyen Cavally. Pays des Oubi: village de Ke'eta et environs; 5°59'N, 7°28'W; 09 Jul. 1907 • L. Aké Assi 12088 (K), Forêt près de Ziriglo; 5°37'N, 7°21'W; 04 Jun. 1973 • L. Aké Assi 13218 (G), entre Taï et Grabo: Troya; 5°42'28.06'N, 7°23'42.49'W; 10 Jan. 1976 • L. Bernardi 8403 (G, K, P), De oppido Tienkulà, ad orientem per 5–9 km in silva alta; 6°08'N, 7°25'W; 02 Mar. 1962 • N. Stäuble 848 (G, MO), Taï; 5°52'N, 7°27'W; Feb. 1982 • N. Stäuble 849 (G, MO), Taï; 5°52'N, 7°27'W; 27 Dec. 1981 • P.R.J. Bamps 2600 (P), Zro; 6°17'45.96'N, 7°34'22.69'W; Apr. 1970 – San-Pédro • C.C.H. Jongkind 4737 (WAG), Forêt Classée Monogaga, just south of Sassandra – San Pedro road; 4°51'48'N, 6°26'30'W; 25 Mar. 2000 – Sassandra • C. Geerling 2328 (WAG), Dakpadou-Sago; 5°16'N, 5°58'W; 29 Mar. 1968 • J. de Koning 2657 (BR, MO, WAG), near Fuyt plantation; 5°03'N, 6°14'W; 11 Nov. 1973 – Soubré • A.J.B. Chevalier 19061 (P), Guideko, Bassin de la moyenne Sassandra entre Guidéko et la Zozro; 5°57'N, 6°35'W; 10 Jun. 1907 • A.J.B. Chevalier 19222 (P), entre le moyen Sassandra et le moyen Cavally; 5°55'59.75'N, 7°03'46.35'W; 06 Jul. 1907 • A.J.B. Chevalier 19253bis (P), entre moy. Sassandra et moy. Cavally; 5°55'59.75'N, 7°03'46.35'W; 06 Jul. 1907 • A.J.B. Chevalier 19282 (P), entre le moyen Sassandra et le moyen Cavally; 5°55'59.75'N, 7°03'46.35'W; 04 Jul. 1907 • M. Scouppe 222 (G), Taï National Park, P.N. de Taï, zone du Centre, secteur Soubré, groupe de transect VH, RL6; 5°43'21.19'N, 7°53'25.45'W; alt. 220 m; 28 Jan. 2010 • P.R.J. Bamps 2507 (P), Guézon – Buyo; 6°30'12.96'N, 7°03'18.22'W; Feb. 1970 – Unknown major area • A. de Rouw 334 (WAG), Pauleoula; 5°49'N, 7°24'W; 08 Dec. 1986. Liberia – Grand Gedeh • J.J.F.E. de Wilde 3656 (K, MA, MO, P, U, WAG), Eastern Province, Putu District. Near Kanweake, a village c. 70 km S. of Chiehn (Zwedru village); 5°30'N, 8°03'W; 26 Mar. 1962 • J.T. Baldwin jr 7091 (K), Zwedru; 6°04'N, 8°08'W; 09 Aug. 1947. Sierra Leone – Eastern Province • B. Saradugu 57 (K, SL, WAG), Gola National Park, central block; 7°39'42.7'N, 10°52'02.6"W; alt. 285 m; 31 Oct. 2013.

#### ﻿ Uvariopsis pedunculosa

Taxon classificationPlantaeMagnolialesAnnonaceae

(Diels) Robyns & Ghesq., Ann. Soc. Sci. Bruxelles, Ser. B liii. 321 (1933)

**Figure 77.**
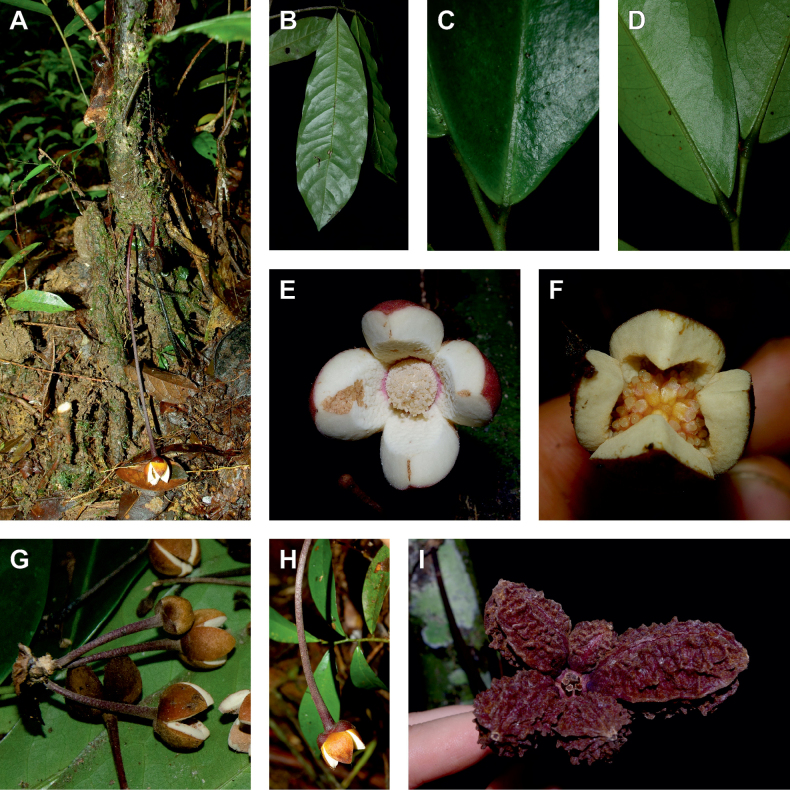
*Uvariopsispedunculosa* (Diels) Robyns & Ghesq **A** base of trunk with flower **B** leaf, lower side **C** base of leaf, upper side **D** base of leaf, lower side **E** male flower, top view **F** female flower, top view **G** inflorescence **H** flower, side view **I** fruit, top view. **A, C, D, F, H** Couvreur 594 **B, G** Bidault 2300 **E** Couvreur 878 **I** Couvreur 885. Photos **A, C–F, H, I** Thomas Couvreur **B, G** Ehoarn Bidault (CC BY-NC-ND 3.0).

**Figure 78.**
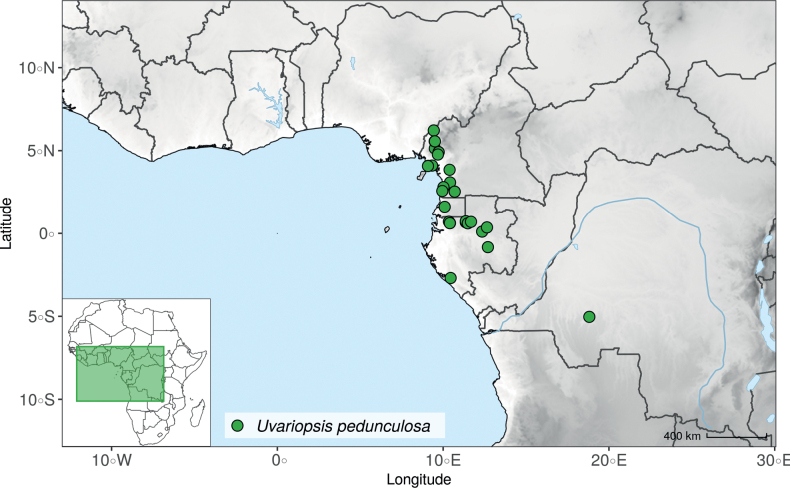
Distribution map of *Uvariopsispedunculosa*. Shades of grey represent elevation, from white (sea level) to darker grey (higher elevation). The inset shows the extent of the map over Africa.

Figs 77 , 78 , 80P–S

≡ Tetrastemma pedunculosum Diels, Bot. Jahrb. Syst. 53(3–5): 441 (1915). Type. Cameroon – South Region • G.A. Zenker 3868 (holotype: B! (B 10 0153122); isotypes: BM! (BM000554078), BR! (BR0000008824196, BR0000008824226), E! (E00718574), HBG! (HBG502486), K! (K000199041), M! (M0107937), P! (P00362601, P00362599), US! (US00098850)), Bipinde, Urwaldgebiet; 3°05'N, 10°25'E; 1909.
= Uvariopsis vanderystii Robyns & Ghesq., Ann. Soc. Sci. Bruxelles, Ser. B liii. 64 (1933). Type. Democratic Republic of the Congo – Bandundu • H.J.R. Vanderyst 9973 (holotype: BR! (BR0000008824387)), Kikwit; 5°02'S, 18°49'E; 1921.

##### Description.

Shrub to tree 2–8 m tall, D.B.H 1.5–6.5 cm; young branches pubescent to glabrous, old branches glabrous. Petiole 2–5 mm long, 1.8–3 mm wide, glabrous. Leaf lamina 172–290 mm long, 57–108 mm wide, length:width ratio 2.3–3.9, elliptic to obovate, coriaceous, base acute, apex acuminate, acumen 3–23 mm long, surface above glabrous, surface below glabrous; midrib impressed above, raised below, glabrous above, glabrous below; secondary veins 8–15 pairs, brochidodromous to weakly brochidodromous, impressed above, raised below; tertiary veins reticulate. Flowers unisexual, male and female flowers dimorphic, on same individuals (plant monoecious). Flower buds globose. Male inflorescences borne on trunk, composed of 1 flower. Peduncle inconspicuous. Flower pedicel 14–320 mm long, 0.9–3 mm in diameter, sparsely pubescent to glabrous. Bracts 1 at base and 1 towards the middle or lower half of the pedicel, upper bract ca. 2 mm long, ca. 2 mm wide, broadly ovate, adpressed, semi clasping the pedicel. Sepals 2, 2–7 mm long, 6–7.5 mm wide, very broadly ovate, basally fused, enclosing the petals in bud, sparsely pubescent outside, glabrous inside, dark purplish red. Petals 4, 9–12 mm long, 5–7 mm wide, length:width ratio 1.3–2.2, broadly elliptic to elliptic, fused at base over 20–40 % of their length, pubescent outside, glabrous inside, cream outside, orangish to reddish brown inside. Stamens 650 to 900, 0.5–1 mm long, 0.1–0.3 mm wide, anthers linear, connective prolongation truncate or absent. Female inflorescences borne on trunk, composed of 1 flower. Flower pedicel 80–325 mm long, 1–3 mm in diameter, sparsely pubescent. Bracts 1 at base and 1 towards the middle or lower half of the pedicel, upper bract ca. 2 mm long, ca. 2 mm wide, broadly ovate, adpressed, semi clasping the pedicel. Sepals 2, 6–18 mm long, 9–12 mm wide, very broadly ovate, basally fused, enclosing the petals in bud, sparsely pubescent outside, glabrous inside, dark purplish red. Petals 4, 8–14 mm long, 6–10 mm wide, length:width ratio 1.3–2.2, broadly elliptic to elliptic, fused at base over 20–40 % of their length, pubescent outside, glabrous inside, cream outside, orangish to reddish brown inside. Carpels 50 to 140, 2–4 mm long, 1–1.5 mm wide, pubescent, free; stigma 0.2–0.5 mm long, 0.2–0.5 mm wide, globose. Fruiting pedicel 129–197 mm long, 2–2.2 mm in diameter, pubescent to glabrous. Monocarps, 4–9, 10–17 mm long, 7–11 mm wide, length:width ratio 1.3–1.6 (measures taken from unripe fruits), cylindrical, very verrucose, pubescent, blackish brown, sessile.

##### Distribution.

Endemic to Lower Guinean Domain of the Guineo-Congolian Region: Cameroon, Democratic Republic of the Congo, Gabon.

##### Habitat and ecology.

Lowland to submontane mature or secondary rain forests. Altitude: 200–1100 m asl.

##### Phenology.

Flowers collected in from February to June and from October to December. Fruits collected in from April to June and in November and December.

##### Notes.

*Uvariopsispedunculosa* resembles *Up.dioica*, *Up.guineensis* and *Up.solheidii* in having elliptic to obovate leaves, with acute base and acuminate apex. It has female flower pedicels 80–325 mm long, which is longer than in all *Uvariopsis* species, except in *Up.congolana* (200–400 mm long) and *Up.solheidii* (up to 198 mm long). It differs from *Up.congolana* in having 4 petals (vs. 3 petals) and verrucose monocarps (vs. longitudinally ridged monocarps). It differs from *Up.solheidii* in having broadly elliptic to elliptic petals, fused at base over 20–40% of their length (vs. ovate to narrowly ovate and free petals). In the Revision of the Flora of West Tropical Africa, Keay (Keay 1952) synonymised the name *Uvariopsispedunculosa* (Diels) Robyns & Ghesq. with *Up.dioica* (Diels) Robyns & Ghesq. Based on the examination of many herbarium specimens (including the type specimen), we found that the specimens previously identified under the name *Up.pedunculosa* were morphologically very different from *Up.dioica*. Indeed, *Up.dioica* has free petals whereas *Up.pedunculosa* has basally fused petals. Rather, *Up.pedunculosa* is morphologically similar to specimens identified as *Up.vanderystii* Robyns & Ghesq. Thus, in the Annonaceae Flora of Cameroon (Couvreur et al. 2022), we synonymized *Uvariopsisvanderystii* Robyns & Ghesq. with *Uvariopsispedunculosa* (Diels) Robyns & Ghesq. Phylogenetically, our results show that the holotype of *Up.pedunculosa* (Zenker 3868) did not cluster with *Up.dioica*, but with the specimens previously identified as *Up.vanderystii* (e.g. Couvreur 602) (Fig. 1, Suppl. materials 1, 2) confirming the synonymisation of the name *Up.vanderystii* with *Up.pedunculosa*.

##### Preliminary conservation status.

This species has already been assessed (as *Uvariopsisvanderystii*) as Vulnerable VU under criteria B2ab(iii), mainly because of continuing decline in AOO due to the threat of agricultural expansion (Cheek 2014c). Here, the EOO of *Uvariopsispedunculosa* is estimated at 463,493 km^2^ and its AOO is estimated at 96 km^2^. It occurs in more than 20 locations. Based on AOO, it would qualify for Endangered EN B2b(iii), but do not meet any other subcriteria (a or c). Given the threat mentioned above, we assign a preliminary conservation status of Near Threatened NT.

##### Additional specimens examined.

Cameroon – Littoral • T.L.P. Couvreur 1173 (K, MPU, P, WAG, YA), Mapubi, 30 km before Edea on Yaoundé-Edea road. On forestry road, 5 km direction to Sanaga river; 3°50'44.56'N, 10°23'21.98'E; alt. 219 m; 28 Feb. 2018 – South Region • G.P. Tchouto Mbatchou 3242 (KRIBI, SCA, WAG), Campo-Ma'an area, Massif des Mamelles, along the path to Mamelles highlands; 2°33'57'N, 9°56'58'E; alt. 280 m; 23 Apr. 2001 • G.P. Tchouto Mbatchou ELEX15 (WAG), Campo Ma'an area, Mont d' Elephant, Bidou area towards Hevecam, path to the summit; 2°47'52'N, 10°01'12'E; alt. 180 m; 16 Oct. 2001 • G.P. Tchouto Mbatchou ONOX182 (WAG), Campo Ma'an area, Onoyong, between plots ONO1 and 10; 2°31'39'N, 10°41'49'E; alt. 360 m; 18 Mar. 2001 • G.P. Tchouto Mbatchou ONOX274 (WAG), Campo Ma'an area, Onoyong, between plots ONO1 and 10; 2°31'39'N, 10°41'49'E; alt. 360 m; 18 Mar. 2001 – South-West Region • D.W. Thomas 2756 (K, MO), on the southern slope of Mount Cameroon, above Batoke; 4°05'N, 9°06'E; alt. 500 m; 29 Dec. 1983 • D.W. Thomas 7364 (P), Takamanda Forest Reserve; 6°13'N, 9°26'E; alt. 500 m; 30 Apr. 1987 • G.K. Gottsberger 170307/11 (ULM, WAG), c. 2 km from Banyang Mbo Research Station; 5°08'N, 9°30'E; 17 Mar. 2007 • G.W.J. Mildbraed 10745 (B, K), Likomba – Pflanzung, 15–35 km NE von Victoria; 4°06'N, 9°20'E; alt. 50 m; 03 Dec. 1928 • J.F. Villiers 1427 (P), Massif Ntali, crète sommitale, 30 km SE Mamfé; 5°33'53.69'N, 9°29'26.07'E; alt. 1100 m; 14 Jun. 1982 • R.G. Letouzey 13849 (MO, P), Crète de Nta Ali, entre cotes 1009 et 1202; 30 km SE Mamfe (feuille IGN 1/200.000 Mamfe); 5°33'39.99'N, 9°30'09.81'E; alt. 1266 m; 19 Jun. 1975 • T.L.P. Couvreur 1063 (MPU, WAG, YA), on forest trail, north of Ngomboku village; 4°54'48.49'N, 9°43'28.73'E; alt. 793 m; 06 Apr. 2016 • T.L.P. Couvreur 1066 (MPU, WAG, YA), on forest trail, north of Ngomboku village; 4°54'38.74'N, 9°43'48.18'E; alt. 815 m; 06 Apr. 2016 • T.L.P. Couvreur 517 (MPU, YA); Fako, on trail trough palm oil plantation, 3 km before lava flow and Seme Beach hotel when coming from Limbe; 4°04'28.6'N, 9°05'06.64'E; alt. 479 m; 18 Oct. 2013 – Unknown major area • W.G. Gosline 244 (K, MO, P, WAG, YA), Kupe-Muanenguba Division, Ajang saprophyte plot; 4°46'N, 9°41'E; alt. 950 m; 01 Dec. 1999. Equatorial Guinea – Centro Sur • B. Senterre 2989 (BRLU), SO du Parc National de Monte Alén, 2 km au NE du site de traversée du rio Uolo pour aller aux cataractas; 1°36'33.62'N, 10°05'32.96'E; alt. 750 m; 23 Jun. 2002. Gabon – Estuaire • T.L.P. Couvreur 602 (MPU, YA), Crystal Mountains, on trail behind the dam at Tchimbélé; 0°37'02.43'N, 10°24'41.26'E; alt. 617 m; 16 Nov. 2013 • T.L.P. Couvreur 607 (MPU, YA), Crystal Mountains, on trail befind tunnel leading under dam; 0°36'47.64'N, 10°24'10.96'E; alt. 492 m; 16 Nov. 2013 – Nyanga • J.L.C.H. van Valkenburg 2940 (BR, LBV, WAG), Moukalaba Doudou, national park, south of the road to Kachimba; 2°41'36'S, 10°27'06'E; alt. 500 m; 22 Feb. 2004 – Ogooué-Ivindo • E. Bidault 2300 (BR, BRLU, LBV, MO, MO, P, WAG), concession forestière Rougier-Ivindo; 0°06'23.9'N, 12°21'06.3'E; alt. 380 m; 29 Oct. 2015 • M.S.M. Sosef 2241 (BR, K, LBV, MO, WAG), c. 30 km down the Ivindo River from the IRET Research Station, SW of Makokou; 0°21'30'N, 12°38'42'E; alt. 350 m; 07 Nov. 2005 – Ogooué-Lolo • G.M.P.C. Le Testu 8525 (P), region de Lastoursville, Lastoursville; 0°50'N, 12°42'E; 18 Nov. 1930 – Woleu-Ntem • J.M. Reitsma 2554 (LBV, MO, WAG), Inventory Oveng; primary rain forest, ca 25 km WSW of Mintsic; 0°44'N, 11°22'E; 08 Nov. 1986 • J.M. Reitsma 891 (LBV, MO, WAG), chantier Rougier-Océan, Oveng; 0°40'N, 11°22'E; alt. 760 m; 08 May. 1985 • T.L.P. Couvreur 594 (MPU, YA), 40 km from Sam, on raod main road to Medouneu; 0°42'14.82'N, 10°20'53.39'E; alt. 589 m; 14 Nov. 2013 • T.L.P. Couvreur 878 (LBV, WAG, YA), on road from Mitzic to Lalara (N2), just after the bridge over the Lara, c.500 m in forest; 0°36'15.66'N, 11°29'12'E; alt. 574 m; 15 Nov. 2015 • T.L.P. Couvreur 885 (LBV, WAG, YA), c.15 km south of Mitzic (N2), in Foreex logging conssession, 5 km after the bridge after the main base camp (Saint Germain, c.25 km from road N2), on abandoned logging road left to the main road; 0°41'51.34'N, 11°40'50.29'E; alt. 531 m; 16 Nov. 2015.

#### ﻿ Uvariopsis solheidii

Taxon classificationPlantaeMagnolialesAnnonaceae

(De Wild.) Robyns & Ghesq., Ann. Soc. Sci. Bruxelles, Ser. B liii. 321 (1933)

**Figure 79.**
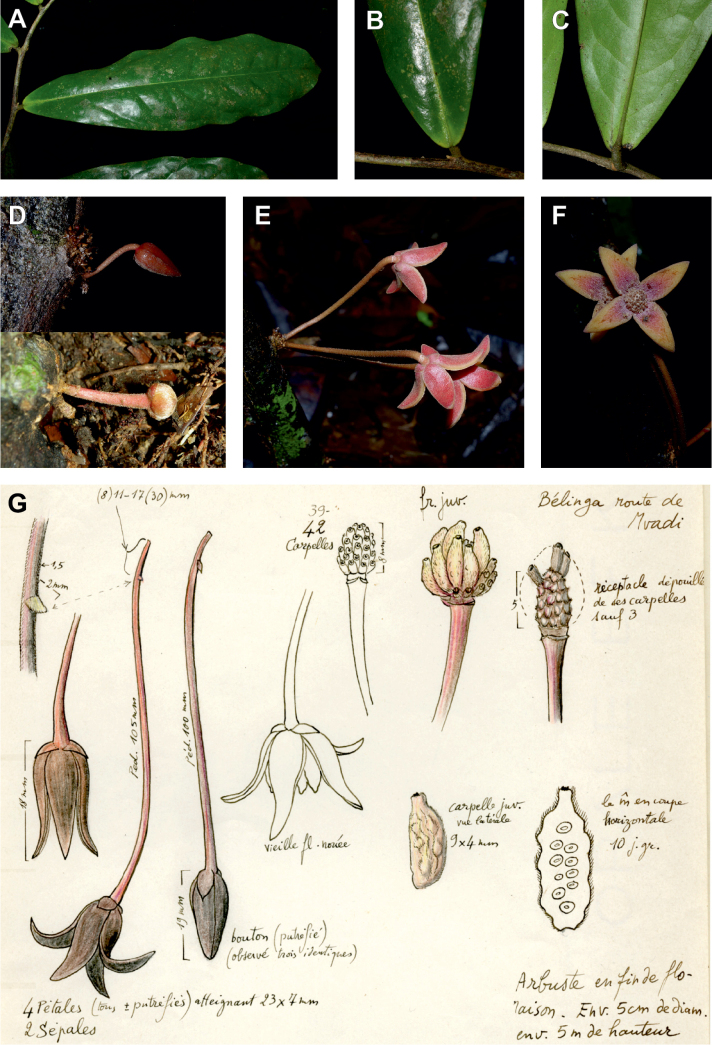
*Uvariopsissolheidii* (De Wild.) Robyns & Ghesq **A** leaf, upper side **B** leaf base, upper side **C** leaf base, lower side **D** flower buds, side view, var. solheidii (top), var. letestui (bottom) **E** inflorescence, side view **F** male flowers, top view **G** drawing of: flowers with details (left), young fruits (top right), young monocarp (bottom right). **A–C, D** (bottom) Couveur 550 **D** (top), **E, F** Couvreur 855 **G** Hallé 3474. Photos Thomas Couvreur. Drawing Nicolas Hallé, part of specimen Hallé 3474 (P).

**Figure 80.**
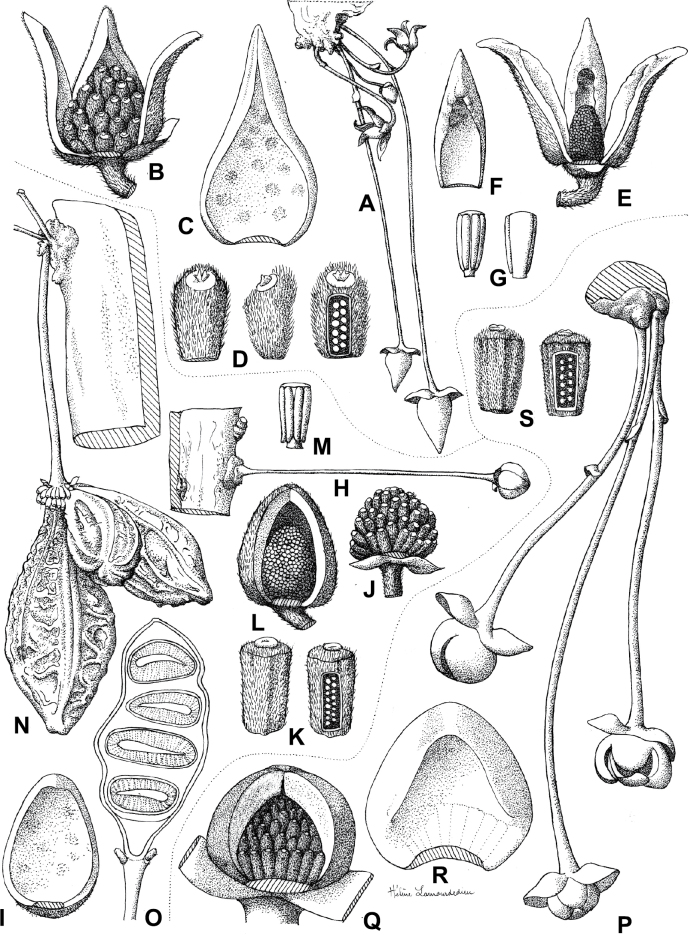
Uvariopsissolheidii(De Wild.)Robyns & Ghesq. –var.solheidii**A** male inflorescence **B** female flower, one petal removed, side view **C** petal of female flower, inner view **D** carpel, side and front views, detail of ovules **E** male flower, one petal removed, side view **F** petal of male flower, inner view **G** stamen, front and side views – var. letestui (Pellegr.) Dagallier & Couvreur **H** flower bud **I** petal of female flower, inner view **J** female flower, all four petals removed, side view **K** carpel, front view and detail of ovules **L** male flower, one petal removed, side view **M** stamen, front view **N** fruit **O** longitudinal section of **A** monocarp *Uvariopsispedunculosa* (Diels) Robyns & Ghesq **P** female inflorescence **Q** female flower, one petal removed, side view **R** petal of female flower, inner view **S** carpel, side view and detail of ovules. **A** from Tisserant 2422 **B–G** from Tisserant 804 **H–M** from Hallé 3060 **N, O** from Hallé 2975 **P–S** from Le Testu 8525. Drawings by Hélène Lamourdedieu, from Le Thomas (1969; pl. 54, p. 299), Publications Scientifiques du Muséum national d’Histoire naturelle, Paris.

**Figure 81.**
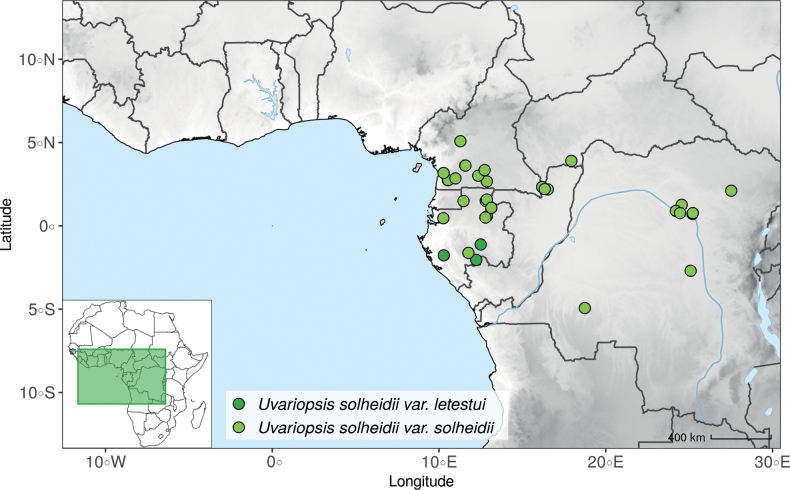
Distribution map of *Uvariopsissolheidii*. Shades of grey represent elevation, from white (sea level) to darker grey (higher elevation). The inset shows the extent of the map over Africa.

Figs 79 , 80A–O , 81

≡ Tetrastemma solheidii De Wild., Ann. Mus. Congo Belge, Bot. sér. 5, 3(1): 85 (1909). Type. Democratic Republic of the Congo – Orientale • A.F. Solheid 96 (holotype: BR! (BR0000008824240)); Banalia, Env. de Yambuya; 1°16'N, 24°33'E; 1906.
= Uvariopsis batesii Robyns & Ghesq., Ann. Soc. Sci. Bruxelles, Ser. B liii. 320 (1933). Type. Cameroon – South Region • G.L. Bates 1367 (holotype: BM! (BM000554077)), Bitye; 3°00'41.45“N, 12°21'04.18"E; 1919.
= Uvariopsis letestui Pellegr.; syn. nov. concerning Uvariopsisletestuivarletestui (see details under this variety).

##### Description.

Shrub to tree 1.5–8 m tall, D.B.H 2–6 cm; young branches tomentose to shortly tomentose, old branches glabrous. Petiole 2–5 mm long, 1.5–3 mm wide, tomentose to glabrous. Leaf lamina 131–260 (285) mm long, 40–93 mm wide, length:width ratio 2.1–3.9, elliptic to obovate, coriaceous, base acute to rounded to subcordate, apex attenuate to narrowly acuminate, acumen 4–20 mm long, surface above glabrous, surface below glabrous; midrib impressed above, raised below, glabrous above, tomentose to glabrous below; secondary veins 7–13 pairs, brochidodromous to weakly brochidodromous, impressed above, raised below; tertiary veins reticulate. Flowers unisexual, male and female flowers dimorphic, on same individuals (plant monoecious, but individuals with only pistillate flowers were also reported; e.g. Le Testu 8458). Flower buds long ovoid to conical. Male inflorescences borne on small thickenings of the trunk, mainly between the base towards 50 cm, up to ca. 2.50 m, above the female inflorescence, composed of 1 to 2 flowers. Peduncle inconspicuous. Flower pedicel 9–30 mm long, 0.5–1 mm in diameter, pubescent to sparsely pubescent. Bracts 1 to 2 at base and none to 1 towards the lower half of the pedicel, upper bract 1–2 mm long, 1.5–3 mm wide, broadly ovate, adpressed, semi clasping the pedicel, pubescent outside, glabrous inside. Sepals 2, 1.5–2 mm long, 1.5–2 mm wide, broadly ovate, free, pubescent outside, glabrous inside, brown to red. Petals 4, 5–10 mm long, 2.5–3.5 mm wide, length:width ratio 2–3.2, ovate to narrowly ovate, free, straight to curving outward at anthesis, pubescent to sparsely pubescent outside, glabrous inside, brown to light red outside, dull pink to dark purplish red inside. Stamens ca. 300, 0.2–0.5 mm long, 0.2–0.3 mm wide, anthers linear, connective prolongation truncate. Female inflorescences borne on small thickenings of the trunk, mainly between the base towards ca. 50 cm, up to ca. 2.50 m, below the male inflorescence, composed of 1 to 2 flowers. Flower pedicel 30–198 mm long, 1–1.5 mm in diameter, pubescent to glabrous. Bracts 1 to 2 at base and none to 1 towards the lower 30% of the pedicel, upper bract 1–2 mm long, 1.5–3 mm wide, broadly ovate, adpressed, semi clasping the pedicel, pubescent outside, glabrous inside. Sepals 2, 1.5–4 mm long, 2–5 mm wide, broadly ovate, free, pubescent outside, glabrous inside, brown to red. Petals 4, 7.5–17 mm long, 3.5–7 mm wide, length:width ratio 1.3–3.6, ovate to narrowly ovate, free, straight to curving outward at anthesis, pubescent outside, glabrous inside, brown to light red outside, dull pink to dark purplish red inside. Carpels 30 to 65, 1–3.5 mm long, 1–1.5 mm wide, velutinous, free; stigma globose. Fruiting pedicel 30–176 mm long, 1–2 mm in diameter, pubescent to glabrous. Monocarps, 1–5, 21–70 mm long, 12–30 mm wide, length:width ratio 1.8–3 (measures taken from both dried fruits and their associated specimen label, note that monocarps seem to sink when drying), cylindrical, with 4–6 longitudinal ridges and several transversal ridges or wrinkles, sparsely pubescent to glabrous, bright red, subsessile; stipe 0–4 mm long, 4–5 mm wide, pubescent to glabrous. Seeds 2–10 per monocarp, uniseriate to biseriate, 11–18 mm long, 10–12 mm wide, ellipsoid.

##### Distribution.

Element of the Lower Guinean Domain and Congolia Domain of the Guineo-Congolian Region: Cameroon, Central African Republic, Democratic Republic of the Congo, Gabon, Republic of the Congo.

##### Habitat and ecology.

Lowland to submontane mature or old secondary rain forests. Altitude: 0–1200 m asl.

##### Phenology.

Flowers collected from January to May, in November and December. Fruits collected in February, from April to August, in October and December.

##### Vernacular names.

Central African Republic: ‘Molo-Mobay’ in Bissongo (Tisserant 2422), ‘Ita ti ngaingai’ in Bambindjere (Fay 8384). Gabon: ‘Héli’ in Bakota (Hallé 3029).

##### Notes.

*Up.solheidii* resembles *Up.dioica*, *Up.guineensis*, and *Up.pedunculosa* in having elliptic to obovate leaves, with acute to rounded base and attenuate to acuminate apex. Leaf base of *Up.solheidii* can also be subcordate, and differs from these species in having young branches and petioles tomentose to shortly tomentose (vs. pubescent to glabrous) and in having cylindrical monocarps with longitudinal and transversal ridges (vs. smooth in *Up.dioica*, with a single longitudinal ridge in *Up.guineensis* and verrucose in *Up.pedunculosa*).

Here we make the two names *Uvariopsissolheidii* (De Wild.) Robyns & Ghesq. and *Uvariopsisletestui* Pellegr. synonyms. They have usually been distinguished based on the shape of their petals (narrowly ovate petals in *Ud.solheidii* vs. elliptic-ovate petals in *Ud.letestui*), the position of the bracts along the flowering pedicel, and the length of the flower pedicels (longer in *Ud.solheidii* than in *Ud.letestui*, but with an overlap) (Robyns and Ghesquière 1933a; Pellegrin 1948; Le Thomas 1965, 1969). Our observations indicate a high overlap between these supposedly distinctive characters, and difficulties to place some specimens in one or the other group. Our molecular phylogenies retrieve the specimens of *Ud.solheidii* as paraphyletic with the specimens of *Ud.letestui*. Together, they form a strongly supported monophyletic group (Fig. 1, Suppl. materials 1, 2), justifying further the two names actually represent the same species. Given the priority rule (Turland et al. 2018), the name *Uvariopsissolheidii* prevails. We make *Ud.letestui* as the variety Ud.solheidiivar.letestui, to recognize the existence of this morphotype in spite of overlap of some characters with the type variety (see notes under the Ud.solheidiivar.letestui).

##### Conservation status.

This species is widespread in central Africa. It has been assessed as Least Concern LC (Botanic Gardens Conservation International and IUCN SSC Global Tree Specialist Group 2019b). Here its EOO is estimated at 1,429,739 km^2^ and its AOO at 132 km^2^.

#### ﻿ Uvariopsis solheidii var. letestui

Taxon classificationPlantaeMagnolialesAnnonaceae

(Pellegr.) Dagallier & Couvreur, comb. et stat. nov.

urn:lsid:ipni.org:names:77326975-1

Figs 80H–O , 81

≡ Uvariopsis letestui Pellegr. syn. nov., Bull. Soc. Bot. France 95: 139 (1948). Type. Gabon – Ogooué-Lolo • G.M.P.C. Le Testu 8458 (holotype: P! (P00362609), sheet here designated; isotypes: BM! (BM000554079, BM000554080), P! (P00362607)), region de Lastoursville, Koulamotou; 1°07'S, 12°30'E; 20 Oct. 1930.

##### Description.

Male flowers with petals 5–7 mm long, ca. 3 mm wide, length:width ratio ca. 2, ovate, free, straight at anthesis. Female flowers with pedicel 30–110 mm long; petals 7.5–10 mm long, 4.5–7 mm wide, length:width ratio 1.3–1.8, ovate, free, straight at anthesis. Fruiting pedicel 30–110 mm long.

##### Distribution.

Endemic to Lower Guinean Domain of the Guineo-Congolian Region: Gabon.

##### Habitat and ecology.

Lowland to submontane mature rain forests. Altitude: 280–950 m asl.

##### Notes.

Up.solheidiivar.letestui differs from the type variety in having slightly different flowers. The male flowers of Up.solheidiivar.letestui have ovate petals 5–7 mm long, with a length:width ratio of ca. 2 (vs. ovate to narrowly ovate petals, 7–10 mm long with a length:width ratio of ca. 3). The female flowers of Up.solheidiivar.letestui have generally smaller flower pedicels (30–110 mm long vs. 63–198 mm long), and ovate petals 7.5–10 mm long, with a length:width ratio between 1.3 and 1.8 (vs. ovate to narrowly ovate petals, 9–17 mm long with a length:width ratio between 1.8 and 3.6). Both male and female flowers of Up.solheidiivar.letestui have straight petals at anthesis (vs. curved outward at anthesis in the type variety).

##### Additional specimens examined.

Gabon – Ngounié • T.O.B.E.B. Stévart 3993 (BR, LBV, MO, WAG), Birougou National Park, Massif du Chaillu, 40 km SE of Mbigou, 5.5 km E of Moukimbi village. MBG transect T59; 2°02'39'S, 12°14'01'E; alt. 780 m; 16 Feb. 2011 – Ogooué-Ivindo • J. Florence 1578 (P), Station d'Ipassa, 10 Km S de Makokou; 0°30'N, 12°47'E; alt. 500 m; 22 Jan. 1979 • N. Hallé 2975 (K, P, P), Bélinga; 1°05'N, 13°08'E; alt. 950 m; 03 Nov. 1964 • N. Hallé 3006 (P), Bélinga; 1°05'N, 13°08'E; 04 Nov. 1964 • N. Hallé 3029 (P), Bélinga; 1°05'N, 13°08'E; 05 Nov. 1964 • N. Hallé 3060 (P), Bélinga; 1°05'N, 13°08'E; 06 Nov. 1964 – Ogooué-Maritime • T.L.P. Couvreur 550 (MPU, YA), along road between Mandji and Rabi. About km 19 from Mandji; 1°46'05.10'S, 10°17'01.5'E; alt. 282 m; 08 Nov. 2013.

#### ﻿ Uvariopsis solheidii var. solheidii

Taxon classificationPlantaeMagnolialesAnnonaceae

Figs 80A–G , 81

##### Description.

Male flowers with petals 7–10 mm long, 2.5–3.5 mm wide, length:width ratio ca. 3, narrowly ovate, free, curving outward at anthesis. Female flowers with pedicel 63–198 mm long; petals 9–17 mm long, length:width ratio 1.8–3.6, narrowly ovate, free, curving outward at anthesis. Fruiting pedicel 53–176 mm long.

##### Distribution.

Element of the Lower Guinean Domain and Congolia Domain of the Guineo-Congolian Region: Cameroon, Central African Republic, Democratic Republic of the Congo, Gabon, Republic of the Congo.

##### Habitat and ecology.

Lowland to submontane mature or old secondary rain forests. Altitude: 0–1200 m asl.

##### Additional specimens examined.

Cameroon – Central Region • B.-A. Nkongmeneck 273 (P), Mt Ngoro à 58 km de Lint. Feuille IGN 1/200.000 LINTE; 5°04'53.52'N, 11°16'56.76'E; 17 Apr. 1982 • J.-M. Onana 2848 (K, SCA, WAG, YA), Mefou Proposed National Park, Ndanan 1, River N Didoumou; 3°37'N, 11°34'E; alt. 720 m; 26 Mar. 2004 • M.R. Cheek 11606 (K, SCA, WAG, YA), Mefou National Park Ndanan 1. Trail to Oncoba flag. (l after bridge); 3°37'13'N, 11°35'04'E; alt. 710 m; 10 Mar. 2004 – East Region • J.N. Asonganyi 310 (P), between Somalomo and Milon, 69 km SE Akonolinga, Map IGN 1/200.000 Akonolinga; 3°21'N, 12°44'E; 18 Jun. 1981 – South Region • A. Koufani 154 (P), Ngongonjie hill, near Akonetye; 2°40'N, 12°52'E; alt. 650 m; 30 Aug. 1978 • G.L. Bates s.n (BM), Bitye, Yaunde, Cameroons; 3°00'41.45'N, 12°21'04.18'E; 1919 • G.P. Tchouto Mbatchou 2843 (KRIBI, WAG), Efoulan, Ongongo and Nkolomekok hills in Akom II area; 2°44'43'N, 10°31'48'E; alt. 1000 m; 25 Apr. 2000 • G.P. Tchouto Mbatchou 3089 (KRIBI, SCA, WAG, YA), Efoulan; 2°44'45'N, 10°32'52'E; alt. 960 m; 04 Dec. 2000 • G.P. Tchouto Mbatchou EGONX191 (WAG), Campo Ma'an area, Akom II, Efoulan and Egongo hills; 2°44'46'N, 10°32'53'E; alt. 840 m; 06 Dec. 2000 • J.F. Villiers 893 (P), colline de Nkoltsia, 23 km NW de Bipindi; 3°10'30'N, 10°16'30'E; 27 Apr. 1974 • J.J.F.E. de Wilde 8373B (WAG), East face of Zingui hill, 21 km on the road from Ebolowa to Kribi (counted from the crossing at Ebolowa); 2°51'N, 10°59'E; alt. 760 m; 21 Jul. 1975. Central African Republic – Lobaye – Équipe C. Tisserant 2422 (BM, P, WAG); Mbaïki, Station de Boukoko; 3°54'N, 17°56'E; 26 Apr. 1952 • Équipe C. Tisserant 804 (K, P, WAG); Mbaïki, Forèt Soms bois; 3°54'N, 17°56'E; 23 Mar. 1948 – Sangha-Mbaéré • J.M. Fay 8384 (K, MO, P, WAG), Ndakan Gorilla Study Area; 2°20'N, 16°11'E; alt. 380 m; 26 May. 1988. Democratic Republic of the Congo – Bandundu • J.L.P. Louis 15474 (BM, K, P); 4°55'54'S, 18°44'42'E; alt. 470 m; 05 Dec. 1939 – Maniema • R.E. Gereau 7613 (MO), Secteur Bangengele; parc National proposé de la Lomani, ca. 330 m à l'Est du camp T7 Lomami et 1.3 km à l'Est de la rivière Lomami sur la piste au camp Kaka Barkata; 2°41'42'S, 25°05'18'E; alt. 450 m; 28 Apr. 2015 – Orientale • J. Bokdam 3674 (KIS, WAG); Banalia, 22 km along road from Kisangani to Bengamisa; 0°43'N, 25°12'E; 16 Nov. 1972 • J. Bokdam 4153B (WAG); Banalia, 23 km along road from Kisangani to Bengamisa; 0°46'N, 25°13'E; 31 May. 1973 • J.-B. Ndjango 549 (EPU, K, K, YBI); Haut-Uele, Asonga Hill, c. 75 km South of Isiro. Outskirts of the Ituri forest. Temporary camp; 2°06'35'N, 27°31'17'E; alt. 960 m; 24 Jul. 2011 • J.L.P. Louis 13439 (K); Isangi, Bassao (île), Un peu en aval de Loléko; 0°54'N, 24°12'E; 27 Jan. 1939 • J.L.P. Louis 14540 (BM, K, MO, P); Isangi, Yangambi; 0°46'N, 24°27'E; alt. 470 m; 09 Sep. 1939 • J.L.P. Louis 8357 (BR, MO, P); Isangi, Yangambi; 0°46'N, 24°27'E; alt. 470 m; 08 Mar. 1938. Gabon – Estuaire • M.E. Leal 268 (LBV, WAG), Monts de Cristal, Mbe National Parc, south of Mont Mbilan; 0°27'40'N, 10°15'30'E; alt. 550 m; 12 Feb. 2005 – Ngounié • M.E. Leal 1906 (LBV, MO, WAG), Bouvala hills. Col between the two summits; 1°37'15'S, 11°45'45'E; alt. 995 m; 06 Oct. 2007 – Ogooué-Ivindo • A.H. Gentry 33052 (MO, P), Makokou. Transect 1; 0°34'N, 12°52'E; alt. 480 m; 01 Jul. 1981 • J. Florence 1158 (P), Station d'Ipassa, 10 km S de Makokou; 0°30'N, 12°47'E; alt. 500 m; 04 May. 1978 • N. Hallé 3474 (K, P, P, U), Bélinga. Route de Mvadi; 1°05'N, 13°08'E; alt. 800 m; 12 Dec. 1964 – Woleu-Ntem • MINKébé Series R67 (WAG), Minkébé area, 10 x 10 m inventory plot R, placed 2–12 m south at 1380–1390 m on transect A; 1°30'N, 12°48'E; Apr. 1990 • MINKébé Series W289 (WAG), crest to the south of Mount Minkébé; 1°35'N, 12°52'E; 10 May. 1990 • T.L.P. Couvreur 856 (LBV, WAG, YA), Oyem, on road to Mbolonzok (off main road to Mongono and Equatorial Guinea); 1°29'21.34'N, 11°27'58.98'E; alt. 675 m; 13 Nov. 2015 – Unknown major area • N. Hallé 2975 (U). Republic of the Congo – Sangha • S.T. Ndolo Ebika 294 (E, IEC, WAG), Nouablé-Ndoki National Park, Goualougo Study Site, 37.53 km E-SE de Bomassa; 2°11'21.34'N, 16°31'24.35'E; alt. 383 m; 18 Jan. 2008 • S.T. Ndolo Ebika 659 (E, IEC, WAG), Périphérie du Nouable Ndoki National Park: 19 Km E de Bomassa; 2°12'23.29'N, 16°20'19.93'E; alt. 381 m; 06 Jun. 2011.

#### ﻿ Uvariopsis submontana

Taxon classificationPlantaeMagnolialesAnnonaceae

Kenfack, Gosline & Gereau, Novon 13(4): 444 (2003)

**Figure 82.**
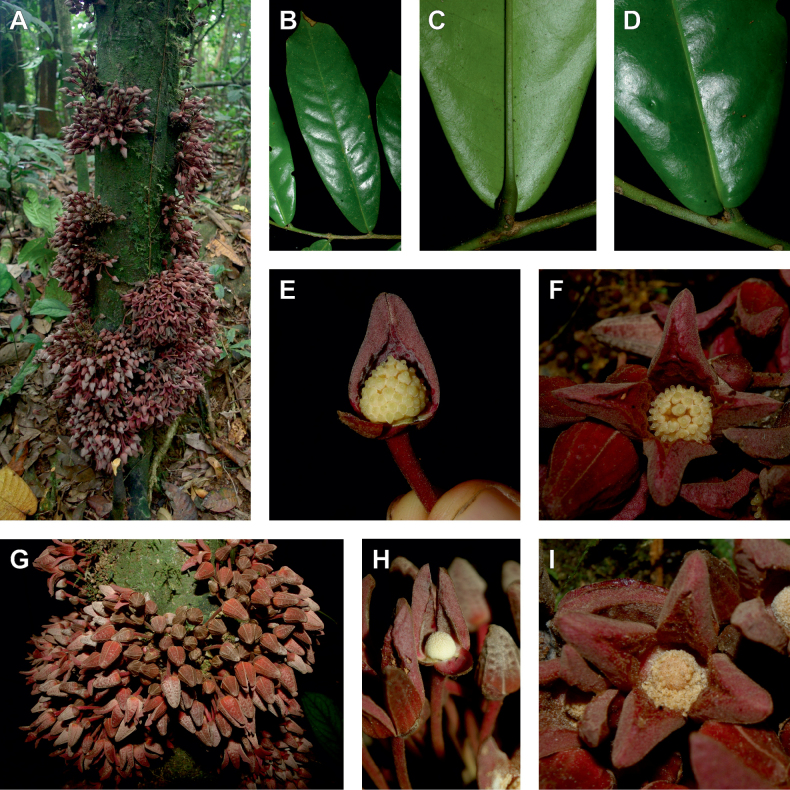
*Uvariopsissubmontana* Kenfack, Gosline & Gereau **A** trunk with clumps of inflorescences **B** leaf, upper side **C** leaf base lower side **D** leaf base, upper side **E** female flower, two petals removed, side view **F** flower, top view **G** clumps of inflorescences on the trunk **H** male flower, two petals removed, side view **I** male flower, top view. **A–I** Couvreur 627. Photos Thomas Couvreur.

**Figure 83.**
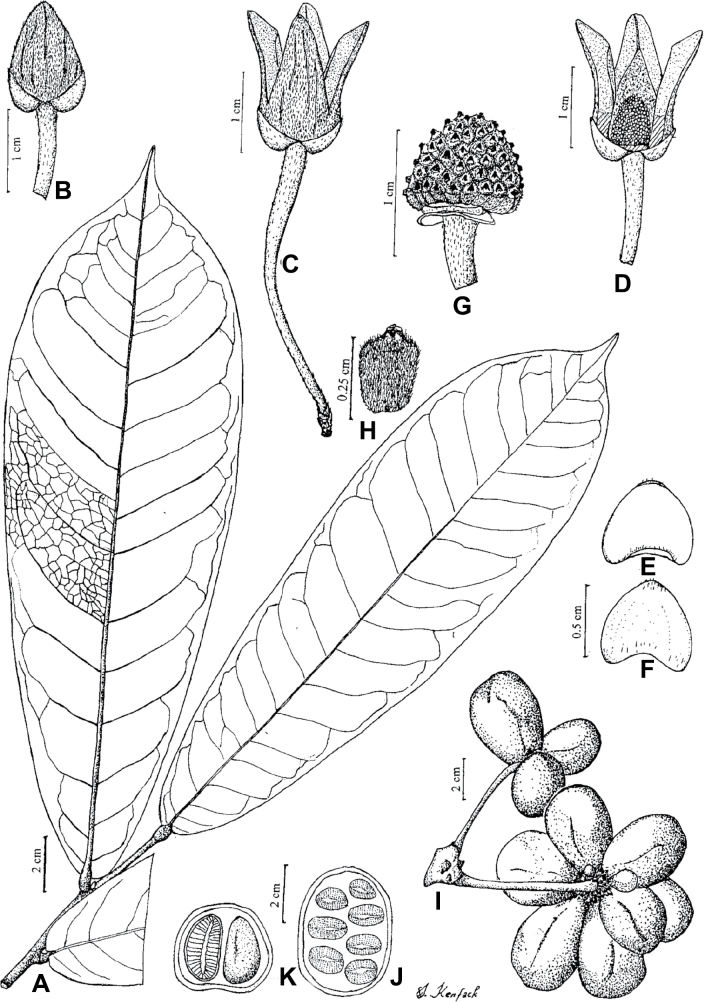
*Uvariopsissubmontana* Kenfack, Gosline & Gereau **A** leaves **B** female flower bud, side view **C** male flower, side view **D** male flower, one petal removed, side view **E** inner surface of sepal **F** outer surface of sepal **G** female flower, all four petals removed, side view **H** carpel, lateral view **I** infructescence **J** longitudinal section of monocarp **K** transversal section of monocarp. **A–H** from Kenfack 1334 (type) **I–K** from Kenfack 1373. Drawings by David Kenfack, from “The Genus *Uvariopsis* (Annonaceae) in Tropical Africa, with a Recombination and One New Species from Cameroon”, Novon 13:4, Figure 1, p. 445 (Kenfack et al. 2003), with permission from the Missouri Botanical Garden Press, St. Louis.

**Figure 84.**
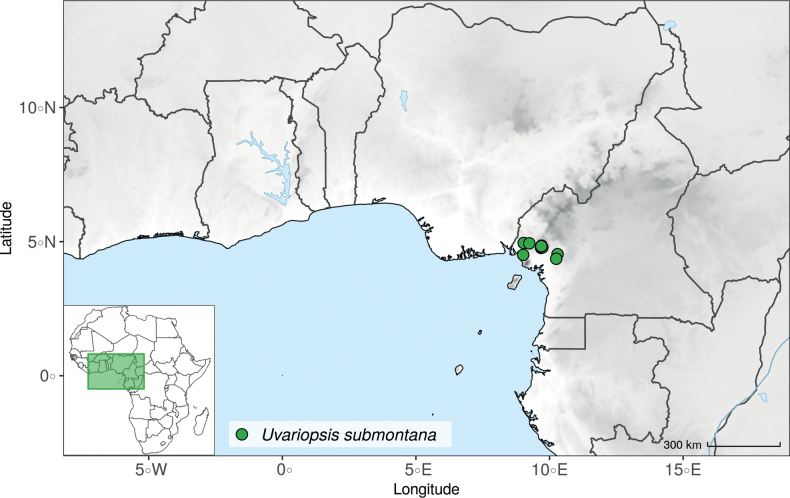
Distribution map of *Uvariopsissubmontana*. Shades of grey represent elevation, from white (sea level) to darker grey (higher elevation). The inset shows the extent of the map over Africa.

Figs 82 , 83 , 84 ; Table 5

##### Type.

Cameroon – South-West Region • D. Kenfack 1334 (holotype: YA; isotypes: K! (K000683145), MO, SCA), Rumpi Hills; 4°57'N, 9°02'E; alt. 800 m; 06 Feb. 2000.

##### Description.

Tree 4.5–25 m tall, D.B.H 5–30 cm; young branches sparsely pubescent to glabrous, old branches glabrous. Petiole 3–8 mm long, 2.5–4 mm wide, sparsely pubescent to glabrous. Leaf lamina 160–380 mm long, 50–110 mm wide, length:width ratio 2.7–4.3, obovate, coriaceous, base rounded to subcordate, apex attenuate to acuminate, acumen 8–30 mm long, surface above glabrous, surface below glabrous; midrib impressed above, raised below, glabrous above, glabrous below; secondary veins 9–25 pairs, brochidodromous to weakly brochidodromous, impressed above, raised below; tertiary veins reticulate. Flowers unisexual, male and female flowers slightly dimorphic, on same individuals (plant monoecious). Flower buds ovoid to conical. Male inflorescences borne in dense clumps on thickenings at base of the trunk, sparser above 2 m., above the clumps of female inflorescences, mainly between the base and the lower 5 m of the trunk, composed of 6 to 50 flowers. Peduncle inconspicuous. Flower pedicel 25–50 mm long, ca. 1 mm in diameter, pubescent to sparsely pubescent. Bracts 2 to 4 at base, upper bract 1–1.6 mm long, 1–1.6 mm wide, broadly ovate, pubescent outside, glabrous inside. Sepals 2, 5–11 mm long, 6–12 mm wide, broadly ovate, pubescent to sparsely pubescent outside, glabrous inside, dark red. Petals 4, 8–19 mm long, 4–8 mm wide, length:width ratio 1.8–4.7, ovate to narrowly ovate, free to fused at base over 30% of their length, pubescent outside, glabrous inside, pinkish to dark red outside. Stamens 100 to 200, 0.4–0.7 mm long, 0.3–0.4 mm wide, anthers linear, connective prolongation truncate. Female inflorescences borne in dense clumps on thickenings at base of the trunk, below the clumps of male inflorescences, composed of 6 to 50 flowers. Flower pedicel 24–60 mm long, ca. 1 mm in diameter, pubescent to sparsely pubescent. Bracts 2 to 4 at base, upper bract 1–1.6 mm long, 1–1.6 mm wide, broadly ovate, pubescent outside, glabrous inside. Sepals 2, 6–8 mm long, 6–9 mm wide, broadly ovate, pubescent to sparsely pubescent outside, glabrous inside, dark red. Petals 4, 15–17 mm long, 5–9 mm wide, length:width ratio 1.8–4.7, ovate to narrowly ovate, free to fused over 30% of their length, pubescent outside, glabrous inside, pinkish to dark red outside. Carpels 50 to 100, 1.5–3.5 mm long, 0.8–1.5 mm wide, pubescent, free; stigma ca. 0.5 mm long, ca. 0.2 mm wide, globular, glabrous. Fruiting pedicel 25–90 mm long, 3–7 mm in diameter, glabrous. Monocarps, 3–25, 17–80 mm long, 13–55 mm wide, length:width ratio, ovoid to cylindrical, constricted between seeds in dried specimens, sparsely pubescent to glabrous, pale green ripening dark yellow, sessile. Seeds 6–12 per monocarp, biseriate, 18–25 mm long, 8–13 mm wide, ellipsoid.

##### Distribution.

Endemic to Lower Guinean Domain of the Guineo-Congolian Region. Only known from the South-West and Littoral Regions in Cameroon, mainly in Mont Koupé and Ebo National Park.

##### Habitat and ecology.

Submontane mature or old secondary rain forests. Soil: rocky, humic combisol. Altitude: 900–1300 m asl.

##### Phenology.

Flowers collected from January to May and in December.

##### Vernacular names.

Cameroon: ‘Michile’ in Bakossi (Cheek 7131).

##### Notes.

This species resembles *Up.bakeriana*, *Up.citrata* and *Up.korupensis* in having large obovate leaves (up to 38 cm long), with rounded to cordate bases. It differs from *Up.bakeriana* and *Up.citrata* in having longer flower pedicel (24–60 mm vs. 0–8 mm). It is very similar to *Up.korupensis*, but has generally smaller leaves (16–38 cm vs. 28–62 cm in *Up.korupensis*). The female flowers of *Up.submontana* have longer sepals (6–8 mm long vs. 3–5 mm long) and smaller petals (15–17 mm long vs. 15–35 mm long) than *Up.korupensis*, which results in petals less than 3 times longer than the sepals (vs. petals more than 3 times longer than the sepals). Petals of *Up.submontana* are pinkish to dark red inside and outside whereas petals of *Up.korupensis* are cream to brownish outside and cream to pinkish inside (Table 5).

##### Conservation status.

This species is endemic to Cameroon and has been evaluated as Endangered EN B1ab(iii)+2ab(iii) and mainly threatened by the expansion of agriculture (Cheek 2014b).

##### Additional specimens examined.

Cameroon – Littoral • D. Kenfack 1602 (MO), Nkam, Yingui, Bataba. PE80; 4°32'N, 10°18'E; alt. 780 m; 20 Feb. 2002 • T.L.P. Couvreur 627 (MPU, YA), Ebo Wildlife Reserve, Djuma permanent camp. On east trail; 4°21'39.35'N, 10°15'07.7'E; alt. 957 m; 15 Feb. 2013 – South-West Region • J.F. Villiers 2490 (P), 6 km WNW Bomana, 34 km NW Limbé; 4°30'24.99'N, 9°00'50.26'E; 15 Dec. 1984 • M.R. Cheek 7034 (K, WAG, YA), Mount Kupe. Kupe village. Esense river near farm of Philip Taza; 4°46'N, 9°41'E; alt. 1000 m; 19 Jan. 1995 • M.R. Cheek 7131 (K, WAG), Kupe village. Hunters path from Kupe village to the mountain top, running to S and parallel with Esense river (now dry). Towards village largely coffee farms with shade trees. Grid ref. from Magellan reading where path enters forest; 4°47'N, 9°41'E; alt. 840 m; 24 Jan. 1995 • P. Lane 490 (K, SCA, WAG, YA), Mount Kupe. Ndum. Forest trail 2 km S from Etube-Tape village; 4°51'N, 9°42'E; alt. 1100 m; 01 Feb. 1995 • S. Cable 1221 (K, MO, SCA, WAG, YA), Mt Kupe, Nyasoso, Shrike trail; 4°49'00.12'N, 9°42'E; alt. 1100 m; 08 Feb. 1995 • S. Cable 2736 (K, MO, P, SCA, WAG, YA), Kupe-Muanenguba Division. Path on to ridge between Daniel's saprophyte site and Kupe rock; 4°47'N, 9°44'E; alt. 1000 m; 30 May. 1996 • T.L.P. Couvreur 1059 (MPU, WAG, YA), Nyasoso village, on max's trail to Mt Kupe; 4°49'47.02'N, 9°41'34.79'E; alt. 1024 m; 05 Apr. 2016 • T.L.P. Couvreur 965 (MPU, WAG, YA), Rumpi mountains, forest trail, ca. 5 km after Dikome Balue village, ca. 40 km north of Kumba; 4°56'10.37'N, 9°14'30.34"E; alt. 1418 m; 10 Jan. 2016.

#### ﻿ Uvariopsis zenkeri

Taxon classificationPlantaeMagnolialesAnnonaceae

Engl., Notizbl. Königl. Bot. Gart. Berlin 2: 298 (1899)

**Figure 85.**
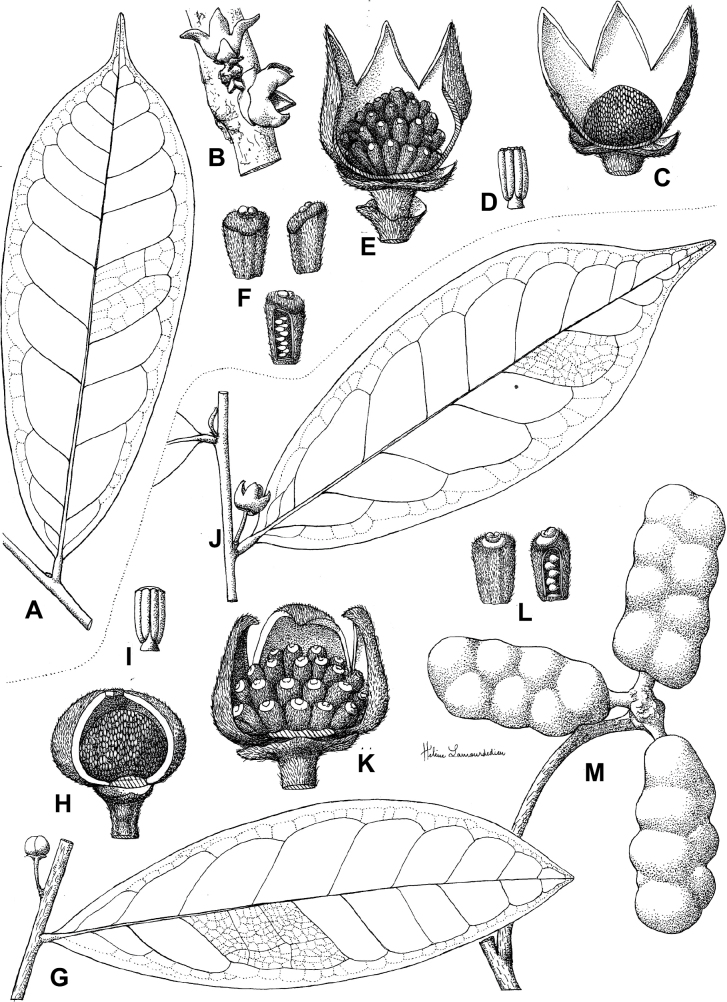
*Uvariopsiszenkeri* Engl **A** leaf, upper side **B** male flowers on brnch **C** male flower, one petal removed **D** stamen **E** female flower, one petal removed **F** carpel, front and side view, detail of ovules. *Uvariopsiscongensis* Robyns & Ghesq. **G** flowering branch with male flower bud **H** male flower, one petal removed **I** stamen **J** flowering branch with female flower **K** female flower, one petal removed **L** carpel, side view and detail of ovules **M** fruit **A–E** from Zenker 1117 **F** from Zenker 63 **G–L** from Tisserant 1363 **M** from Letouzey 5494. Drawings by Hélène Lamourdedieu, Publications Scientifiques du Muséum national d’Histoire naturelle, Paris.

**Figure 86.**
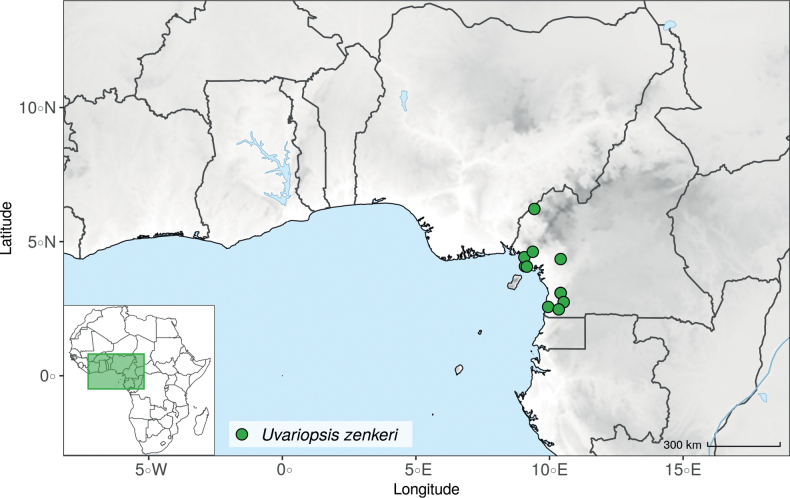
Distribution map of *Uvariopsiszenkeri*. Shades of grey represent elevation, from white (sea level) to darker grey (higher elevation). The inset shows the extent of the map over Africa.

Figs 85A–F , 86 ; Table 6

##### Type.

Cameroon – South Region • G.A. Zenker 1117 (holotype: B! (B 10 0153124); isotypes: BM! (BM000554075), G! (G00420324), GOET! (GOET005734), HBG! (HBG502515), K! (K000199042), LE! (LE00012462), M! (M0107936), P! (P00362604), S! (S07-11029), WU! (WU0025790)), Bipinde, Urwaldgebiet; 3°05'N, 10°25'E; 1896.

##### Description.

Shrub to tree 2–5 m tall, D.B.H 1.5–2 cm; young branches densely pubescent to pubescent, old branches glabrous. Petiole 2–5.5 mm long, 1–1.5 mm wide, pubescent to glabrous. Leaf lamina 110–158 mm long, 36–58 mm wide, length:width ratio 2.1–3.4, elliptic to obovate, papyraceous to coriaceous, base acute to decurrent, apex acuminate, acumen 10–24 mm long, surface above glabrous, surface below puberulent to glabrous when young, glabrous when old; midrib impressed above, raised below, glabrous above, pubescent to glabrous below; secondary veins 6–13 pairs, brochidodromous, impressed above, raised below; tertiary veins reticulate. Flowers unisexual, male and female flowers similar, on same individuals (plant monoecious). Flower buds globose to subovoid. Male and female inflorescences borne on trunk, old branches or old axillary, composed of 1, (sub)sessile flower. Peduncle inconspicuous. Flower pedicel 0–7 mm long, 1–1.5 mm in diameter, pubescent. Bracts 1 to 2 (5 in bud) at base, upper bract 1–4 mm long, 2–5 mm wide, ovate, pubescent outside, glabrous inside. Sepals 2, 3–7 mm long, 4.5–7 mm wide, broadly ovate, basally fused, pubescent outside, glabrous inside, brown. Petals 4, 6–13 mm long, 4–7 mm wide, length:width ratio 1.5–2.2, ovate to triangular, fused at base over ca. 50 % of their length, pubescent outside, glabrous inside, brown outside. Male flowers: stamens 100 to 150, ca. 0.5 mm long, ca. 0.3 mm wide, anthers linear, connective prolongation truncate or absent. Female flowers: carpels 13 to 22, 1.5–3 mm long, 1–1.5 mm wide, velutinous, free; stigma 0.1–0.5 mm long, ca. 1 mm wide, coiled, glabrous. Fruiting pedicel 2–10 mm long, ca. 2.5 mm in diameter, pubescent. Monocarps, 1–3, ca. 37 mm long, ca. 17 mm wide, length:width ratio ca. 2.2, cylindrical, slightly veined and constricted between the seed when dried, tomentose, brown, subsessile; stipe ca. 1 mm long, ca. 2 mm wide, tomentose. Seeds 10–12 per monocarp, biseriate, ca. 13 mm long, ca. 7 mm wide, ellipsoid.

##### Distribution.

Endemic to Lower Guinean Domain of the Guineo-Congolian Region: Cameroon.

##### Habitat and ecology.

Lowland mature or secondary rain forests. Soil: rocky, humic, volcanic. Altitude: 0–m asl.

##### Phenology.

Flowers and fruits collected in April.

##### Notes.

This species resembles *Up.bisexualis*, *Up.congensis* and *Up.oligocarpa* in having elliptic leaves generally less than 16 cm long with decurrent base. It differs from these species in having densely pubescent to pubescent young branches (vs. pubescent to glabrous). It also differs from *Up.bisexualis* with its unisexual flowers (vs. bisexual flowers). *Up.zenkeri* has less carpels (13 to 22 vs. 20 to 40 in *Up.congensis*), which results in generally 1 to 3 monocarps reaching maturity on the ripe fruit (vs. up to 15 mature monocarps in *Up.congensis*). The monocarps of *Up.zenkeri* are tomentose (vs. glabrate to glabrous in *Up.congensis* and pubescent to sparsely pubescent in *Up.oligocarpa*), slightly constricted between the seeds (vs. not constricted between the seeds in *Up.oligocarpa*) (Table 6).

##### Conservation status.

This species is endemic to Cameroon. It occurs in nine locations, only four of which are situated in protected areas (Campo-Ma’an National Park, Ebo National Park, Mont Cameroun National Park, Takamanda National Park). It has been previously assessed as Vulnerable VU under criteria B2ab(i,ii,iii,iv,v) (Texier and Stévart 2021).

##### Additional specimens examined.

Cameroon – South Region • G.A. Zenker 3045 (B, BM, G, K, L, M, MA, WAG), Bipindi; 3°05'N, 10°25'E; 1904 • G.A. Zenker 3228 (G, L, M), Bipinde, Urwaldgebiet; 3°05'N, 10°25'E; 1904 • G.A. Zenker 515 (B, G, G, M, MA, MO, WAG), Bipindi; 3°05'N, 10°25'E; Mar. 1914 • G.P. Tchouto Mbatchou 2846 (KRIBI, WAG), Efoulan, Ongongo and Nkolomekok hills in Akom II area; 2°44'43'N, 10°31'48'E; alt. 1000 m; 25 Apr. 2000 • G.P. Tchouto Mbatchou 3252 (KRIBI, SCA, WAG), Campo-Ma'an area, Massif des Mamelles, Mamelles highlands; 2°33'57'N, 9°56'58'E; alt. 280 m; 23 Apr. 2001 • T.L.P. Couvreur 707 (MPU, WAG, YA), Campo Ma'an National Park, 11 km on trail from Ebinanemeyong village, on road, 7 km from Nyabessan to Campo town; 2°28'24.59'N, 10°20'41.79'E; 14 Feb. 2015 – South-West Region • D.W. Thomas 3455 (B, K, LBV, MO, WAG), forested lower slope of Mount Cameroon, above Batoke; 4°05'N, 9°05'E; alt. 300 m; 24 Apr. 1984 • D.W. Thomas 7372 (MO, P, WAG), Takamanda Forest Reserve; 6°13'N, 9°26'E; alt. 500 m; 30 Apr. 1987 • S.N. Ekema 944 (K, SCA); Ndian, Dikome, Mokoko Forest Reserve; 4°25'11.64'N, 9°03'29.16'E; alt. 120 m; 05 May. 1994 • T.L.P. Couvreur 1027 (MPU, WAG, YA), on trail leading to top of Mt Etinde, after Ekonjo village; 4°04'02.29'N, 9°09'11.15'E; alt. 756 m; 01 Apr. 2016 • T.L.P. Couvreur 978 (WAG, YA), on top of hill, near Small Ekombe village, 3 km after Kumba on road to Ekondo Titi town; 4°37'21.52'N, 9°22'26.62'E; alt. 589 m; 13 Jan. 2016 – Unknown major area • J. Osborne 200 (K, WAG), Littoral Province, Ebo Forest proposed National Park. Ebo Forest Research Station-Bekob drinking stream trail; 4°21'N, 10°25'E; 27 Oct. 2006.

#### ﻿ Uvariopsis

Taxon classificationPlantaeMagnolialesAnnonaceae

sp. nov. 1 ‘Uganda’

Fig. 87

##### Description.

Tree ca. 3 m tall, D.B.H unknown; young branches glabrous, old branches glabrous. Petiole ca. 6 mm long, ca. 1.7 mm wide, glabrous. Leaf lamina ca. 160 mm long, ca. 60 mm wide, length:width ratio ca. 2.6, elliptic, coriaceous, base acute to decurrent, apex attenuate, surface above glabrous, surface below glabrous; midrib impressed above, raised below, glabrous above, glabrous below; secondary veins ca. 12 pairs, brochidodromous to weakly brochidodromous, impressed above, raised below; tertiary veins reticulate. Flowers unknown. Fruits unknown.

**Figure 87.**
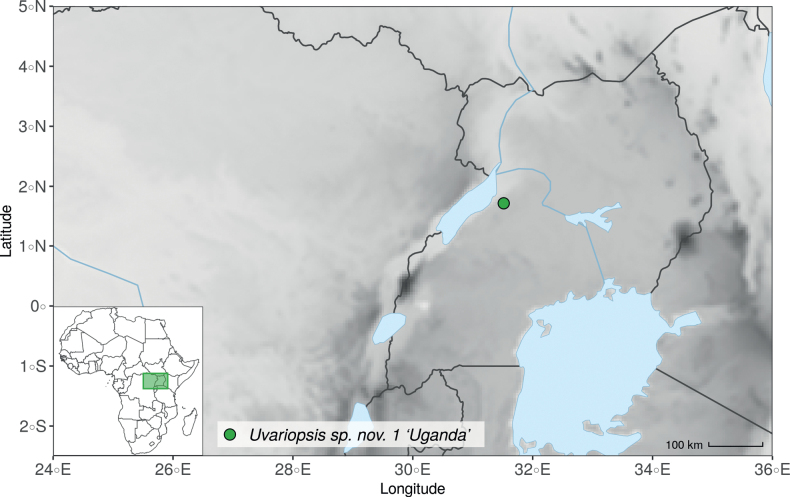
Distribution map of *Uvariopsis* sp. nov. 1 ‘Uganda’. Shades of grey represent elevation, from white (sea level) to darker grey (higher elevation). The inset shows the extent of the map over Africa.

##### Distribution.

Endemic to Zambezian Region. Known from only one locality in Uganda: the Budongo Forest Reserve.

##### Habitat and ecology.

Rain forests. Altitude ca. 1051 m asl.

##### Phenology.

Unknown.

##### Notes.

This species resembles *Up.congensis* in having elliptic leaves with acute to decurrent base and attenuate apex, but it differs in having longer petioles (ca. 6 mm vs. 2–5 mm). Molecular analysis retrieves this species as sister species of the clade formed by *Up.congensis* and *Up.lovettiana*. Further collection of this species is needed to provide a more complete description (particularly for the flowers and fruits). This species is known from a single specimen from the Budongo Forest Reserve. It is thus likely to be Critically Endangered CR. However, further exploration of the region is necessary to know whether this species occurs in other localities.

##### Specimen examined.

Uganda – Western Province • ATBP (Africa Tropical Biodiversity Programme) 666 (MO); Masindi District, Budongo Forest Reserve, Nyakafunjo Nature Reserve; 1°43'N, 31°31'E; alt. 1051 m; 26 Jun. 1998.

#### ﻿ Uvariopsis

Taxon classificationPlantaeMagnolialesAnnonaceae

sp. nov. 2 ‘Tanzania’

Fig. 88

##### Description.

Shrub to tree 4–6 m tall, D.B.H unknown; young branches glabrous, old branches glabrous. Petiole ca. 2 mm long, ca. 2 mm wide, glabrous. Leaf lamina 150–180 mm long, ca. 60 mm wide, length:width ratio ca. 3, elliptic to obovate, coriaceous, base rounded, apex attenuate, surface above glabrous, surface below glabrous; midrib impressed above, raised below, glabrous above, glabrous below; secondary veins ca. 13 pairs, brochidodromous to weakly brochidodromous, impressed above, raised below; tertiary veins reticulate. Flowers not seen (label on specimen Kyoto University Expedition 1039 says 5 petals). Fruiting pedicel ca. 51 mm long, ca. 1 mm in diameter, pubescent. Monocarps, 1–2, ca. 30 mm long, ca. 15 mm wide, length:width ratio ca. 2, cylindrical to obpyriform, smooth, slightly constricted between the seeds, pubescent, subsessile; stipe 0–1 mm long, ca. 1 mm wide, pubescent.

**Figure 88.**
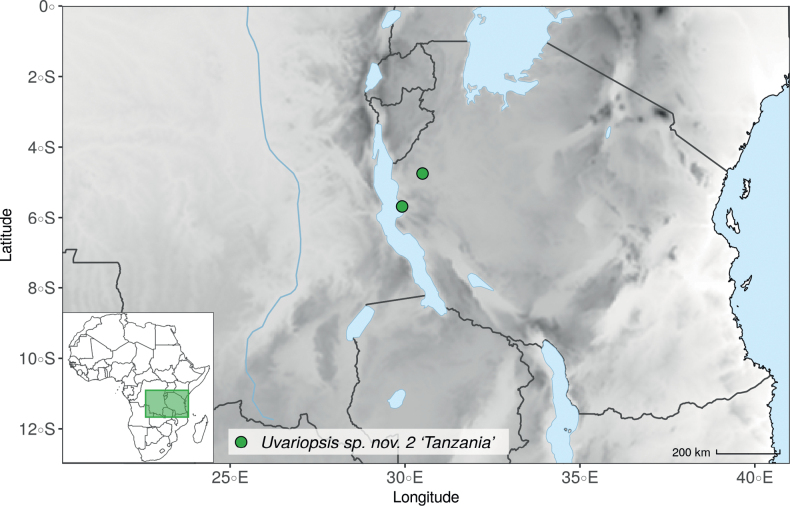
Distribution map of *Uvariopsis* sp. nov. 2 ‘Tanzania’. Shades of grey represent elevation, from white (sea level) to darker grey (higher elevation). The inset shows the extent of the map over Africa.

##### Distribution.

Endemic to Somalia-Masai Region. Only known from the Kigoma region in eastern Tanzania.

##### Habitat and ecology.

Rain forests. Altitude ca. 900 m asl.

##### Phenology.

Fruits collected in December (flowers said to be collected too, but remained not found).

##### Vernacular names.

Tanzania: ‘Ntwelele’ in Tongwe (Kyoto University Expedition 1039).

##### Notes.

This species is very similar to *Up.lovettiana* having similar elliptic leaf shape, but differs by the leaf base being rounded (vs. acute to decurrent in *Up.lovettiana*) and in having pubescent monocarps slightly constricted between the seeds (vs. sparsely pubescent to glabrous monocarps, strongly constricted between the seeds). The monocarps are also subsessile (stipe 0–1 mm long, vs. stipitate, stipe 2.5–14 mm long in *Up.lovettiana*). Confirming the status of new species with molecular analysis is however essential in order to make sure these specimens are not a morphological variation of *Up.lovettiana*.

##### Specimens examined.

Tanzania – Kigoma • G.S. Bidgood 2783 (DSM, K, P, WAG); Kigoma Rural District, T4. Kigoma Distr.: Kasye Forest; 5°41'S, 29°55'E; alt. 900 m; 18 Mar. 1994 – Kyoto University Expedition 1039 (EA); Kigoma Rural District; 4°45'S, 30°30'E; 24 Dec. 1963.

#### ﻿ Uvariopsis

Taxon classificationPlantaeMagnolialesAnnonaceae

sp. nov. 3 ‘Congo’

Fig. 89

##### Description.

Tree ca. 3.5 m tall, D.B.H unknown; young branches glabrous, old branches glabrous. Petiole ca. 2 mm long, ca. 2 mm wide, glabrous. Leaf lamina ca. 160 mm long, ca. 58 mm wide, length:width ratio ca. 2.8, elliptic, coriaceous, base rounded, apex attenuate, surface above glabrous, surface below glabrous; midrib impressed above, raised below, glabrous above, glabrous below; secondary veins ca. 9 pairs, brochidodromous, impressed above, raised below; tertiary veins reticulate. Flowers only flower bud seen. Flower buds ovoid to conical. Inflorescences axillary, composed of 1 flower. Peduncle inconspicuous. Flower pedicel 20–65 mm long, ca. 1 mm in diameter, glabrate to glabrous. Bracts 1 in the lower half of the pedicel, ca. 1 mm long, ca. 1 mm wide, broadly ovate, sparsely pubescent outside, glabrous inside. Sepals 2, ca. 2 mm long, ca. 3 mm wide. Petals 4, ca. 7 mm long, ca. 4 mm wide, length:width ratio red outside. Fruits unknown.

**Figure 89.**
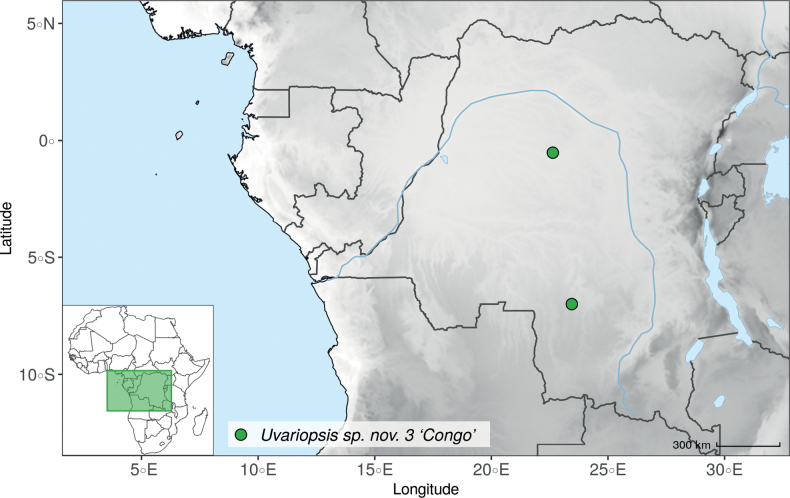
Distribution map of *Uvariopsis* sp. nov. 3 ‘Congo’. Shades of grey represent elevation, from white (sea level) to darker grey (higher elevation). The inset shows the extent of the map over Africa.

##### Distribution.

Endemic to Congolia Domain of the Guineo-Congolian Region. Only known from the Kasai-Oriental and Equateur regions in the Democratic Republic of the Congo.

##### Habitat and ecology.

Rain forests.

##### Phenology.

Flowers collected in May and October.

##### Notes.

This species resembles *Up.solheidii* and *Up.noldeae* in having elliptic leaves with rounded leaf base. It differs from *Up.solheidii* in having young branches and petioles completely glabrous (vs. tomentose in *Up.solheidii*). It differs from *Up.noldeae* in having flowers borne on young branches and axillary (vs. borne on trunk in *Up.noldeae*, but see the discussion above about the unreliability of the flower position to discriminate the species), and in having flower pedicels 20–65 mm long (vs. 70–83 mm long in *Up.noldeae*). This species is known from only two specimens from two different localities distant of more than 700 km from each other in the Congo bassin. It is thus likely to occur in other localities but further exploration of the region is necessary to confirm that.

##### Specimens examined.

Democratic Republic of the Congo – Equateur • C.M. Evrard 6276 (K); Ikela, Yalifake; 0°31'06'N, 22°38'12'E; 06 May. 1959 – Kasai-Oriental • L. Liben 3854 (K); Mwene Ditu, Mwene Ditu, Mwene-Ditu; 7°00'S, 23°27'E; 24 Oct. 1957.

## Supplementary Material

XML Treatment for ﻿Ophrypetaleae

XML Treatment for Dennettia

XML Treatment for Uvariodendron

XML Treatment for Uvariodendron angustifolium

XML Treatment for Uvariodendron anisatum

XML Treatment for Uvariodendron calophyllum

XML Treatment for Uvariodendron citriodorum

XML Treatment for Uvariodendron connivens

XML Treatment for Uvariodendron dzomboense

XML Treatment for Uvariodendron fuscum

XML Treatment for Uvariodendron fuscum var. fuscum

XML Treatment for Uvariodendron fuscum var. giganteum

XML Treatment for Uvariodendron fuscum var. magnificum

XML Treatment for Uvariodendron gorgonis

XML Treatment for Uvariodendron kimbozaense

XML Treatment for Uvariodendron kirkii

XML Treatment for Uvariodendron mbagoi

XML Treatment for Uvariodendron molundense

XML Treatment for Uvariodendron mossambicense

XML Treatment for Uvariodendron occidentale

XML Treatment for Uvariodendron pilosicarpum

XML Treatment for Uvariodendron pycnophyllum

XML Treatment for Uvariodendron schmidtii

XML Treatment for Uvariodendron usambarense

XML Treatment for Uvariopsis

XML Treatment for Uvariopsis bakeriana

XML Treatment for Uvariopsis bisexualis

XML Treatment for Uvariopsis citrata

XML Treatment for Uvariopsis congensis

XML Treatment for Uvariopsis congensis var. angustifolia

XML Treatment for Uvariopsis congensis var. congensis

XML Treatment for Uvariopsis congolana

XML Treatment for Uvariopsis dicaprio

XML Treatment for Uvariopsis dioica

XML Treatment for Uvariopsis etugeana

XML Treatment for Uvariopsis guineensis

XML Treatment for Uvariopsis guineensis var. globiflora

XML Treatment for Uvariopsis guineensis var. guineensis

XML Treatment for Uvariopsis korupensis

XML Treatment for Uvariopsis lovettiana

XML Treatment for Uvariopsis noldeae

XML Treatment for Uvariopsis oligocarpa

XML Treatment for Uvariopsis pedunculosa

XML Treatment for Uvariopsis solheidii

XML Treatment for Uvariopsis solheidii var. letestui

XML Treatment for Uvariopsis solheidii var. solheidii

XML Treatment for Uvariopsis submontana

XML Treatment for Uvariopsis zenkeri

XML Treatment for Uvariopsis

XML Treatment for Uvariopsis

XML Treatment for Uvariopsis

## ﻿Additional information

### Author ORCIDs

Léo-Paul M. J. Dagallier https://orcid.org/0000-0002-3270-1544

Marie Couderc https://orcid.org/0000-0001-7297-155X

Aurélie Grall https://orcid.org/0000-0002-1613-4703

Jan J. Wieringa https://orcid.org/0000-0003-0566-372X

Bonaventure Sonké https://orcid.org/0000-0002-4310-3603

Thomas L. P. Couvreur https://orcid.org/0000-0002-8509-6587

### Data availability

All of the data that support the findings of this study are available in the main text, in the Supplementary Information and are deposited at GBIF, the Global Biodiversity Information Facility, available at https://doi.org/10.15468/zdvvkh.

## Supplementary materials

Supplementary material 1 Phylogenetic tree of the Monodoreae inferred with ASTRAL, based on 334 nuclear genes trees Data type pdf Explanation note The branch support is given as pie charts of the quartet support (QS) such as the black proportion represents the percentage of quartets in the gene trees agreeing with the branch in the species tree. The light blue box encloses the Monodoreae tribe. The name of the specimens is composed of a unique identifier, followed by the species (or variety/subsepecies) name, followed by the 3 first letters of the name of the collector and the collection number. For the details on the specimens see Suppl. material 3. https://binary.pensoft.net/file/909949This dataset is made available under the Open Database License (http://opendatacommons.org/licenses/odbl/1.0/). The Open Database License (ODbL) is a license agreement intended to allow users to freely share, modify, and use this Dataset while maintaining this same freedom for others, provided that the original source and author(s) are credited.Léo-Paul M. J. Dagallier, Frank M. Mbago, Marie Couderc, Myriam Gaudeul, Aurélie Grall, Caroline Loup, Jan J. Wieringa, Bonaventure Sonké, Thomas L. P. Couvreur

Supplementary material 2 RAxML maximum likelihood tree of the Monodoreae, based on a concatenated supermatrix of 334 nuclear genes. Bootstrap support values (in %) are given at the nodes Data type pdf Explanation note The light blue box encloses the Monodoreae tribe. The name of the specimens is composed of a unique identifier, followed by the species (or variety/subsepecies) name, followed by the 3 first letters of the name of the collector and the collection number. The identification of the specimens follow the taxonomic changes treated in this study. For the details on the specimens see Suppl. material 3. https://binary.pensoft.net/file/909950This dataset is made available under the Open Database License (http://opendatacommons.org/licenses/odbl/1.0/). The Open Database License (ODbL) is a license agreement intended to allow users to freely share, modify, and use this Dataset while maintaining this same freedom for others, provided that the original source and author(s) are credited.Léo-Paul M. J. Dagallier, Frank M. Mbago, Marie Couderc, Myriam Gaudeul, Aurélie Grall, Caroline Loup, Jan J. Wieringa, Bonaventure Sonké, Thomas L. P. Couvreur

Supplementary material 3 Details of the vouchers used in this study. Data type ods https://binary.pensoft.net/file/909951This dataset is made available under the Open Database License (http://opendatacommons.org/licenses/odbl/1.0/). The Open Database License (ODbL) is a license agreement intended to allow users to freely share, modify, and use this Dataset while maintaining this same freedom for others, provided that the original source and author(s) are credited.Léo-Paul M. J. Dagallier, Frank M. Mbago, Marie Couderc, Myriam Gaudeul, Aurélie Grall, Caroline Loup, Jan J. Wieringa, Bonaventure Sonké, Thomas L. P. Couvreur

Supplementary material 4 Details of the specimens examined in this study. Data type tsv https://binary.pensoft.net/file/909952This dataset is made available under the Open Database License (http://opendatacommons.org/licenses/odbl/1.0/). The Open Database License (ODbL) is a license agreement intended to allow users to freely share, modify, and use this Dataset while maintaining this same freedom for others, provided that the original source and author(s) are credited.Léo-Paul M. J. Dagallier, Frank M. Mbago, Marie Couderc, Myriam Gaudeul, Aurélie Grall, Caroline Loup, Jan J. Wieringa, Bonaventure Sonké, Thomas L. P. Couvreur
