# Supplementary figures and images for: ﻿Phylogenomic inference of the African tribe Monodoreae (Annonaceae) and taxonomic revision of Dennettia, Uvariodendron and Uvariopsis
Source: PhytoKeys. 2023 Sep 22;233:1–200. doi: 10.3897/phytokeys.233.103096 (PMC10552675; doi:10.3897/phytokeys.233.103096)

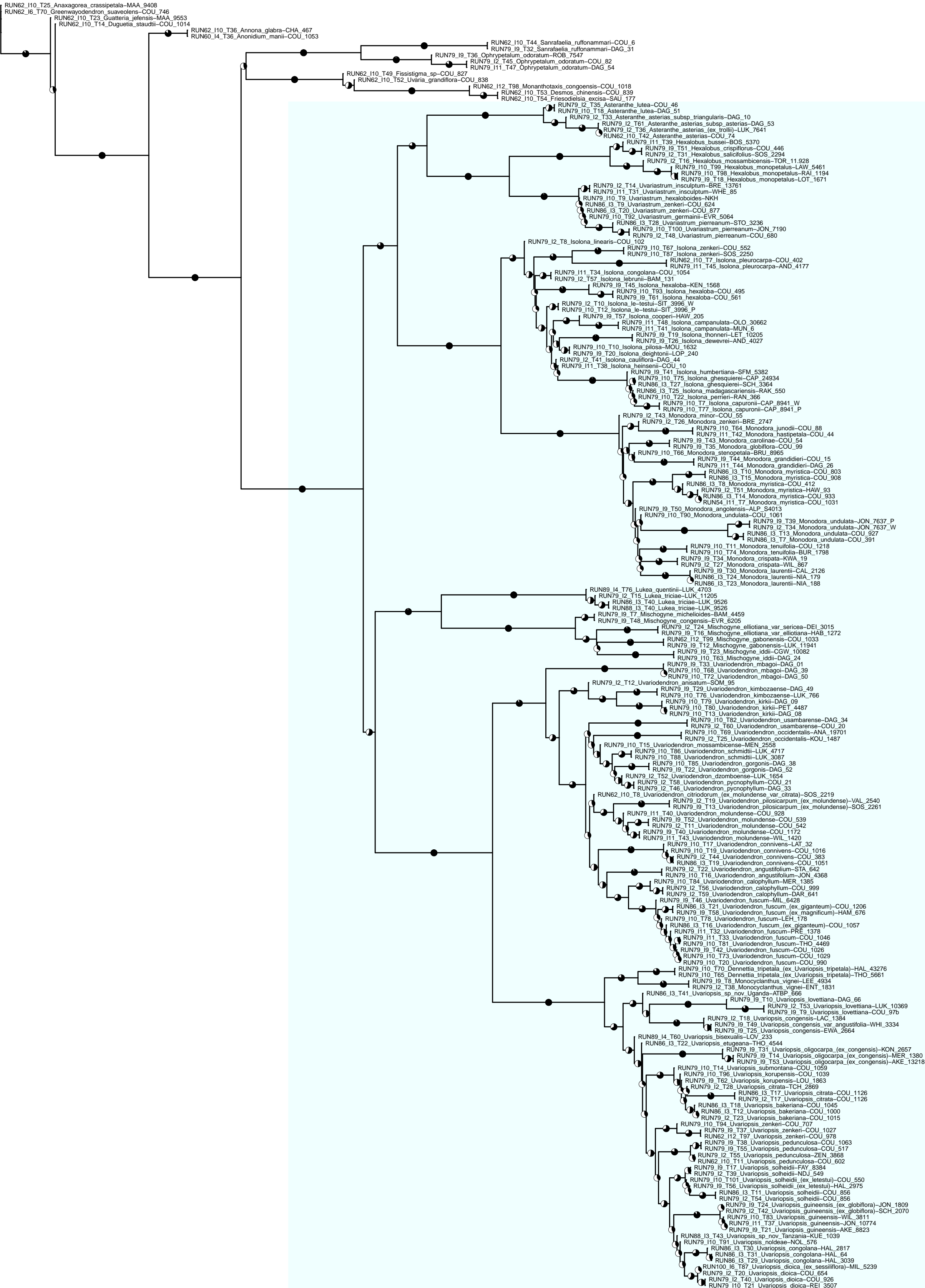

Supplement: Supplementary material 1 — Phylogenetic tree of the Monodoreae inferred with ASTRAL, based on 334 nuclear genes trees [file phytokeys-233-001_article-103096__-s001.pdf]

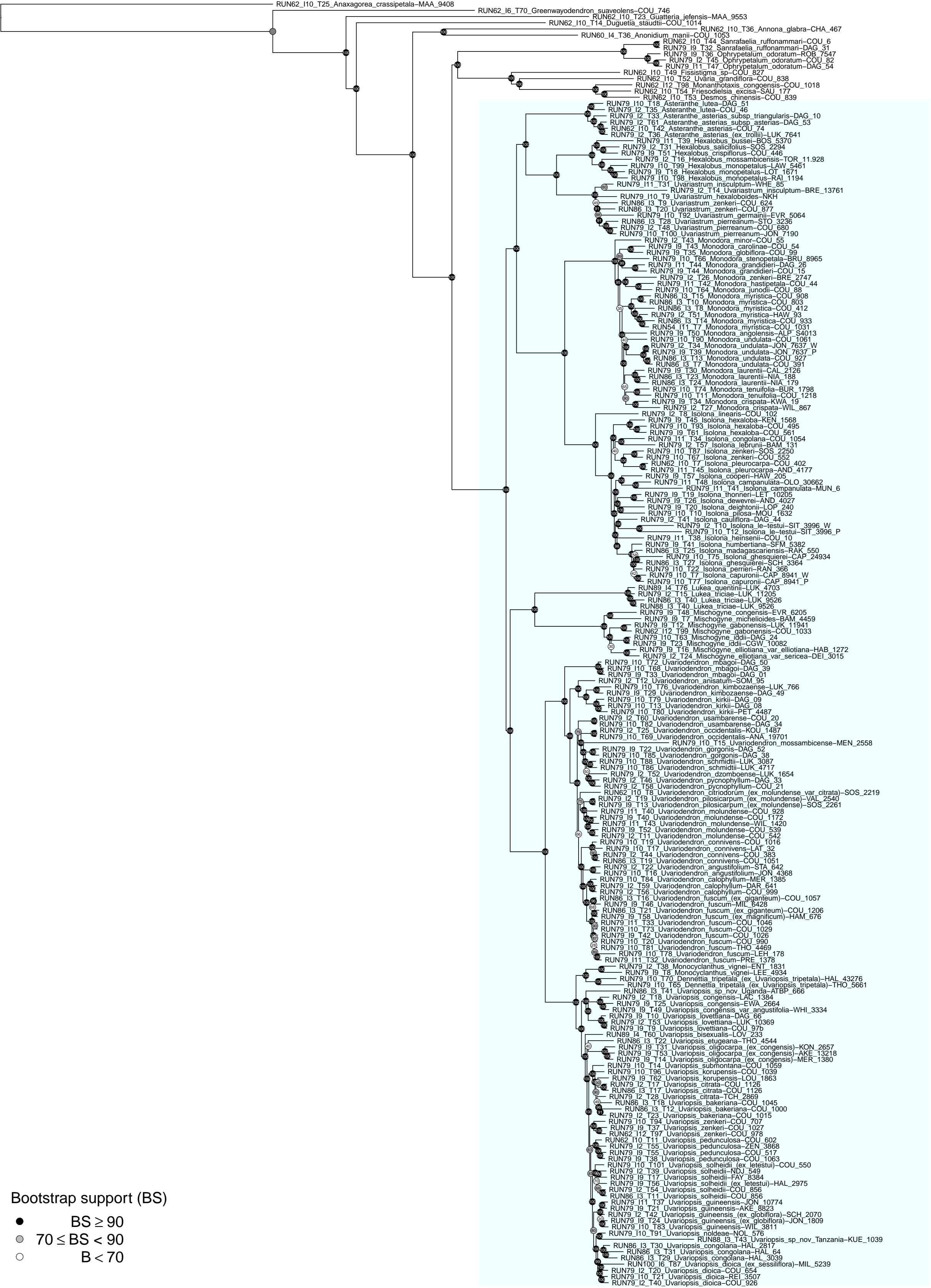

Bootstrap support (BS)

- BS ≥ 90
- 70 ≤ BS < 90
- B < 70

Supplement: Supplementary material 2 — RAxML maximum likelihood tree of the Monodoreae, based on a concatenated supermatrix of 334 nuclear genes. Bootstrap support values (in %) are given at the nodes [file phytokeys-233-001_article-103096__-s002.pdf]
